# Accessing homoleptic neutral and anionic five-coordinate Pr(iv) siloxide complexes

**DOI:** 10.1039/d5sc05500h

**Published:** 2025-10-03

**Authors:** Pragati Pandey, Megan Keener, Thayalan Rajeshkumar, Rosario Scopelliti, Andrzej Sienkiewicz, Ivica Zivkovic, Laurent Maron, Marinella Mazzanti

**Affiliations:** a Group of Coordination Chemistry, Institut des Sciences et Ingénierie Chimiques, École Polytechnique Fédérale de Lausanne (EPFL) CH-1015 Lausanne Switzerland marinella.mazzanti@epfl.ch; b X-ray Diffraction and Surface Analytics Platform, Institut des Sciences et Ingénierie Chimiques, École Polytechnique Fédérale de Lausanne (EPFL) CH-1015 Lausanne Switzerland; c Laboratory for Quantum Magnetism, Institute of Physics, École Polytechnique Fédérale de Lausanne (EPFL) CH-1015 Lausanne Switzerland; d ADSresonances Sarl CH-1920 Martigny Switzerland; e Laboratoire de Physique et Chimie des Nano-objets, Institut National des Sciences Appliquées 31077 Toulouse France laurent.maron@irsamc.ups-tlse.fr

## Abstract

Structurally characterized Pr(iv) complexes are limited to four examples because the ligands and reaction conditions capable of stabilizing Pr(iv) remain elusive. Here we identify reaction conditions allowing the synthesis of Pr(iv) complexes that were originally thought difficult to isolate. The Pr(iv) complexes of the tris(*tert*-butoxy)siloxide (–OSi(O^*t*^Bu)_3_) and triphenylsiloxide (–OSiPh_3_) ligands, [Pr^iv^(OSi(OtBu)_3_)_4_] (2-Pr^OtBu^), [MPr^iv^(OSiPh_3_)_5_] (5M-Pr^Ph^) (M = K, Cs), and [KDB18C6][Pr^iv^(OSiPh_3_)_5_], (5[KDB18C6-Pr^Ph^]) were isolated and fully characterized upon the oxidation of the tetrakis and pentakis(siloxide)praseodymium(iii) ate complexes, [KPr^iii^(OSi(OtBu)_3_)_4_] (1-Pr^OtBu^) and [M_2_Pr^iii^(OSiPh_3_)_5_] (4M-Pr^Ph^) (M = K, Cs), using the thianthrene radical cation tetrafluoroborate oxidant, thiaBF_4_. The crucial role of reagents and reaction conditions, like thiaBF_4_ over the magic blue oxidant and non-coordinating over coordinating solvents, are demonstrated for the isolation of high valent Pr(iv) complexes. The solid state structural and electrochemical properties were studied and further augmented with theoretical calculations. The Pr(iv) oxidation state was further confirmed by electron paramagnetic resonance (EPR) and SQUID magnetometry measurements. Complexes 5M-Pr^Ph^ and 5[KDB18C6]-Pr^Ph^ provide the first example of anionic Ln(iv) complexes demonstrating the possibility of accessing charged Pr(iv) complexes as a tool to manipulate the redox potential and therefore access to more stable complexes with the same ligand.

## Abstract 2

Anionic Ln(iv) complexes were synthesised by tuning the reaction condition, demonstrating the possibility of accessing charged Pr(iv) complexes as a tool to manipulate the redox potential and therefore lead to more stable complexes.

## Graphical abstract

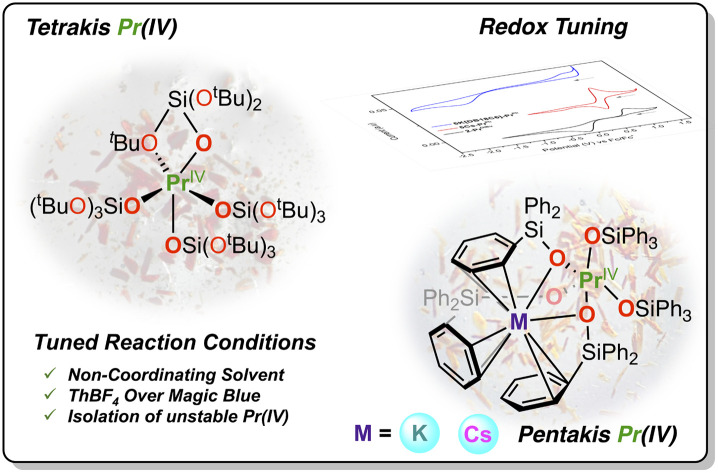

## Introduction

The redox chemistry of the f elements has seen remarkable expansion in the last 20 years with new oxidation states having been identified for both lanthanides and actinides.^1–14^ Molecular compounds containing lanthanides (Ln) in the + ii oxidation state were isolated for all Ln ions (except promethium), despite the fact that the predicted reduction potentials suggested that some of these complexes would be too unstable to isolate and their chemistry too difficult to control.^1^ The discovery of ligands and reaction conditions capable of stabilizing the Ln(ii) oxidation state in reactive intermediates or stable complexes has resulted in attractive magnetic properties^15–18^ and high reactivity towards small molecule activation.^19–29^ In contrast, molecular complexes of Ln's in the +IV oxidation state were for a long time limited to Ce, which has an accessible Ln(iii)/Ln(iv) redox couple that has a broad range of applications.^30–40^ The first molecular complex of Tb(iv) was isolated and characterized by our group in 2019, using the redox-innocent tris(*tert*-butoxy)siloxide (–OSi(O^*t*^Bu)_3_) ligand,^4^ which yielded a five-coordinate tetrakis Tb(iv) complex, [Tb^IV^(OSi(OtBu)_3_)_4_]. Several other molecular compounds of Tb(iv) have been isolated since, using monodentate and polydentate siloxide ligands^41–44^ or imidophosphorane ligands.^14^ The calculated^45^ Pr(iv)/Pr(iii) oxidation potential (+3.4 V *vs.* NHE) is very close to the potential reported for the Tb(iv)/Tb(iii) couple (+3.3 V *vs.* NHE), suggesting similar thermodynamic accessibility when using similar supporting ligands and oxidizing conditions. However, only one ligand system has been identified so far that allowed the isolation of isostructural molecular complexes of Pr(iv) and Tb(iv) in the solid state.

**Scheme 1:**
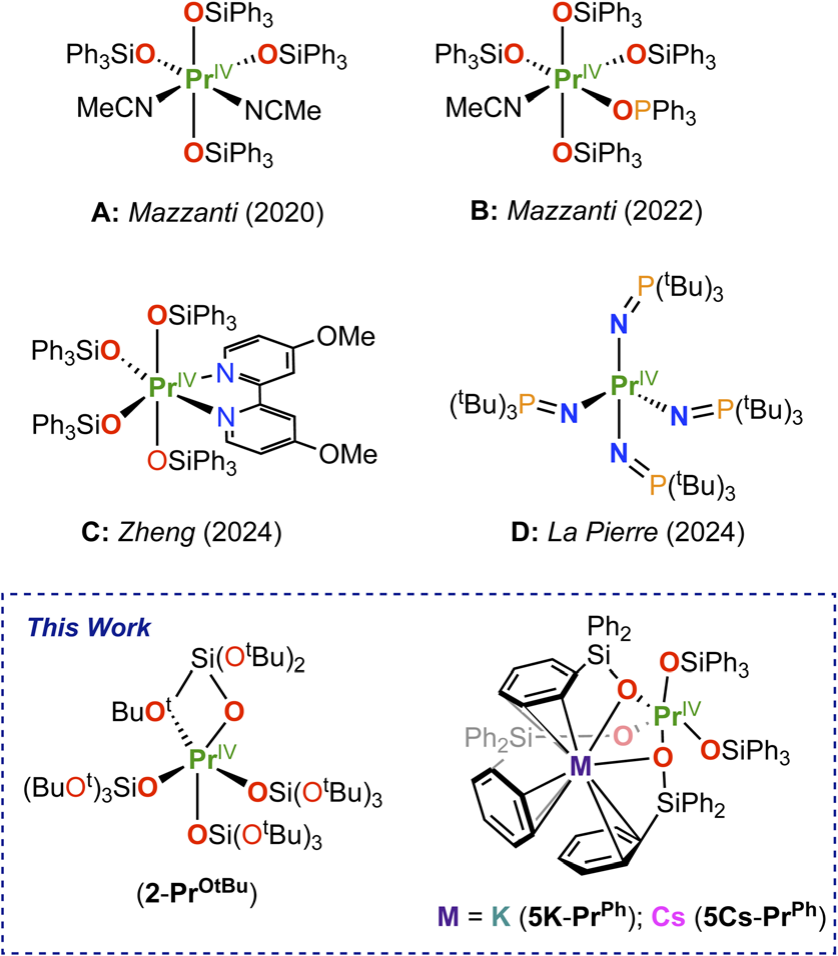
Previously isolated and crystallographically characterized Pr(iv) molecular complexes (A–D) and this work.

Notably, in 2020 we reported the solid state molecular structure of a molecular complex of Pr(iv), [Pr^IV^(OSiPh_3_)_4_(CH_3_CN)_2_] (Scheme 1A), that was prepared using the triphenylsiloxide (–OSiPh_3_) supporting ligand by oxidation in acetonitrile solution of the trivalent analogue, [KPr^iii^(OSiPh_3_)_4_], using the strong oxidant, tris(4-bromophenyl)amminium salt, [N(C_6_H_4_Br)_3_][SbCl_6_], also known as “*magic blue*”.^5^ A similar procedure allowed the isolation of the isostructural Tb(iv) complex, [Tb^iv^(OSiPh_3_)_4_(CH_3_CN)_2_].^41^ The oxidation of the Tb(iv) complex was found to occur at a potential only +0.18 V more than the Pr(iv), as anticipated by calculated redox potentials. We also found that one solvent molecule can be replaced in both Tb(iv) and Pr(iv) complexes by a phosphineoxide ligand (Scheme 1B) leading to higher stability.^46^ Further, Zheng and coworkers showed that both coordinated solvent molecules can be replaced^43,47^ by bidentate ligands (Scheme 1C), increasing the solution stability.

Despite the redox accessibility of the imidophosphorane Pr(iv) analogue (*E*_ox_ = −0.72 V *vs.* Fc/Fc^+^) of the isolated Tb(iv) molecular complex, [Tb^iv^(NP(1,2-bis-tBu-diamidoethane)(NEt_2_))_4_],^14^ the Pr(iv) species generated *in situ* was reported to be not stable at −35 °C or above and could not be isolated in the solid state.^48^ The isolation of a thermally stable four-coordinate molecular Pr(iv) complex^8^ (Scheme 1D) required the use of a different imidophosphorane (NP^t^Bu_3_) ligand that significantly shifted the oxidation potential towards more negative values (−1.37 V *vs.* Fc/Fc^+^).

Therefore, redox accessibility is not the sole parameter that should be considered when pursuing the isolation of Ln(iv) complexes. Indeed, preventing possible decomposition pathways appears to be key for the isolation of Pr(iv) complexes in the solid state, which remain limited to four examples (Scheme 1).^5,46,47,49^ Considering that only four- and six-coordinate neutral complexes of Pr(iv) have been isolated, we set out to explore alternative synthetic routes to isolate five-coordinate Pr(iv) complexes. Notably, previous attempts to isolate in the solid state five-coordinate Pr(iv) complexes analogues of the [Tb^IV^(OSi(O^t^Bu)_3_)_4_] were not successful.^5^

Here, by choosing the appropriate conditions that prevent rapid decomposition pathways, we synthesised and characterized four examples of homoleptic five-coordinate Pr(iv) complexes that were previously thought to be difficult to isolate.

## Result and discussion

### Synthesis and structural characterization

#### Tetrakis-tertbutoxylsiloxide Pr(iii/iv) complexes

We previously reported that all attempts to isolate a tertbutoxylsiloxide complex by oxidation of the Pr(iii) complex, [KPr^iii^(OSi(O^t^Bu)_3_)_4_] (1-Pr^OtBu^), with *magic blue* only resulted in the isolation of a Pr(iii) decomposition product, showing the loss of a siloxide ligand and chloride coordination.^5^ The molecular structure of the decomposition product, [{Pr^iii^(OSi(O^t^Bu)_3_)_3_}_2_(μ-Cl)_3_(μ-K)_3_], suggested that chloride binding to the praseodymium is likely to provide a decomposition pathway for the putative Pr(iv) intermediate species. In order to prevent the unwanted halide abstraction, we reasoned that thianthrene tetrafluoroborate (thiaBF_4_) would be a convenient and innocent oxidizing agent (*E*_ox_ = 0.86 V *vs.* Fc/Fc^+^), which is also stronger than magic blue (*E*_ox_ = 0.67 V *vs.* Fc/Fc^+^). Notably, thiaBF_4_ would avoid the coordinating halides present in AgI or magic blue and would allow the oxidation reactions in non-coordinating solvents. The non-coordinating nature of the BF_4_^−^ counter anion and the formation of highly soluble thianthrene side products would also facilitate the isolation of a molecular complex of Pr(iv). When carrying out this work, Zheng and coworkers reported the successful use of thiaBF_4_ as an oxidizing agent for the synthesis of molecular and supramolecular Tb(iv) complexes.^44^

**Scheme 2:**
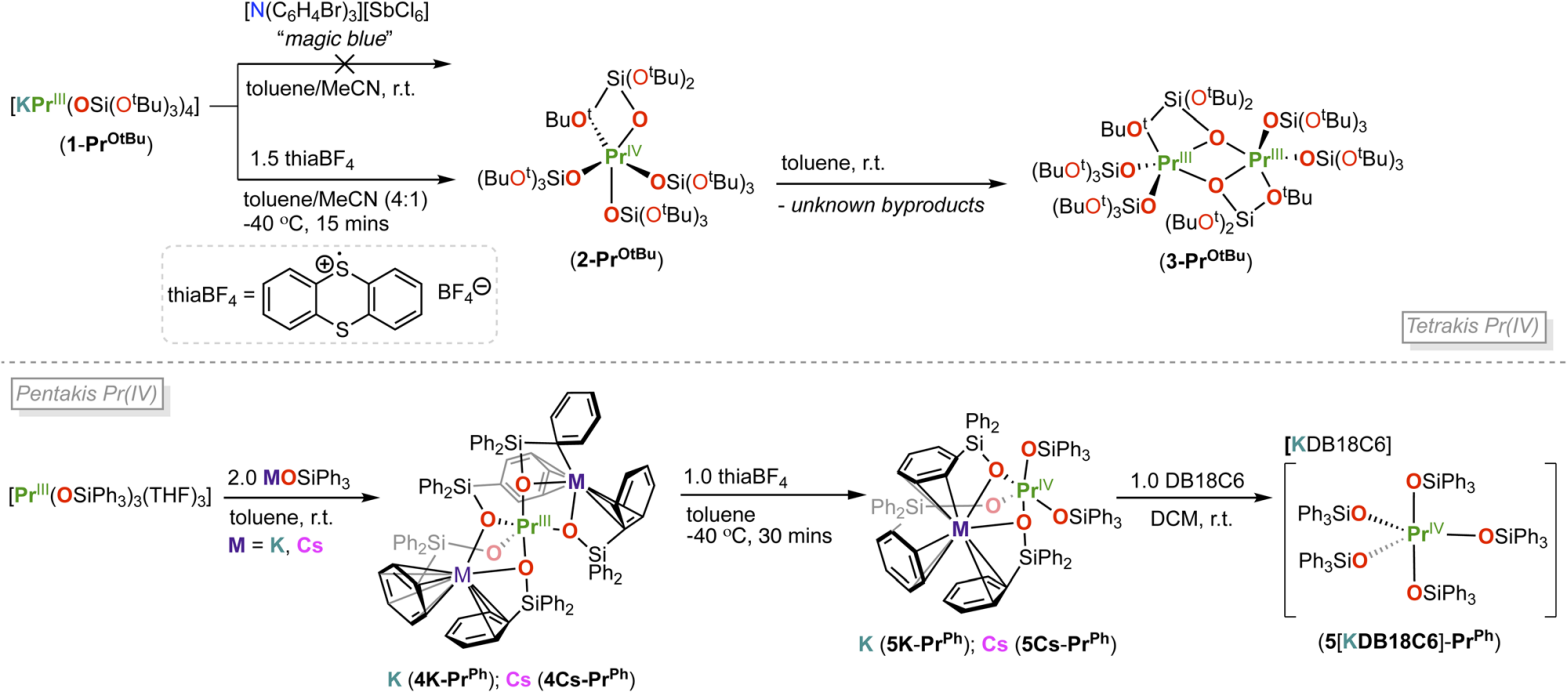
Top: synthesis and decomposition of [Pr^IV^(OSi(O^t^Bu)_3_)_4_], 2-Pr^OtBu^. Bottom: synthesis of complexes, [M_2_Pr^iii^(OSiPh_3_)_5_], 4M-Pr^Ph^, [MPr^iv^(OSiPh_3_)_5_], 5M-Pr^Ph^, (M = K, Cs) and 5[KDB18C6]-Pr^Ph^.

First, we found that the addition of thiaBF_4_ (1.5 equiv.) to 1.0 equiv. of the Pr(iii) complex, [KPr(OSi(OtBu)_3_)_4_], 1-Pr^OtBu^, in acetonitrile/toluene (4 : 1) at −40 °C, resulted in an immediate color change and formation of an orange-brown suspension (Scheme 2 (top)). Isolation of the orange-brown solid and extraction into *n*-hexane yielded a red solid identified as [Pr^IV^(OSi(OtBu)_3_)_4_] (2-Pr^OtBu^) in 48% yield. Single crystals suitable for X-ray diffraction studies were obtained from a saturated solution of 2-Pr^OtBu^ in *n*-hexane at −40 °C after 24 h.

**Fig. 1:**
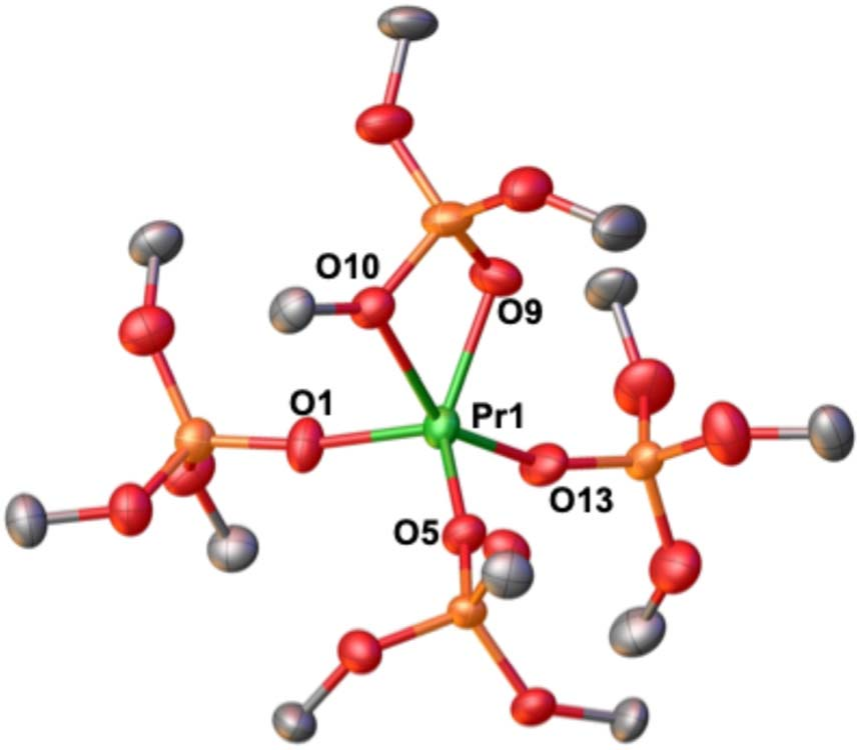
Molecular structure of [Pr(OSi(OtBu)_3_)_4_], 2-Pr^OtBu^, with thermal ellipsoids drawn at the 50% probability level. Hydrogen atoms and methyl groups on the siloxide ligands have been omitted for clarity.

The solid-state molecular structure of complex 2-Pr^OtBu^ (Fig. 1) is isostructural to the previously reported Tb(iv) complex, [Tb^IV^(OSi(OtBu)_3_)_4_] (2-Tb^OtBu^). Both Ln(iv) ions are 5-coordinated by three *κ*_1_-OSi(OtBu)_3_ and one *κ*_2_-OSi(OtBu)_3_ ligands despite a 0.09 Å difference in their Ln(iv) ionic radii (6-coordinate Shannon radii: Pr(iv), 0.85 Å; Tb(iv), 0.76 Å).^50^ The same structure was also reported for the analogous Ce(iv) complex.^51^ The 0.16 Å difference in (Ln–O)_avg_ bond distances between 2-Pr^OtBu^ and the previously^5^ reported 5-coordinate Pr(iii) complex 1-Pr^OtBu^ (Table 1), is comparable to the 0.15 Å difference in the ionic radii determined for Pr(iii) and Pr(iv) 6-coordinate compounds.^50^ The average Pr–O_siloxide_ distances in 2-Pr^OtBu^ (2.11(4) Å) are comparable to those reported for [Pr^IV^(OSiPh_3_)_4_(CH_3_CN)_2_] (2.10(1) Å)^5^ (Table 1).

**Table 1:** Selected bond lengths (Å) and angles (deg) of five-coordinate Ln(iv) complex

	2-Pr^OtBu^	[Tb^IV^(OSi(OtBu)V_3_)_4_]	[Pr^IV^(OSiPh_3_)_4_(CH_3_CN)_2_]	5K-Pr^Ph^	5[KDB18C6]-Pr^Ph^	5Cs-Pr^Ph^
Ln–O_siloxide_ range	2.084(4)–2.147(4)	2.023(3)–2.093(3)	2.088(4)–2.121(4)	2.098(6)–2.230(4)	2.109(4)–2.173(4)	2.134(8)–2.208(7)
Ln–OtBu	2.563(4)	2.474(3)	—	—	—	—
Ln–N(MeCN)	—	—	2.599(6)–2.603(6)	—	—	—

**Fig. 2:**
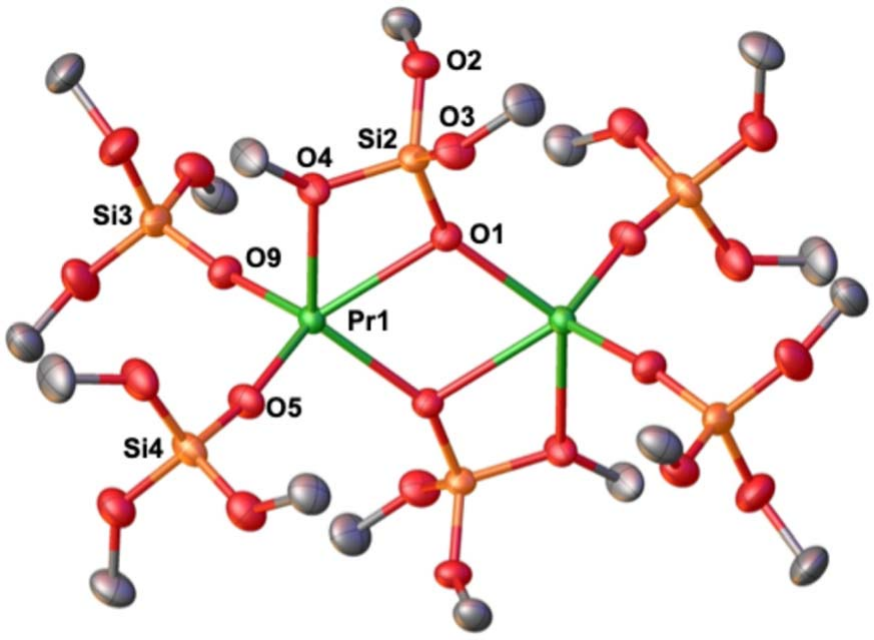
Molecular structure of [Pr(OSi(OtBu)_3_)_3_]_2_, 3-Pr^OtBu^, with thermal ellipsoids drawn at the 50% probability level. Hydrogen atoms and methyl groups on the siloxide ligands have been omitted for clarity.

The ^1^H NMR spectrum of 2-Pr^OtBu^ in tol-*d*_*8*_ and CD_2_Cl_2_ recorded at −40 °C showed the presence of a single broad resonance for the –OSi(OtBu)_3_ ligands at *δ* 1.85 and 1.53 ppm respectively (Fig. S4 and S5). Complex 2-Pr^OtBu^ is stable in tol-*d*_*8*_ and CD_2_Cl_2_ solution at −40 °C for up to one week but starts to decompose when the mixture is brought to room temperature, resulting in the dimeric Pr^iii^ complex, [Pr^iii^(OSi(OtBu)_3_)_3_] (3-Pr^OtBu^), and unknown byproducts, identified by the ^1^H NMR spectroscopy (Fig. S9). Single crystals of 3-Pr^OtBu^ were isolated from a toluene solution of 2-Pr^OtBu^ left at room temperature for 18 hours (Scheme 2 (top), Fig. 2). The solid-state molecular structure of the Pr(iii) decomposition product, 3-Pr^OtBu^, shows the presence of only three siloxide per Pr(iii) centre, while the fate of the fourth siloxide ligand remains unknown.

Pr(iv) complexes comprising siloxide ligands were reported to exhibit characteristic absorption bands in the UV-visible region due to parity-allowed 4f–5d transitions. In accordance with these observations, we next examined the electronic absorption spectra of 1-Pr^OtBu^, 2-Pr^OtBu^, and the KL^OtBu^ ligand in toluene. The electronic absorption spectra of 1-Pr^OtBu^ and KL^OtBu^ ligand showed a sharp absorption in the UV range at a *λ*_max_ = 276 nm. The spectrum of 2-Pr^OtBu^ displayed a characteristic signal in the UV-visible region with a maximum absorption wavelength (*λ*_max_) at 378 nm (Fig. S49 and S50).

#### Pentakis-triphenylsiloxide Pr(iii/iv) complexes

Considering the successful outcome of the oxidation of the complex 1-Pr^OtBu^, we decided to pursue the synthesis of homoleptic five-coordinate Pr(iv) complexes that were previously unsuccessful using magic blue in THF or MeCN solutions. In particular, we pursued the synthesis of a pentakis-siloxide Pr(iv) complex, with the goal of evaluating differences in redox properties by addition of a fifth anionic ligand in the presence of different cations. It should be noted that all Pr(iv) complexes isolated so far are neutral.

We previously reported that pentakis-Ln(iii) complexes^46^ of the –OSiPh_3_ ligand could only be prepared in apolar solvents (toluene), while the fifth siloxide ligand rapidly de-coordinates in THF or MeCN preventing the effective use of magic blue as oxidizing agent. Therefore, we set out to use thiaBF_4_ as an oxidant in toluene to isolate a five coordinate Pr(iv) complex of the –OSiPh_3_ ligand.

First, the previously reported [Pr^iii^(OSiPh)_3_)_3_(THF)_3_]^5^ complex was reacted for an hour with 2.0 equiv. of KOSiPh_3_ in toluene at room temperature. Single crystals of the pentakis Pr(iii) complex, [K_2_Pr^iii^(OSi(Ph)_3_)_5_] (4K-Pr^Ph^), suitable for X-ray diffraction analysis were isolated in 82% yield from a saturated toluene solution stored at −40 °C. Complex [Cs_2_Pr^iii^(OSiPh_3_)_5_] (4Cs-Pr^Ph^), was isolated in 86% yield by analogous reaction conditions as 4K-Pr^Ph^, but CsOSiPh_3_ was used. Additionally, the analogous Ce(iii) penta-coordinated analogue, [K_2_Ce^iii^(OSiPh_3_)_5_], (4K-Ce^Ph^), was also prepared in 78% yield, and single crystals were grown from toluene solution layered with *n*-hexane at room temperature over 7 days. The quality of the XRD crystal structure allowed to determine the connectivity but does not allow to discuss metric parameters.

**Fig. 3:**
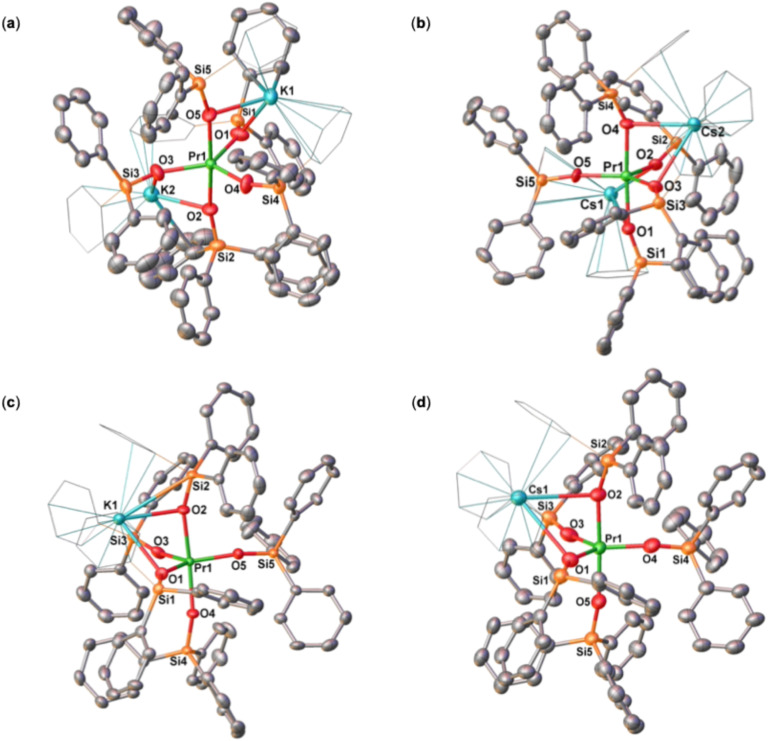
Molecular structure of complexes, (a) [K_2_Pr(OSiPh)_3_)_5_], 4K-Pr^Ph^, (b) [Cs_2_Pr(OSiPh)_3_)_5_], 4Cs-Pr^Ph^, (c) [KPr(OSiPh)_3_)_5_], (5K-Pr^Ph^), and (d) [CsPr(OSiPh)_3_)_5_], (5Cs-Pr^Ph^), with thermal ellipsoids drawn at the 50% probability level. Phenyl groups bound to the alkali ions are drawn as wireframe for clarity. Hydrogen atoms on the siloxide ligands, residual solvent molecules and disordered phenyl groups have been omitted for clarity.

The solid-state molecular structure of complexes 4K-Pr^Ph^, 4Cs-Pr^Ph^ and 4K-Ce^Ph^ (Fig. 3a, b and S48) are isostructural to that of the previously reported Nd analogue.^46^ The solid-state molecular structures of 4K-Pr^Ph^, 4Cs-Pr^Ph^ and 4K-Ce^Ph^ show a five-coordinated Ln(iii) metal center bound by five monodentate triphenylsiloxide ligands in a distorted trigonal bipyramidal geometry. The presence of five triphenylsiloxide ligands around the Pr(iii) ion in 4K-Pr^Ph^ does not significantly affect the Pr–O_siloxide_ distances (2.199(4)–2.315(3) Å) compared to those found in the six-coordinate tetrakis complex, [KPr(OSiPh_3_)_4_(THF)_3_] (2.248(9)–2.304(9) Å). Interestingly the Pr–O_siloxide_ bond distances (2.279(11)–2.318(9) Å)) for the 4Cs-Pr^Ph^ are similar to those found for 4K-Pr^Ph^ (Pr–O_siloxide_: 2.199(4)-2.315(3)Å). The –OSiPh_3_ ligands bind the two potassium (K) cations in close proximity to the Pr(iii) metal center through the anionic oxygen and cation–π interactions with the phenyl groups. The two K cations lie at a distance from the Pr(iii) center of 3.675(1) Å for K1 and 3.764(1) Å for K2. The Cs cation lies at a significantly longer distance from the Pr^iii^ center (Pr(iii) –Cs(1) = 4.073(1) and Pr(iii) –Cs(2) = 4.092(1) Å) compared to the K ion in 4K-Pr^Ph^ (3.675(1) Å for K1 and 3.764(1) Å for K2) probably due to steric hindrance of the larger Cs cation. These results suggest that although the lower Lewis acidity of the Cs cation compared to the K ion should result in a stronger Pr^iii^-siloxide interaction, due to the larger size of the Cs, the Pr-siloxide bonding interactions remain similar.

With the electron-rich pentakis-siloxide complexes 4M-Pr^Ph^ (M = K, Cs) in hand, we next investigated their oxidation reactions to yield tetravalent pentakis Pr(iv) complexes. Treatment of a cold toluene suspension of 4M-Pr^Ph^ (K, Cs) with 1.0 equivalent of thiaBF_4_ at −40 °C, allowed the isolation of the Pr(iv) complexes, [KPr(OSi(Ph)_3_)_5_] (5K-Pr^Ph^) and [CsPr(OSi(Ph)_3_)_5_] (5Cs-Pr^Ph^) in 51% and 54% yields, respectively (Scheme 2 (bottom)).

Dark red single crystals of complexes 5M-Pr^Ph^ (K, Cs) were isolated from a saturated toluene solution layered with *n*-hexane at −40 °C after 24 hours.

The solid-state molecular structure of complexes 5M-Pr^Ph^ (K, Cs) displays a distorted trigonal bipyramidal geometry around the Pr(iv) center (Fig. 3c and d). The Pr(iv) ion in 5K-Pr^Ph^ is coordinated by five anionic –OSiPh_3_ ligands with Pr–O_siloxide_ distances ranging from 2.098(6) to 2.230(4) Å, slightly longer than those found in the neutral six-coordinated tetrakis complex, [Pr^IV^(OSiPh_3_)_4_(CH_3_CN)_2_] (2.088(4)–2.121(4) Å). The presence of the longer bond distances is most likely the result of steric interactions between the five siloxide ligands. The Pr(iv)–O_siloxide_ distances in 5Cs-Pr^Ph^ are slightly shorter (2.134(8)–2.208(7) Å) compared to those in 5K-Pr^Ph^, most likely due to the presence of the less electron-withdrawing Cs cation compared to K. The K cation lies in close proximity of the Pr(iv) center (3.805(2) Å) and bridges the siloxide ligands through the anionic oxygen and through cation–π interactions with the siloxide phenyl groups. The Cs cation lies at a much larger distance (Cs–Pr = 4.120(2) Å).

**Fig. 4:**
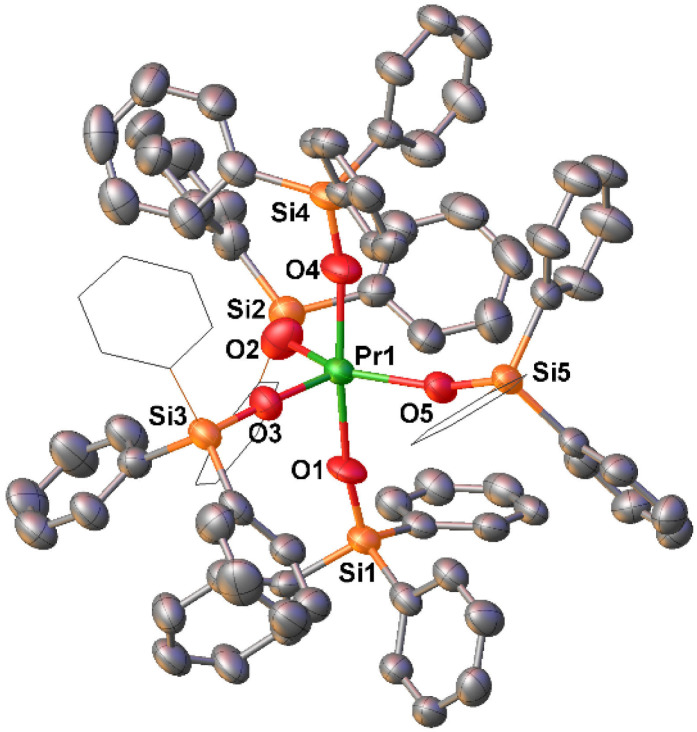
Molecular structure of [KDB18C6][Pr^iv^(OSiPh_3_)_5_], 5[KDB18C6]-Pr^Ph^, with thermal ellipsoids drawn at the 50% probability level. Phenyl groups of siloxide ligands in equatorial position are drawn as wireframe for clarity. Hydrogen atoms and the [KDB18C6] counterion have been omitted for clarity.

In hopes of evaluating inner-versus outer sphere cation effects, we found addition of 1.0 equiv. dibenzo-18-crown-6 (DB18C6) to a dichloromethane (DCM) solution of 5K-Pr^Ph^ allowed the isolation of orange coloured single crystals of [KDB18C6][Pr^iv^(OSiPh_3_)_5_], (5[KDB18C6]-Pr^Ph^) in 72% yield upon layering with *n*-hexane at −40 °C overnight. The solid state molecular structure of 5[KDB18C6]-Pr^Ph^ shows an ion pair with an anionic five coordinated Pr(iv) complex and a potassium cation coordinated to DB18C6 (Fig. 4). For 5[KDB18C6]-Pr^Ph^ the Pr–O_siloxide_ distances ranging from 2.109(4)–2.173(4) Å; are slightly shorter than in 5K-Pr^Ph^ and closer to those found in 5Cs-Pr^Ph^ complex, in agreement with stronger bonding of five anionic ligands with Pr(iv) in the absence of inner-sphere cation or in the presence of a weakly coordinating Cs ion.

The electronic absorption spectra of 5K-Pr^Ph^, 5Cs-Pr^Ph^ and 5[KDB18C6]-Pr^Ph^ measured in DCM (0.15 mM) at room temperature showed a maximum absorption wavelengths (*λ*_max_) at 371, 364 and 355 nm in the UV-visible region characteristic of Pr(iv) (Fig. S53–S55).

To evaluate the solution stability of 5M-Pr^Ph^, we measured the time dependent ^1^H NMR spectra of 5M-Pr^Ph^ at −40 °C and room temperature in CD_2_Cl_2_ and tol-*d*_*8*_. At −40 °C, we found both 5M-Pr^Ph^, complexes are stable for up to 14 days (Fig. S19, and S29). However, at room temperature, the formation of decomposition products begins after two hours (Figure S17, S22, S30 and S31), with full decomposition after 3 days. Further, it was observed that at −40 °C and at room temperature, the ^1^H NMR spectra of 5K-Pr^Ph^ and 5Cs-Pr^Ph^ are similar irrespective of the deuterated solvent suggesting that in both cases the cation remains bound in solution on the NMR time scale (Fig. S35 and S36). The presence of the bound K cation was confirmed by measuring the ^1^H NMR spectrum of 5K-Pr^Ph^ in the presence of 1.0 equiv. of DB18C6 in CD_2_Cl_2_ at −40 °C and room temperature. The addition of DB18C6 results in the presence of three separate signals which are significantly shifted compared to those found for 5K-Pr^Ph^ and 5Cs-Pr^Ph^.

### Electrochemistry

**Fig. 5:**
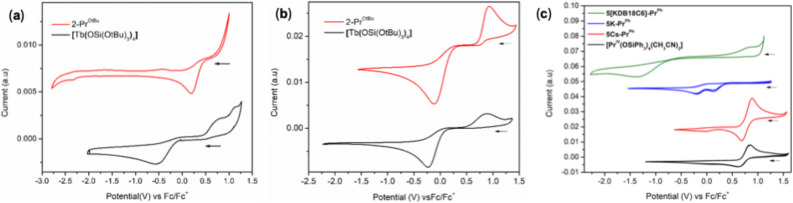
Cyclic voltammograms of 2-Pr^OtBu^ and [Tb^IV^(OSi(OtBu)_3_)_4_] complexes in (a) THF and (b) DCM. (c) Cyclic voltammograms of complexes, 5K-Pr^Ph^, 5Cs-Pr^Ph^, 5[KDB18C6]-Pr^Ph^ and [Pr^IV^(OSiPh_3_)_4_(CH_3_CN)_2_] in DCM. Electrolyte 0.1 M [NBu_4_][B(C_6_F_5_)_4_]; [analyte] = 0.005 M; υ = 100 mV s^−1^.

Next, electrochemical studies were performed to evaluate the redox properties of the Pr(iv) complexes. Interestingly, the cyclic voltammogram (CV) of 2-Pr^OtBu^ obtained in THF and DCM solvents displayed different redox events (Fig. 5a and b). The CV in THF resulted in an irreversible reduction event at *E*_pc_ = 0.20 V *versus* Fc/Fc^+^. In contrast, for the reported Tb(iv) analogue, [Tb^IV^(OSi(OtBu)_3_)_4_],^4^ both oxidation and reduction events in THF (*E*_pa_ = 0.83 V and *E*_pc_ = −0.55 V *versus* Fc/Fc^+^) were reported (Fig. 5a), probably as a result of decreased ligand lability compared to the Pr(iv) complex.

However, a contrasting behaviour was observed in non-coordinating DCM, where 2-Pr^OtBu^ showed reduction and oxidation events with *E*_pa_ = 0.93 V and *E*_pc_ = −0.12 V *versus* Fc/Fc^+^. The values measured for 2-Pr^OtBu^ are shifted to more positive potentials compared to the values measured in DCM for [Tb^IV^(OSi(OtBu)_3_)_4_] (*E*_pa_ = 0.89 V and *E*_pc_ = −0.23 V *versus* Fc/Fc^+^). The large Δ*E* between anodic and cathodic peaks in both complexes most likely originates from the different binding modes of the –OSi(OtBu)_3_ ligands in the absence of coordinating cations in non-coordinating solvents for the Ln(iii) (all *k*_1_-siloxides) and for the Ln(iv) (3 *k*_1_ and 1 *k*_2_ siloxide) complexes as already reported for the cerium analogue.^34^ Notably, upon addition of 2.2.2-cryptand to a solution of 1-Pr^OtBu^, we isolated crystals of the [K(2.2.2cryptand)][Pr^iii^(*k*_1_-OSi(OtBu)_3_)_4_] complex where all siloxides are binding in *k*_*1*_ mode (See SI and Fig. S42). The oxidation potential is only 0.04 V more positive for 2-Pr^OtBu^ and is in line with the calculated difference of 0.1 V between Pr(iv) and Tb(iv) ions.

The redox properties of 5M-Pr^Ph^ (K, Cs) were also studied by cyclic voltammetry in DCM (Fig. 5c). The cyclic voltammogram of 5K-Pr^Ph^ displays two irreversible reduction events at *E*_pc1_ = 0.13 V and *E*_pc2_ = −0.19 *vs.* Fc/Fc^+^ in DCM, most likely due to chemical equilibria resulting from the strong binding of the K cation. However, removal of K with DB18C6 resulted in the appearance of an oxidation (0.86 V) and reduction event (−1.33 V, Δ*E* = 2.19), resulting in a significantly greater peak separation for 5[KDB18C6]-Pr^Ph^. The large peak separation observed for 5[KDB18C6]-Pr^Ph^ is most likely due to changes in the coordination sphere concurrent with changes in the Pr(iv) oxidation state and ion-pair formation effects occurring in the absence of coordinating cations. Furthermore, a quasi-reversible redox event was recorded for the Cs complex in DCM (*E*_pc_ = 0.68 V and *E*_pa_ = 0.89 *vs.* Fc/Fc^+^ in DCM). The oxidation potential measured for 5Cs-Pr^Ph^ is 0.04 V less positive than that measured for 2-Pr^OtBu^. A similar shift had also been reported for [Tb^IV^(OSi(OtBu)_3_)_4_] (ref. 4) compared to [Pr^IV^(OSiPh_3_)_4_(CH_3_CN)_2_].^5^ The higher reversibility and the shift of the reduction event to a more positive potential for complex 5Cs-Pr^Ph^ compared to complex 5[KDB18C6]-Pr^Ph^ is most likely due to the presence of bound Cs that facilitates the reduction process.

Surprisingly, the redox potentials measured for 5Cs-Pr^Ph^ are similar to those reported for the six-coordinate Pr(iv) complex, [Pr^IV^(OSiPh_3_)_4_(CH_3_CN)_2_] (*E*_pa_ = 0.85 V and reduction potential *E*_pc_ = 0.62 V *vs.* Fc/Fc^+^), suggesting that the replacement of two acetonitrile ligands by an anionic –OSiPh_3_ ligand does not significantly affect the redox properties (Table 2). The similarity of oxidation potential may be correlated to the presence of an overall weaker Pr(iv)–O_siloxide_ interaction in pentakis complexes due to the steric encumbrance created by the presence of the five bulky ligands. Notably, the Pr–O_siloxide_ distances are significantly elongated in 5Cs-Pr^Ph^ compared to those found in [Pr^IV^(OSiPh_3_)_4_(CH_3_CN)_2_] (Table 1) and 5[KDB18C6]-Pr^Ph^; DFT computational studies corroborated this interpretation (*vide infra*).

**Table 2:** Electrochemical data in V *vs.* Fc^0^/Fc^+^ for Ln(iv) complexes in DCM and/or THF(^a^) and previously reported data measured in THF(^a*^)^4,5^ CH_3_CN (^b^) with scan rate 100 mV s^−1^

Complexes	2-Pr^OtBu^	[Tb^IV^(OSi(OtBu)_3_)_4_]	[Pr^IV^(OSiPh_3_)_4_(CH_3_CN)_2_]	5K-Pr^Ph^	5[KDB18C6]-Pr^Ph^	5Cs-Pr^Ph^
*E*_pc_	0.20^*a*^; −0.12	−0.55^a*^; −0.23	−0.38^a*^; 0.48^b^; 0.62	0.13; −0.19	−1.33	0.68
*E*_pa_	—; 0.93	0.83^a*^; 0.89	0.67^a*^; 0.69^b^; 0.85	—	0.86	0.89
Δ*E*	1.05	1.38^a*^;1.12	1.05^a^; 0.21^b^; 0.23	—	2.19	0.21

### Magnetic measurements

**Fig. 6:**
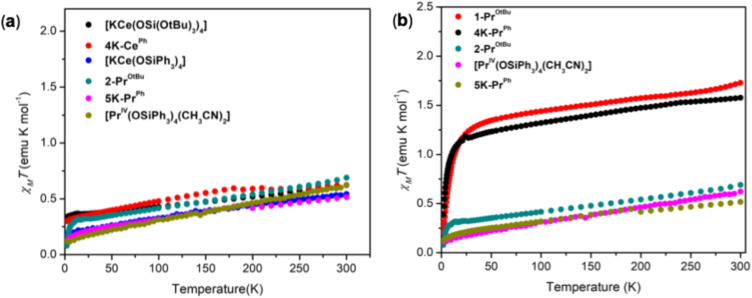
(a) Plot of *χ*_M_*T versus* temperature data for solid samples of 1-Pr^OtBu^ (red) 4K-Pr^Ph^ (black), 2-Pr^OtBu^ (green), 5K-Pr^Ph^ (olive green) and [Pr^IV^(OSiPh_3_)_4_(CH_3_CN)_2_] (pink) collected under an applied magnetic field of 1 T. (b) Plot of *χ*_M_*T versus* temperature data for solid samples of Pr(iv) and Ce(iii) complexes collected under an applied magnetic field of 1 T.

The *χ*_M_*T versus* T data *(χ*_M_ = molar magnetic susceptibility) were measured for 2-Pr^OtBu^ and compared with the previously reported^51^ 4f^1^ isoelectronic cerium analogue [KCe(OSi(OtBu)_3_)_4_] in the temperature range 300 to 2 K (Fig. 6a). The *χ*_M_*T* = 0.689 and 0.623 (at 300 K) and 0.079, 0.331 emu K mol^−1^ (at 2 K) were measured for 2-Pr^OtBu^ and [KCe(OSi(OtBu)_3_)_4_], respectively. The *χ*_M_*T* values are in agreement with predicted values for a 4f^1^ complex (*χ*_M_*T* = 0.8 emu K mol^−1^) and much lower than the 1.7 emu K mol^−1^ found for the 4f^2^ Pr(iii) in 2-Pr^OtBu^ (predicted 1.6 emu K mol^−1^) (Fig. 6b). The *χ*_M_*T versus* T data of pentakis 5K-Pr^Ph^, 4K-Pr^Ph^ and 4K-Ce^Ph^ were also measured in the temperature range 300 to 2 K. and compared with previously reported [Pr^IV^(OSiPh_3_)_4_(CH_3_CN)_2_] complex. The *χ*_M_*T* = 0.516, 0.624 (at 300 K), and 0.126 0.297 emu K mol^−1^ (at 2 K) measured for 5K-Pr^Ph^ and 4K-Ce^Ph^ respectively, are in agreement with the predicted 4f^1^ and are comparable to the values reported for [Pr^IV^(OSiPh_3_)_4_ (CH_3_CN)_2_]^5^ (*χ*_M_*T* = 0.622 emu K mol^−1^ at 300 K) (Fig. 6a). A value of *χ*_M_*T* = 1.57 emu K mol^−1^ was measured for 4K-Pr^Ph^ at 300 K in agreement with the presence of a 4f^2^ ion (Fig. 6b). Similar values of *χ*_M_*T* were also measured for the recently reported Pr(iv) imidophosphorane ([Pr(NP^*t*^Bu_3_)_4_]) (*χ*_M_*T* = 0.64 emu K mol^−1^)^8^ although this value was found to be smaller than the analogous Ce(iii) imidophosphorane ([Cs(THF_*x*_)Ce(NP^*t*^Bu_3_)_4_]) (*χ*_M_*T* = 0.89 emu K mol^−1^). These measurements further support the Pr(iv) oxidation state for 2-Pr^OtBu^ and 5K-Pr^Ph^. The comparative study of five coordinate Pr(iv) siloxide complexes with six and four coordinated analogues suggests that the geometry and coordination number has only marginal effect on the magnetic properties of Pr(iv) complexes with –OSiPh_3_ ligands. These results are in line with the presence of an overall similar magnitude of the crystal field for the Ce(iii) and all Pr(iv) complexes.

To further confirm the Pr(iv) oxidation state of complex 2-Pr^OtBu^, X-band electron paramagnetic resonance (EPR) spectra were measured at 6 K in the solid and solution-state (*n*-hexane) (10 mM). (Fig. S64). Further, EPR spectra of 5M-Pr^Ph^ and 5[KDB18C6]-Pr^Ph^ complexes were also recorded in the solid state at 6 K (Fig. S65–S67). All spectra of 2-Pr^OtBu^, 5M-Pr^Ph^ and 5[KDB18C6]-Pr^Ph^ showed intense hyperfine structure as expected for the Pr^4+^ (4f^1^, *S*_eff_ = ½, I = 5/2) ion^52–54^ that are consistent with the spectra reported by La Pierre and coworkers for complexes containing Pr(iv).^8,48,55^ We also found that the [Pr^IV^(OSiPh_3_)_4_(CH_3_CN)_2_] complex, which we previously reported EPR silent, also showed a characteristic Pr(iv) spectrum when the measurements were carried out at relatively high concentration (20 mM) in acetonitrile at 6 K (Fig. S68).

### Computational studies

To gain further insights, DFT calculations (B3PW91 functional) including dispersion effects have been carried out on complexes 2-Pr^OtBu^ and 5M-Pr^Ph^ (K, Cs). In the latter, the effect of the alkali cation was also investigated by computing the putative [Pr(OSi(Ph)_3_)_5_]^−^, 5-Pr^Ph^ anion. The optimized geometry of 2-Pr^OtBu^ is in very good agreement with the experimental values (see Table S11 in SI) with and without dispersion included. The ground state is a doublet with the unpaired spin located at the Pr center in line with a Pr(iv) complex. The bonds were thus analyzed using Natural Bonding Orbital (NBO) method. The presence of one Pr–O_siloxide_ single bond and three Pr–O_siloxide_ double bonds is found at the first order NBO. These bonds are found to be strongly polarized toward O (90%) and involve df (50–50) atomic orbital at the Pr center. These polarizations explain the Pr–O_siloxide_ Wiberg Bond Index of 0.70–0.77 despite the presence of double bonds. The extra Pr–OtBu interaction is even weaker since only weak donation is found at the second order donor–acceptor level with a WBI of 0.24. For sake of comparison, the Ce(iv) analog of 2-Pr^OtBu^ was optimized and compared since with a closed-shell singlet ground state the oxidation state + iv of Ce is obvious. The geometry and bonding situation are very similar to that described above which is further corroborating the presence of a Pr(iv) center. Finally, the decomposition of 2-Pr^OtBu^ onto 3-Pr^OtBu^ was computed to be marginally favorable (−2.5 kcal.mol^−1^) assuming that the fourth siloxide ligand forms a dimer. The bonding of this Pr(iii) complex is similar to that found for 1-Pr^OtBu^ and its Ce(iii) analog (see SI), where the Pr–O_siloxide_ are only single bond (polarized toward O). This can be easily explained by the fact that due to the increased charge at the metal center in 2-Pr^OtBu^ the Ln–O distances are shorter for the tetravalent state allowing π-type interactions.

A similar analysis was carried out on complexes 5M-Pr^Ph^ (M = K, Cs). In both cases, the optimized geometry obtained for the doublet ground state compare very well with the experimental one (see Table S26 and S30 in SI). The unpaired spin located at the Pr centre is again in line with the presence of Pr(iv) complexes. The bonding analysis is somewhat similar to that observed for 2-Pr^OtBu^ or [Pr^IV^(OSiPh_3_)_4_(CH_3_CN)_2_] (see SI) with the presence of one Pr–O_siloxide_ single bond and four (rather than three) Pr–O_siloxide_ double bonds, strongly polarized toward O (92–94%). However, the associated WBI appears to differ significantly from one alkali atom to the other and from that found for 2-Pr^OtBu^. Therefore, for sake of comparison, the penta-coordinated alkali free anion was computed and analyzed ([5-Pr^Ph^]^−^). In 5Cs-Pr^Ph^ and [5-Pr^Ph^]^−^, the Pr–O_siloxide_ are in the 0.64–0.77 range which is somewhat similar to that found for 2-Pr^OtBu^ and its Ce(iv) analog but also to [Pr^IV^(OSiPh_3_)_4_(CH_3_CN)_2_]. This clearly indicates that the presence of the Cs cation does not perturb the bonding, meaning that Cs does not interact significantly with the oxygen of the siloxide ligands, in line with the electrochemistry observation. In a same way, the similarity between the CVs of [Pr^IV^(OSiPh_3_)_4_(CH_3_CN)_2_] and of 5Cs-Pr^Ph^ and [5-Pr^Ph^]^−^ is explained by the similar bonding situation in the three complexes largely due to steric effects preventing a strong interaction with the five siloxide ligands in 5Cs-Pr^Ph^ and [5-Pr^Ph^]^−^ as evidenced by the increased Pr–O distances in the latter (around 2.15 Å in average) compared to [Pr^IV^(OSiPh_3_)_4_(CH_3_CN)_2_] (2.08 Å In average). On the other hand, in the 5K-Pr^Ph^, two Pr–O_siloxide_ WBI are reduced to 0.57–0.60 while the others remain in the 0.74–0.75 range. This is due to the presence of strong K–O_siloxide_ interactions, that reduce the electron density at the oxygen of the siloxide ligand resulting in a less efficient overlap with the Pr(iv) centre.

## Experimental

### Synthesis of [Pr^IV^(OSi(O^t^Bu)_3_)_4_] (2-Pr^OtBu^)

In an argon filled glovebox, a cold (−40 °C) purple solution of thiaBF_4_ (0.030 g, 0.097 mmol, 1.5 equiv.) in CH_3_CN (0.8 mL) was added dropwise, under stirring to a cold (−40 °C) colorless solution of [KPr(OSi(OtBu)_3_)_4_] (1-Pr^OtBu^), prepared as previously reported,^5^ (0.080 g, 0.065 mmol, 1 equiv.) in toluene (0.2 mL). The resulting purple suspension was stirred at −40 °C in freezer for 15 min. After 15 min the color of the suspension changed to red purple. The suspension was filtered and the red solid collected over a porosity 4 filter-frit, at −40 °C and then washed with MeCN (2 × 3 mL) to remove the byproducts. The red solid was then dissolved in cold (−40 °C) *n*-hexane (0.8 mL), resulting in a dark red solution which was filtered over a 0.22 μm porosity filter frit. The cold red *n*-hexane solution was concentrated under reduced pressure to yield 49% (0.038 g, 0.032 mmol) a red solid. X-ray quality single red crystals of 2-Pr^OtBu^ were obtained overnight from concentrated *n*-hexane solution of 2-Pr^OtBu^ at −40 °C. ^1^H NMR (400 MHz, Tol-*d*_*8*_, 233 K) *δ* 1.85 (br, OC(CH_3_)_3_), ppm. ^1^H NMR (400 MHz, CD_2_Cl_2_, 233 K) *δ* 1.53 (br, OC(CH_3_)_3_), ppm. Anal. Cal. For C_48_H_108_O_16_Si_4_Pr: C, 48.26; H, 9.11; N, 0.00. Found: C, 47.64; H, 9.13; N, 0.00.

### Synthesis of [K_2_Pr^iii^(OSi(Ph)_3_)_5_] (4K-Pr^Ph^)

A solution of KOSiPh_3_ (56 mg, 0.178 mmol, 2.1 equiv.) in toluene (1 mL) was added at room temperature to a pale green solution of [Pr(OSiPh_3_)_3_(THF)_3_], prepared as previously reported^5,56^ (100 mg, 0.85 mmol, 1 equiv.) in toluene (1 mL). The resultant pale green solution was stirred for 1 hour at room temperature. The volatiles were removed under vacuum, the residue was triturated with *n*-hexane (3 × 2 mL) and dried for 30 min affording white solid. Toluene (1.0 mL) was added, and the colorless solution storage at −40 °C affording white crystalline powder of [K_2_Pr(OSi(Ph)_3_)_5_], 4K-Pr^Ph^, in 81% yield (110 mg, 0.069 mmol). Anal. Calcd for [K_2_Pr(OSi(Ph)_3_)_5_]. (Toluene) (1688.10 g mol^−1^): C_97_H_83_K_2_O_5_PrSi_5_: C, 69.01; H, 4.96; N, 0.00. Found: C, 68.91; H, 4.94; N, 0.00. The fractional toluene content is residual co-crystallized solvent left from partial drying also observed in ^1^H NMR spectrum. ^1^H NMR (CD_2_Cl_2_, 400 MHz, 298 K): *δ* = 6.73 (s, ^15^H, Ph), 5.66 (s, 30H, Ph), 4.47 (br, s, 30H, Ph) ppm. Single crystals suitable for X-ray diffraction analysis were obtained from a toluene solution of 4K-Pr^Ph^ layered with *n*-hexane at rt.

### Synthesis of [KPr^iv^(OSi(Ph)_3_)_5_] (5K-Pr^Ph^)

To a colorless solution of 4K-Pr^Ph^ (100 mg, 0.063 mmol, 1 equiv.) in toluene (1 mL), dark purple solid of thiaBF_4_ (21 mg, 0.069 mmol, 1.1 equiv.) was added at −40 °C and reaction mixture was stirred for 30 min at −40 °C. During the stirring color of reaction mixture was changed to deep red. The red color reaction mixture was filtered off over porosity 4 filter-frit. Deep red filtrate was evaporated to dryness under reduced pressure to get dark red solid. The dark red solid was redissolved in toluene (1 mL) and layered with *n*-hexane at −40 °C to get dark red crystals of 5K-Pr^Ph^ in 51% yield. (50 mg, 0.032 mmol). Anal. Calcd for [KPr(OSi(Ph)_3_)_5_]. (Toluene)_2_ (1741.22 g mol^−1^): C_104_H_91_O_5_Si_5_PrK: C, 71.73; H, 5.27; N, 0.00. Found: C, 71.50; H, 5.29; N, 0.00. The toluene content is residual co-crystallized solvent left from partial drying and also observed in ^1^H NMR spectrum. ^1^H NMR (Tol-d_*8*_, 400 MHz, 233 K): *δ* = 6.95–6.91 (t, 30H, Ph), 6.74 (s, 45H, Ph) ppm. ^1^H NMR (Tol-d_*8*_, 400 MHz, 298 K): *δ* = 6.95–6.91 (t, 30H, Ph), 6.73 (s, 45H, Ph) ppm. ^1^H NMR (CD_2_Cl_2_, 400 MHz, 233 K): *δ* = 7.04 (br, s, ^15^H, Ph), 6.68 (br, s, 60H, Ph) ppm. ^1^H NMR (CD_2_Cl_2_, 400 MHz, 298 K): *δ* = 7.07 (br, s, ^15^H, Ph), 6.72 (br, s, 60H, Ph) ppm.

### Synthesis of [KDB18C6][Pr^iv^(OSiPh_3_)_5_] (5[KDB18C6]-Pr^Ph^)

To a dark red solution of 5K-Pr^Ph^ (50 mg, 0.032 mmol, 1.0 equiv.) in DCM (0.5 mL), white solid of dibenzo-18-crown-6 (DB18C6) (11.5 mg, 0.032 mmol, 1.0 equiv.) was added and reaction mixture was stirred for 5 min at rt. During the stirring color of reaction mixture was changed to orange from dark red. The orange-colored solution was layered with *n*-hexane and kept at −40 °C to get orange crystals of 5[KDB18C6]-Pr^Ph^ in 72% yield. (44 mg, 0.022 mmol). Anal. Calcd for 5[KDB18C6]-Pr^Ph^. (CH_2_Cl_2_) (2002.33 g mol^−1^): C_111_H_101_O_11_Si_5_PrCl_2_: C, 66.58; H, 5.08; N, 0.00. Found: C, 66.15; H, 5.08; N, 0.01. The DCM content is residual co-crystallized solvent left from partial drying.^1^H NMR (CD_2_Cl_2_, 400 MHz, 233 K): *δ* = 7.32 (s, 15H, Ph), 6.94 (t, 30H, Ph), 6.90 (m, 8H, DB18C6), 6.57 (d, 30H, Ph), 4.14 (s, 8H, DB18C6), 3.91 (s, 8H, DB18C6) ppm. ^1^H NMR (CD_2_Cl_2_, 400 MHz, 298 K): *δ* = 7.26 (s, ^15^H, Ph), 7.04–7.01 (m, 8H, DB18C6), 6.94 (t, 30H, Ph), 6.63 (s, 30H, Ph), 4.21 (t, 8H, DB18C6), 3.98 (t, 8H, DB18C6) ppm.

## Conclusions

In summary, we have identified reaction conditions (solvent, oxidizing agent, and cation effect) that have allowed the synthesis and full characterization of four new complexes of Pr(iv), while presenting an unprecedented penta-coordination environment. The use of the strong oxidizing agent, thiaBF_4_, prevented previously observed decomposition pathways of the complex, [Pr^iv^(OSi(OtBu)_3_)_4_] (2-Pr^OtBu^), and allowed its isolation in the solid state, which was found to be isostructural with the previously reported Tb(iv) analogue. Despite the very similar oxidation potential measured for 2-Pr^OtBu^, and for its previously reported Tb(iv) analogue, the Pr(iv) complex is significantly more labile and more prone to undergo fast decomposition pathways rendering the choice of the oxidizing agents crucial for its isolation. The isolated complex is relative stable in cold toluene and DCM but decomposes rapidly at room temperature to afford the Pr(iii) dimeric complex, [Pr(OSi(OtBu)_3_)_3_]_2_ (3-Pr^OtBu^). The oxidation of the Pr(iii) pentakis-triphenylsiloxide complexes, 4M-Pr^Ph^ (K, Cs) with thiaBF_4_ in toluene allowed the isolation of the first example of anionic penta-coordinated Pr(iv) complexes. The complexes were both isolated with inner-sphere bound K and Cs cations as complexes, 5M-Pr^Ph^ (K, Cs), and as an ion pair, 5[KDB18C6]-Pr^Ph^, for complex 5K-Pr^Ph^. The solution stability of these pentakis complexes is slightly increased compared to the previously reported tetrakis complex, [Pr^IV^(OSiPh_3_)_4_(CH_3_CN)_2_] although we found that the presence of an additional anionic ligand does not result in a complex that is easier to oxidize. Computational and structural analysis suggests that steric hindrance prevents the formation of strong π-bonding interactions between the –OSiPh_3_ ligands and the Pr(iv) center resulting in an overall similar bonding mode in both tetrakis-and pentakis-complexes. All Pr(iv) complexes show similar magnetic properties independent of the geometry and number of –OSiPh_3_ ligands, which are also similar to the Ce(iii) analogues in agreement with the computed electronic structures. The possibility of accessing charged Pr(iv) complexes provide a tool to manipulate the redox potential and therefore access to more stable complexes with the same ligand, but care needs to be taken in preventing unfavorable steric interactions. Future studies will be directed to explore this route to access a broad range of Pr(iv) complexes.

## Author contributions

P. P. designed and carried out all the experiments and analysed the data; M. K. performed preliminary experiments. M. M. designed and supervised the project and analysed the data; T. R. and L. M. carried out the computational study; R. S. measured and analyzed the X-ray data, I. Z. measured and analysed the magnetic data; A. S. measured and analysed the EPR data; P. P, L. M. and M. M. wrote the manuscript with contributions of all authors, and all authors have given approval for the final version of the manuscript.

## Conflicts of interest

There are no conflicts to declare.

## Supplementary Material

SC-OLF-D5SC05500H-s001

SC-OLF-D5SC05500H-s002

## Data Availability

The data that support the findings of this study are openly available in the Zenodo repository, https://doi.org/10.5281/zenodo.17280527. CCDC 2470050–2470056 and 2471366 contain the supplementary crystallographic data for this paper.^57*a*–*h*^ Supplementary information: synthetic details, analytical data including depictions of all spectra and coordinate data of all computationally optimised species, are documented in the supplementary information (SI). See DOI: https://doi.org/10.1039/d5sc05500h.
