# Supplementary material for: Accessing homoleptic neutral and anionic five-coordinate Pr(iv) siloxide complexes
Source: Chem Sci. 2025 Oct 3;16(44):21056–67. doi: 10.1039/d5sc05500h (PMC12517353; doi:10.1039/d5sc05500h)
Supplement: SC-016-D5SC05500H-s001 [file SC-016-D5SC05500H-s001.pdf]

Supplementary Information (SI) for Chemical Science.
This journal is © The Royal Society of Chemistry 2025

Supporting Information

Accessing Homoleptic Neutral and Anionic Five-coordinate Pr(IV) Siloxide Complexes

Pragati Pandey,[a] Megan Keneer,[a] Thayalan Rajeshkumar,[e] Rosario Scopelliti,[b] Andrzej Sienkiewicz,[c,d] Ivica Zivkovic,[c] Laurant Maron,*[e] and Marinella Mazzanti*[a]

*laurent.maron@irsamc.ups-tlse.fr; *marinella.mazzanti@epfl.ch

[a]Group of Coordination Chemistry, Institut des Sciences et Ingénierie Chimiques, École Polytechnique Fédérale de Lausanne (EPFL), CH-1015 Lausanne, Switzerland

[b]X-ray Diffraction and Surface Analytics Platform, Institut des Sciences et Ingénierie Chimiques, École Polytechnique Fédérale de Lausanne (EPFL), CH-1015 Lausanne, Switzerland

[c]Laboratory for Quantum Magnetism, Institute of Physics, École Polytechnique Fédérale de Lausanne (EPFL), CH-1015 Lausanne, Switzerland

[d]ADSresonances Sarl, CH-1920 Martigny, Switzerland

[e]Laboratoire de Physique et Chimie desNano-objets, Institut National des Sciences Appliquées, 31077 Toulouse, France

Table of Content

	Page No.
**1. Experimental Details**	**S3-S4**
**2. Synthesis and Characterization**	**S5-S9**
**3. NMR Spectroscopy Data**	**S9-S47**
**4. X-ray Crystal Structure Determination Details**	**S48-S58**
**5. UV-Vis Spectra**	**S59-S62**
**6. Electrochemistry**	**S63-S66**
**7. EPR**	**S67-S70**
**8. Magnetism**	**S71-S73**
**9. Computational Details**	**S74-S162**
**10. References**	**S163**

Experimental Section:

General Methods. Unless otherwise noted, all manipulations were carried out at ambient temperature under an inert argon or nitrogen atmosphere using Schlenk techniques and an MBraun glovebox equipped with a purifier unit. The water and oxygen levels were always kept at less than 0.1 ppm. Glassware was dried overnight at 140 °C before use.

NMR experiments were carried out using NMR tubes adapted with J-Young valves. NMR spectra were recorded on Bruker 400, 500, or 600 MHz spectrometers and referenced to residual solvent resonances of THF (d8-THF), toluene (d8-toluene), or Acetonitrile (d3-MeCN) in Pyrex NMR tubes adapted with J-Young valves. Chemical shifts were recorded in units of parts per million and referenced against residual proteo solvent peaks.

Elemental analyses were performed under an inert atmosphere of nitrogen with a ThermoScientific Flash 2000 Organic Elemental Analyzer.

Cyclic voltammetry data were carried out at room temperature in an argon-filled glovebox described above. Data were collected using a Biologic SP-300 potentiostate connected to a personal computer. All samples were measured with 0.1 M $[NBu_4][B(C_6F_5)_4]$ supporting electrolyte in THF DCM or CH_3CN solution. The experiments were carried out with a platinum disk (d = 5 mm) working electrode, a platinum wire counter electrode, and an Ag/AgCl reference electrode. Potential calibration was performed at the end of each data collection cycle using the ferrocene/ferrocenium couple as an internal standard.

Materials. Unless otherwise noted, reagents were purchased from commercial suppliers and used without further purification. Anhydrous solvents were purchased from Aldrich and further distilled from K/benzophenone (THF, and toluene), sodium sand/benzophenone (n-hexane) or CaH_2 (MeCN). Deuterated solvents for NMR spectroscopy were purchased from Cortecnet, freeze-degassed and distilled over K/benzophenone (THF-d8, toluene-d8) or CaH_2 (MeCN-d_3). Potassium bis(trimethylsilyl)amide (KHMDS), cerium chloride, $[N(C_6H_4Br)_3][SbCl_6]$ dibenzo-18-crown-6 (DB18C6) was purchased from Sigma Aldrich. $HOSi(O^tBu)_3$ was purchased from Sigma Aldrich and was sublimed prior to use. $KOSiPh_3$ and $KOSi(OtBu)_3$ were prepared by stirring $HOSiPh_3$ and $HOSi(OtBu)_3$ respectively with a slight excess of KH in THF, followed by filtration and removal of the solvent under vacuum.

$[KCe(O(SiO^tBu)_3)_4]$,[1] $Cs(HMDS)$[2], $[KPr(O(SiO^tBu)_3)_4]$ (**1-Pr^{OtBu}**)[3] $[Pr^{IV}(OSiPh_3)_4(CH_3CN)_2]$[3] and $[Tb^{IV}(O(SiO^tBu)_3)_4]$[4] were prepared according to the previously published method. $CsOSiPh_3$ was prepared by stirring $HOSiPh_3$ with 1 eq of Cs(HMDS) in THF followed by extraction with toluene and removal of solvent under vaccum. $[NBu_4][B(C_6F_5)_4]$ was prepared according to the published procedure.[5] $ThiaBF_4$ was prepared according to the published procedure.[6]

EPR analysis were performed on a Bruker Elexsys E500 spectrometer working at 9.4 GHz frequency with an Oxford ESR900 cryostat for 4-300 K operation.

Magnetization were performed using a QuantumDesign MPMS3 superconducting quantum interference device (SQUID) magnetometer in a temperature range 2-300 K. The powder sample was constrained in glasswool and enclosed in an evacuated quartz capsule and placed inside a plastic straw. The measurement was performed with applied magnetic field of 1 T in the zero-field cooled (ZFC) regime and performed on two independent samples for reproducibility. Diamagnetic corrections were applied using Pascal's constants.[7] The magnetic moment was calculated using the formula: μeff = $\sqrt{8XT}$

UV/Vis data were recorded using 1.0 mm cuvettes equipped with a J-Young valve and a Perkin Elmer 950 spectrometer.

2. Synthesis and Characterization

Synthesis of [Pr(OSi(O^{t}Bu)$_3$)$_4$] (2-Pr^{OtBu}). In an argon filled glovebox, a cold (-40° C) purple solution of thiaBF$_4$ (0.030 g, 0.097 mmol, 1.5 equiv) in CH_3CN (0.8 mL) was added dropwise, under stirring to a cold (–40 °C) colorless solution of [KPr(OSi(OtBu)$_3$)$_4$] (**1-Pr^{OtBu}**) (0.080 g, 0.065 mmol, 1 equiv) in toluene (0.2 mL). The resulting purple suspension was stirred at −40 °C in freezer for 15 min. After 15 min the color of the suspension changed to red purple. The suspension was filtered and the red solid collected over a porosity 4 filter-frit, at –40 °C and then washed with MeCN (2 × 3 mL) to remove the byproducts. The red solid was then dissolved in cold (–40 °C) *n*-hexane (0.8 mL), resulting in a dark red solution which was filtered over a 0.22 µm porosity filter frit. The cold red *n*-hexane solution was concentrated under reduced pressure to yield 49% (0.038 g, 0.032 mmol) a red solid. X-ray quality single red crystals of **2-Pr^{OtBu}** were obtained overnight from concentrated *n*-hexane solution of **2-Pr^{OtBu}** at –40 °C. ^{1}H NMR (400 MHz, Tol-*d*$_8$, 233K) δ 1.85 (br, OC(CH$_3$)$_3$), ppm. ^{1}H NMR (400 MHz, CD_2Cl_2, 233K) δ 1.53 (br, OC(CH$_3$)$_3$), ppm. Anal. Cal. For $C_{48}H_{108}O_{16}Si_4Pr$: C, 48.26; H, 9.11; N, 0.00. Found: C, 47.64; H, 9.13; N, 0.00.

Synthesis of [Pr(OSi(O^{t}Bu)$_3$)$_3$]$_2$ (3-Pr^{OtBu}). A solution of HOSi(OtBu)$_3$ (127.5 mg, 0.482 mmol, 3 eq.) in toluene (2 mL) was added at room temperature to a clear solution of Pr((N(SiMe$_3$)$_2$)$_3$ (100 mg, 0.161 mmol, 1 eq.) in toluene (1 mL). The resulting colorless solution was stirred for 3 hours at room temperature. The solvent was removed under vacuum and the off-white residue was triturated with n-hexane (2 mL) and dried under reduced pressure to give off white solid. Toluene (1 mL) was added, and the colorless solution was storage at –40 °C affording white crystalline powder of of [Pr(OSi(O^{t}Bu)$_3$)$_3$]$_2$, **3-Pr^{OtBu}**, in 83% yield (124 mg, 0.133 mmol). Anal. Calcd for [Pr(OSi(O^{t}Bu)$_3$)$_3$]$_2$ (1862.37 g mol^{-1}): $C_{72}H_{162}O_{24}Pr_2Si_6$: C, 46.43; H, 8.78; N, 0.00. Found: C, 46.43; H, 8.88; N, 0.00. ^{1}H NMR (Tol-d$_8$, 400 MHz, 298 K): δ 8.76 (s, 54H, OC(CH$_3$)$_3$), 1.10 (s, 9H, OC(CH$_3$)$_3$), −14.52 (S, 18H, OC(CH$_3$)$_3$), ppm.

Decomposition of 2-Pr^{OtBu} to 3-Pr^{OtBu} A scintillation vial was charged with a red solution of **2-Pr^{OtBu}** (0.020g, 0.017 mmol, 1 equiv) in 0.3 mL of toluene. The vial was kept at room temperature overnight resulting in color change from red to colorless. The solvent was evaporated under reduced pressure to yield **3-Pr^{OtBu}** as off white solid (the ^{1}H NMR spectrum shows only the signals assigned to **3-Pr^{OtBu}**). X-ray quality

crystals were obtained from saturated solution of **3-Pr^{OtBu}** in toluene at –40° C. Yield 0.011 g, 0.006 mmol, 70%. ^{1}H NMR (400 MHz, Tol-d_8, 298K) δ 8.76 (s, 54H, $OC(CH_3)_3$), 1.10 (s, 9H, $OC(CH_3)_3$), −14.52 (S, 18H, $OC(CH_3)_3$), ppm.

Synthesis of [$K_2Pr(OSi(Ph)_3)_5$] (4K-Pr^{Ph}). A solution of $KOSiPh_3$ (56 mg, 0.178 mmol, 2.1 equiv.) in toluene (1 mL) was added at room temperature to a pale green solution of [$Pr(OSiPh_3)_3(THF)_3$] (100 mg, 0.85 mmol, 1 equiv.) in toluene (1 mL). The resultant pale green solution was stirred for 1 hour at room temperature. The volatiles were removed under vacuum, the residue was triturated with n-hexane (3 x 2 mL) and dried for 30 min affording white solid. Toluene (1.0 mL) was added, and the colorless solution storage at –40 °C affording white crystalline powder of [$K_2Pr(OSi(Ph)_3)_5$], **4K-Pr^{Ph}**, in 81% yield (110 mg, 0.069 mmol). Anal. Calcd for [$K_2Pr(OSi(Ph)_3)_5$].(Toluene) (1,688.10g mol^{-1}): $C_{97}H_{83}K_2O_5PrSi_5$: C, 69.01; H, 4.96; N, 0.00. Found: C, 68.91; H, 4.94; N, 0.00. The fractional toluene content is residual co-crystallized solvent left from partial drying also observed in ^{1}H NMR spectrum. ^{1}H NMR (CD_2Cl_2, 400 MHz, 298 K): δ = 6.73 (s, 15H, Ph), 5.66 (s, 30H, Ph), 4.47 (br, s, 30H, Ph) ppm. Single crystals suitable for X-ray diffraction analysis were obtained from a toluene solution of **4K-Pr^{Ph}** layered with n-hexane at rt.

Synthesis of [$Cs_2Pr(OSi(Ph)_3)_5$] (4Cs-Pr^{Ph}). A solution of $CsOSiPh_3$ (72.5 mg, 0.178 mmol, 2.1 equiv.) in toluene (1 mL) was added at room temperature to a pale green solution of [$Pr(OSiPh_3)_3(THF)_3$] (100 mg, 0.85 mmol, 1 equiv.) in toluene (1 mL). The resultant pale green solution was stirred for 3 hours at room temperature. During reaction white ppt crashed out. All the volatiles were removed under vacuum, the residue was triturated with n-hexane (3 x 2 mL) and dried for 2 hours affording off white solid. Toluene (2 mL) was added, and the colorless solution storage at –40 °C affording off white crystalline powder of [$Cs_2Pr(OSi(Ph)_3)_5$], 4Cs-Pr^{Ph}. in 78% yield (112 mg, 0.063 mmol). Anal. Calcd for [$Cs_2Pr(OSi(Ph)_3)_5$].(Toluene) (1783.70 g mol^{-1}): $C_{97}H_{83}Cs_2O_5PrSi_5$: C, 62.10; H, 4.46; N, 0.00. Found: C, 61.94; H, 4.48; N, 0.00. The toluene content is residual co-crystallized solvent left from partial drying also observed in ^{1}H NMR spectrum.^{1}H NMR (CD_2Cl_2, 400 MHz, 298 K): δ = 6.85 (br, s, 15H, Ph), 6.05 (br, s, 30H, Ph), 5.59 (br, 30H, Ph) ppm. Single crystals suitable for X-ray diffraction analysis were obtained from a toluene solution of **4Cs-Pr^{Ph}** layered with n-hexane at −40 °C.

Synthesis of [KPr(OSi(Ph)$_3$)$_5$] (5K-Pr^{Ph}). To a colorless solution of **4K-Pr^{Ph}** (100 mg, 0.063 mmol, 1 equiv.) in toluene (1mL), dark purple solid of thiaBF$_4$ (21 mg, 0.069 mmol, 1.1 equiv.) was added at –40 °C and reaction mixture was stirred for 30 min at –40 °C. During the stirring color of reaction mixture was changed to deep red. The red color reaction mixture was filtered off over porosity 4 filter-frit. Deep red filtrate was evaporated to dryness under reduced pressure to get dark red solid. The dark red solid was redissolved in toluene (1 mL) and layered with n-hexane at –40 °C to get dark red crystals of **5K-Pr^{Ph}** in 51% yield. (50 mg, 0.032 mmol). Anal. Calcd for [KPr(OSi(Ph)$_3$)$_5$].(Toluene)$_2$ (1741.22 g mol^{-1}): $C_{104}H_{91}O_5Si_5PrK$: C, 71.73; H, 5.27; N, 0.00. Found: C, 71.50; H, 5.29; N, 0.00. The toluene content is residual co-crystallized solvent left from partial drying also observed in ^{1}H NMR spectrum. ^{1}H NMR (Tol-d$_8$, 400 MHz, 233 K): δ = 6.95-6.91 (t, 30H, Ph), 6.74 (s, 45H, Ph) ppm. ^{1}H NMR (Tol-d$_8$, 400 MHz, 298 K): δ = 6.95-6.91 (t, 30H, Ph), 6.73 (s, 45H, Ph) ppm. ^{1}H NMR (CD_2Cl_2, 400 MHz, 233 K): δ = 7.04 (br, s, 15H, Ph), 6.68 (br, s, 60H, Ph) ppm. ^{1}H NMR (CD_2Cl_2, 400 MHz, 298 K): δ = 7.07 (br, s, 15H, Ph), 6.72 (br, s, 60H, Ph) ppm.

Synthesis of [KDB18C6][Pr^{IV}(OSiPh$_3$)$_5$] (5[KDB18C6]-Pr^{Ph}). To a dark red solution of **5K-Pr^{Ph}** (50 mg, 0.032 mmol, 1.0 equiv.) in DCM (0.5 mL), white solid of dibenzo-18-crown-6 (DB18C6) (11.5 mg, 0.032 mmol, 1.0 equiv.) was added and reaction mixture was stirred for 5 min at r.t. During the stirring color of reaction mixture was changed to orange from dark red. The orange-colored solution was layered with n-hexane and kept at –40 °C to get orange crystals of **5[KDB18C6]-Pr^{Ph}** in 72% yield. (44 mg, 0.022 mmol). Anal. Calcd for 5[KDB18C6]-Pr^{Ph}.(CH_2Cl_2) (2002.33 g mol^{-1}): $C_{111}H_{101}O_{11}Si_5PrCl_2$: C, 66.58; H, 5.08; N, 0.00. Found: C, 66.15; H, 5.08; N, 0.01. The DCM content is residual co-crystallized solvent left from partial drying.^{1}H NMR (CD_2Cl_2, 400 MHz, 233 K): δ = 7.32 (s, 15H, Ph), 6.94 (t, 30H, Ph), 6.90 (m, 8H, DB18C6), 6.57 (d, 30H, Ph), 4.14 (s, 8H, DB18C6), 3.91 (s, 8H, DB18C6) ppm. ^{1}H NMR (CD_2Cl_2, 400 MHz, 298 K): δ = 7.26 (s, 15H, Ph), 7.04-7.01 (m, 8H, DB18C6), 6.94 (t, 30H, Ph), 6.63 (s, 30H, Ph), 4.21 (t, 8H, DB18C6), 3.98 (t, 8H, DB18C6) ppm.

Synthesis of [CsPr(OSi(Ph)$_3$)$_5$] (5Cs-Pr^{Ph}). To a white suspension of **4Cs-Pr^{Ph}** (90 mg, 0.063 mmol, 1 equiv.) in toluene (2 mL), dark purple solid of thiaBF$_4$ (21 mg, 0.069 mmol, 1.1 equiv.) was added at –40 °C and reaction mixture was stirred for 30 min at –40 °C. During the stirring color of reaction mixture was changed to deep red. The red

color reaction mixture was filtered off over porosity 4 filter-frit. Red filtrate was evaporated to dryness under reduced pressure to get dark red solid. Dark red solid was redissolved in toluene (1 mL) and allowed to stand for week at –40 °C resulting in mixture of red and white crystals identified as of **5Cs-Pr^{Ph}** and thianthrene respectively. Better quality crystals were obtained from layering saturated solution of **5Cs-Pr^{Ph}** in DCM (0.5 mL) with hexane (0.5 mL) at –40 °C for two days. Dark red crystals were filtered over frit and washed with n-hexane (3 x 1 mL) and dried under reduced pressure to give 54% yield (45 mg, 0.027 mmol). Anal. Calcd for [CsPr(OSi(Ph)$_3$)$_5$]].(Toluene) (1742.81 g mol^{-1}): $C_{97}H_{83}O_5Si_5PrCs$: C, 66.84; H, 4.80; N, 0.00. Found: C, 66.52; H, 5,02; N, 0.00. The toluene content is residual co-crystallized solvent left from partial drying. (However, the toluene signals are not observed in ^{1}H NMR spectra in CD_2Cl_2 as it was recorded with different batch of compound which was dried overnight under high vacuum). ^{1}H NMR (CD_2Cl_2, 400 MHz, 233 K): δ = 7.00 (br, s, 15H, Ph), 6.64 (br, s, 60H, Ph) ppm. ^{1}H NMR (CD_2Cl_2, 400 MHz, 298 K): δ = 7.04 (t, 15H, Ph), 6.75-6.71 (br, m, 60H, Ph) ppm. ^{1}H NMR (Tol-d_8, 400 MHz, 233 K): δ = 6.95 (br, s, 15H, Ph), 6.67 (br, s, 60H, Ph) ppm. ^{1}H NMR (Tol-d_8, 400 MHz, 298 K): δ = 6.94 (t 15H, Ph), 6.82 (br, s, 30H, Ph), 6.71 (br, s, 30H, Ph) ppm.

Synthesis of [K$_2$Ce(OSi(Ph)$_3$)$_5$] (4K-Ce^{Ph}). Following the procedure used for the synthesis of **4K-Pr^{Ph}**, the addition of $KOSiPh_3$ (83.8 mg, 0.27 mmol, 2.1 equiv.) in toluene (1.0 mL) to a colorless solution of [Ce(OSiPh$_3$)$_3$(THF)$_3$] (150 mg, 0.127 mmol, 1 equiv.) in toluene (1.0 mL) afforded analytically pure [K$_2$Ce(OSi(Ph)$_3$)$_5$] ($K_2CeL_5^{Ph}$) as white crystalline solid in 78 % yield (158 mg, 0.099 mmol). Single crystals suitable for X-ray diffraction analysis were obtained from a toluene solution of $K_2CeL_5^{Ph}$ layered with n-hexane at rt. Anal. Calcd for [K$_2$Ce(OSi(Ph)$_3$)$_5$] (1595.18 g mol^{-1}): $C_{90}H_{75}K_2O_5CeSi_5$: C, 67.76; H, 4.74. Found: C, 67.65; H, 4.73. ^{1}H NMR (Tol-d_8, 400 MHz, 298 K): δ = 6.91-6.87 (t, 15H, Ph), 6.50-6.47 (t, 30H, Ph), 6.07 (s, 30H, Ph) ppm.

Synthesis of [K(2.2.2cryptand)][Pr^{III}(k_1-OSi(OtBu)$_3$)$_4$]. To a colorless solution of **1-Pr^{OtBu}** (10 mg, 0.0081 mmol, 1.0 equiv.) in THF (0.5 mL), white solid of [2.2.2cryptand] (3.1 mg, 0.0081 mmol, 1.0 equiv.) was added and reaction mixture was stirred for 5 min at room temperature. After that the saturated clear solution was layered with n-hexane and kept at room temperature to get colorless crystals of **[K(2.2.2cryptand)][Pr^{III}(k_1-OSi(OtBu)$_3$)$_4$]** in 81% yield. (10.5 mg, 0.0065 mmol).

However, even after several trials the quality of the diffracted crystals were not good to get publishable data but still connectivity of atoms was established to get reliable structure. Anal. Calcd for [K(2.2.2cryptand)][Pr^{III}(k_1-OSi(OtBu)$_3$)$_4$] (1610.20 g mol^{-1}): $C_{66}H_{144}O_{22}N_2Si_4Pr$: C, 49.23; H, 9.01; N, 1.74. Found: C, 48.98; H, 8.78; N, 1.69. ^{1}H NMR (THF-d_8, 400 MHz, 298 K): δ = 3.59 (s, 12H, Crypt), 3.55 (t, 12H, Crypt), 2.57 (t, 12H, Crypt), 0.85 (s, 108H, OSi(OtBu)$_3$) ppm.

3. NMR Spectroscopy Data

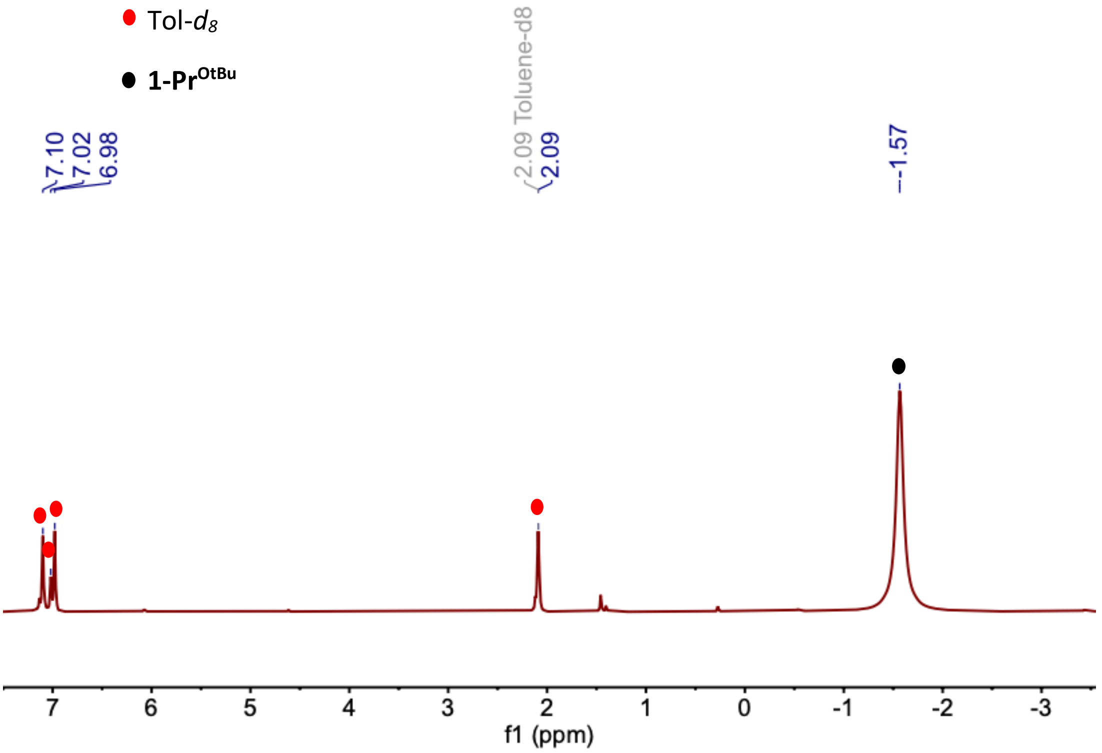

Figure S1. ^{1}H NMR (400 MHz, Tol-d_8, 298K) spectrum of **1-Pr^{OtBu}**

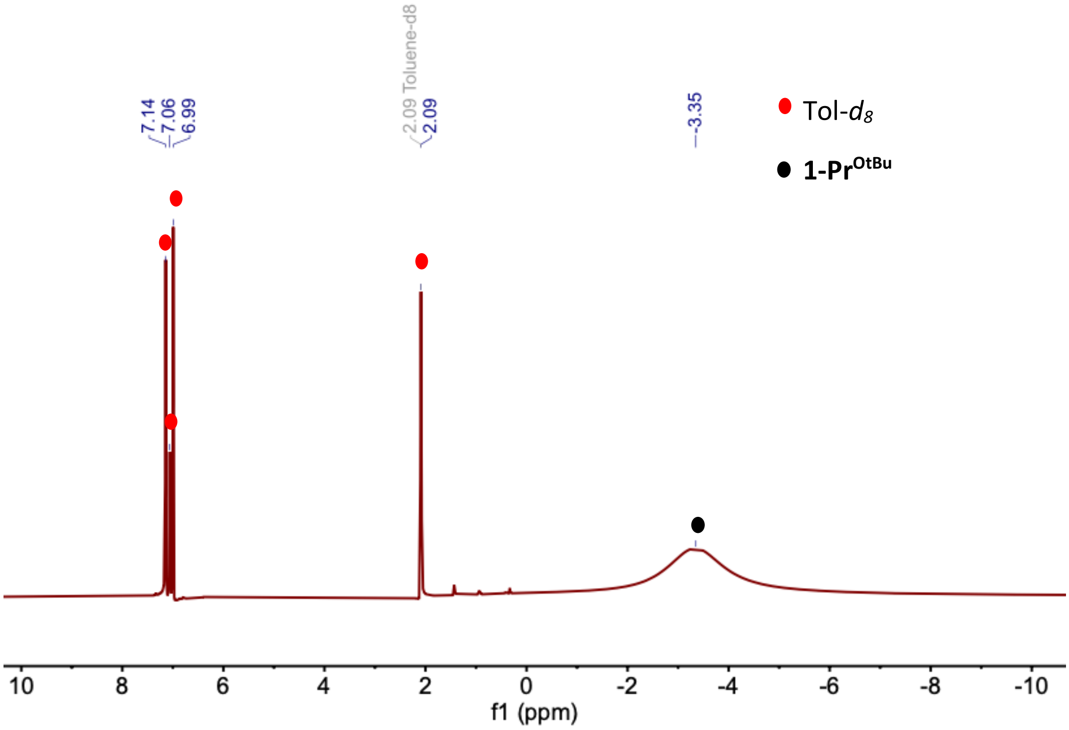

Figure S2. ^{1}H NMR (400 MHz, Tol-d_8, 233K) spectrum of **1-Pr^{OtBu}**

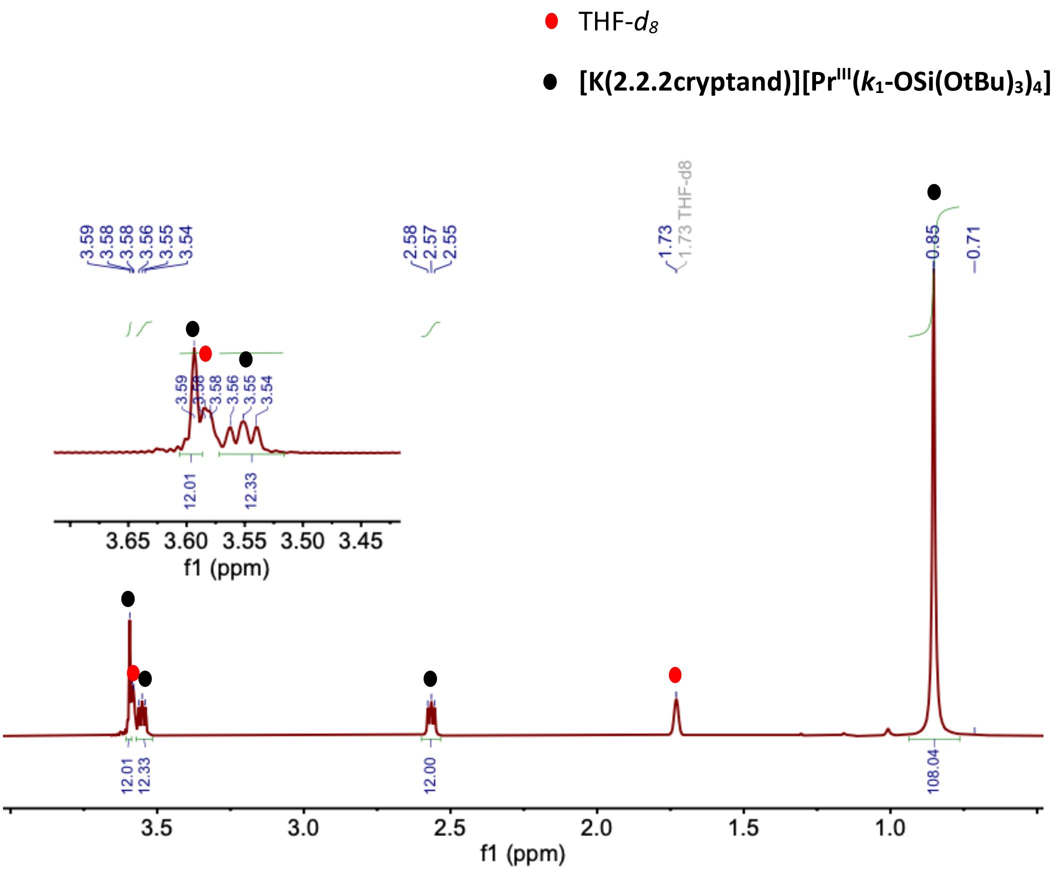

Figure S3. ^{1}H NMR (400 MHz, THF-d_8, 298K) spectrum of **[K(2.2.2cryptand)][Pr^{III}(k_1-OSi(OtBu)$_3$)$_4$].**

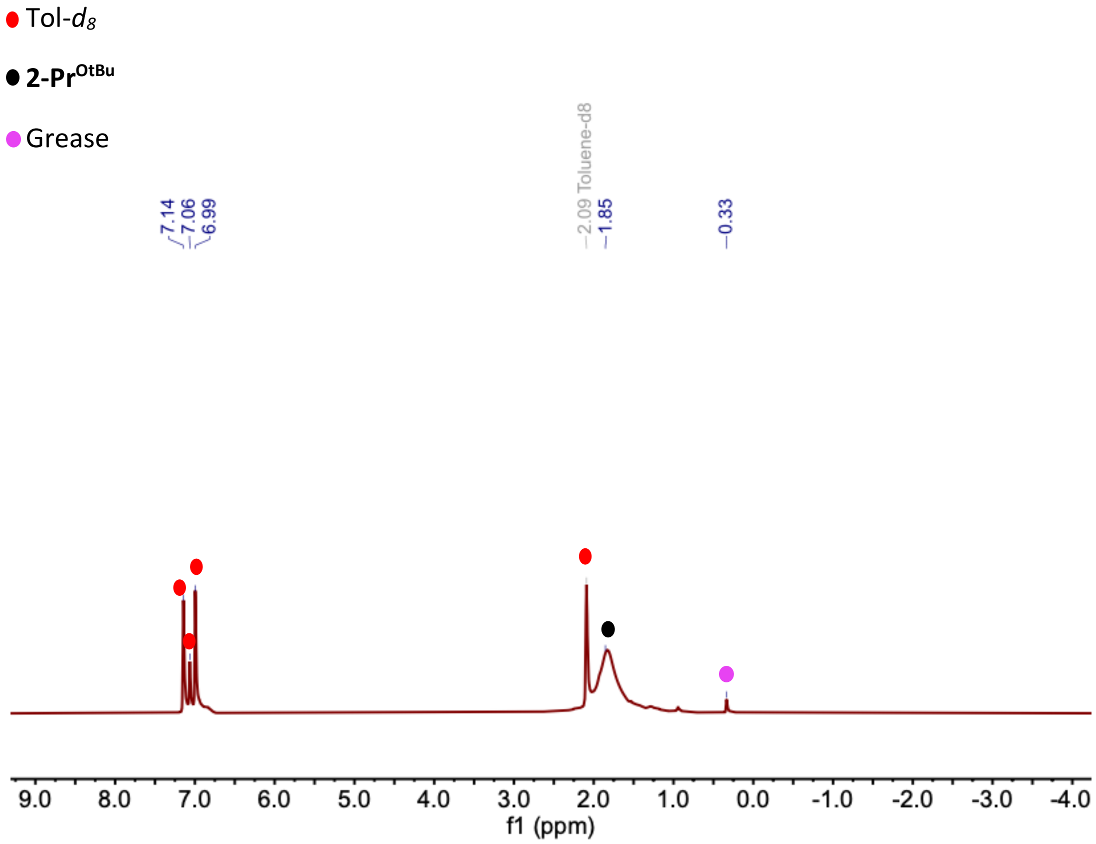

Figure S4. ^{1}H NMR (400 MHz, Tol-d_8, 233K) spectrum of **2-Pr^{OtBu}**.

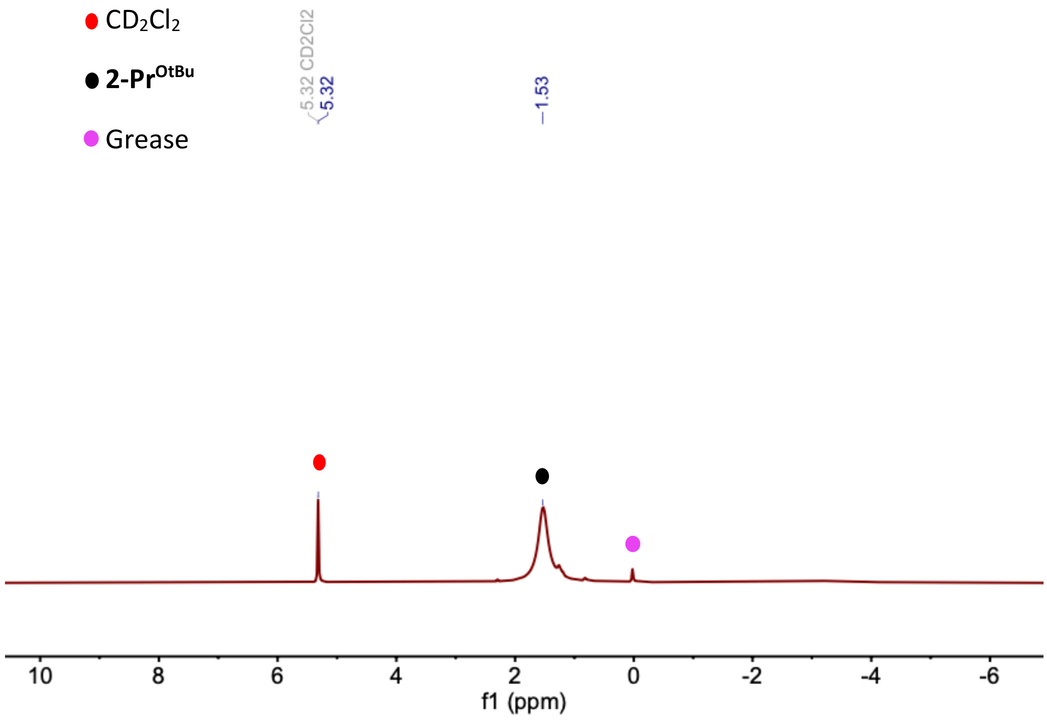

Figure S5. ^{1}H NMR (400 MHz, CD_2Cl_2, 233K) spectrum of **2-Pr^{OtBu}**.

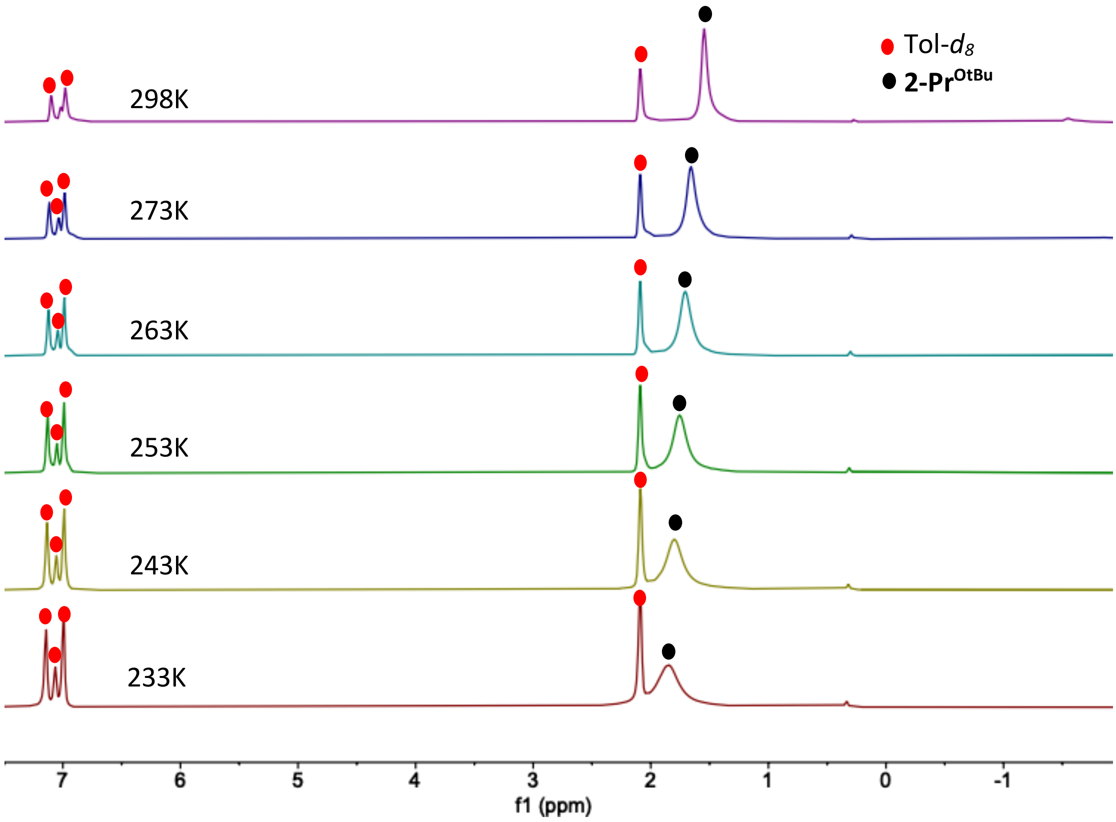

Figure S6. Variable temperature ^{1}H NMR (400 MHz, Tol-d_8) spectra of **2-Pr^{OtBu}**.

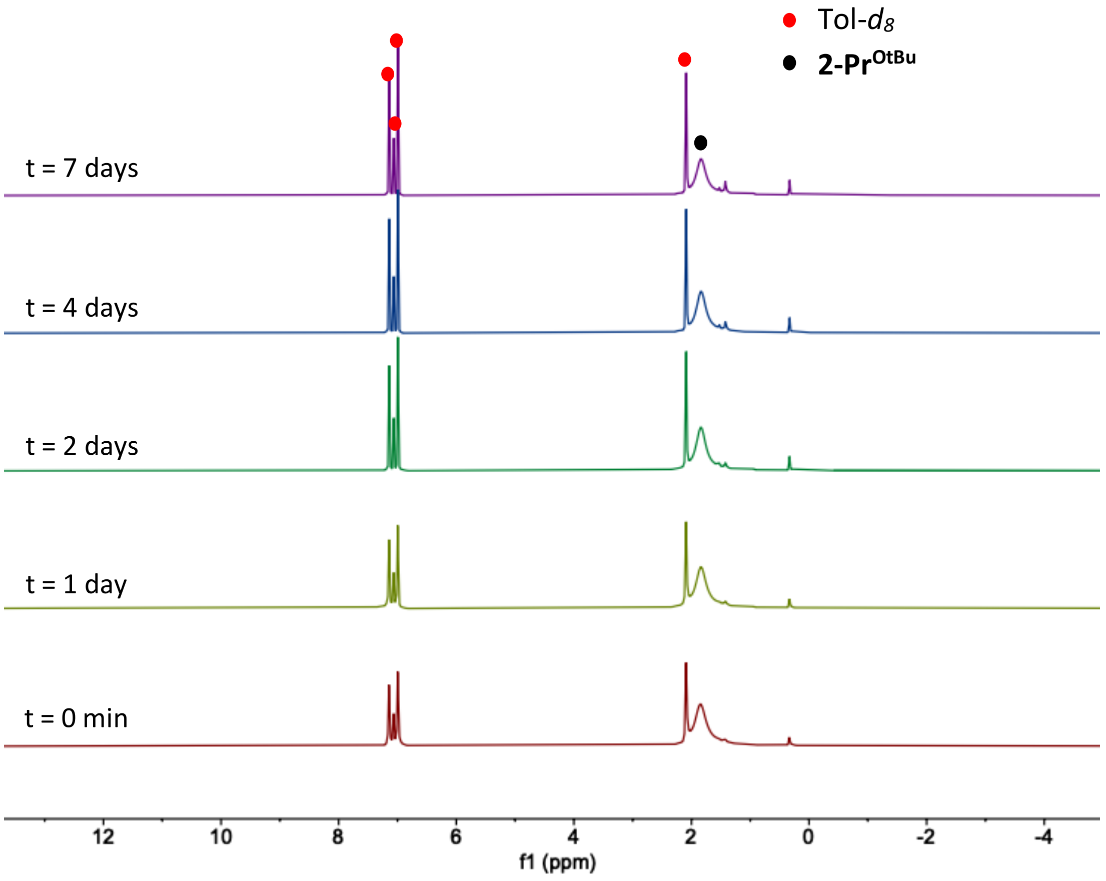

Figure S7. Time dependent ^{1}H NMR (400 MHz, Tol-d$_8$, 233K) solution stability spectra of **2-Pr^{OtBu}**.

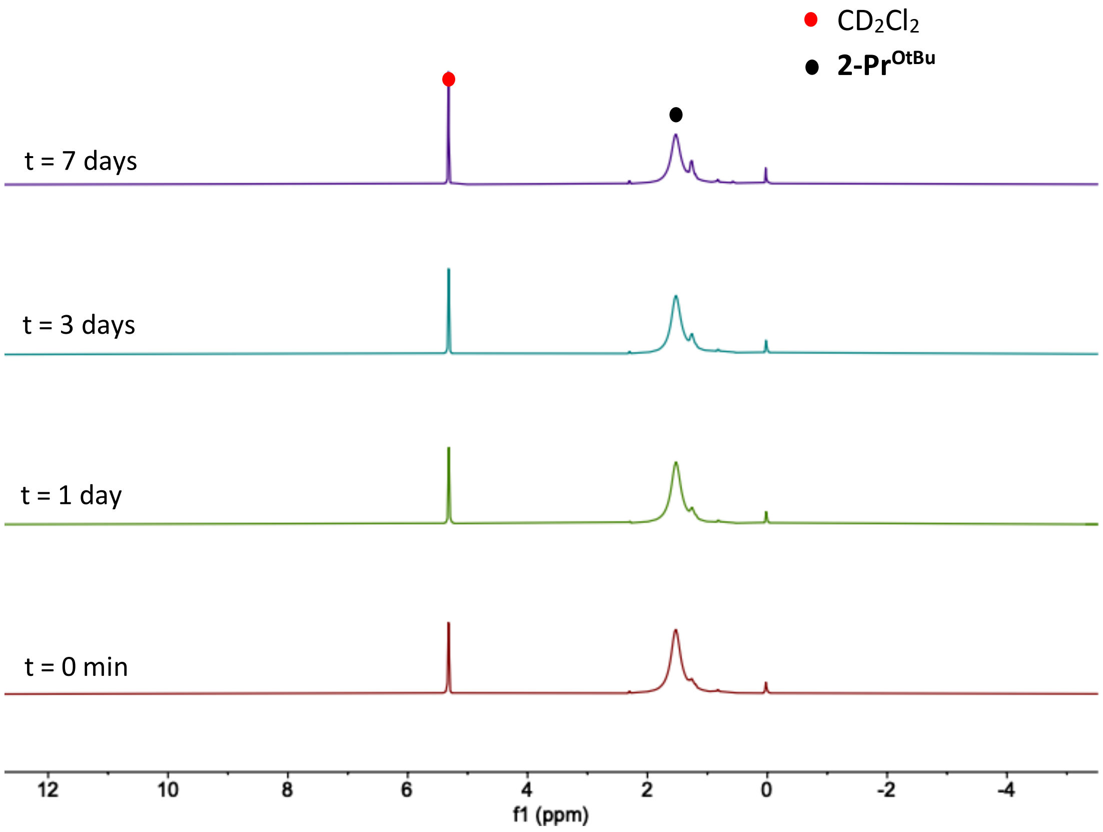

Figure S8. Time dependent ^{1}H NMR (400 MHz, CD_2Cl_2, 233K) solution stability spectra of **2-Pr**$^{\mathbf{OtBu}}$.

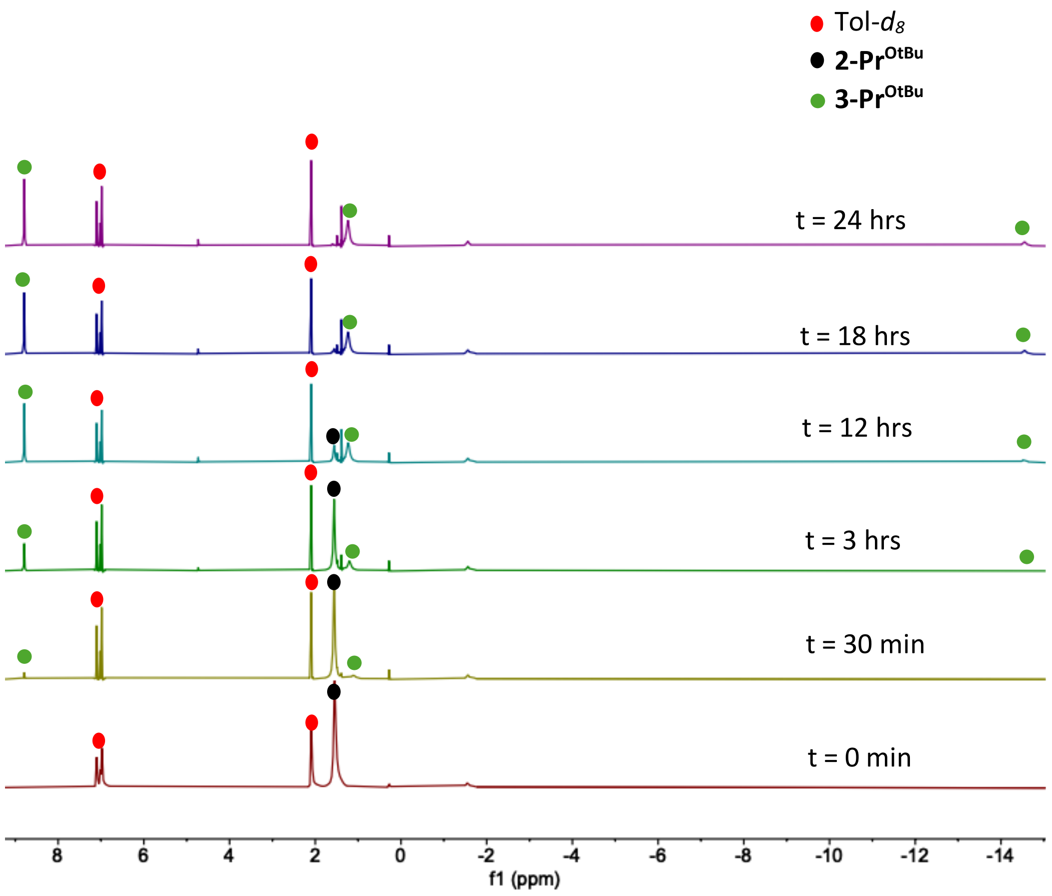

Figure S9. Time dependent ^{1}H NMR (400 MHz, Tol-d_8) spectra of **2-Pr^{OtBu}** at 298K.

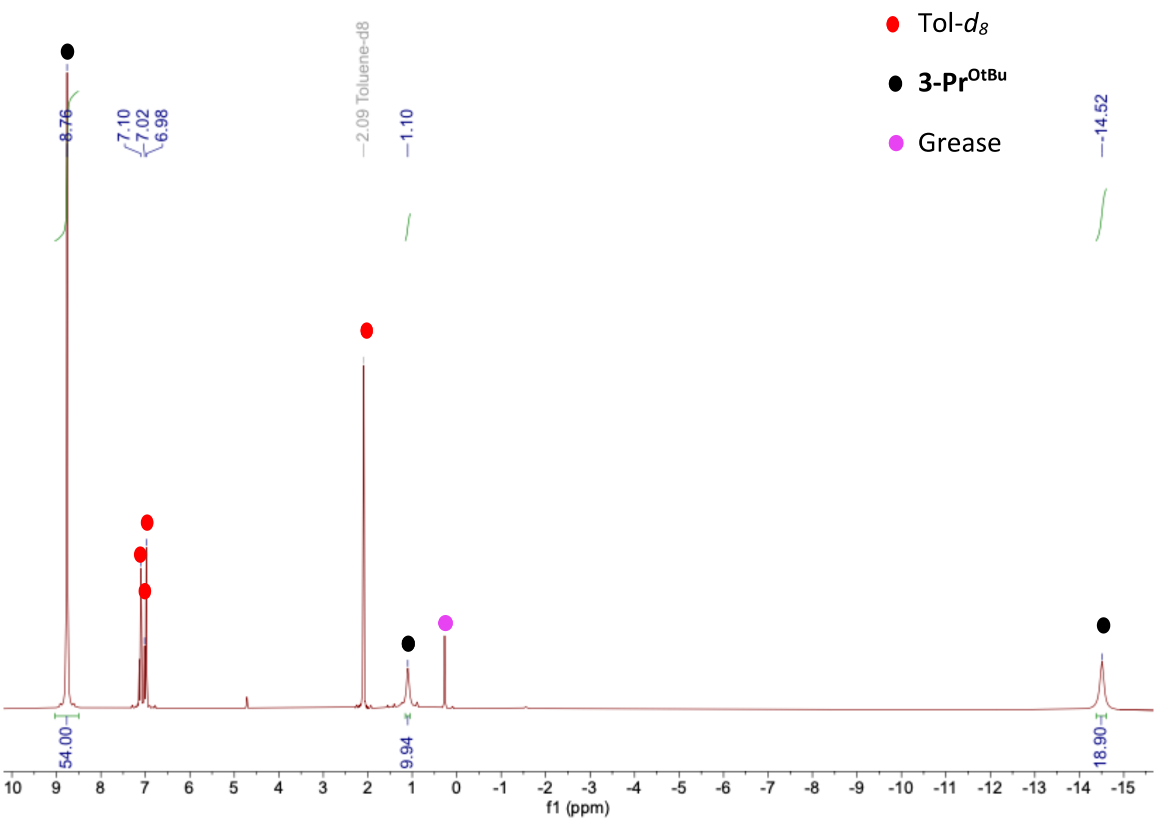

Figure S10. ^{1}H NMR (400 MHz, Tol-d_8, 298K) spectrum of **3-Pr^{OtBu}**.

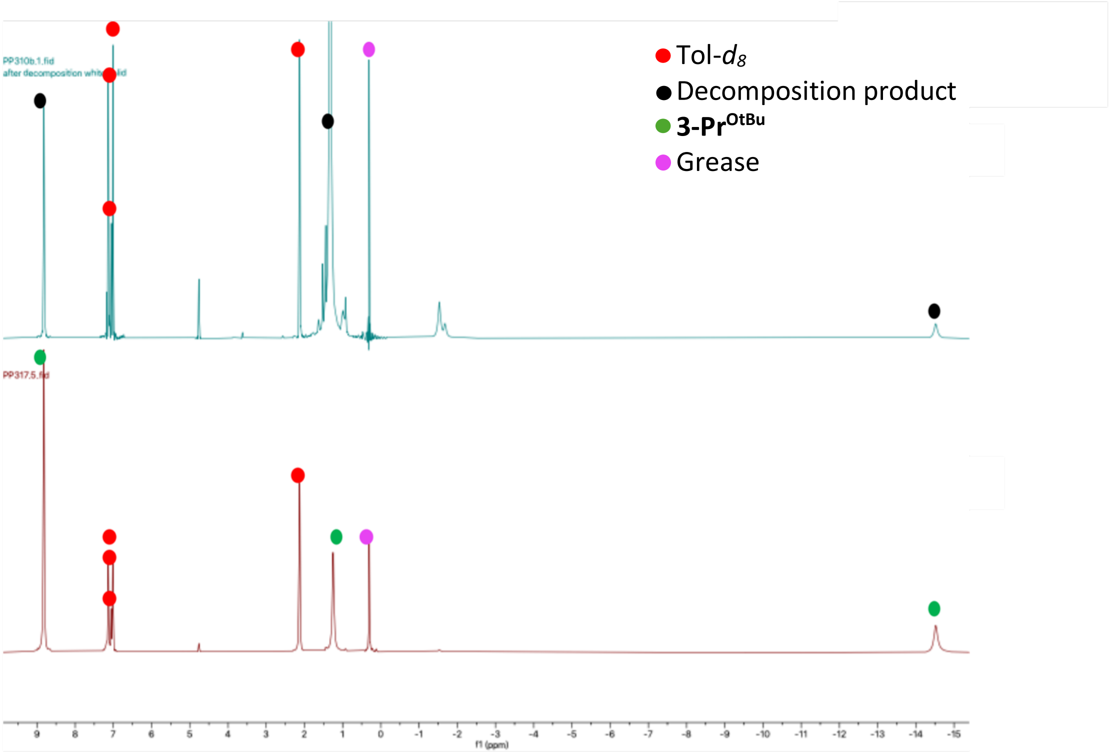

Figure S11. ^{1}H NMR spectrum (400 MHz, Tol-d_8, 298 K) of **2-Pr^{OtBu}** after 18 hours (top) and ^{1}H NMR spectrum of **3-Pr^{OtBu}** at room temperature (bottom).

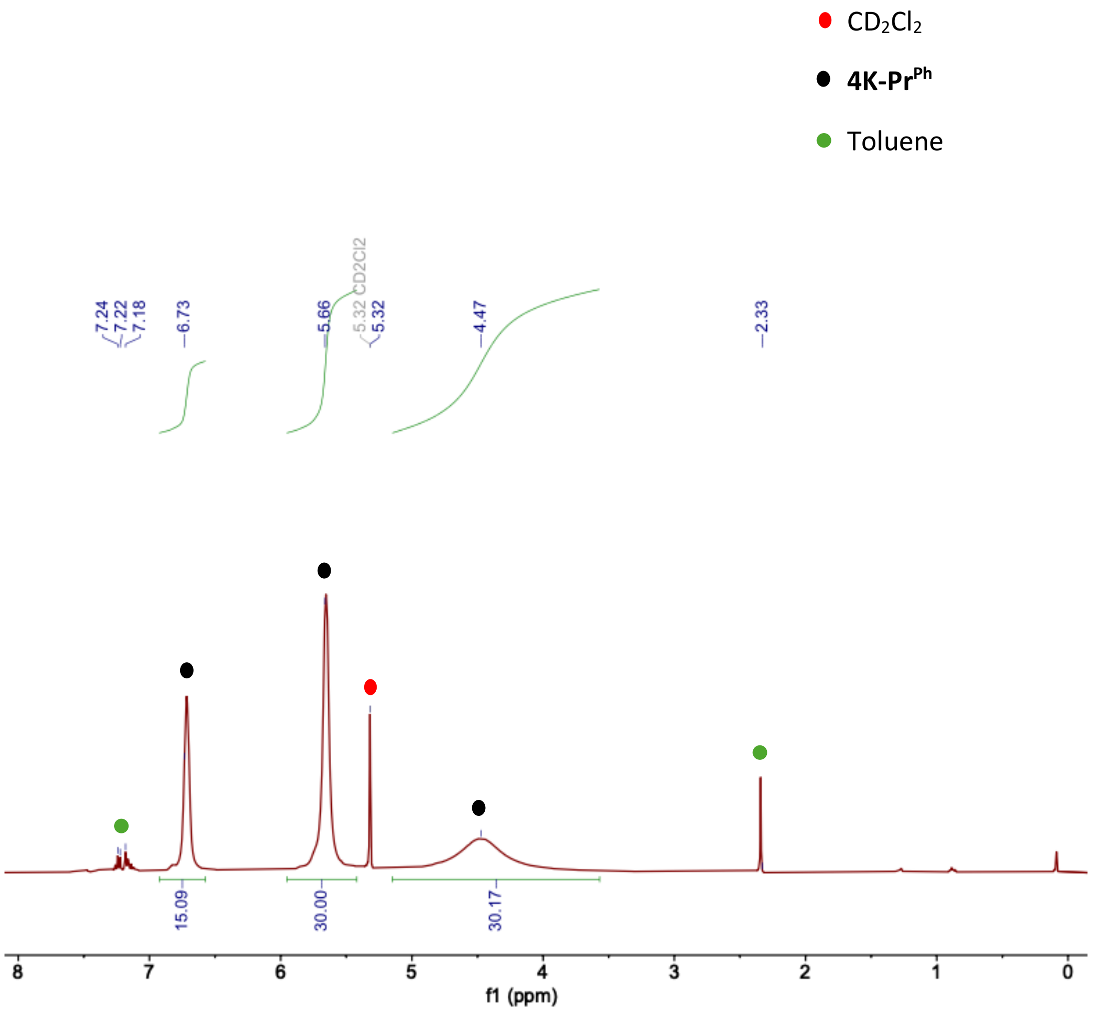

Figure S12. ^{1}H NMR spectrum (400 MHz, CD_2Cl_2 298K) of **4K-Pr^{Ph}**.

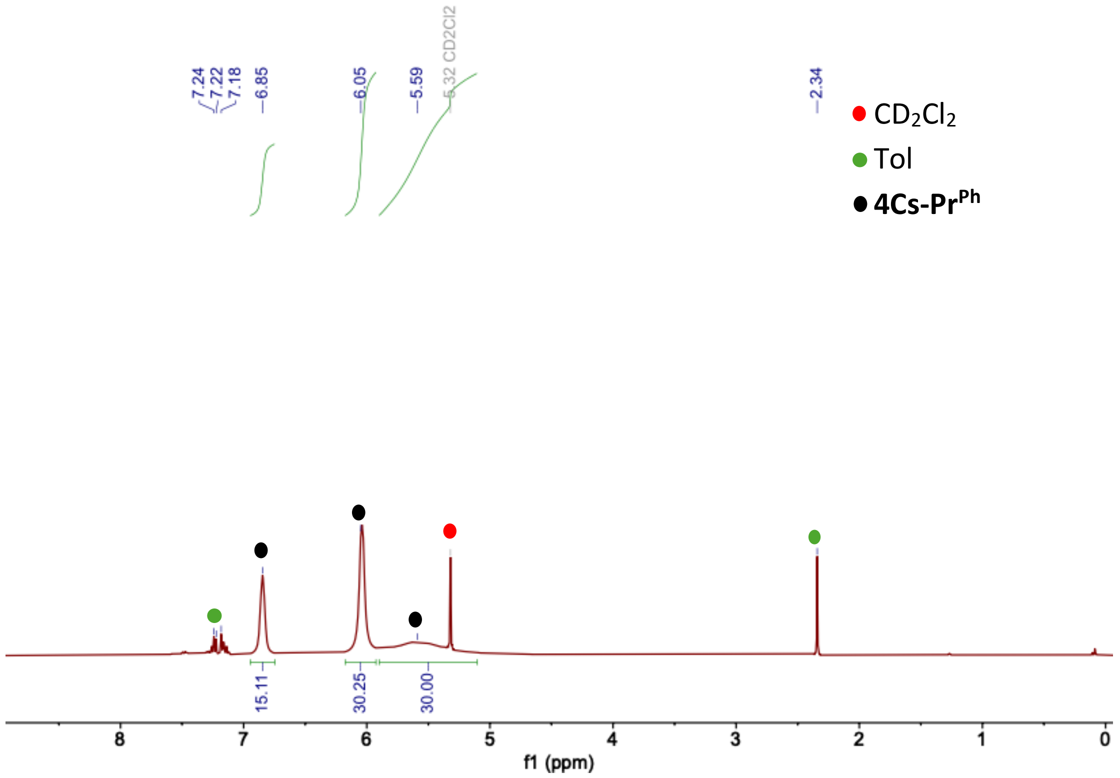

Figure S13. ^{1}H NMR spectrum (400 MHz, CD_2Cl_2, 298K) of **4Cs-Pr^{Ph}**.

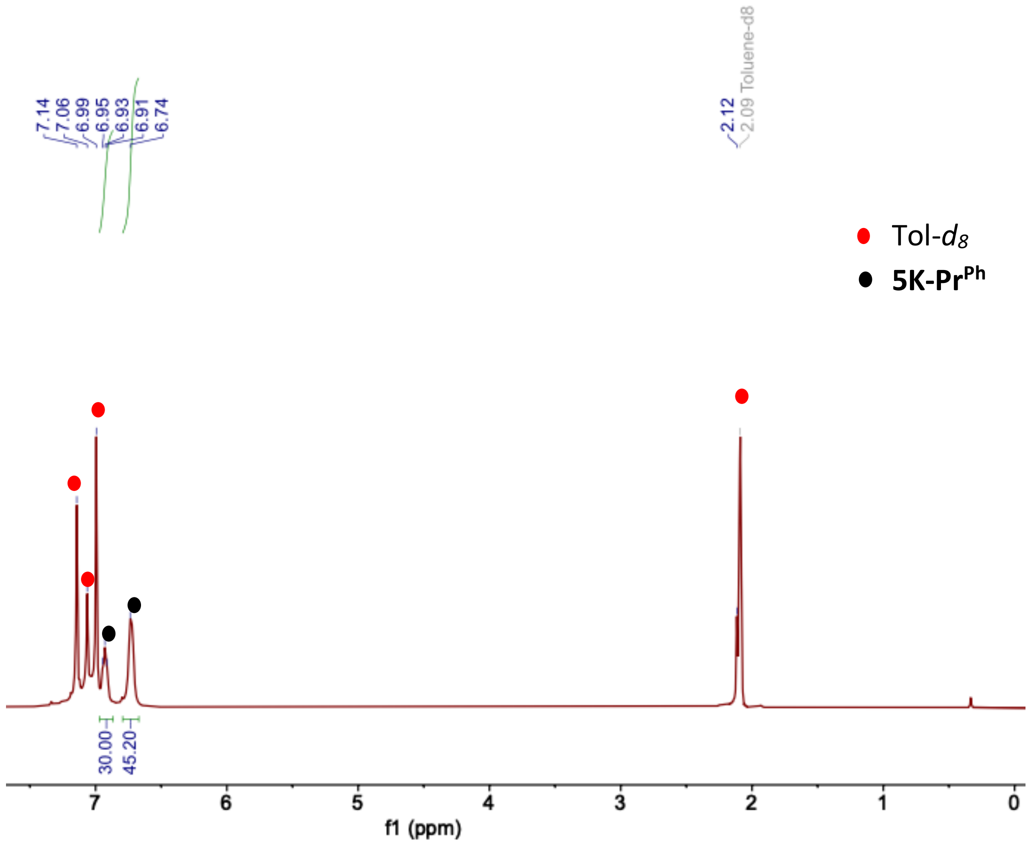

Figure S14. ^{1}H NMR spectrum (400 MHz, Tol-*d*₈ 233K) of **5K-Pr^Ph^**.

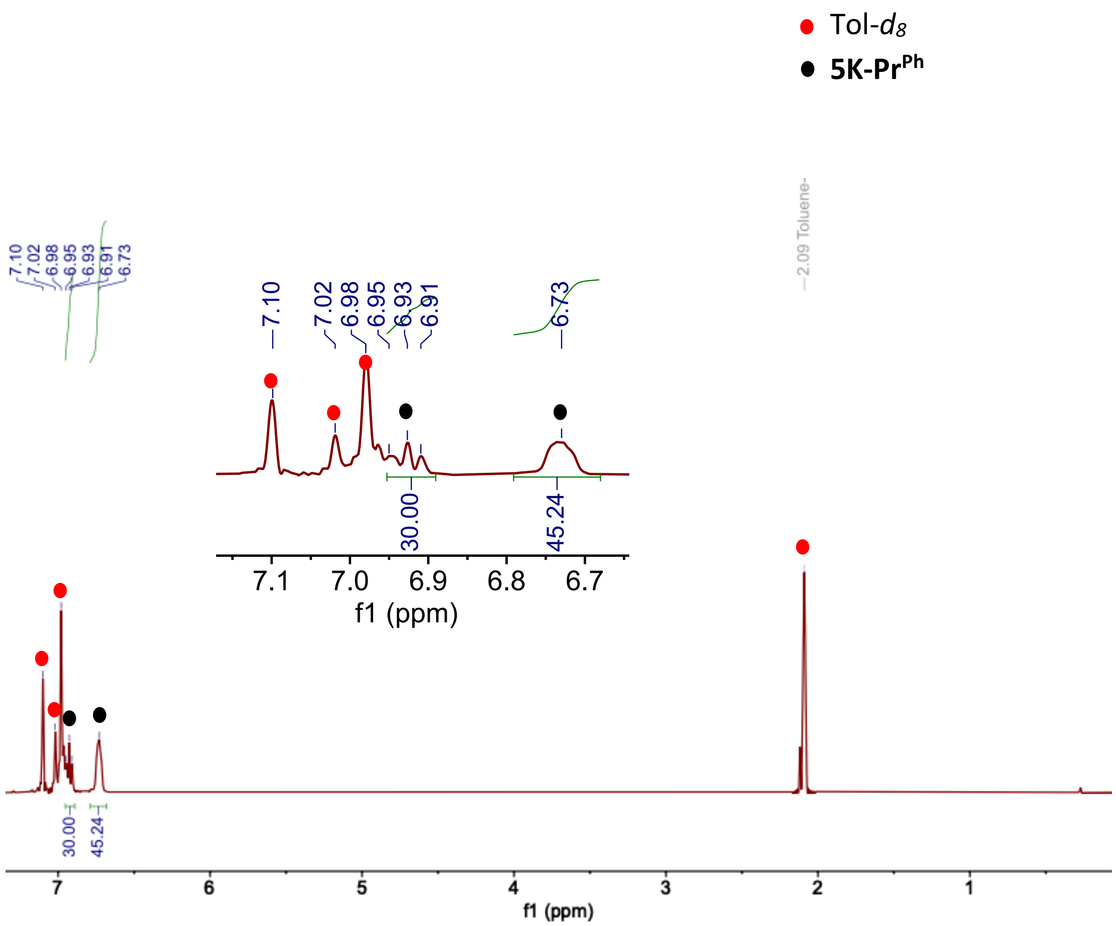

Figure S15. ^{1}H NMR spectrum (400 MHz, Tol-d_8 298K) of **5K-Pr^{Ph}**.

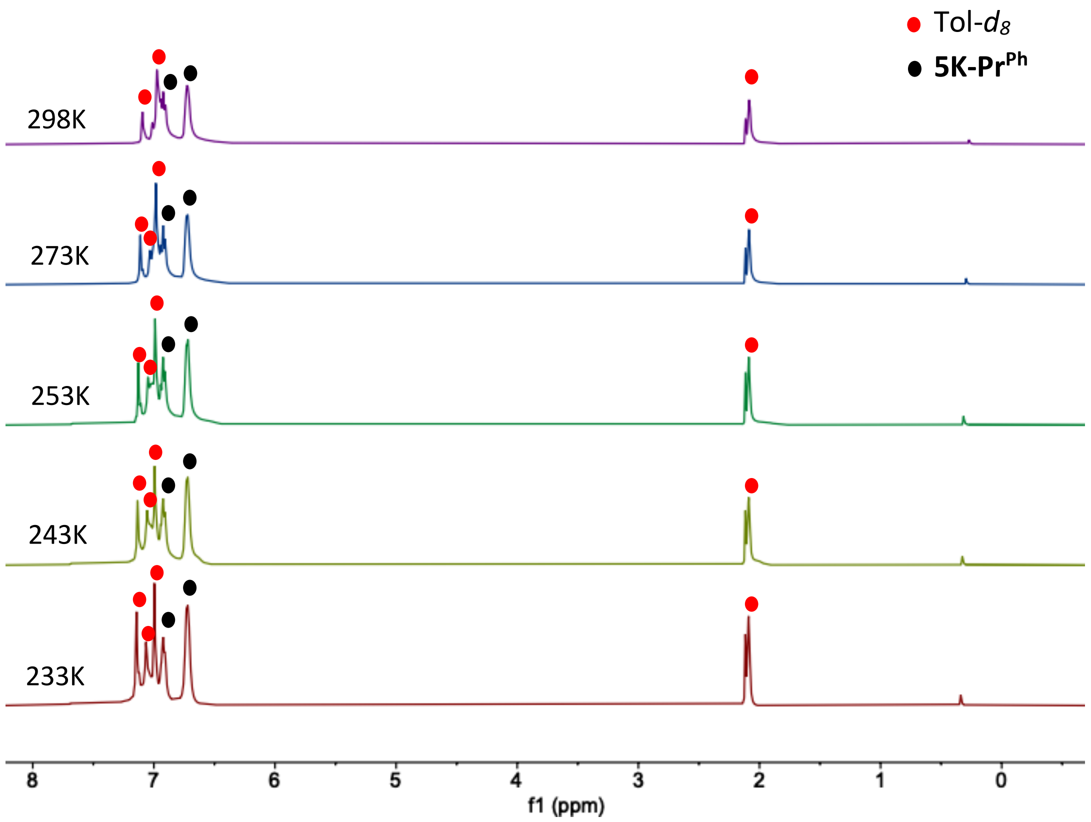

Figure S16. Variable temperature ^{1}H NMR (400 MHz, Tol-d_8) spectra of **5K-Pr^{Ph}**.

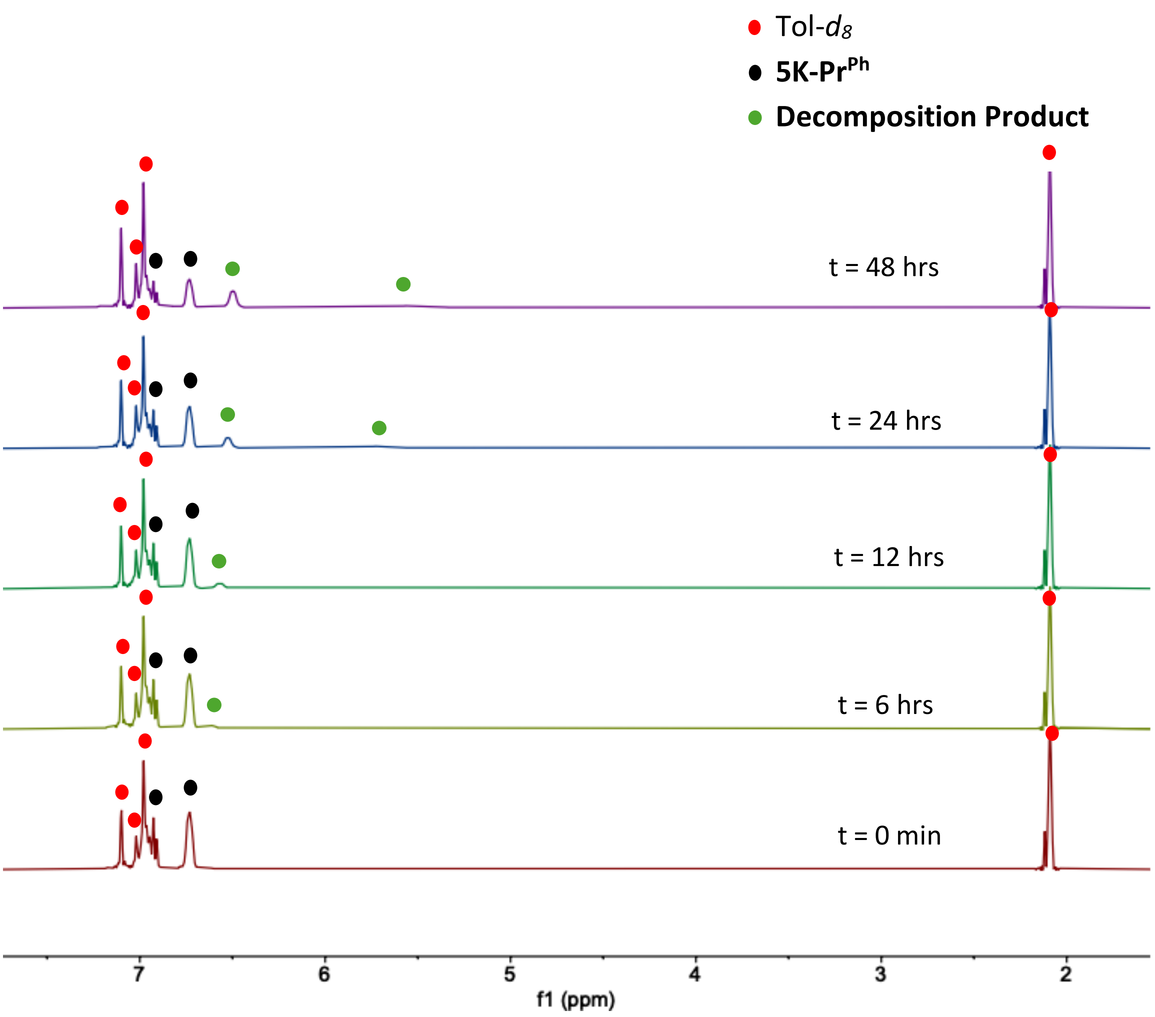

Figure S17. Time dependent ^{1}H NMR (400 MHz, Tol-d_8) spectra of **5K-Pr^{Ph}** at 298K.

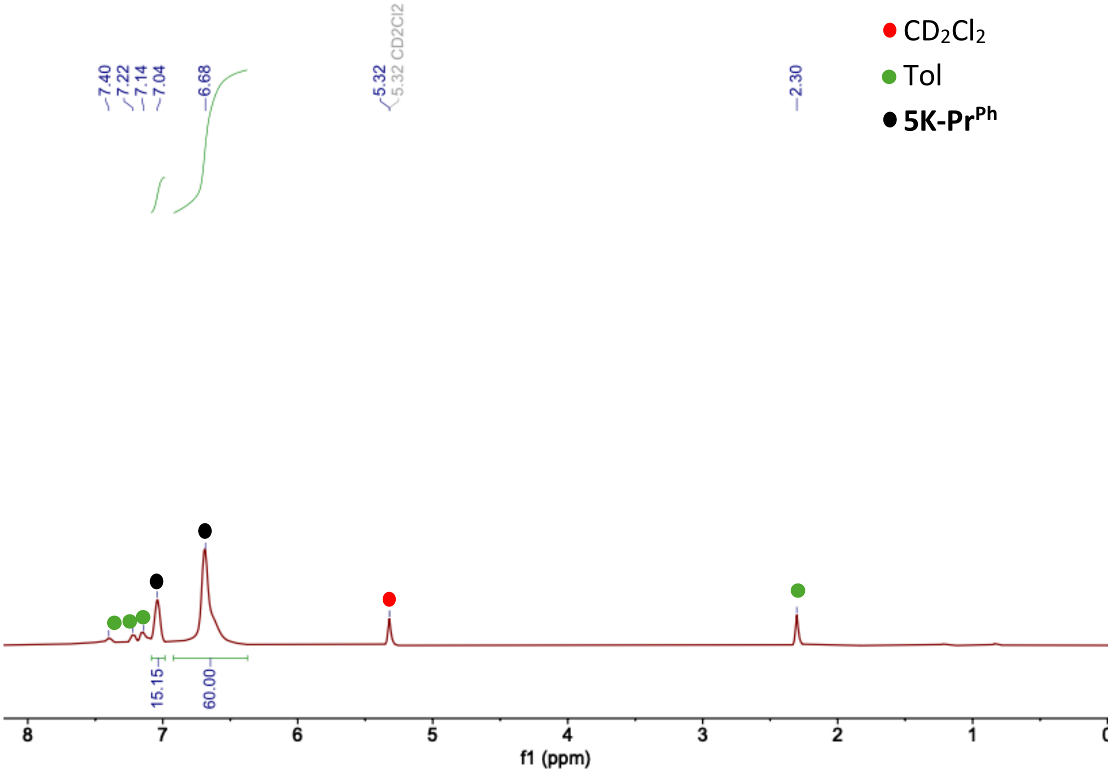

Figure S18. ^{1}H NMR (400 MHz, CD_2Cl_2) spectrum of **5K-Pr^{Ph}** at 233K.

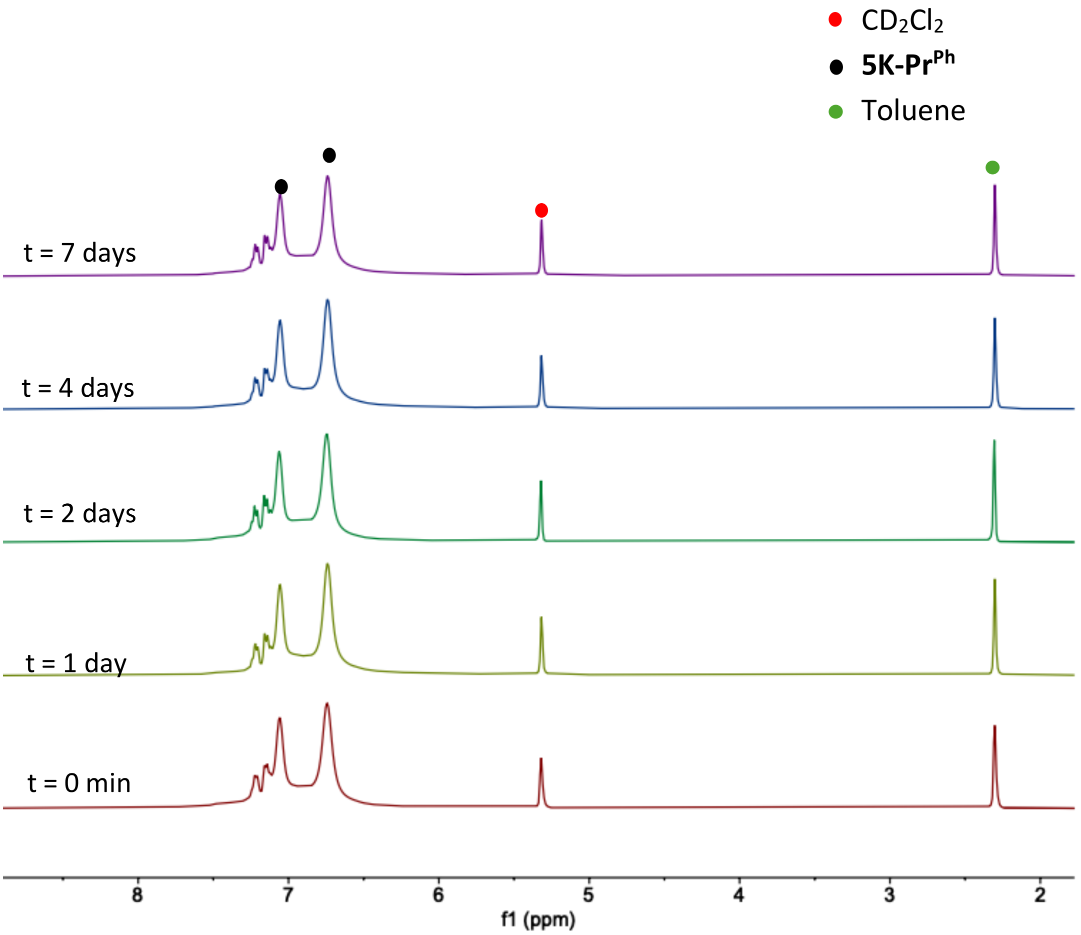

Figure S19. Time dependent ^{1}H NMR (400 MHz, CD_2Cl_2, 233K) solution stability spectra of **5K-Pr^{Ph}**.

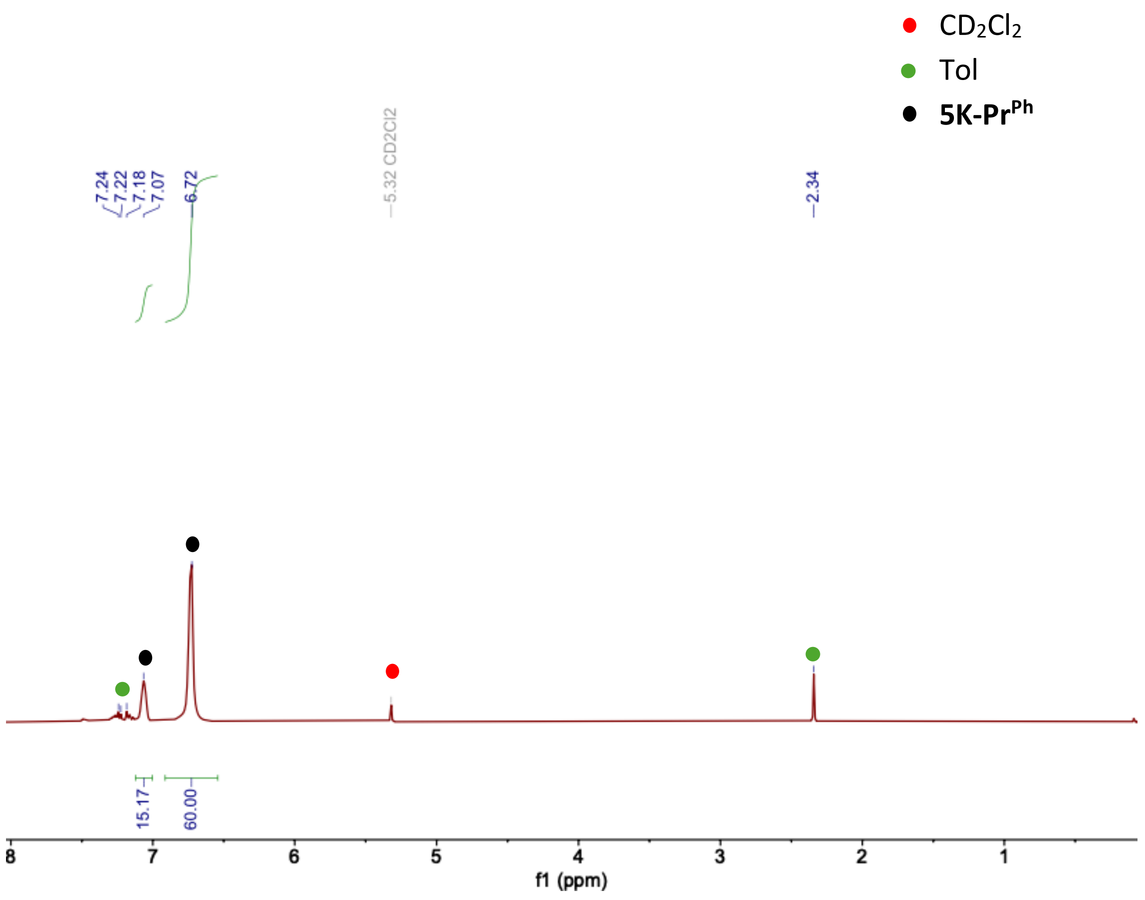

Figure S20. ^{1}H NMR (400 MHz, CD_2Cl_2) spectrum of **5K-Pr^{Ph}** at 298K.

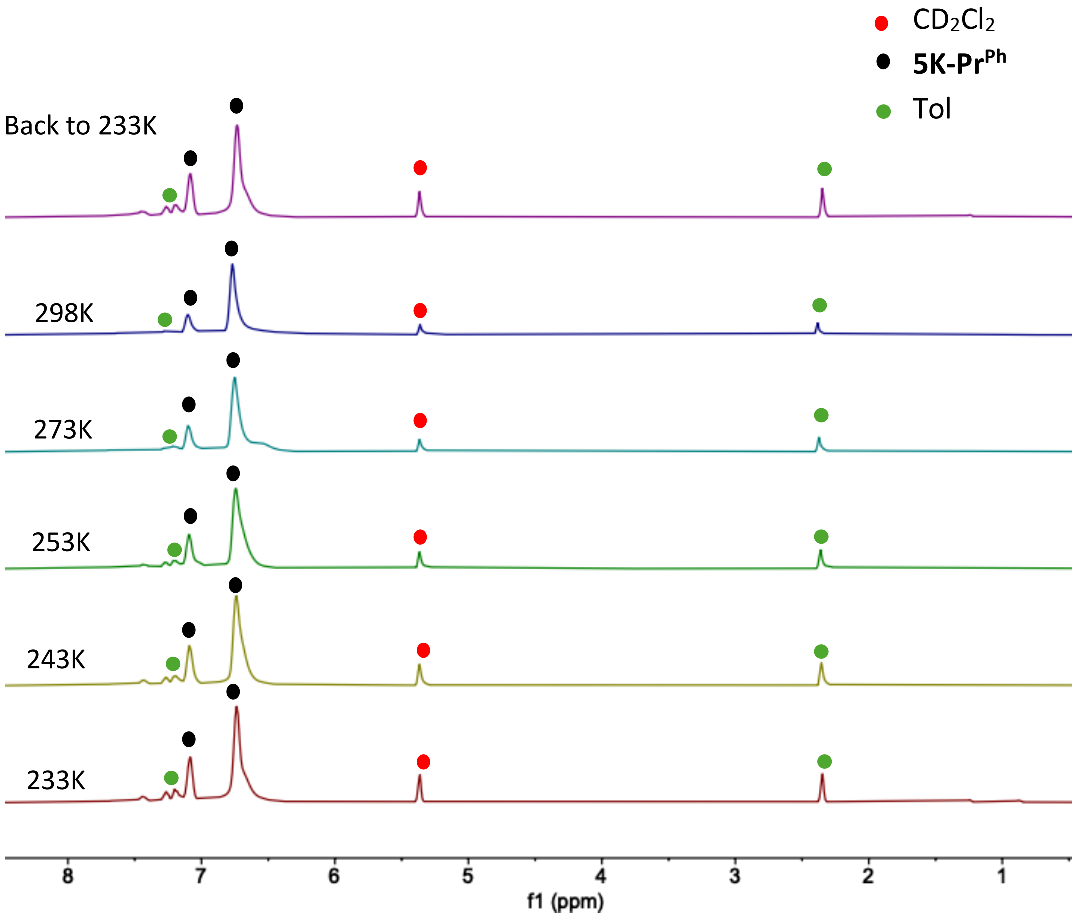

Figure S21. Variable temperature ^{1}H NMR (400 MHz, CD_2Cl_2) spectra of **5K-Pr^{Ph}**.

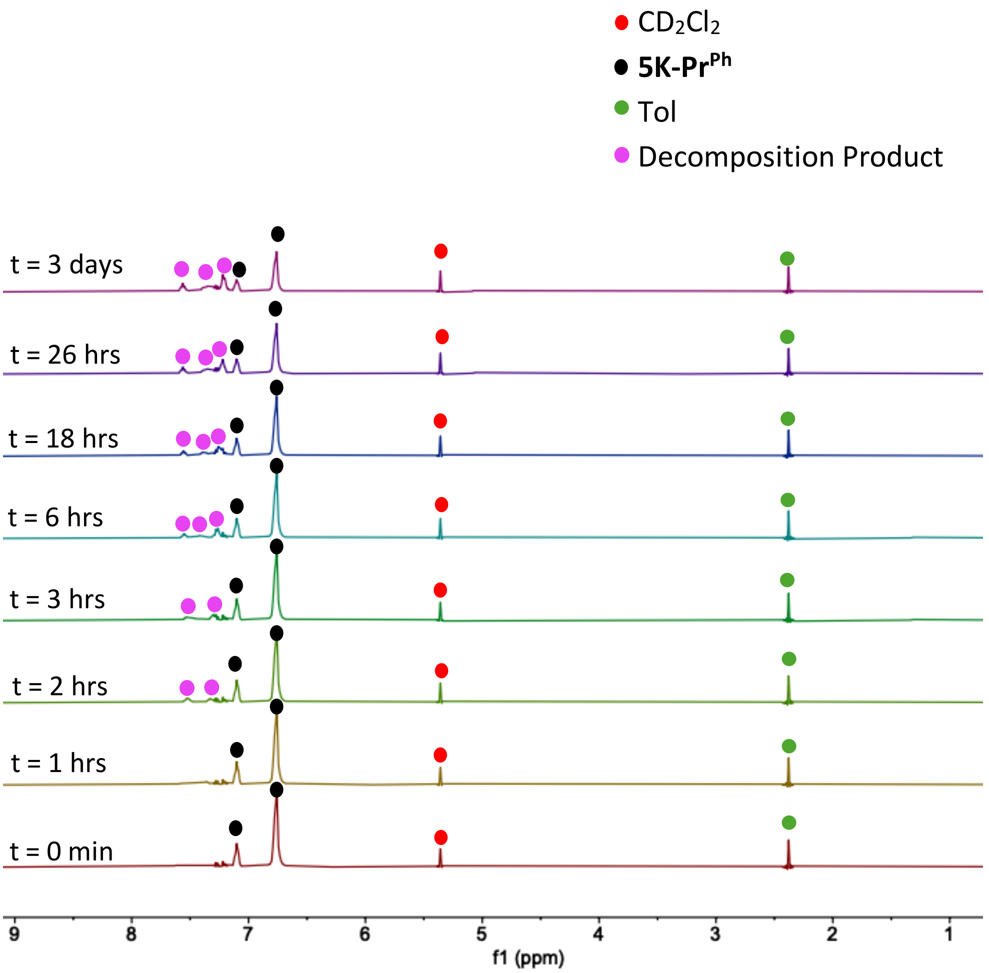

Figure S22. Time dependent ^{1}H NMR (400 MHz, CD_2Cl_2) spectra of **5K-Pr^{Ph}** at 298K.

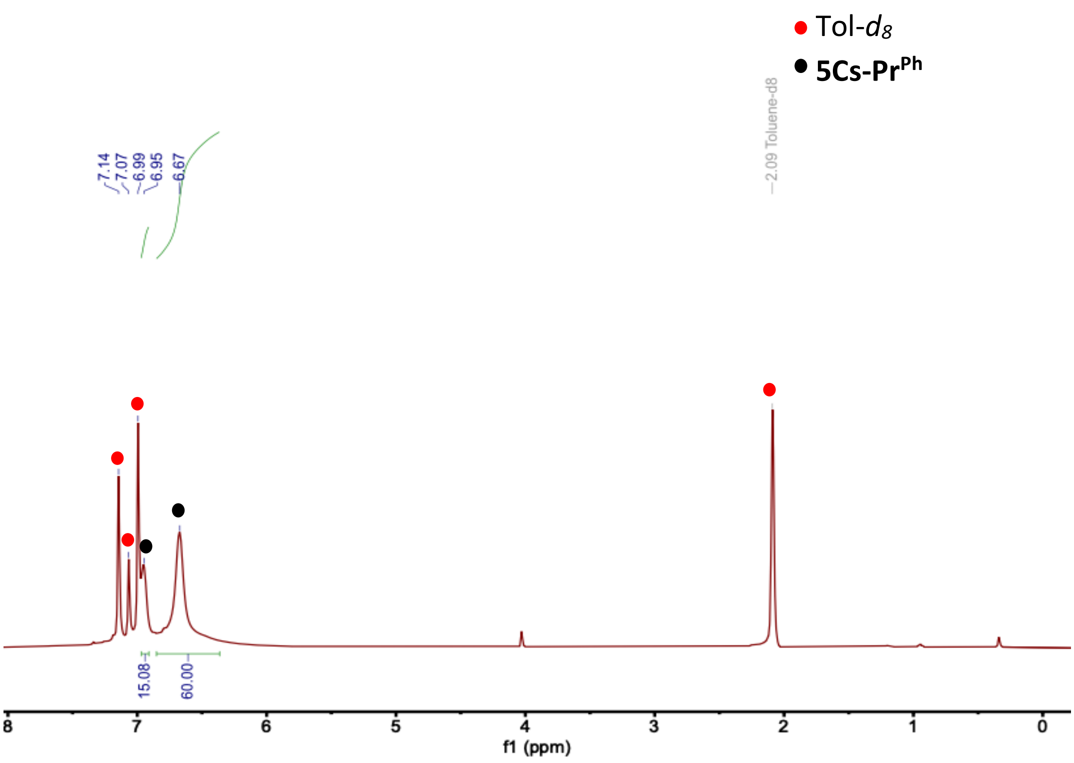

Figure S23. ^{1}H NMR (400 MHz, Tol-d_8) spectrum of **5Cs-Pr^{Ph}** at 233K.

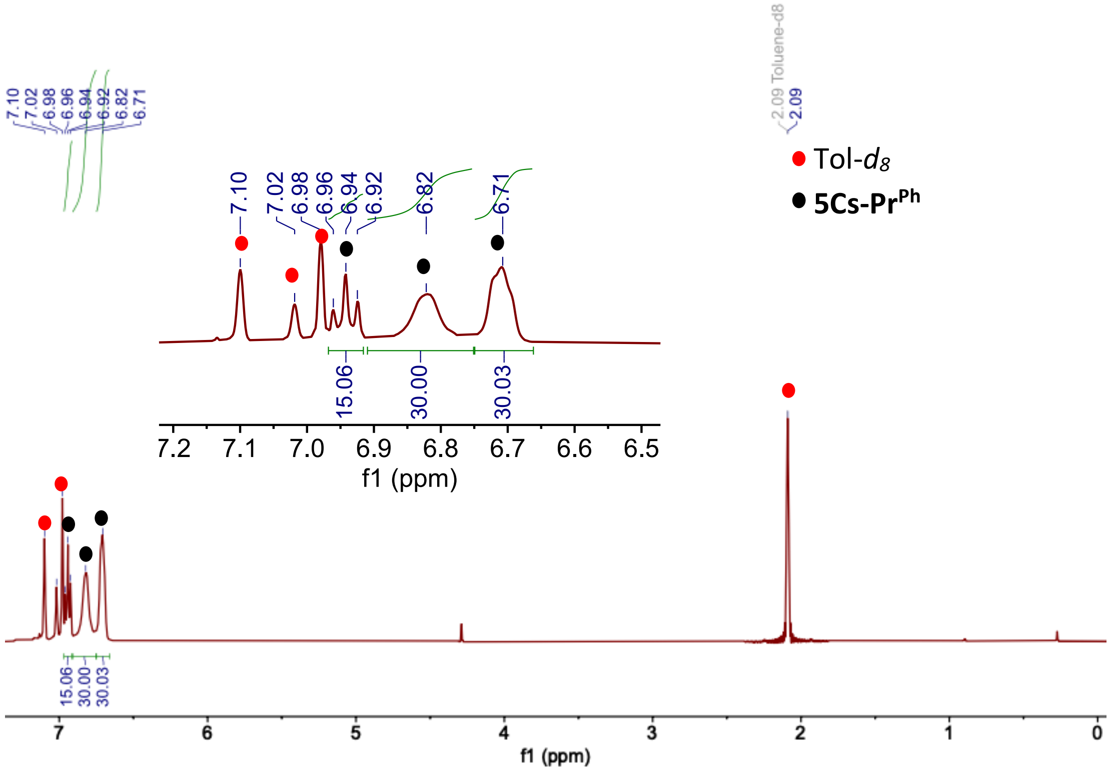

Figure S24. ^{1}H NMR (400 MHz, Tol-d_8) spectrum of **5Cs-Pr^{Ph}** at 298K.

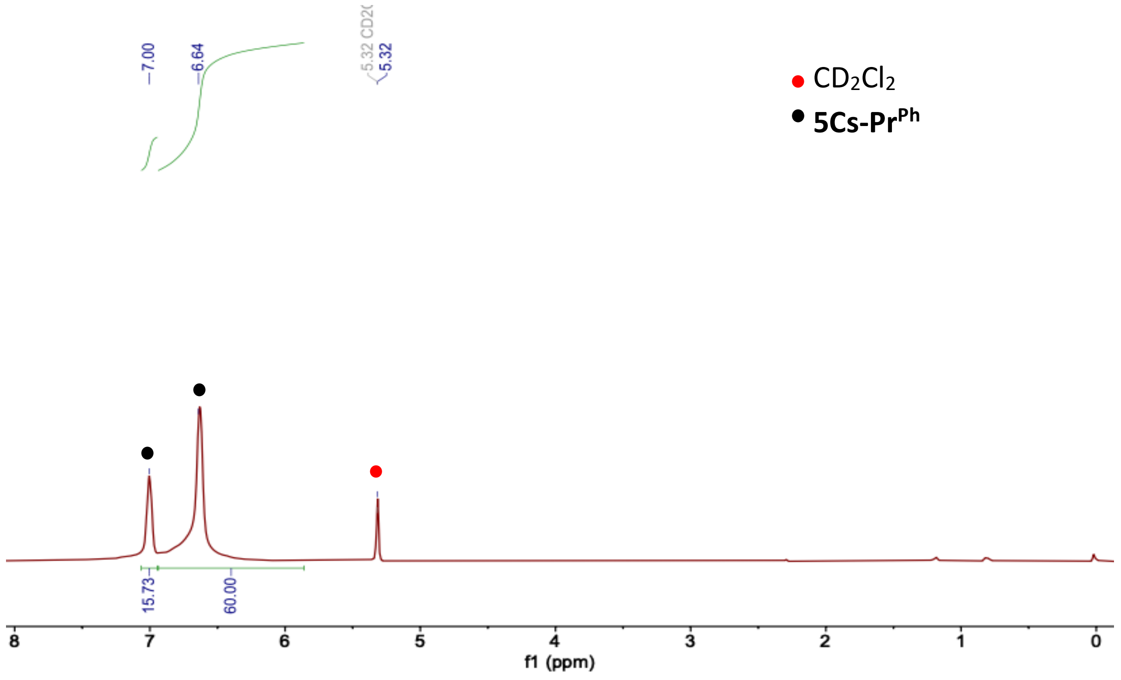

Figure S25. ^{1}H NMR (400 MHz, CD_2Cl_2) spectrum of **5Cs-Pr^{Ph}** at 233K.

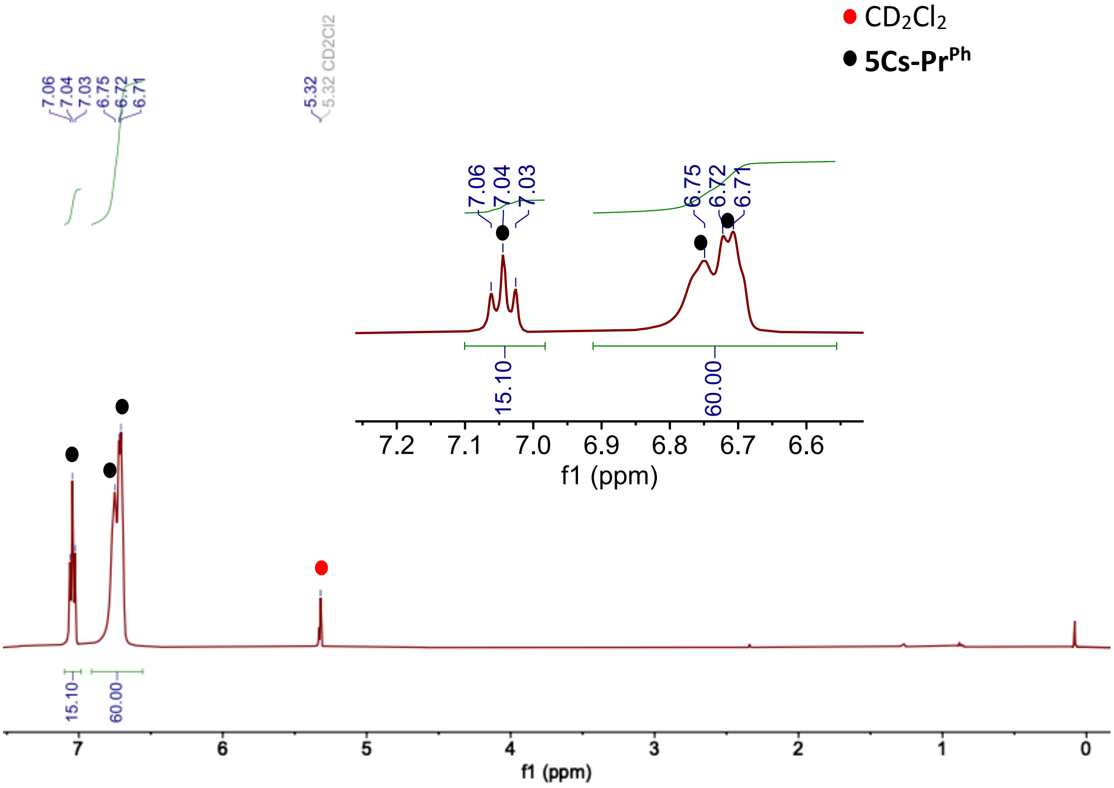

Figure S26. ^{1}H NMR (400 MHz, CD_2Cl_2) spectrum of **5Cs-Pr^{Ph}** at 298K.

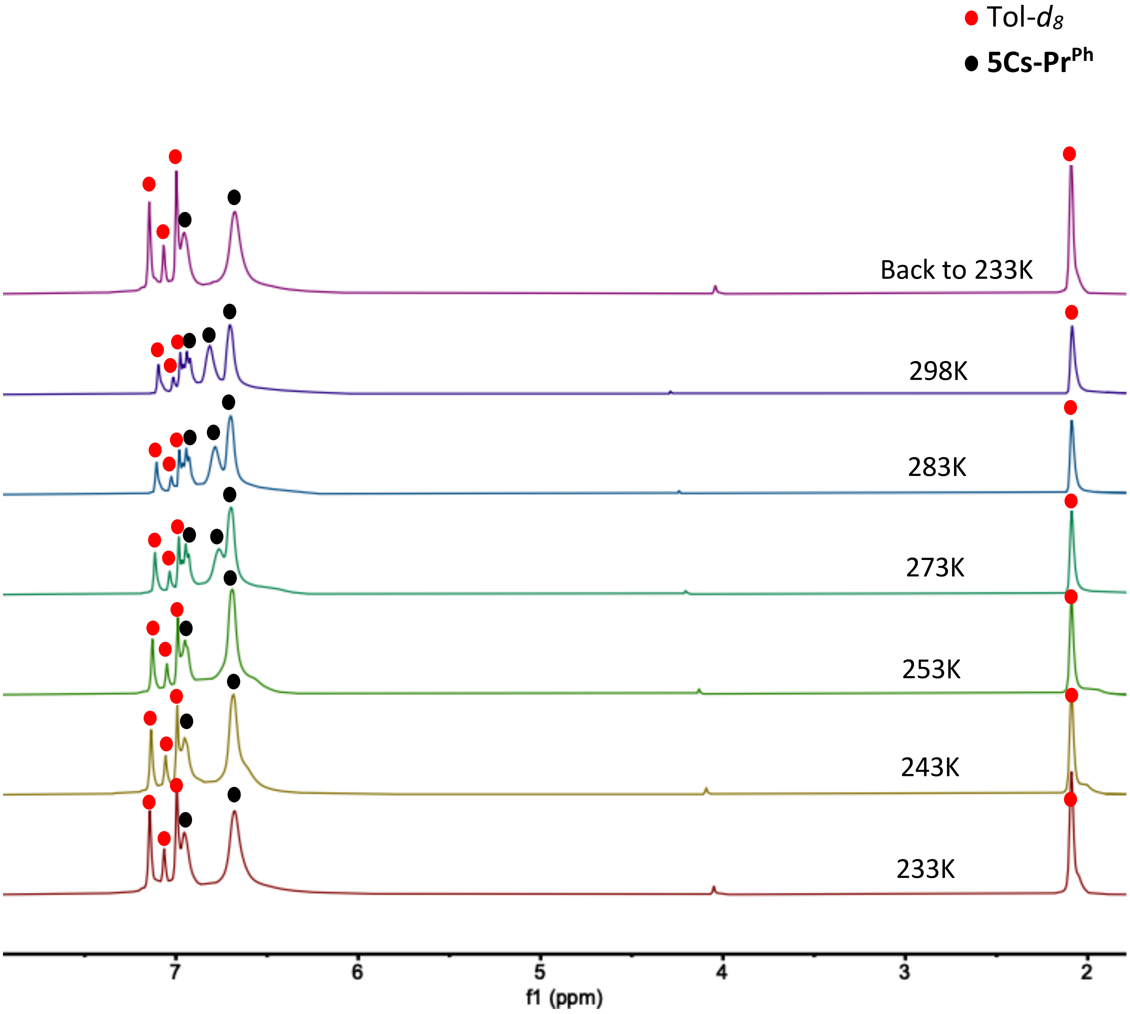

Figure S27. Variable temperature ^{1}H NMR (400 MHz, Tol-d_8) spectra of **5Cs-Pr^{Ph}.**

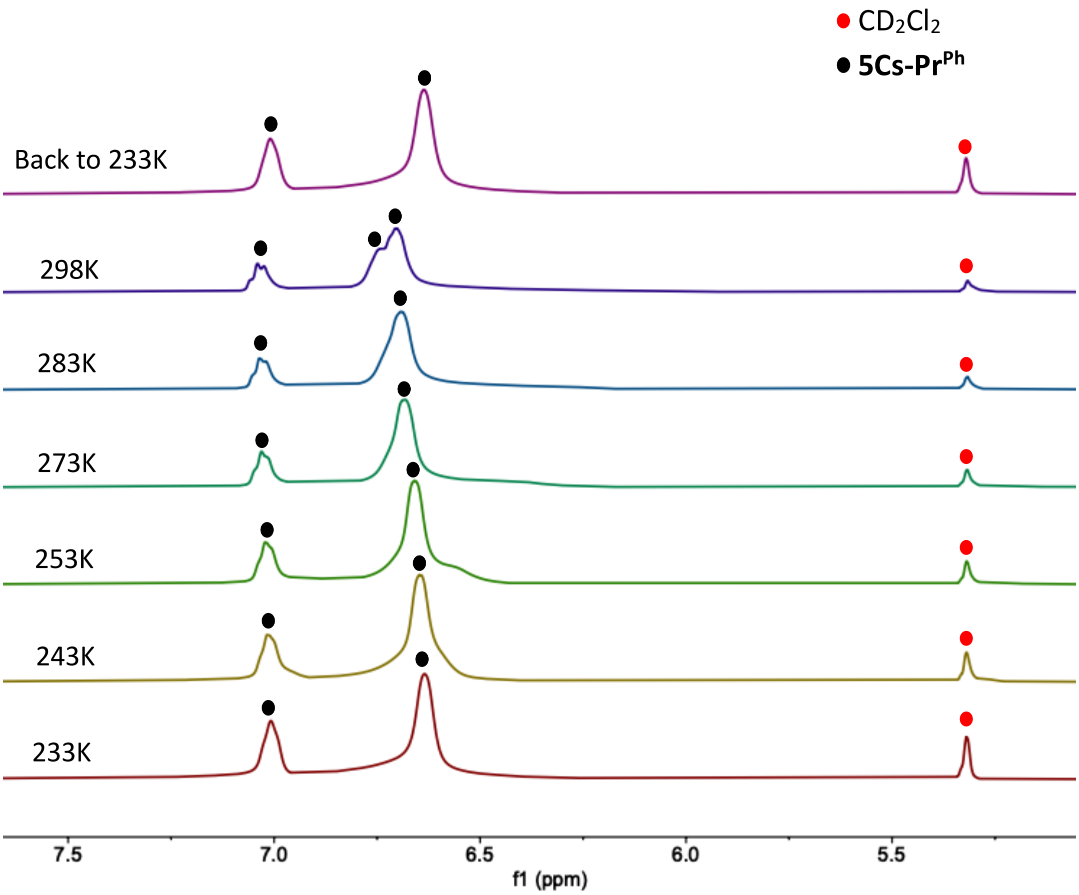

Figure S28. Variable temperature ^{1}H NMR (400 MHz, CD_2Cl_2) spectra of **5Cs-Pr^{Ph}.**

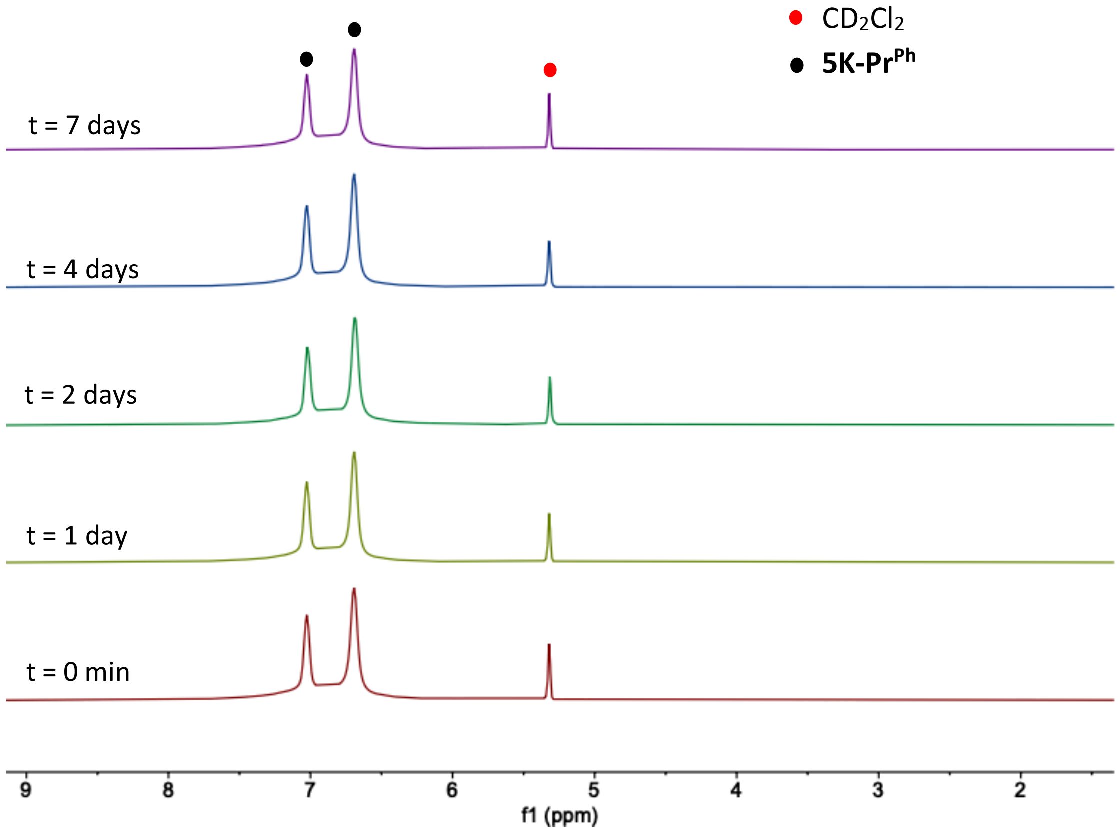

Figure S29. Time dependent ^{1}H NMR (400 MHz, CD_2Cl_2, 233K) solution stability spectra of **5Cs-Pr^{Ph}**.

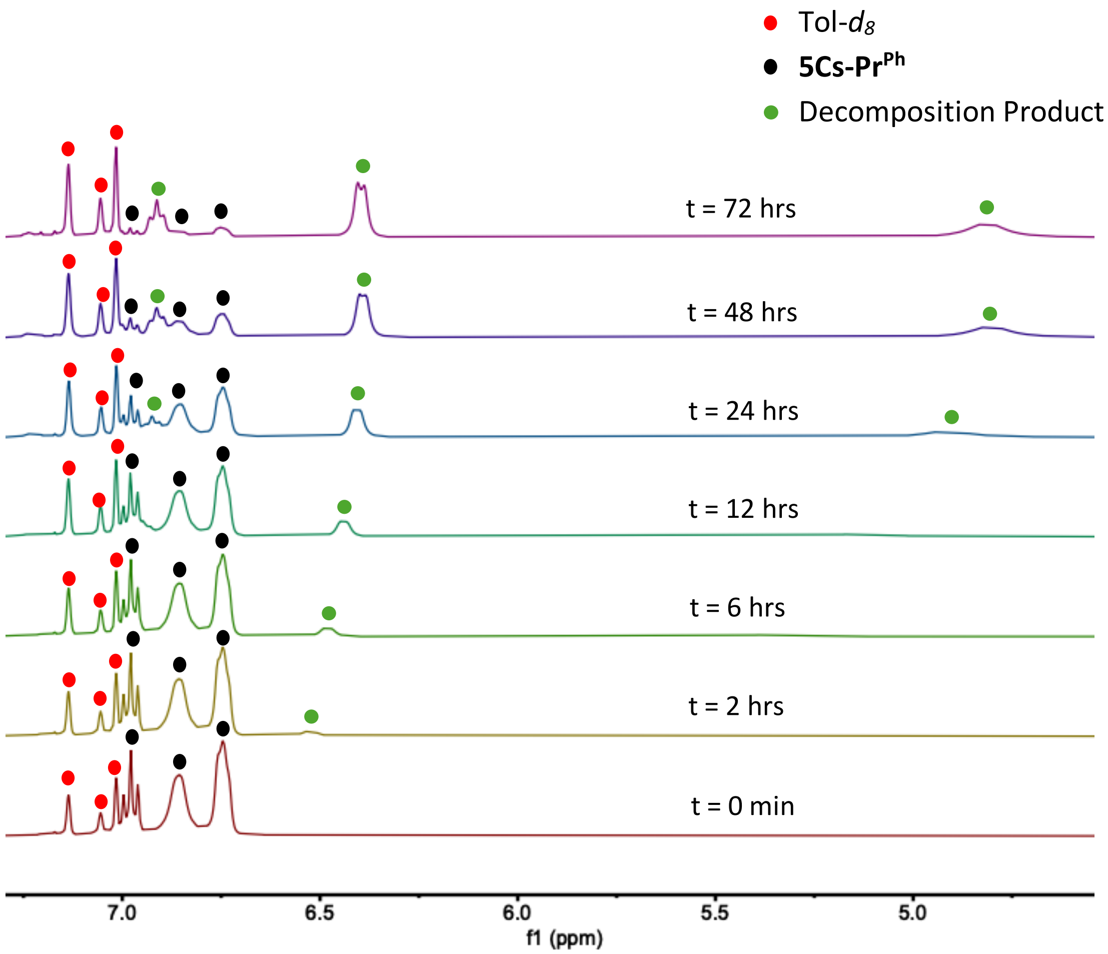

Figure S30. Time dependent ^{1}H NMR (400 MHz, Tol-d_8) spectra of **5Cs-Pr^{Ph}** at 298K.

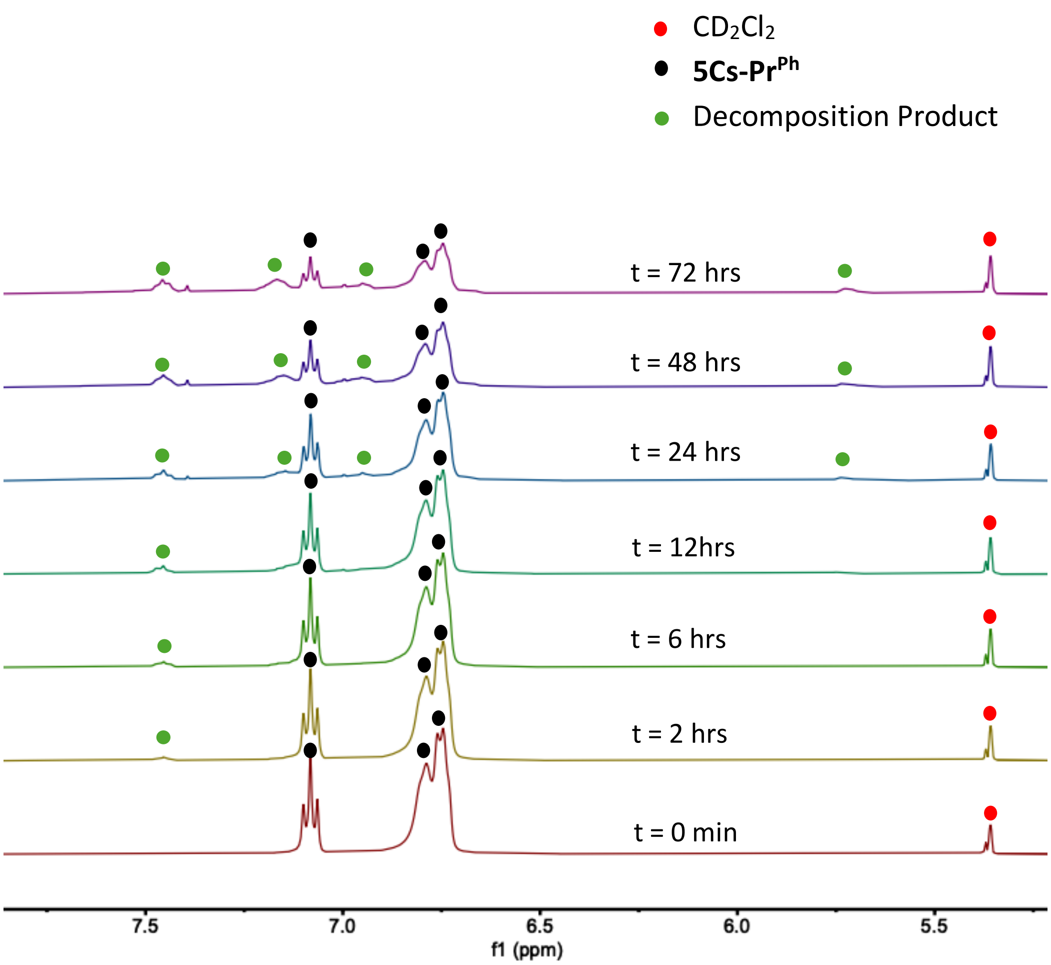

Figure S31. Time dependent ^{1}H NMR (400 MHz, CD_2Cl_2) spectra of **5Cs-Pr^{Ph}** at 298K.

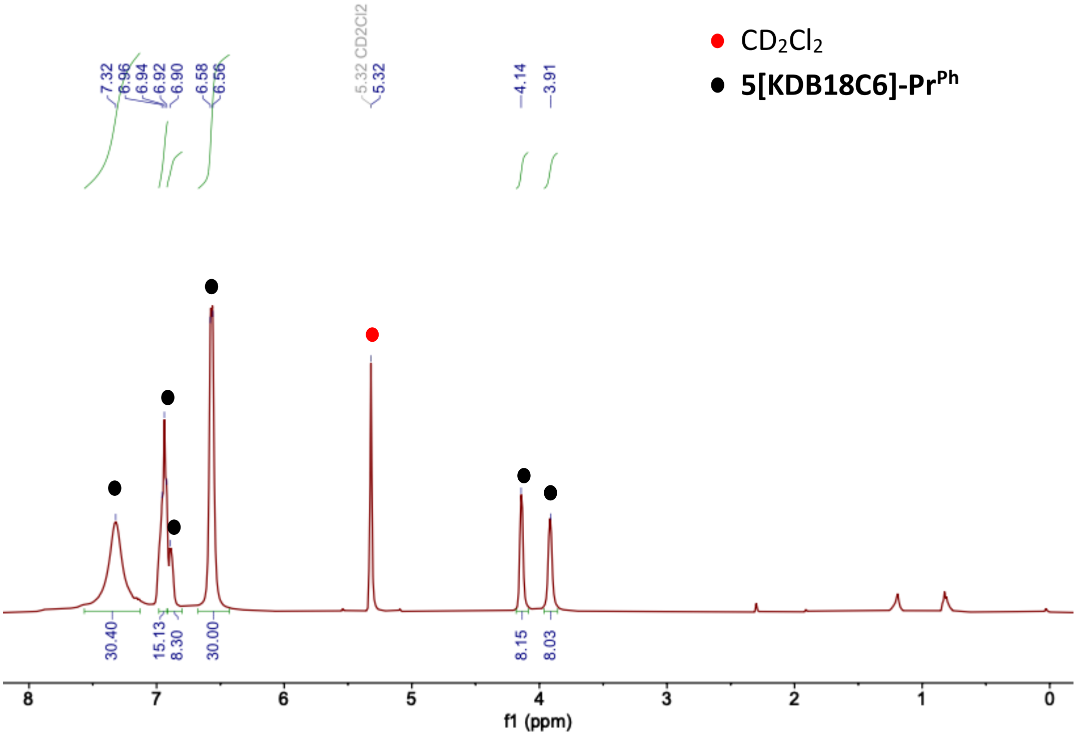

Figure S32. ^{1}H NMR (400 MHz, CD_2Cl_2) spectrum of **5[KDB18C6]-Pr^{Ph}** at 233K.

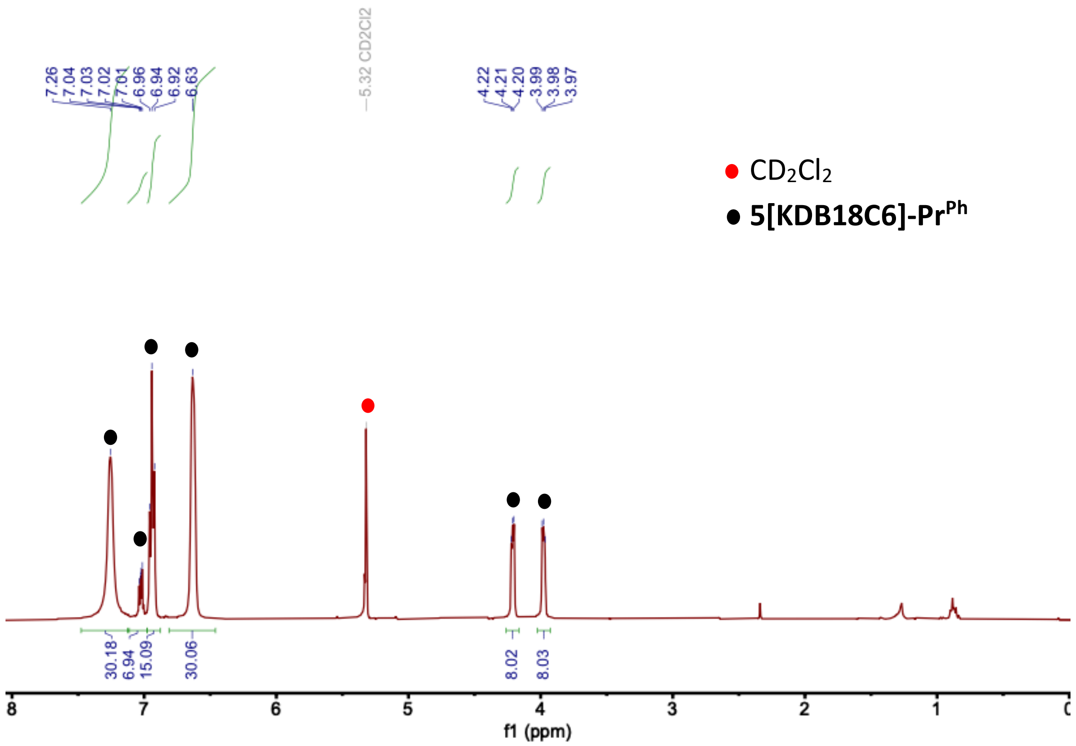

Figure S33. ^{1}H NMR (400 MHz, CD_2Cl_2) spectrum of **5[KDB18C6]-Pr^{Ph}** at 298K.

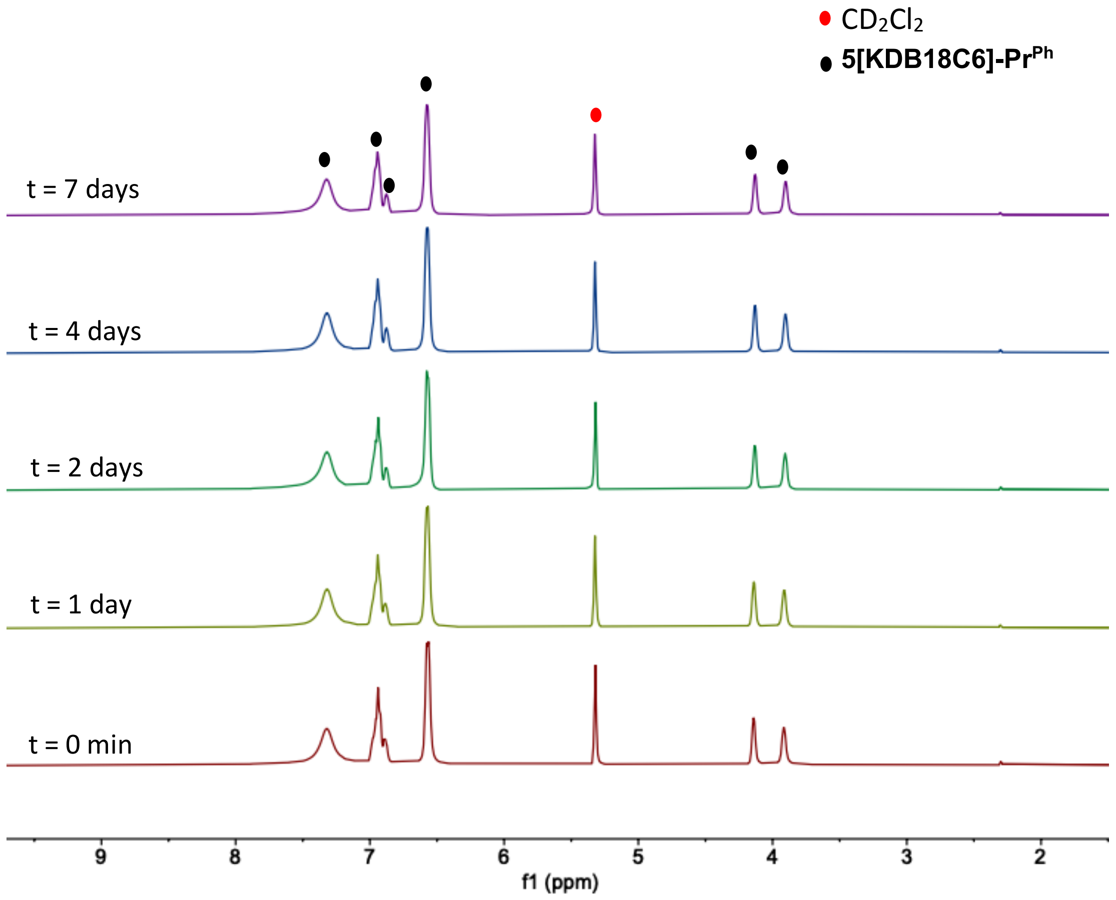

Figure S34. Time dependent ^{1}H NMR (400 MHz, CD_2Cl_2, 233K) solution stability spectra of **5[KDB18C6]-Pr^{Ph}**.

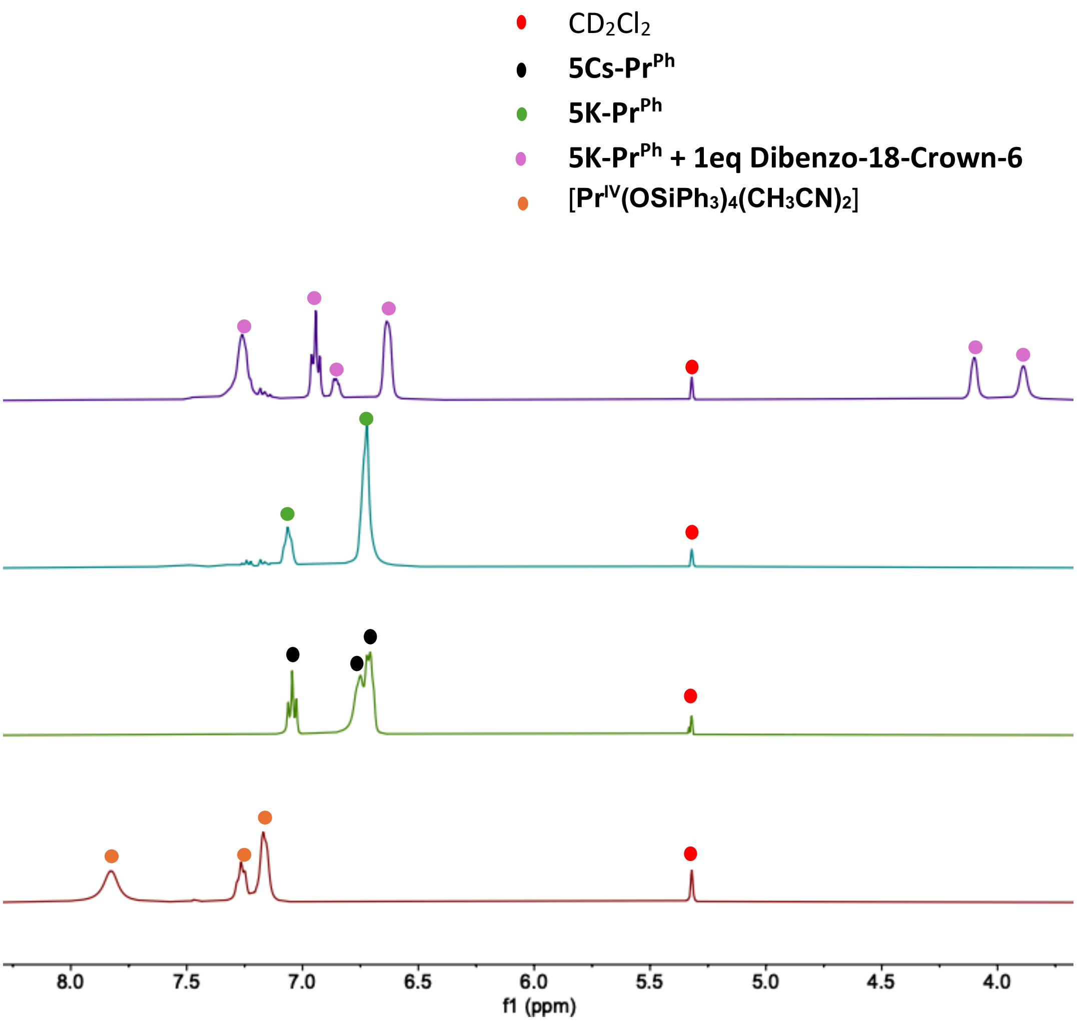

Figure S35. Comparative ^{1}H NMR (400 MHz, CD_2Cl_2) spectra of [**Pr^{IV}(OSiPh$_3$)$_4$(CH$_3$CN)$_2$**], **5Cs-Pr^{Ph}**, **5K-Pr^{Ph}** and **5K-Pr^{Ph}** after addition of 1 eq of Dibenzo-18-crown-6 at 298K.

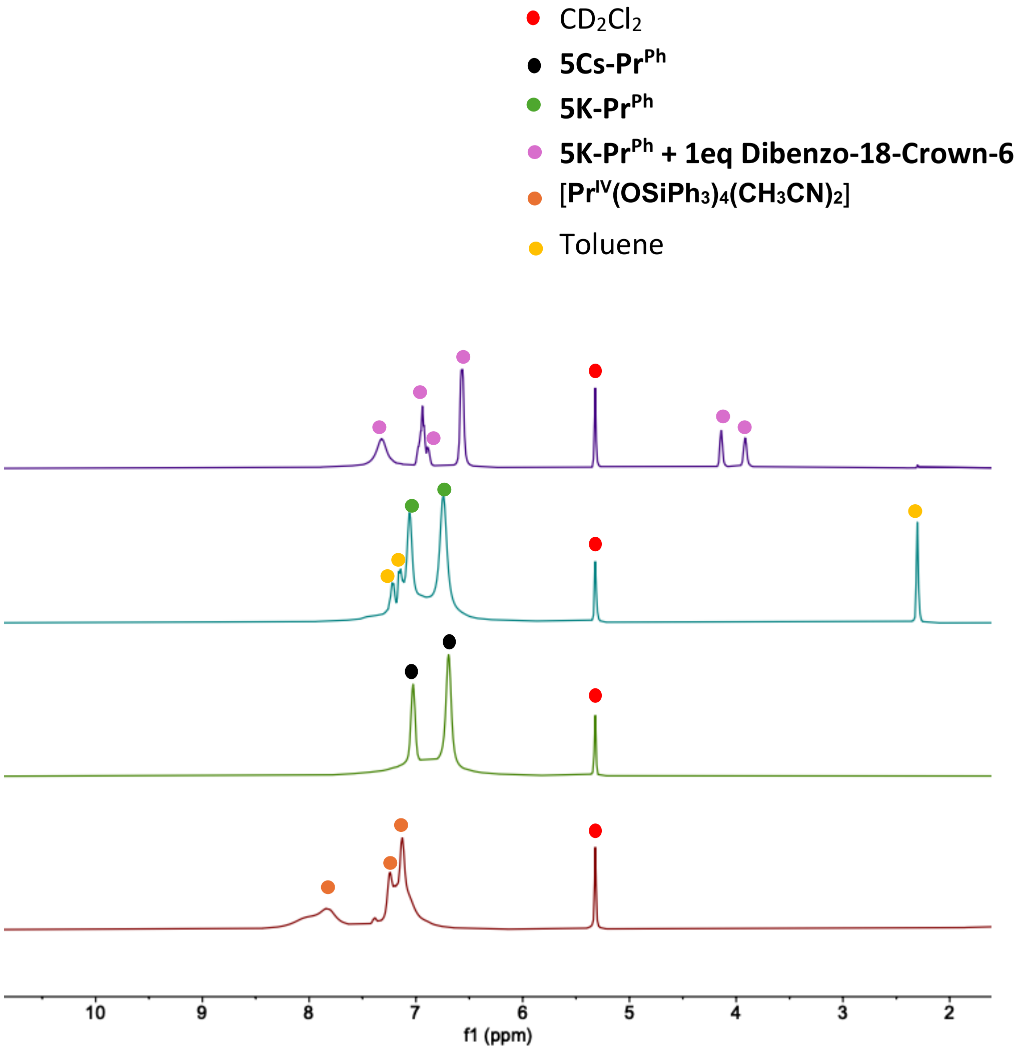

Figure S36. Comparative ^{1}H NMR (400 MHz, CD_2Cl_2) spectra of [**$Pr^{IV}(OSiPh_3)_4(CH_3CN)_2$**], **5Cs-Pr^{Ph}**, **5K-Pr^{Ph}** and **5K-Pr^{Ph}** after addition of 1 eq of Dibenzo-18-crown-6 at 233K.

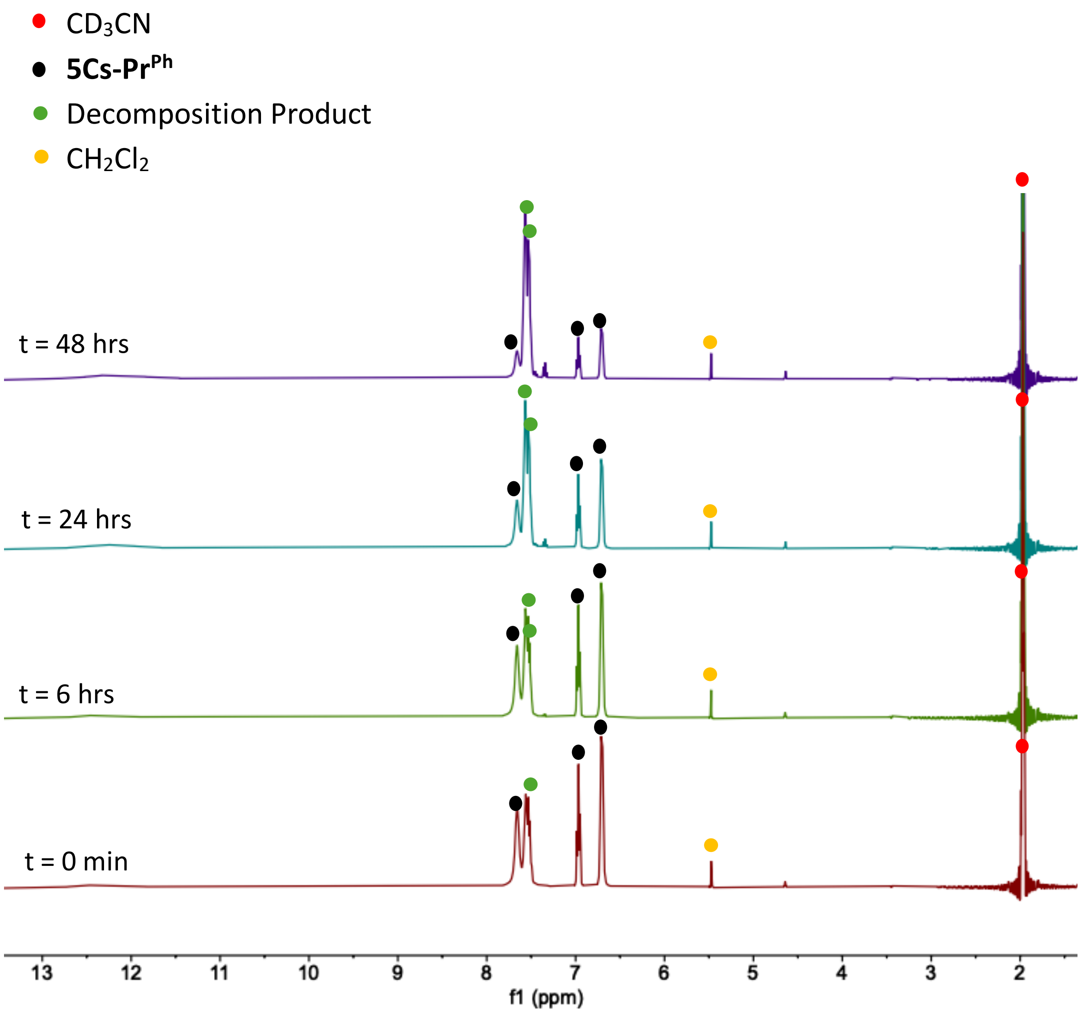

Figure S37. Time dependent ^{1}H NMR (400 MHz, CD_3CN) spectra of **5Cs-Pr^{Ph}** at 298K.

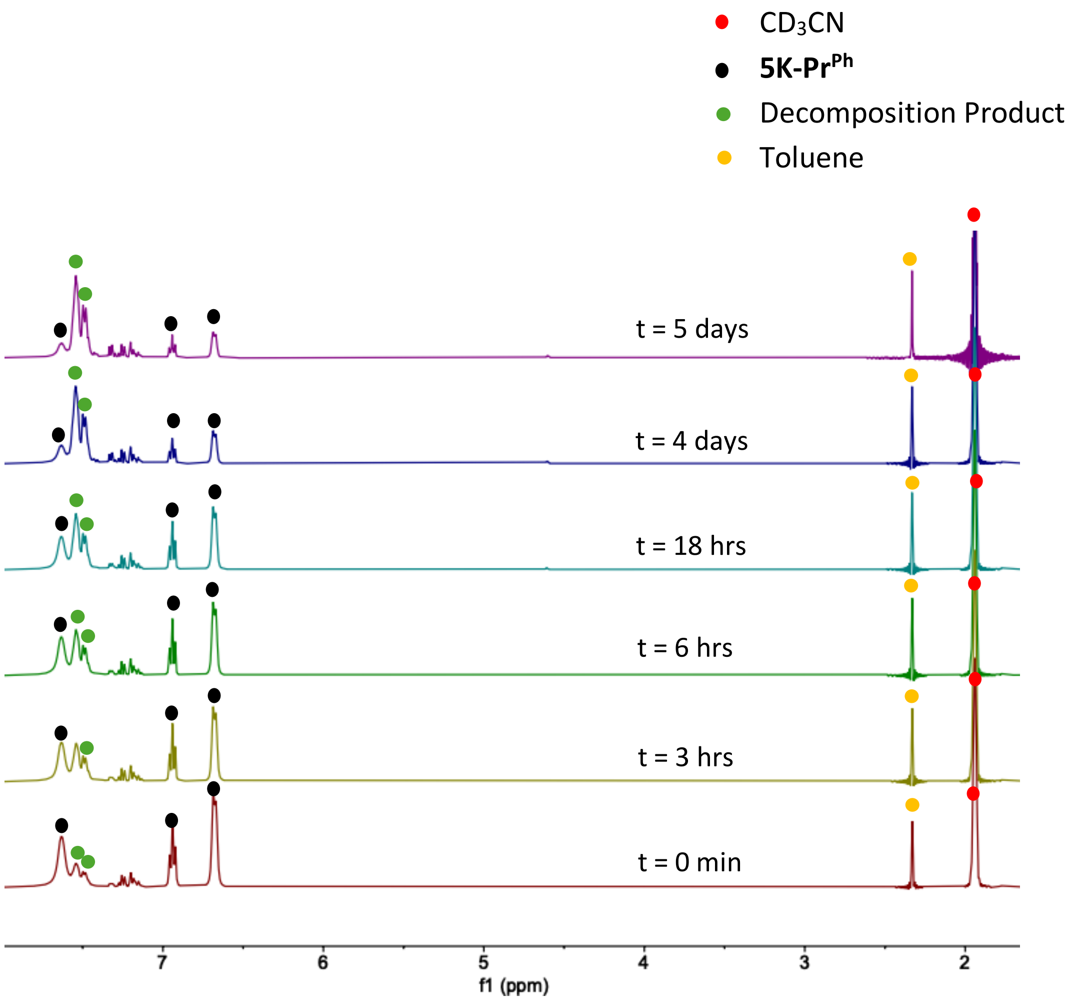

Figure S38. Time dependent ^{1}H NMR (400 MHz, CD_3CN) spectra of **5K-Pr^{Ph}** at 298K.

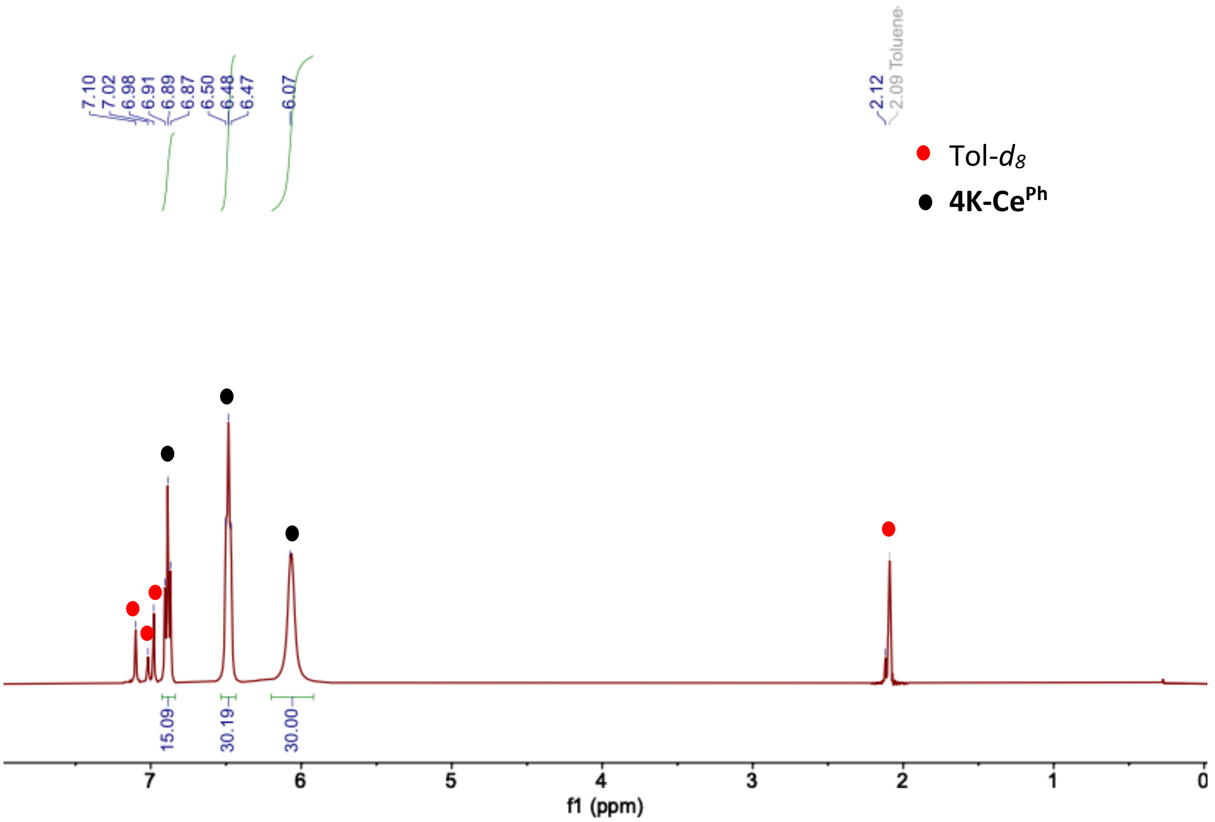

Figure S39. ^{1}H NMR spectrum (400 MHz, Tol-d_8, 298K) of **4K-Ce^{Ph}**.

4. X–Ray Data Collection and Refinements

X-ray crystallography data for the analyzed crystal structures were selected and mounted on various Rigaku diffractometers (XtaLAB Synergy R, DW system, HyPix-Arc 150 detector or SuperNova, Dual, Cu at home/near, AtlasS type detectors). The crystals were kept at a steady T = 139.99(10) K during data collection. Data were measured using ω scans with Cu K_{α} radiation. The diffraction pattern was indexed and the total number of runs and images was based on the strategy calculation from the program CrysAlisPro system (CCD 43.143a 64-bit (release 25-10-2024))[8]. The maximum resolution that was achieved was $\Theta = 72.855^{\circ}$ (0.81 Å). The unit cells were refined using CrysAlisPro 1.171.43.143a (Rigaku OD, 2024)[8]. Data reduction, scaling and absorption corrections were performed using CrysAlisPro 1.171.43.143a (Rigaku OD, 2024)[8]. A multi-scan absorption correction was performed using CrysAlisPro 1.171.43.143a (Rigaku Oxford Diffraction, 2024)[8]. The empirical absorption correction was done using spherical harmonics, implemented in SCALE3 ABSPACK scaling algorithm. The structure was solved with the ShelXT (Sheldrick, 2015)[9] solution program using dual methods and by using Olex2 1.5 (Dolomanov et al., 2009)[10] as the graphical interface. The model were refined with ShelXL 2019/3 (Sheldrick, 2015)[11] using full matrix least squares minimisation on $\boldsymbol{F}^2$. All non-hydrogen atoms were refined anisotropically. Hydrogen atom positions were calculated geometrically and refined using the riding model. CCDC numbers are 2470050-2470056 and 2471366. The Cambridge Crystallographic Data Center via www.ccdc.cam.ac.uk/data_request/cif.

Table S1. Crystallographic data and pertinent refinement parameters for **Pr** complexes.				
**Compounds**	**2-Pr^{OtBu}**	**3-Pr^{OtBu}**	**4K-Pr^{Ph}**	**5K-Pr^{Ph}**
Formula	$C_{48}H_{108}O_{16}PrSi_4$	$C_{72}H_{162}O_{24}Pr_2Si_6$	$C_{118}H_{107}K_2O_5PrSi_5$	$C_{104}H_{91}KO_5PrSi_5$
$D_{calc.}$/ g cm^{-3}	1.201	1.231	1.280	1.320
μ/mm^{-1}	6.803	8.523	5.378	5.768
Formula Weight	1194.61	1862.37	1964.59	1741.22
Colour	Clear dark orange	clear pale colourless	clear pale colourless	clear intense red
Shape	plate-shaped	prism	prism	plate
Size/mm^3	0.38×0.27×0.07	0.16×0.09×0.07	0.14×0.08×0.04	0.22×0.14×0.10
T/K	139.99(10)	139.99(10)	140.00(10)	139.99(10)
Crystal System	monoclinic	monoclinic	monoclinic	monoclinic
Space Group	$C2/c$	$C2/c$	$P2_1/n$	$P2_1$
a/Å	24.1029(3)	25.9527(5)	19.0205(3)	13.73996(12)
b/Å	13.5279(2)	14.2269(3)	23.9582(7)	24.71494(17)
c/Å	40.7549(6)	27.4839(5)	22.3860(4)	14.12364(13)
α/°	90	90	90	90
β/°	96.2414(15)	98.0362(17)	92.3363(16)	113.9745(11)
γ/°	90	90	90	90
V/Å^3	13209.8(3)	10048.1(3)	10192.8(4)	4382.36(7)
Z	8	4	4	2
Z'	1	0.5	1	1
Wavelength/Å	1.54184	1.54184	1.54184	1.54184
Radiation type	Cu K$_\alpha$	Cu K$_\alpha$	Cu K$_\alpha$	Cu K$_\alpha$
Θ_{min}/°	3.690	3.248	2.968	3.425
Θ_{max}/°	72.855	72.852	72.976	72.813
Measured Refl's.	33765	22885	52870	35525
Indep't Refl's	12735	9752	19833	16970
Refl's I≥2 σ(I)	11218	8442	13355	16786
R_{int}	0.0431	0.0373	0.0856	0.0406
Parameters	839	734	1405	1047
Restraints	1283	822	1094	1
Largest Peak	0.810	0.753	0.679	1.838
Deepest Hole	-0.517	-1.174	-0.850	-1.295
GooF	1.183	1.041	1.006	1.163
wR_2 (all data)	0.1454	0.1012	0.1401	0.1155
wR_2	0.1410	0.0966	0.1196	0.1153
R_1 (all data)	0.0770	0.0466	0.0997	0.0460
R_1	0.0684	0.0399	0.0588	0.0456

**Table S2**. Crystallographic data and pertinent refinement parameters for **Pr** complexes.				
**Compounds**	**4Cs-Pr^{Ph}**	**5Cs-Pr^{Ph}**	**4K-Ce^{Ph}**	**5[KDB18C6]-Pr^{Ph}**
Formula	$C_{104}H_{91}Cs_2O_5PrSi_5$	$C_{104}H_{91}CsO_5PrSi_5$	$C_{93.5}H_{79}CeK_2O_5Si_5$	$C_{112}H_{103}Cl_4KO_{11}PrSi_5$
$D_{calc.}$/ g cm^{-3}	1.455	1.377	1.321	1.335
μ/mm^{-1}	11.443	8.448	6.268	5.929
Formula Weight	1967.94	1835.119	1641.33	2087.20
Colour	clear pale colourless	clear intense orange	clear pale colourless	clear intense orange
Shape	irregular	prism	needle	needle
Size/mm^3	0.27×0.21×0.13	0.09×0.06×0.04	0.28×0.23×0.14	0.31×0.04×0.03
T/K	139.99(10)	140.00(10)	200.00(11)	140.00(10)
Crystal System	monoclinic	monoclinic	triclinic	monoclinic
Space Group	$P2_1$	$P2_1$	P-1	$P2_1/n$
a/Å	13.7994(3)	13.7546(18)	13.6841(9)	14.1726(2)
b/Å	24.8092(4)	24.859(3)	23.2083(15)	28.1742(7)
c/Å	14.2323(3)	14.1649(18)	27.761(2)	26.7761(4)
α/°	90	90	109.761(6)	90
β/°	112.803(3)	113.942(16)	95.331(6)	103.8097(17)
γ/°	90	90	90.676(5)	90
V/Å^3	4491.64(18)	4426.6(11)	8252.6(10)	10382.7(3)
Z	2	2	4	4
Z'	1	1	2	1
Wavelength/Å	1.54184	1.54184	1.54184	1.54184
Radiation type	Cu K$_\alpha$	Cu K$_\alpha$	Cu K$_\alpha$	Cu K$_\alpha$
Θ_{min}/°	3.369	3.41	3.056	2.312
Θ_{max}/°	72.878	66.59	72.471	75.535
Measured Refl's.	40619	37886	78686	99230
Indep't Refl's	17329	15611	31787	20180
Refl's I≥2 σ(I)	15826	9915	11191	11772
R_{int}	0.0843	0.1516	0.2397	0.1005
Parameters	1057	1097	1855	1180
Restraints	1	1363	3559	0
Largest Peak	1.737	1.4275	1.070	1.832
Deepest Hole	-2.402	-1.1123	-1.253	-1.090
GooF	1.062	0.9344	0.954	1.013
wR_2 (all data)	0.2294	0.1784	0.2740	0.2107
wR_2	0.2199	0.1436	0.1855	0.1828
R_1 (all data)	0.0938	0.1165	0.2340	0.1329
R_1	0.0865	0.0714	0.0966	0.0711

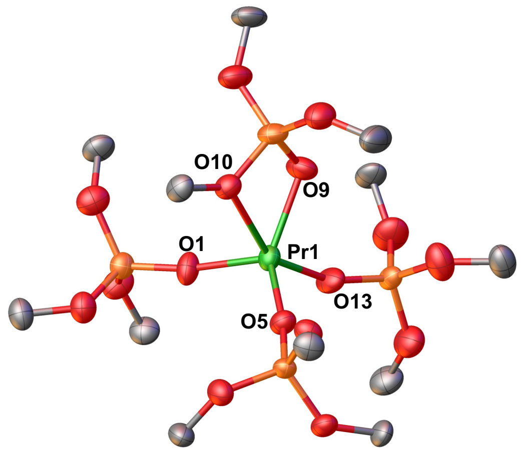

Figure S40. Molecular structure of **2-Pr^{OtBu}**, with thermal ellipsoids drawn at the 50% probability level. Hydrogen atoms on the siloxide ligands, have been omitted for clarity. Pertinent bond distances (Å) and angles (o): Pr1–O1: 2.090(4); Pr1–O5: 2.123(4); Pr1–O9: 2.147(4); Pr1–O10: 2.563(4); Pr1–O13: 2.084(4); O1–Pr1–O10: 93.40(14); O10–Pr1–O9: 60.90(15); O9–Pr1–O13: 92.80(18); O13–Pr1–O5: 101.39(17).

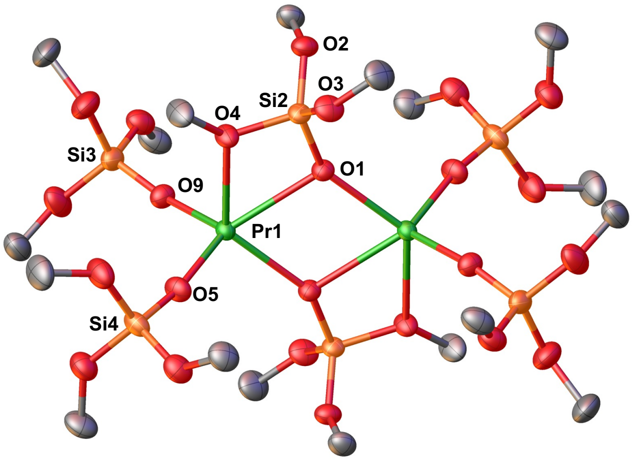

Figure S41. Molecular structure of **3-Pr^{OtBu}**, with thermal ellipsoids drawn at the 50% probability level. Hydrogen atoms on the siloxide ligands, have been omitted for clarity. Pertinent bond distances (Å) and angles (o): Pr1–O1: 2.380(2); Pr1–O5: 2.185(2); Pr1–O9: 2.169(2); O5–Pr1–O9: 110.82(10); O9–Pr1–O1: 104.15(9); O1–Pr1–O1’: 71.50(8).

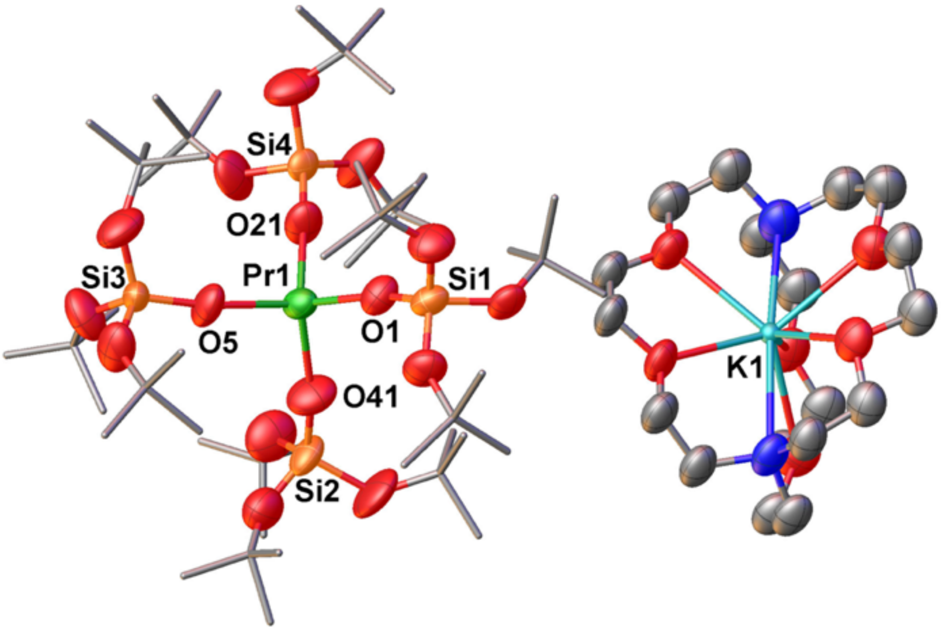

Figure S42. Molecular structure of **[K(2.2.2cryptand)][Pr^{III}(*k*$_1$-OSi(OtBu)$_3$)$_4$]** with thermal ellipsoids drawn at the 50% probability level. The ter-butoxy groups drawn as stick. Hydrogen atoms on the siloxide ligands, have been omitted for clarity. As the crystal data quality is not good only connectivity of the atoms is shown here.

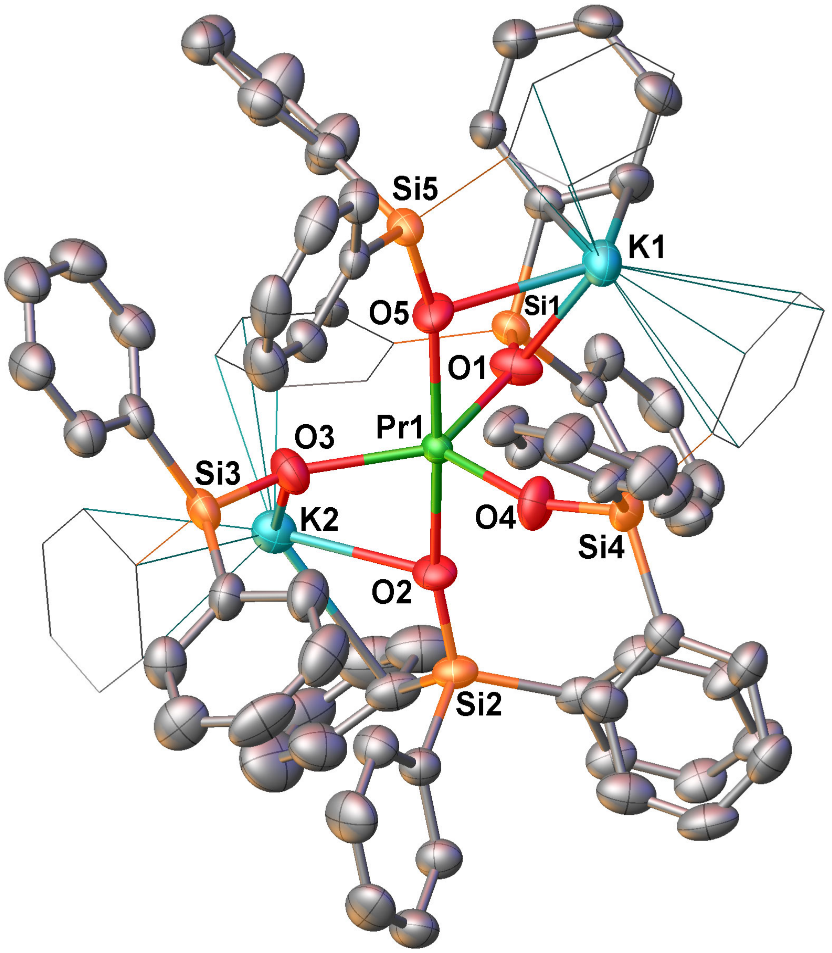

Figure S43. Molecular structure of **4K-Pr^{Ph}**, with thermal ellipsoids drawn at the 50% probability level. Hydrogen atoms on the siloxide ligands, have been omitted for clarity. Pertinent bond distances (Å) and angles (o): Pr1–O1: 2.280(4); Pr1–O2: 2.315(3); Pr1–O3: 2.261(3); Pr1–O4: 2.199(4); Pr1–O5: 2.293(3); K1–O1: 2.671(4); K1–O5: 2.818(4); K2–O2: 2.745(4); K2–O3: 2.878(4); K1–Pr1: 3.6747(11); K2–Pr1: 3.7643(13); O1–Pr1–O4: 104.86(15); O4–Pr1–O2: 97.21(16); O2–Pr1–O3: 85.09(13); O3–Pr1–O5: 93.72(13); O5–Pr1–O1: 85.48(14); O5–Pr1–O2: 172.15(16).

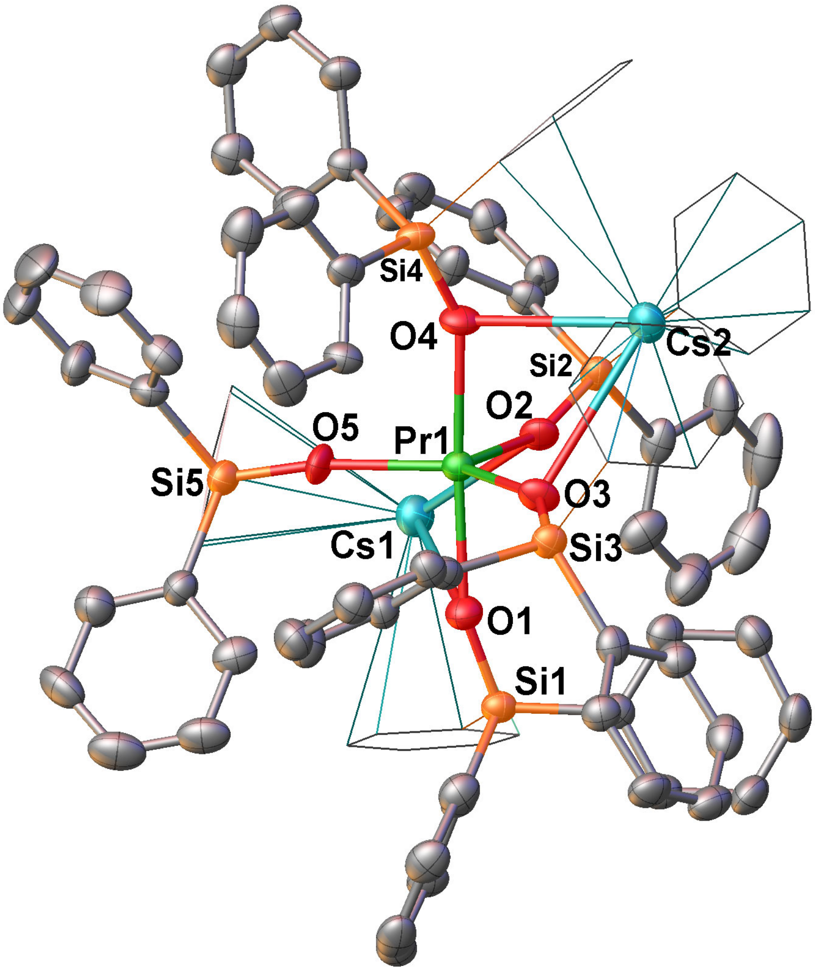

Figure S44. Molecular structure of **4Cs-Pr^{Ph}**, with thermal ellipsoids drawn at the 50% probability level. Hydrogen atoms on the siloxide ligands, have been omitted for clarity. Pertinent bond distances (Å) and angles (o): Pr1–O1: 2.300(10); Pr1–O2: 2.284(12); Pr1–O3: 2.312(11); Pr1–O4: 2.318(9); Pr1–O5: 2.279(11); Cs1–O1: 3.705(11); Cs1–O2: 3.141(13); Cs1–Pr1: 4.0735(13); Cs2–Pr1: 4.0918(13); O1–Pr1–O2: 87.7(4); O2–Pr1–O4: 92.9(4); O4–Pr1–O3: 85.0(4); O3–Pr1–O5: 138.8(5); O4–Pr1–O1: 175.7(4).

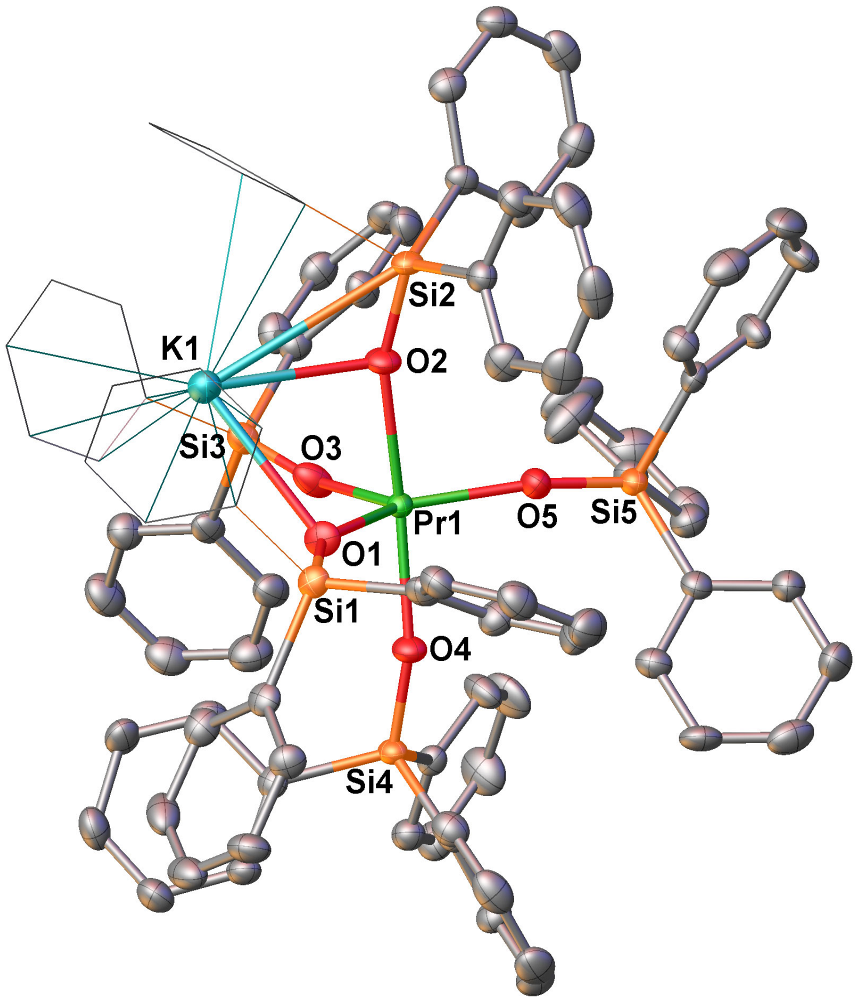

Figure S45. Molecular structure of **5K-Pr^{Ph}**, with thermal ellipsoids drawn at the 50% probability level. Hydrogen atoms on the siloxide ligands, have been omitted for clarity. Pertinent bond distances (Å) and angles (o): Pr1–O1: 2.176(5); Pr1–O2: 2.230(4); Pr1–O3: 2.098(6); Pr1–O4: 2.142(4); Pr1–O5: 2.129(5); K1–O1: 2.856(6)); K1–O2: 2.898(7); O1–Pr1–O3: 90.1(2); O3–Pr1–O2: 90.1(2); O2–Pr1–O5: 91.7(2); O5–Pr1–O4: 92.6(2), O4–Pr1–O1: 88.22(19); O2–Pr1–O4: 171.98(18).

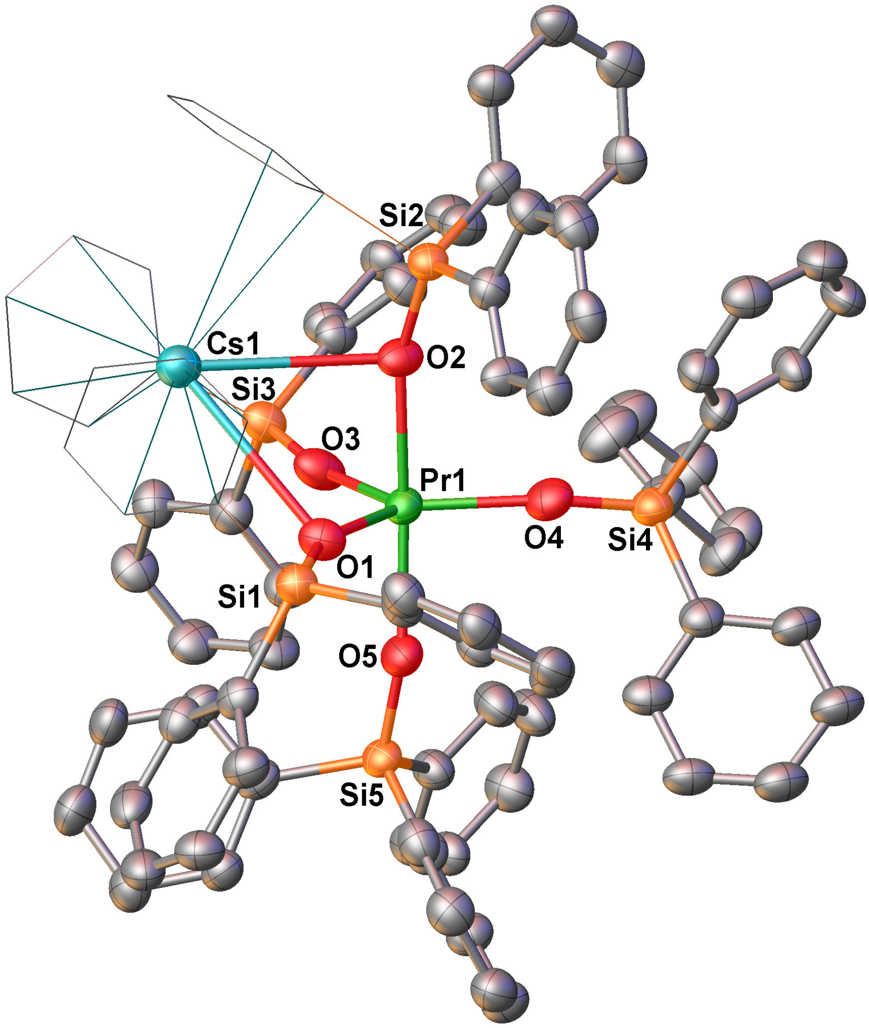

Figure S46. Molecular structure of **5Cs-Pr^{Ph}**, with thermal ellipsoids drawn at the 50% probability level. Hydrogen atoms on the siloxide ligands, have been omitted for clarity. Pertinent bond distances (Å) and angles (o): Pr1–O1: 2.182(8); Pr1–O2: 2.208(7); Pr1–O3: 2.134(8); Pr1–O4: 2.164(8); Pr1–O5: 2.173(8); Cs1–O1: 3.269(8); Cs1–O2: 3.369(8); O1–Pr1–O3: 109.7(3); O3–Pr1–O2: 90.5(3); O2–Pr1–O1: 86.6(3); O4–Pr1–O5: 92.7(3); O2–Pr1–O5: 173.4(3).

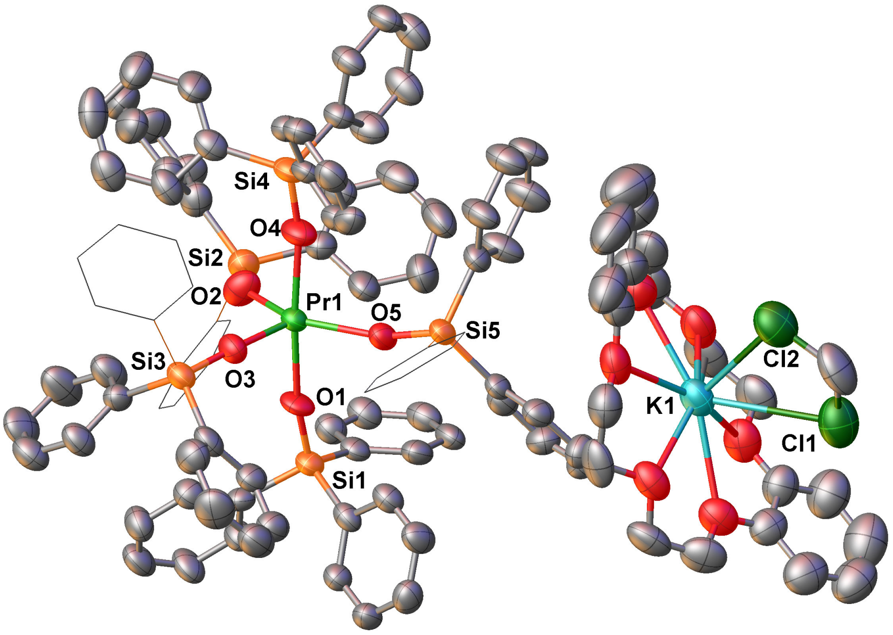

Figure S47. Molecular structure of **5[KDB18C6]-Pr^{Ph}**, with thermal ellipsoids drawn at the 50% probability level. Hydrogen atoms on the siloxide ligands, have been omitted for clarity. Pertinent bond distances (Å) and angles (o): Pr1–O1: 2.173(4); Pr1–O2: 2.136(5); Pr1–O3: 2.109(4); Pr1–O4: 2.164(4); Pr1–O5: 2.163(4); K1–Cl1: 3.409(3); K1–Cl2: 3.261; O1–Pr1–O4: 172.92(2); O1–Pr1–O5: 87.13(2); O5–Pr1–O4: 90.34(2); O4–Pr1–O2: 90.1(2); O2–Pr1–O3: 103.84(2); O3–Pr1–O1: 93.13(2).

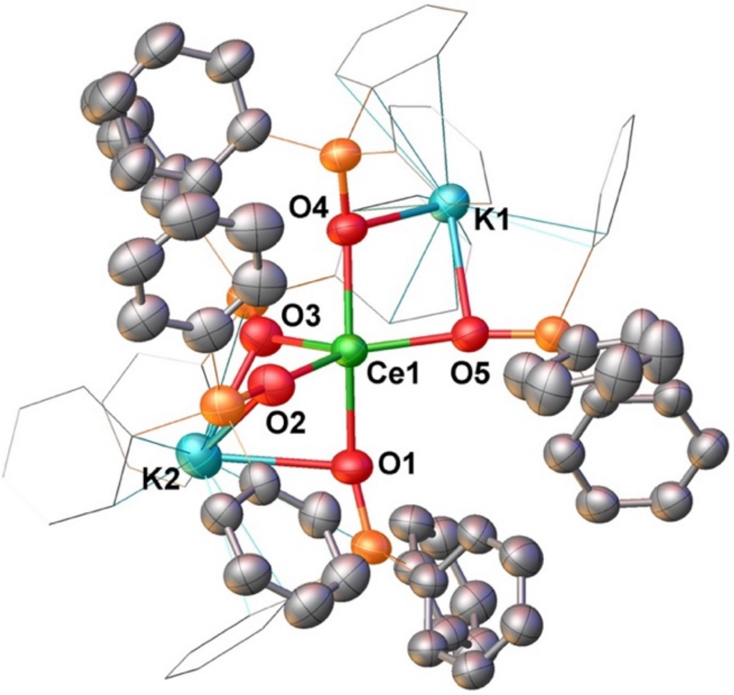

Figure S48. Molecular structure of [$K_2Ce(OSiPh)_3)_5$], with thermal ellipsoids drawn at the 50% probability level. Phenyl groups bound to the potassium ions are drawn as wireframe. Hydrogen atoms on the siloxide ligands, have been omitted for clarity. Pertinent bond distances (Å) and angles (o): Ce1–O1: 2.363(10); Ce1–O2: 2.268(10); Ce1–O3: 2.305(9); Ce1–O4: 2.335(9); Ce1–O5: 2.343(9); K1–O4: 2.781(10); K1–O5: 2.870(9); K2–O1: 2.926(10); K2–O2: 3.020(12); O1–Ce1–O2: 88.8(4); O2–Ce1–O3: 91.4(4); O3–Ce1–O4: 87.5(3); O4–Ce1–O5: 84.2(3); O5–Ce1–O1: 93.7(3); O1–Ce1–O4: 167.1(3).

5. UV-Vis Spectra

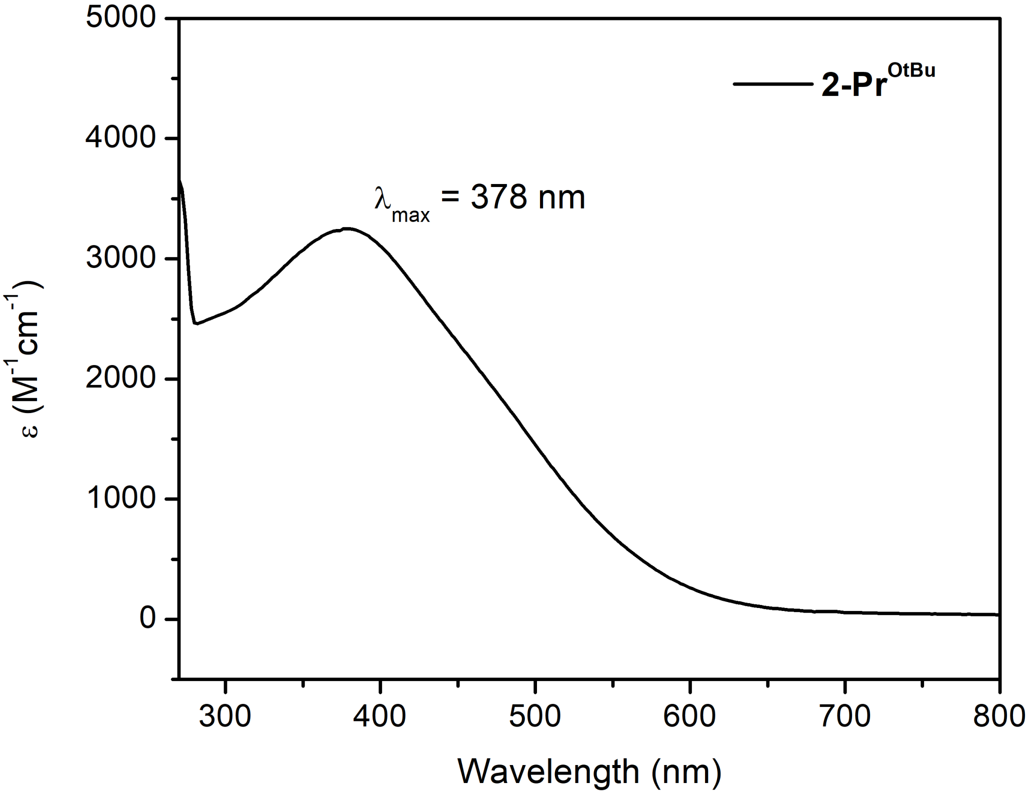

Figure S49 UV-vis electronic absorption spectrum of praseodymium complex **2-Pr^{OtBu}** (1.0 mM) in toluene at room temperature.

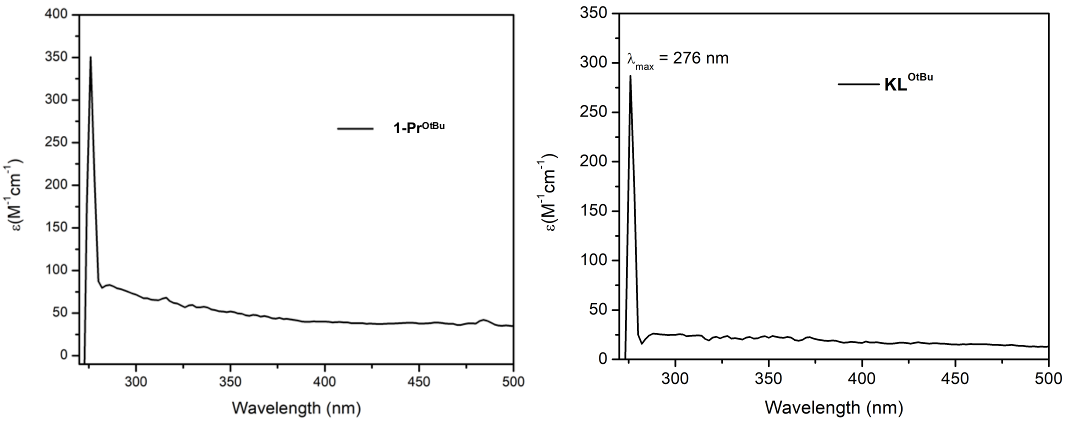

Figure S50. UV-vis electronic absorption spectra of praseodymium(III) complex **1-Pr^{OtBu}** (left) and **KL^{OtBu}** (right) (1.0 mM) in toluene at room temperature.

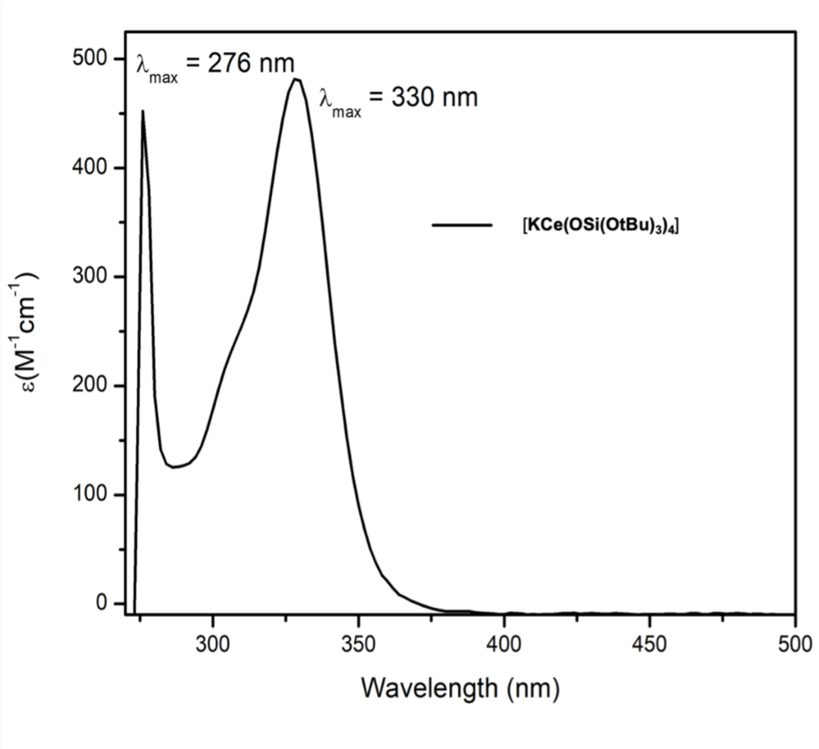

Figure S51. UV-vis electronic absorption spectrum of [**KCe(OSi(OtBu)$_3$)$_4$**] (1.0 mM) in toluene at room temperature.

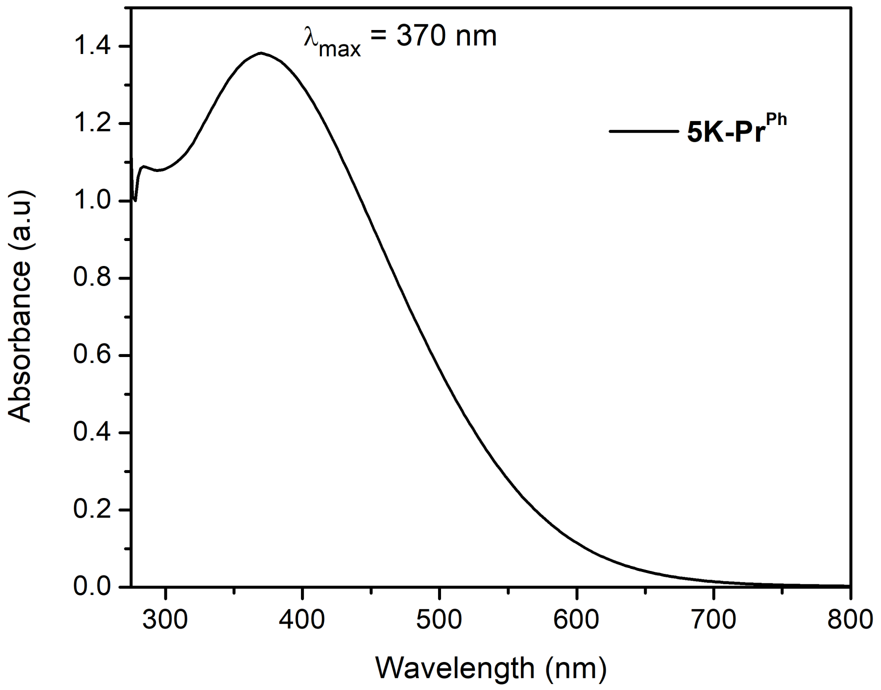

Figure S52. UV-Visible spectrum of **5K-Pr^{Ph}** in toluene (3 mM) at room temperature.

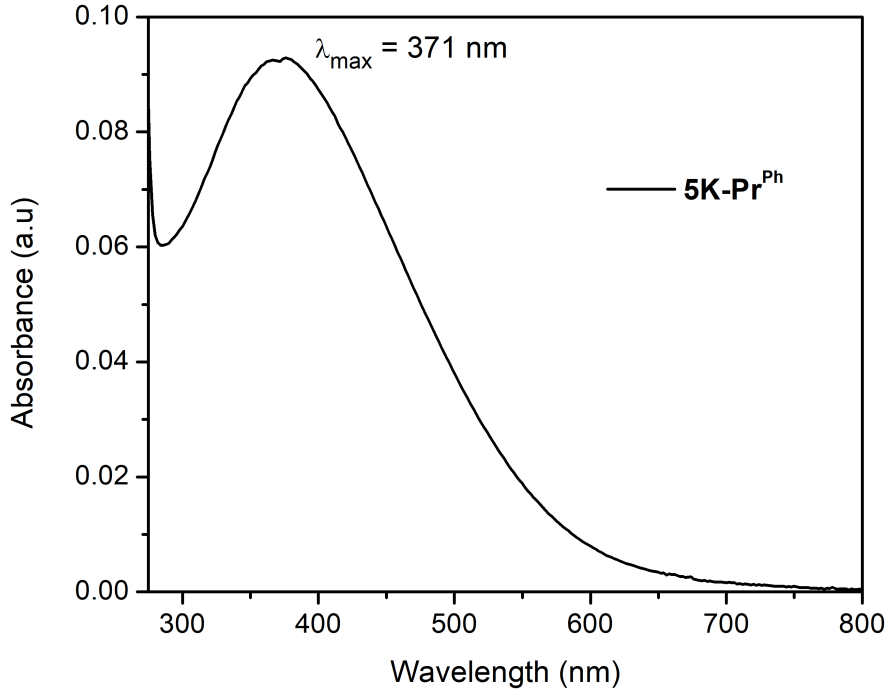

Figure S53. UV-Visible spectrum of **5K-Pr^{Ph}** in DCM (0.15 mM) at room temperature.

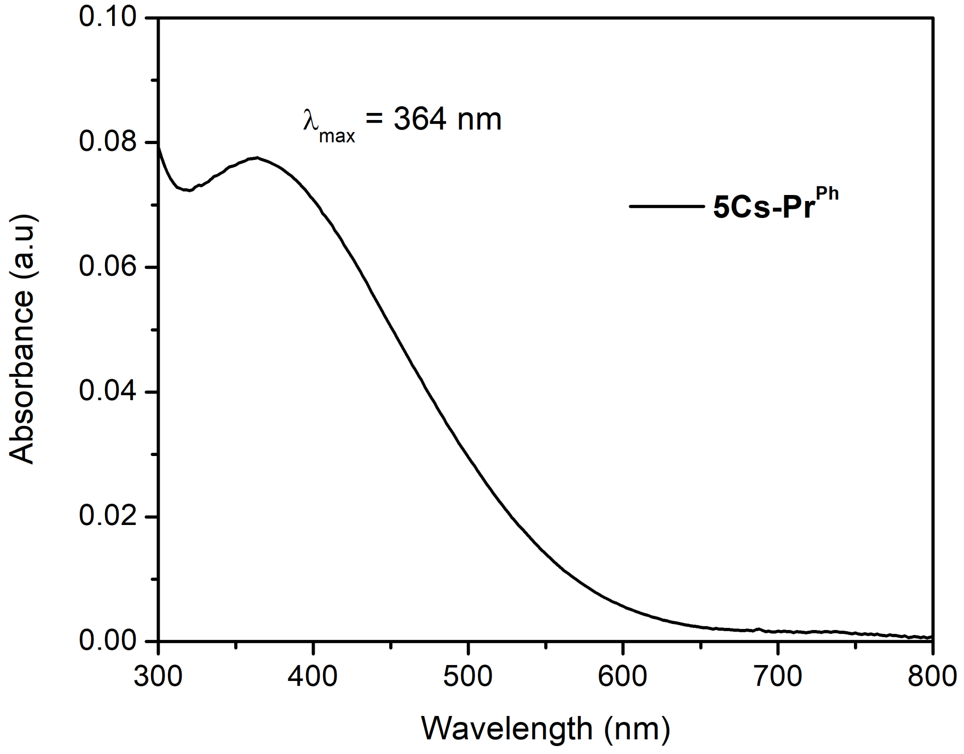

Figure S54. UV-Visible spectrum of **5Cs-Pr^{Ph}** in DCM (0.15 mM) at room temperature.

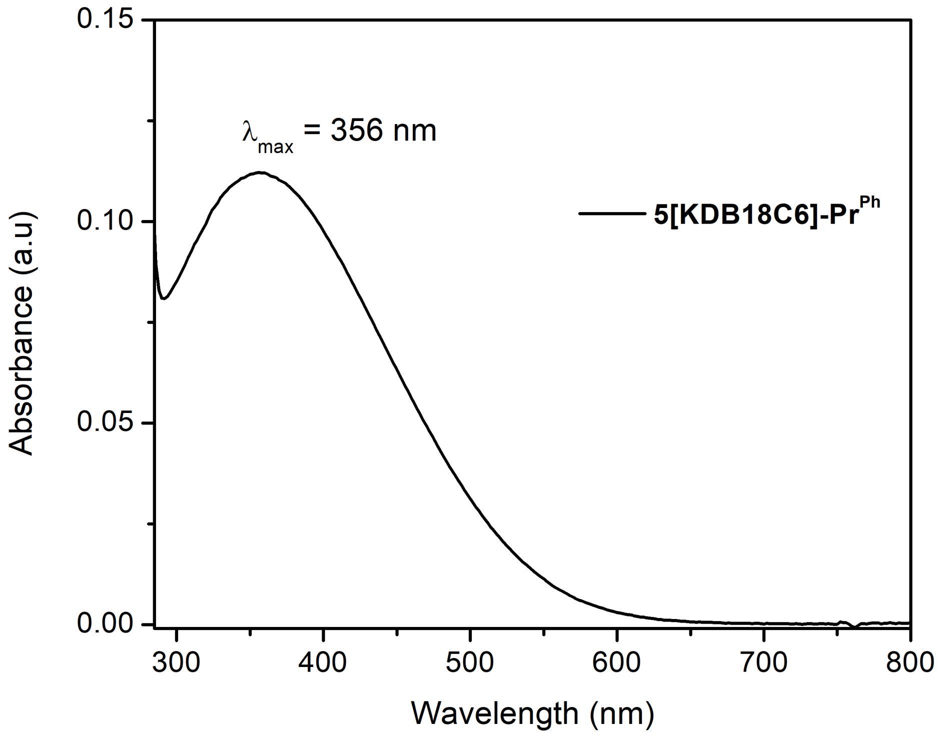

Figure S55. UV-Visible spectrum of **5[KDB18C6]-Pr^{Ph}** in DCM (0.15 mM) at room temperature.

6. Electrochemistry

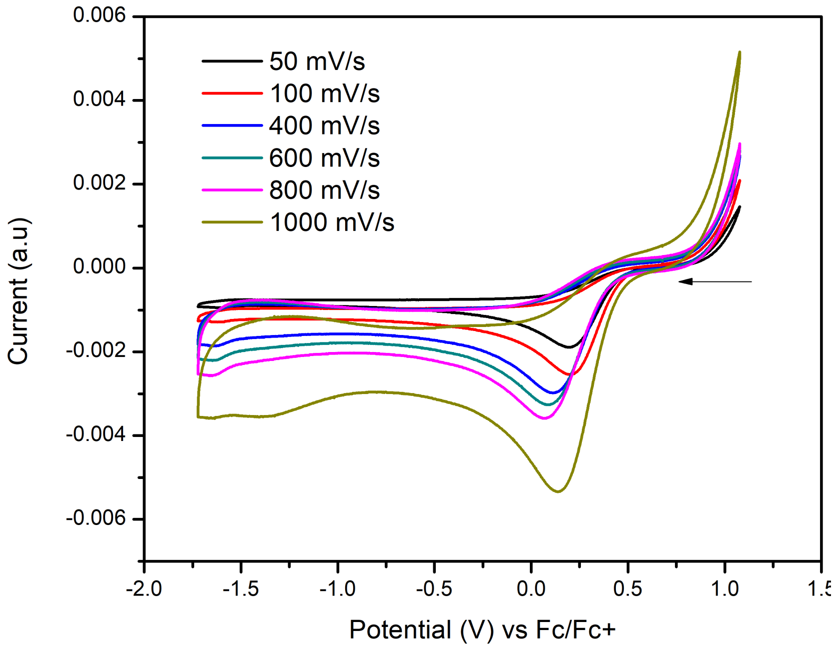

Figure S56. Scan rate dependent cyclic voltammogram for **2-Pr^{OtBu}** in THF.

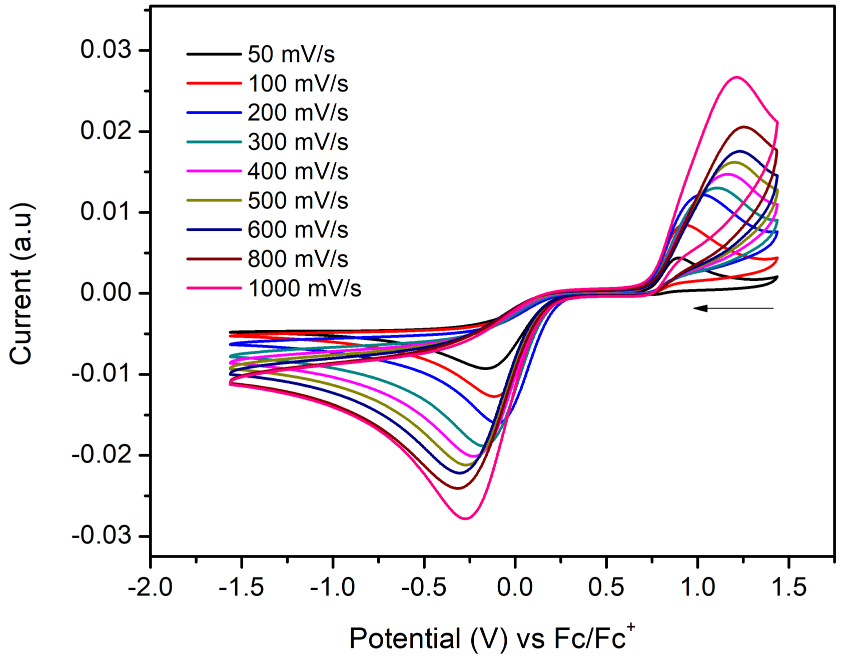

Figure S57. Scan rate dependent cyclic voltammogram for **2-Pr^{OtBu}** in DCM

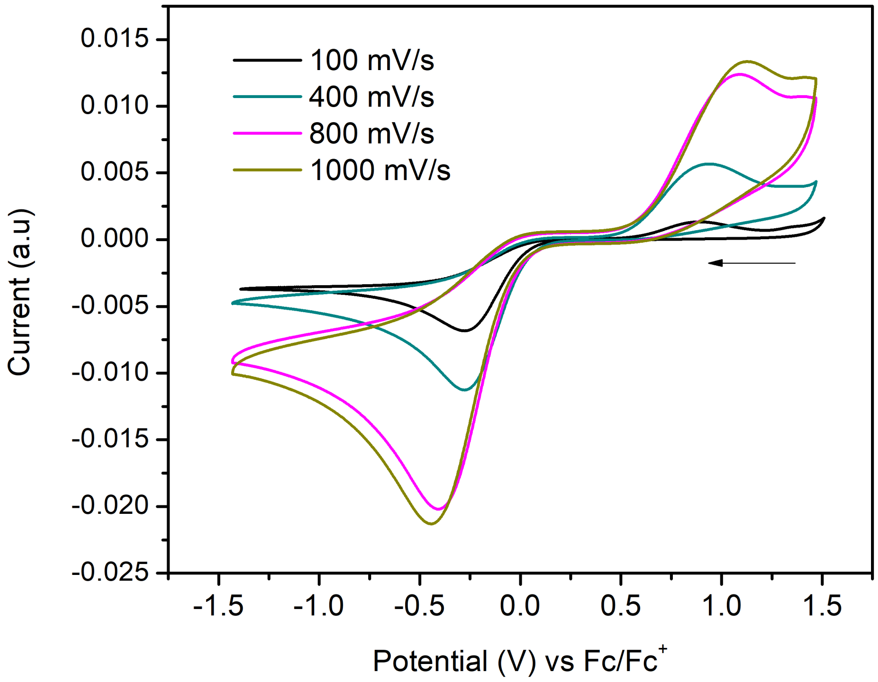

Figure S58. Scan rate dependent cyclic voltammogram for **[$Tb^{IV}(OSi(OtBu)_3)_4$]** in DCM.

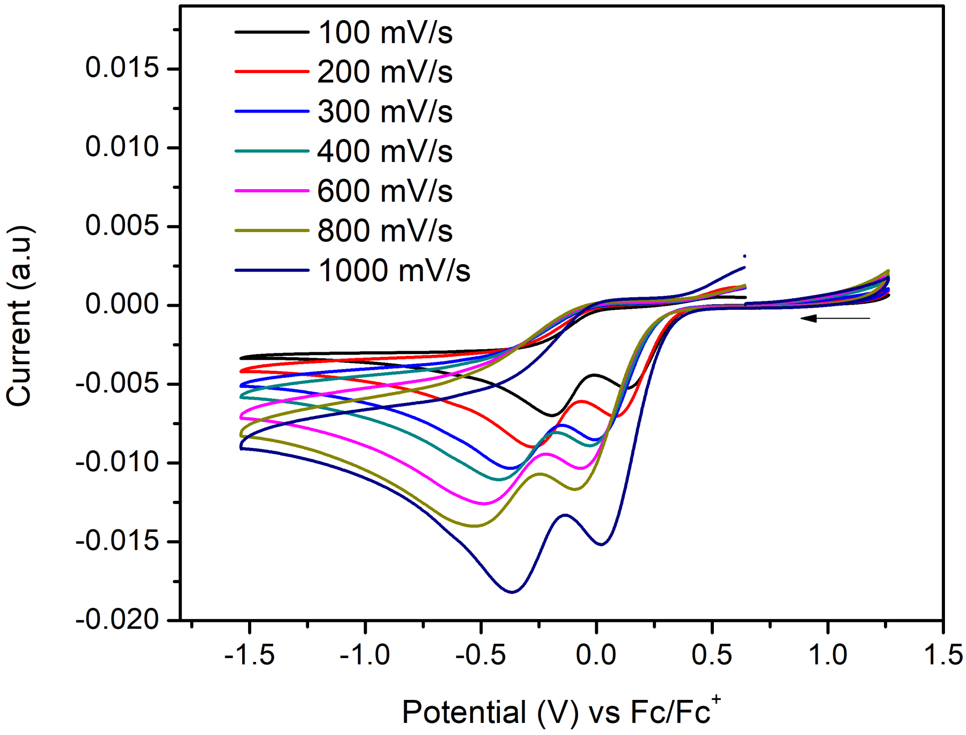

Figure S59. Scan rate dependent cyclic voltammogram for **5K-Pr^{Ph}** in DCM.

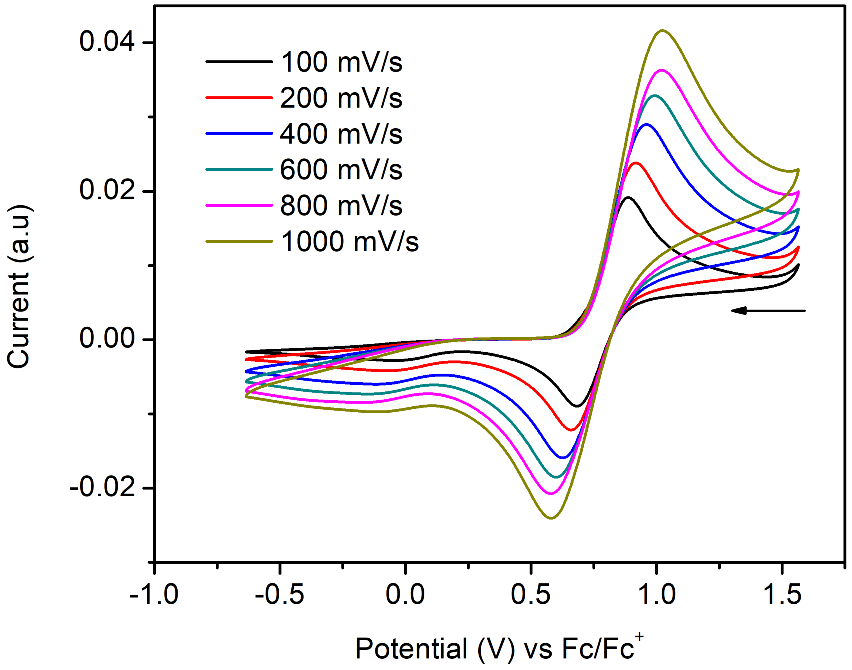

Figure S60. Scan rate dependent cyclic voltammogram for **5Cs-Pr^{Ph}** in DCM

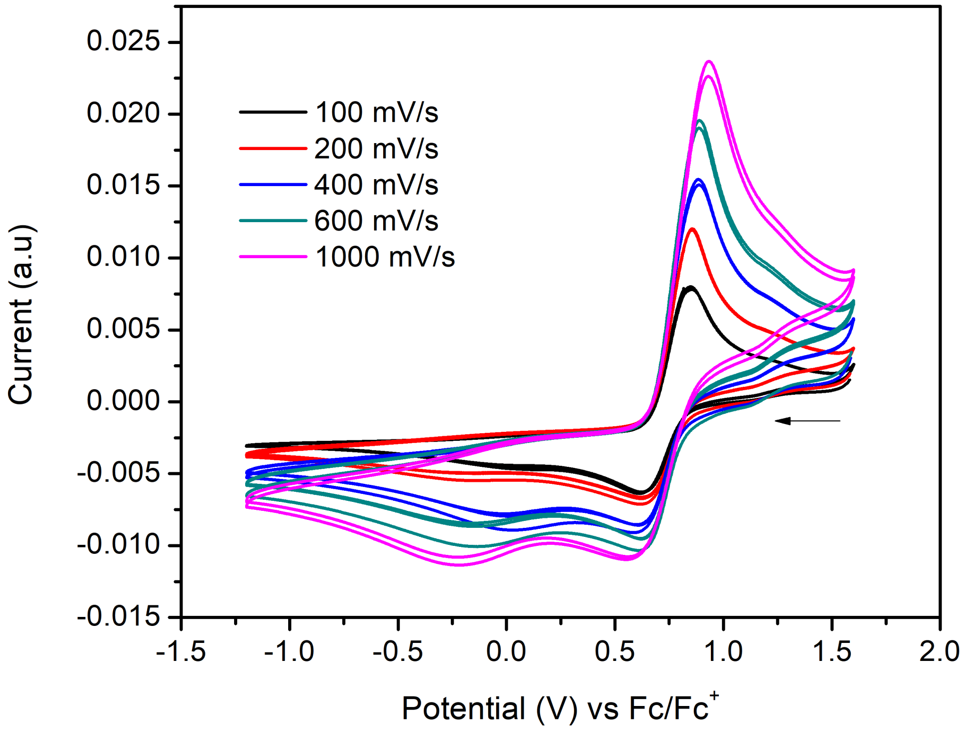

Figure S61. Scan rate dependent cyclic voltammogram for **[$Pr^{IV}(OSiPh_3)_4(CH_3CN)_2$]** in DCM.

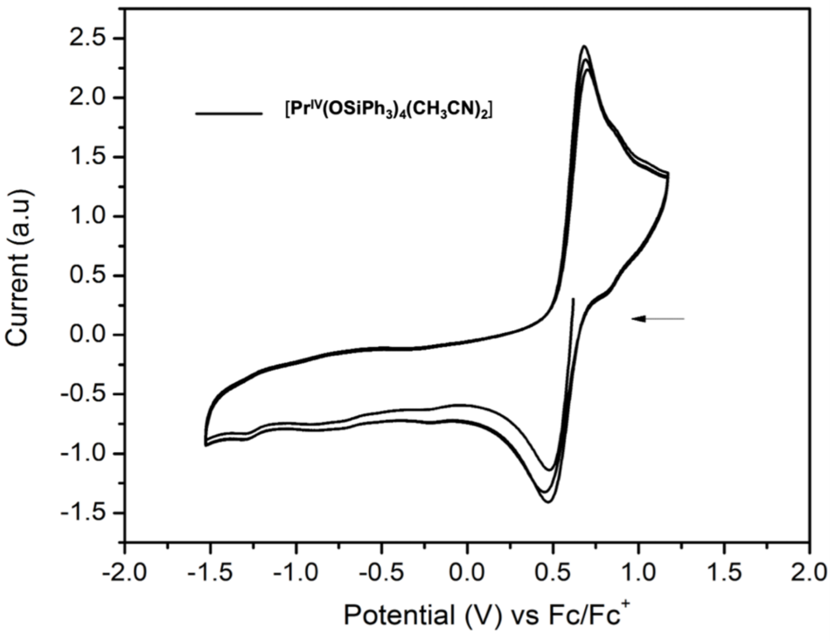

Figure S62. Cyclic voltammogram for **[$Pr^{IV}(OSiPh_3)_4(CH_3CN)_2$]** in CH_3CN at 100 mV/s.

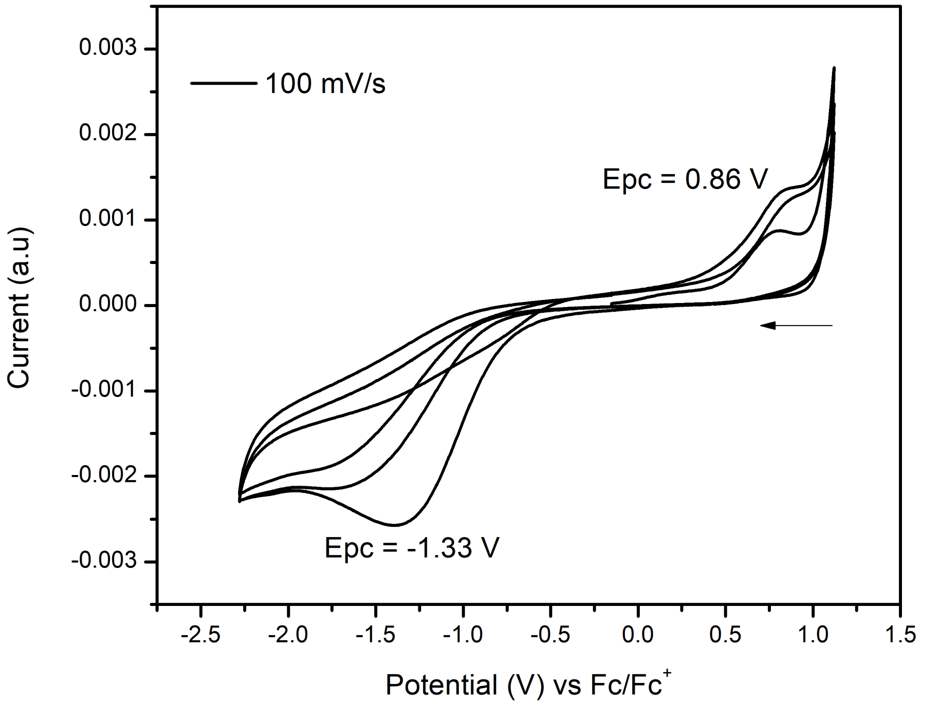

Figure S63. Cyclic voltammogram for **5[KDB18C6]-Pr^{Ph}** in DCM at 100 mV/s with 3 scan cycles. After 1st scan cycle disappearance of reduction peak observed.

7. EPR

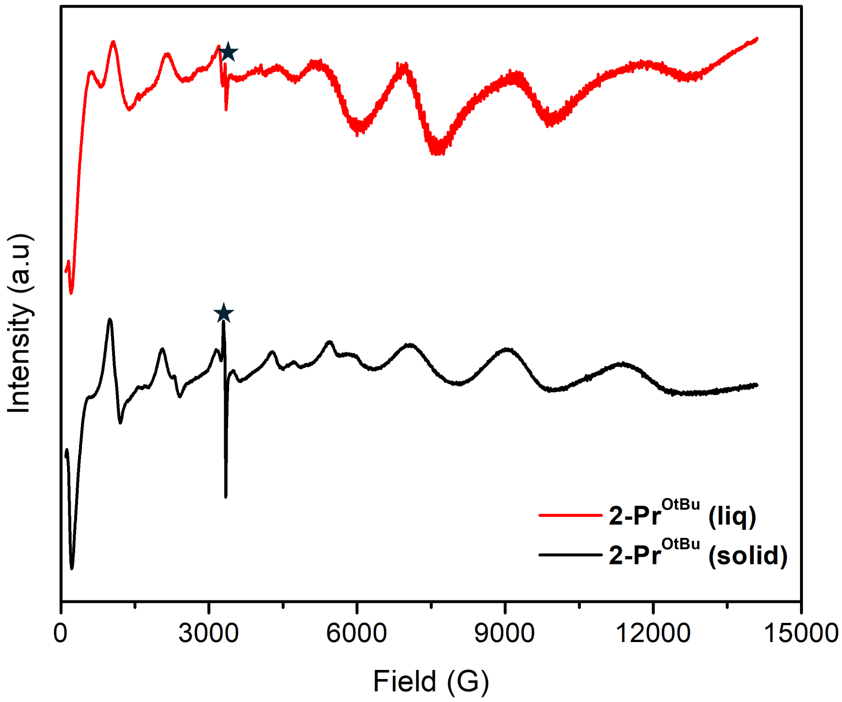

Figure S64. X-band (9.4 GHz) EPR spectrum of complex **2-Pr^{OtBu}** in solid (black) and solution state (10 mM in *n*-hexane) (red) at 6K; (* Denotes the free radical impurity due to oxidant).

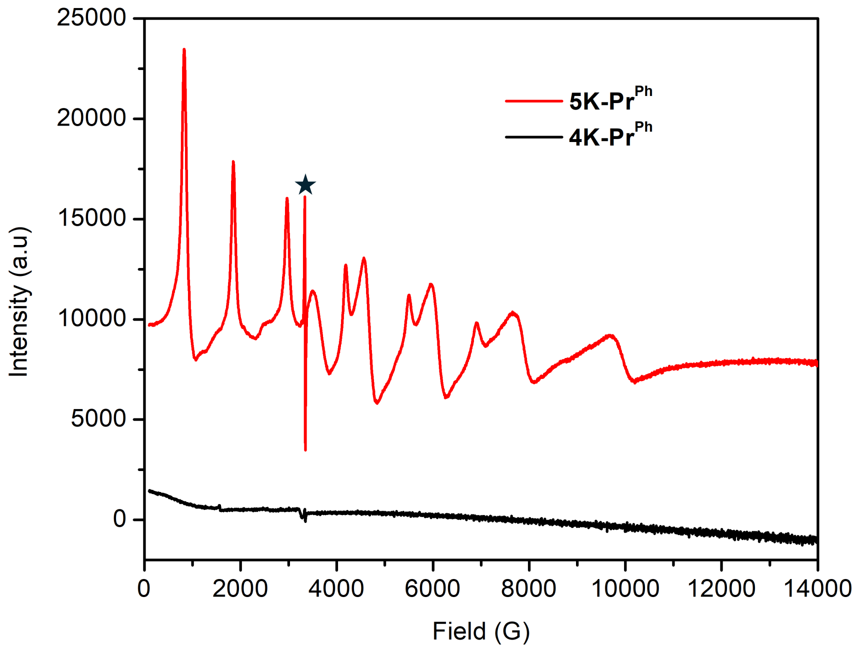

Figure S65. X-band (9.4 GHz) EPR spectrum of complex **4K-Pr^{Ph}** and **5K-Pr^{Ph}** in solid state at 6K; (* Denotes the free radical impurity due to oxidant).

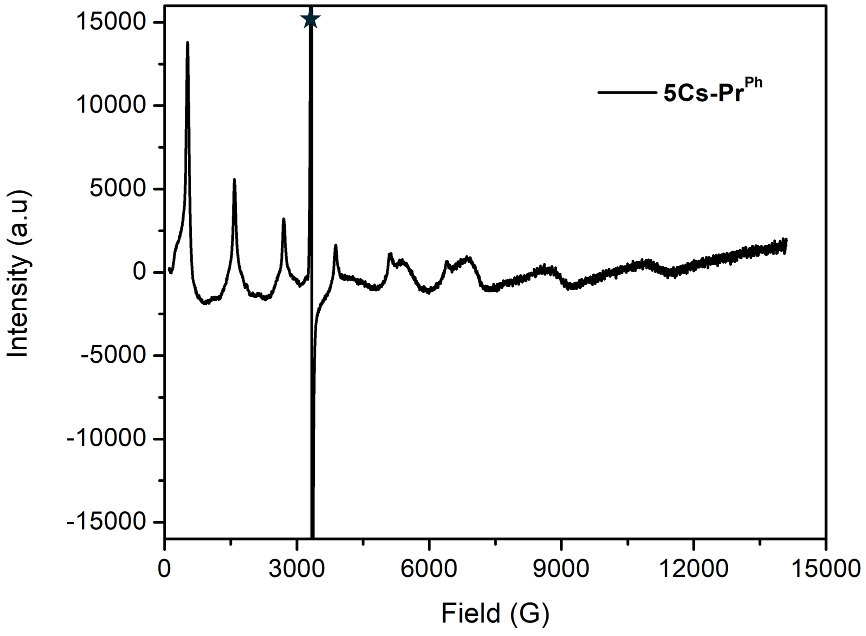

Figure S66. X-band (9.4 GHz) EPR spectrum of complex **5Cs-Pr^{Ph}** in solid state at 6K; (* Denotes the free radical impurity due to oxidant).

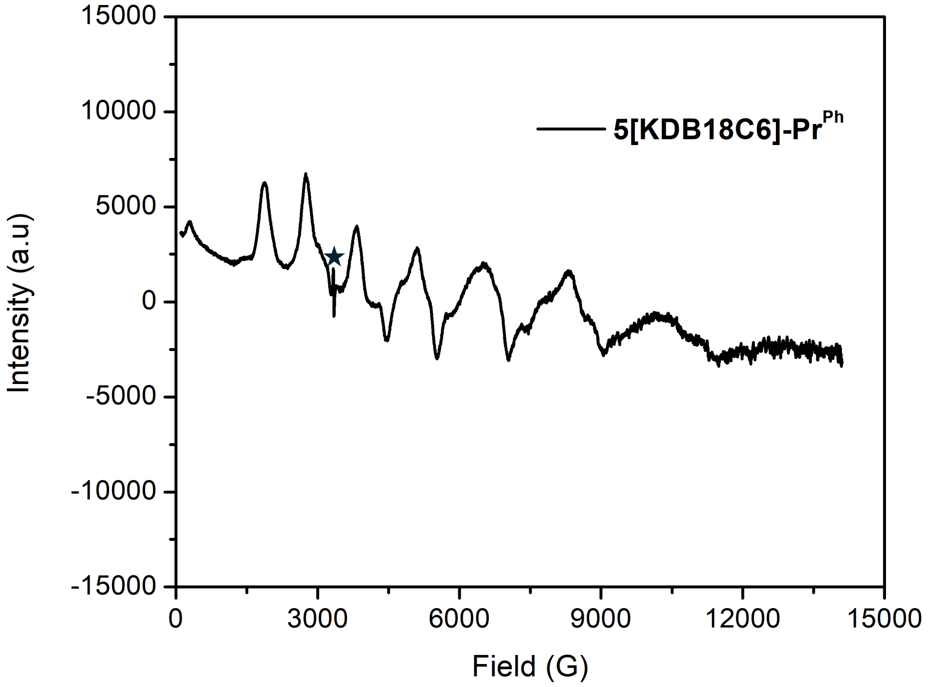

Figure S67. X-band (9.4 GHz) EPR spectrum of complex **5[KDB18C6]-Pr^{Ph}** in solid state at 6K; (* Denotes the free radical impurity due to oxidant).

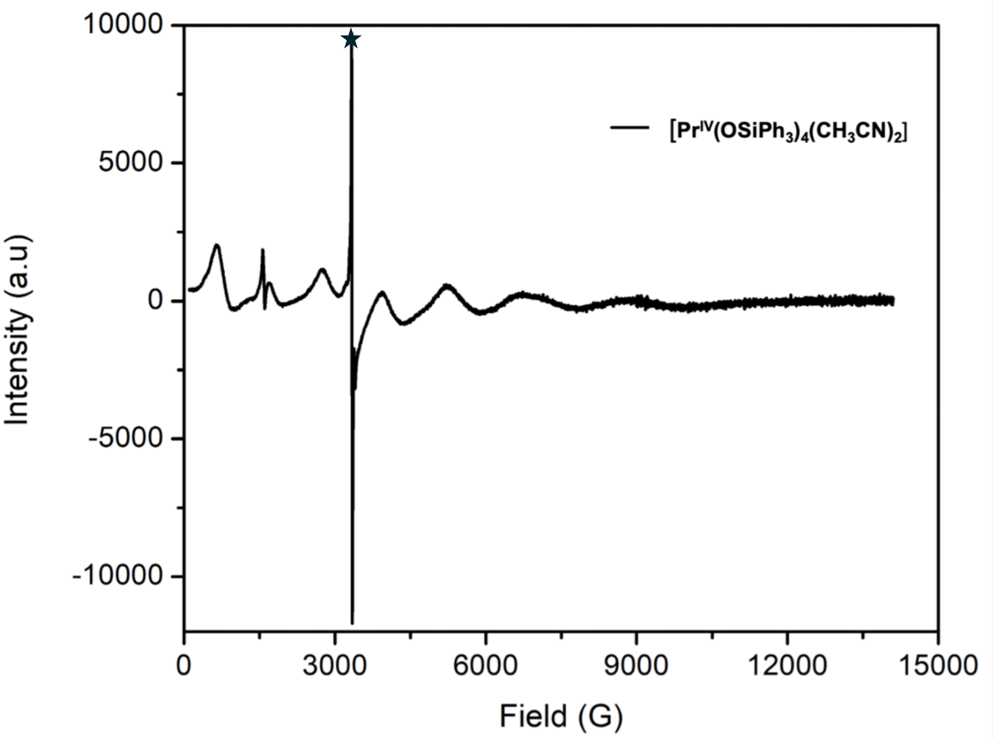

Figure S68. X-band (9.4 GHz) EPR spectrum of complex **[$Pr^{IV}(OSiPh_3)_4(CH_3CN)_2$]** in solution state (20 mM in CH_3CN) at 6K; (* denotes the free radical impurity due to oxidant).

8. Magnetism

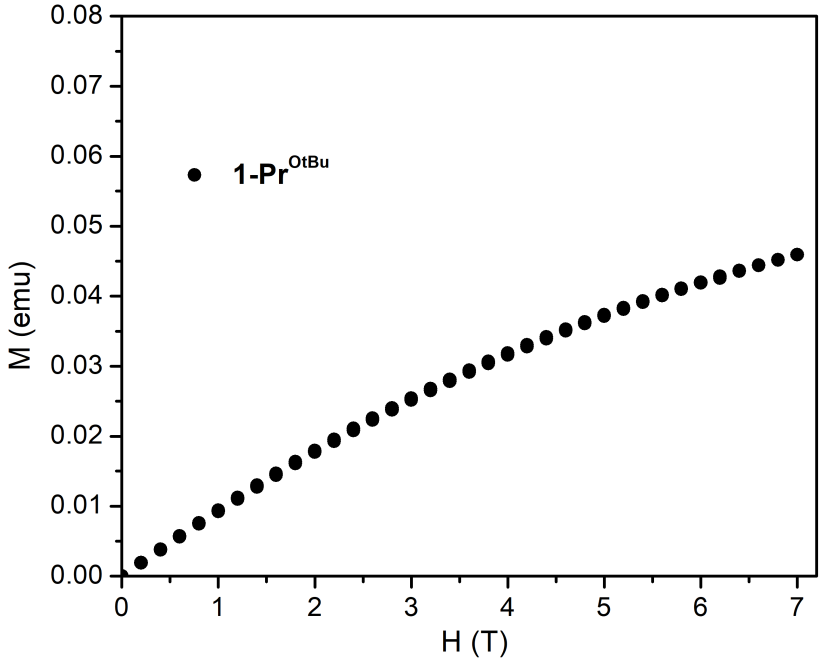

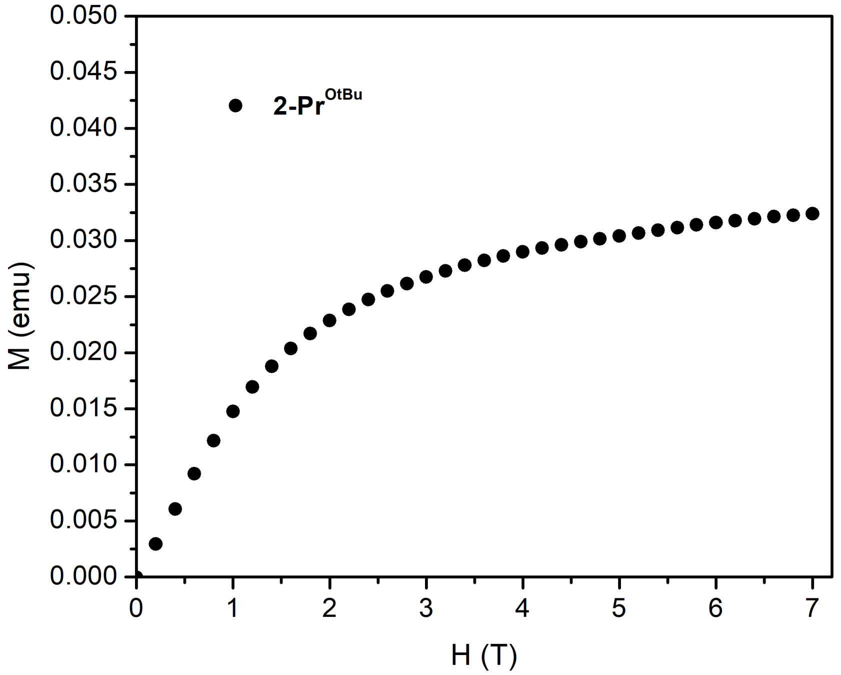

Figure S69. Magnetization data for **1-Pr^{OtBu}** (top) and **2-Pr^{OtBu}** (bottom) at 2 K from 0 to 7 T.

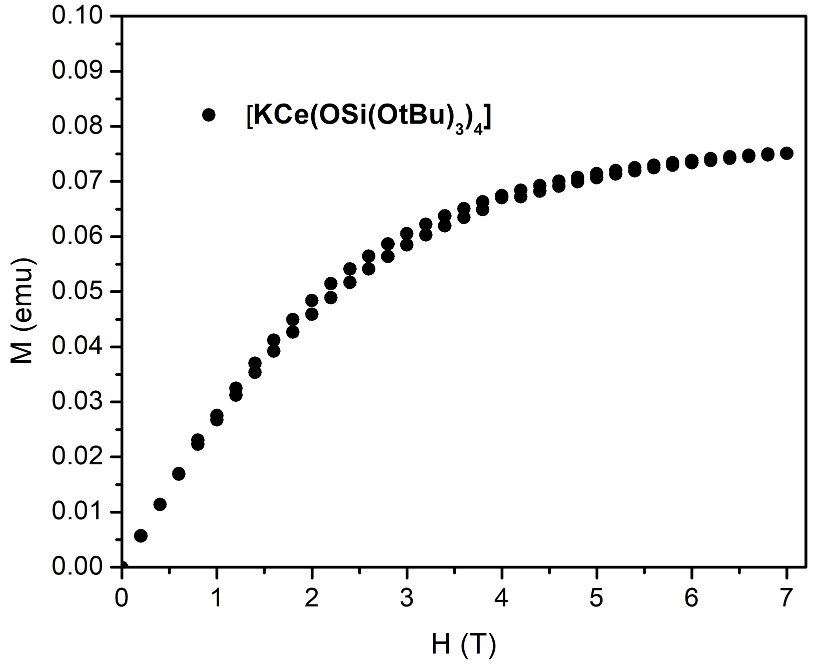

Figure S70. Magnetization data for [**KCe(OSi(OtBu)$_3$)$_4$**] at 2 K from 0 to 7 T.

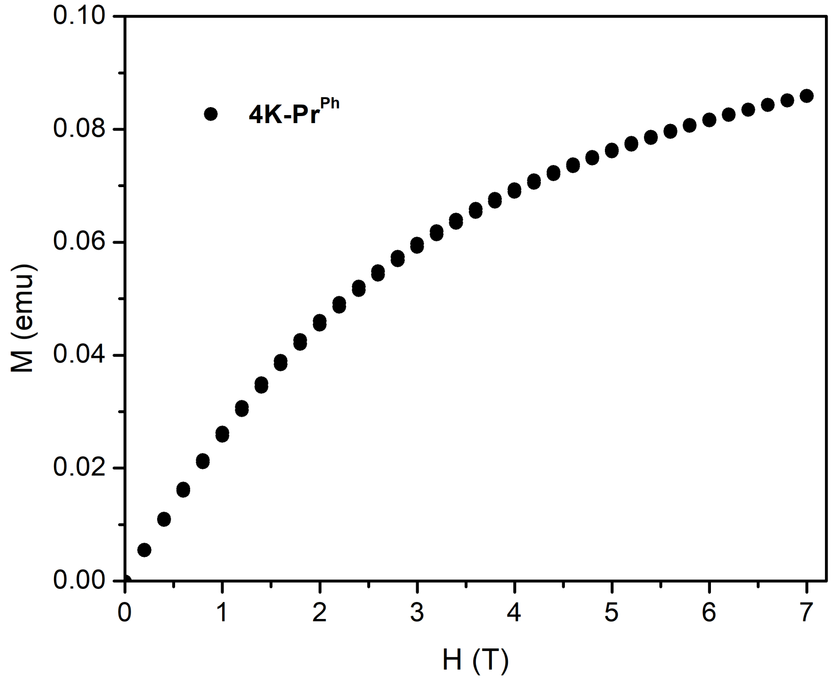

Figure S71. Magnetization data for **4K-Pr^{Ph}** at 2 K from 0 to 7 T.

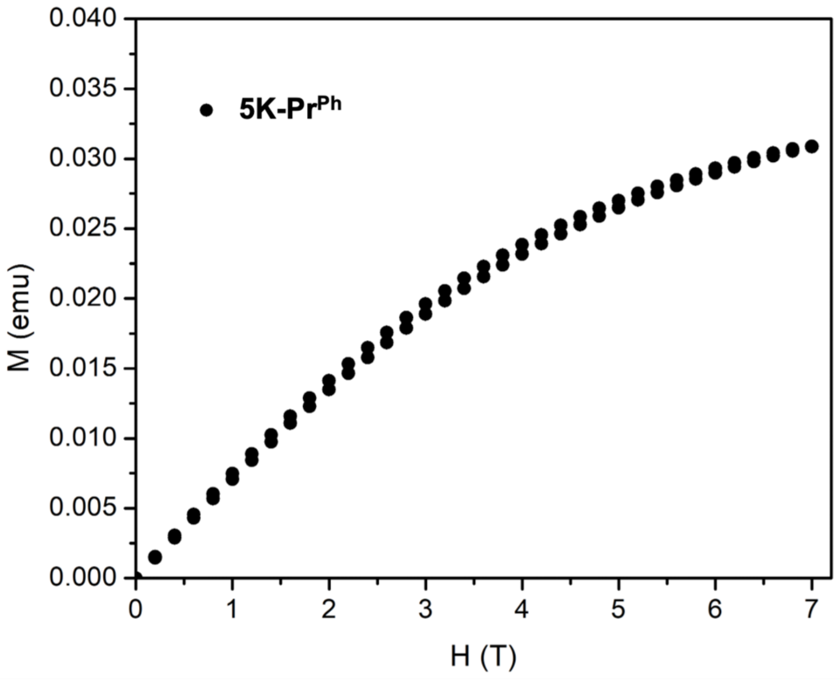

Figure S72. Magnetization data for **5K-Pr^{Ph}** at 2 K from 0 to 7 T.

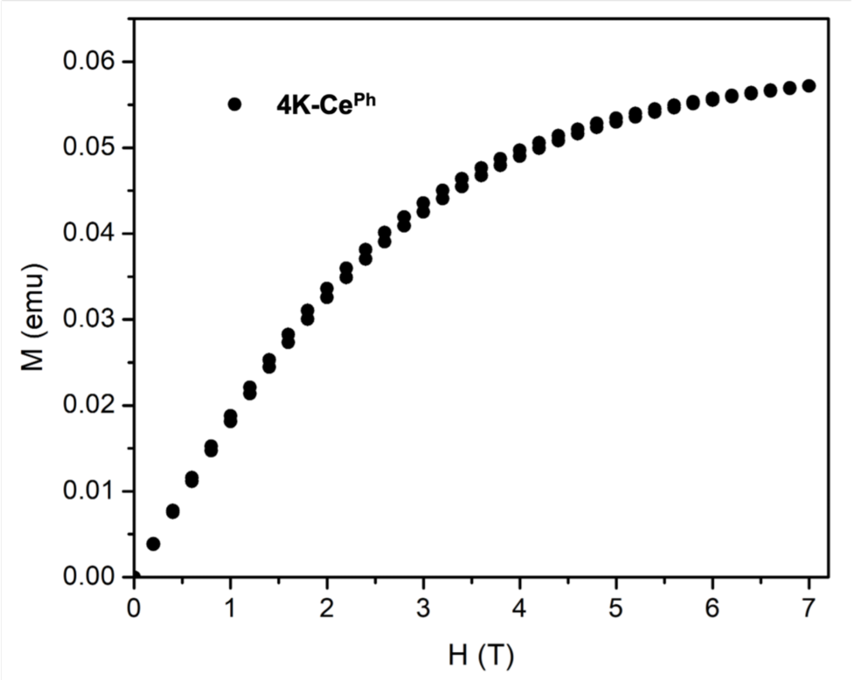

Figure S73. Magnetization data for **4K-Ce^{Ph}** at 2 K from 0 to 7 T.

8. Computational Details.

The optimization of Cerium and Praseodymium complexes was carried out by employing DFT hybrid functional (B3PW91)[12] along with small core pseudopotential Stuggart basis set[13] for cerium, potassium, silicon and cesium atoms with additional polarization functions[14] for potassium, silicon and cesium atoms. Pople basis sets[15] (6-31G**) were employed for the rest of the atoms. Disperison corrections were included in our calculations by employing D3 version of Grimme's dispersion with Becke-Johnson damping.[16] Frequency calculations were performed to locate minima for the optimized structures. All the calculations were performed using Gaussian 09 suite of programs.[17] Natural bond order analysis carried out by employing NBO 6.0 implemented in gaussian program.[18]

Table S3. Selected structural parameters comparison between DFT optimized and X-ray structures for $Ce[OSi(O^tBu)_3]_4$ complex (s=0)

Atom labels	DFT		X-ray
	No dispersion	dispersion	
Ce1-O6	2.11	2.11	2.08
Ce1-O10	2.16	2.16	2.16
Ce1-O13	2.64	2.55	2.58
Ce1-O14	2.12	2.10	2.10
Ce1-O18	2.12	2.10	2.09

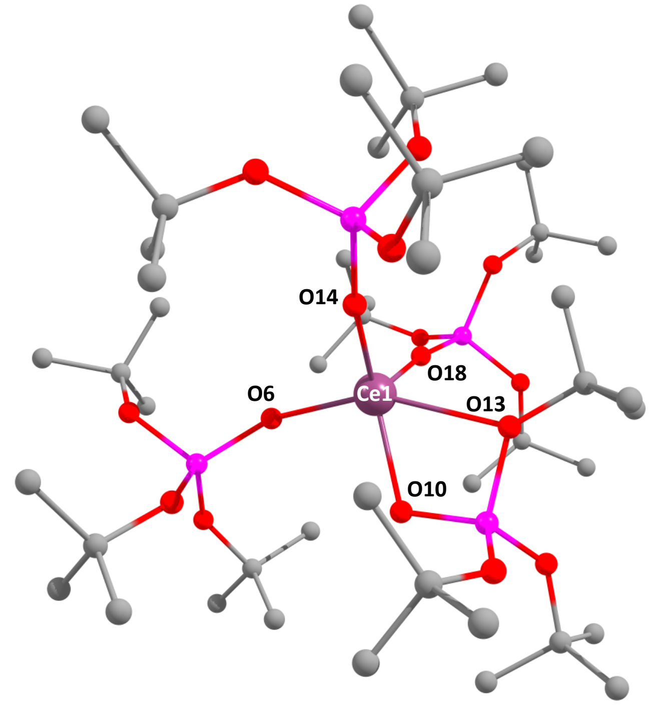

Table S4. Computed natural charges for selected atoms in Ce[OSi(O^{t}Bu)$_3$]$_4$ (dispersion)

Atom labels	Natural charges
Ce1	2.09761
O6	-1.18543
O10	-1.17125
O13	-0.96707
O14	-1.19965
O18	-1.19916

Table S5. Computed Wiberg bond index between selected atoms in Ce[OSi(O^{t}Bu)$_3$]$_4$ complex (dispersion)

Atom labels	Wiberg bond index	Atom labels	Wiberg bond index	Atom labels	Wiberg bond index
Ce1	0.0000	Ce1	0.0000	Ce1	0.0000
O6	0.7367	O10	0.7133	O13	0.2250
Atom labels	Wiberg bond index	Atom labels	Wiberg bond index		
Ce1	0.0000	Ce1	0.0000		
O14	0.7402	O18	0.7387		

Bonding orbitals (Alpha molecular orbital) between cerium and oxygen atoms in the first coordination sphere of Ce[OSi(O^{t}Bu)$_3$]$_4$ complex (dispersion)

```
(1. 94304) BD ( 1)Ce  1- O  6
(  8.57%)   0.2928*Ce  1 s(  0.45%)p 1.13(  0.51%)d99.99( 51.15%)f99.99( 47.61%)g 0.60(  0.27%)
( 91.43%)   0.9562* O  6 s( 16.25%)p 5.15( 83.71%)d 0.00(  0.03%)
(1.91742) BD ( 2)Ce  1- O  6
(  6.26%)   0.2501*Ce  1 s(  0.23%)p 0.89(  0.20%)d99.99( 46.89%)f99.99( 52.18%)g 2.18(  0.50%)
( 93.74%)   0.9682* O  6 s(  0.14%)p99.99( 99.80%)d 0.39(  0.06%)
(1.93879) BD ( 1)Ce  1- O 10
(  9.22%)   0.3036*Ce  1 s(  1.03%)p 0.22(  0.22%)d46.32( 47.84%)f48.97( 50.58%)g 0.31(  0.33%)
( 93.80%)   0.9685* O 10 s(  0.00%)p 1.00( 99.88%)d 0.00(  0.12%)
(1.94059) BD ( 1)Ce  1- O 14
(  7.94%)   0.2817*Ce  1 s(  0.03%)p24.17(  0.73%)d99.99( 47.59%)f99.99( 51.22%)g14.30(  0.43%)
( 92.06%)   0.9595* O 14 s( 15.18%)p 5.59( 84.81%)d 0.00(  0.01%)
(1.93159) BD ( 2)Ce  1- O 14
(  6.35%)   0.2520*Ce  1 s(  0.08%)p 2.38(  0.18%)d99.99( 47.48%)f99.99( 51.87%)g 4.98(  0.38%)
```

(93.65%) 0.9677* O 14 s(0.02%)p99.99(99.95%)d 0.98(0.02%)
(1.94193) BD (1)Ce 1- O 18
(7.86%) 0.2804*Ce 1 s(0.31%)p 2.01(0.62%)d99.99(51.51%)f99.99(47.25%)g 1.03(0.32%)
(92.14%) 0.9599* O 18 s(11.19%)p 7.94(88.80%)d 0.00(0.01%)
(1.92590) BD (2)Ce 1- O 18
(6.14%) 0.2478*Ce 1 s(0.01%)p 6.72(0.10%)d99.99(52.08%)f99.99(47.31%)g34.65(0.50%)
(93.86%) 0.9688* O 18 s(0.08%)p99.99(99.90%)d 0.22(0.02%)

Table S6. NBO Second order perturbation analysis (Alpha molecular orbital) for Ce[OSi(O^{t}Bu)$_3$]$_4$ complex (dispersion)

Donor NBO	Acceptor NBO	E(2) kcal/mol
(1.87243) LP (1) O 6<br>s(44.73%)p 1.23(55.22%)d 0.00(0.05%)	(0.14568) LV (1)Ce 1<br>s(0.11%)p 1.55(0.17%)d99.99(47.58%)f99.99(51.91%)g 1.93(0.22%)	15.82
(1.87243) LP (1) O 6<br>s(44.73%)p 1.23(55.22%)d 0.00(0.05%)	(0.07277) LV (2)Ce 1<br>s(14.10%)p 0.04(0.55%)d 2.43(34.24%)f 3.57(50.37%)g 0.05(0.73%)	5.11
(1.87243) LP (1) O 6<br>s(44.73%)p 1.23(55.22%)d 0.00(0.05%)	(0.05630) BD*(2)Ce 1- O 14<br>(93.65%) 0.9677*Ce 1 s(0.08%)p 2.38(0.18%)d99.99(47.48%)f99.99(51.87%)g 4.98(0.38%)<br>(6.35%) -0.2520* O 14 s(0.02%)p99.99(99.95%)d 0.98(0.02%)	5.54
(1.87243) LP (1) O 6<br>s(44.73%)p 1.23(55.22%)d 0.00(0.05%)	(0.06812) BD*(1)Ce 1- O 18<br>(92.14%) 0.9599*Ce 1 s(0.31%)p 2.01(0.62%)d99.99(51.51%)f99.99(47.25%)g 1.03(0.32%)<br>(7.86%) -0.2804* O 18 s(11.19%)p 7.94(88.80%)d 0.00(0.01%)	8.34
(1.89540) LP (1) O 10<br>s(49.25%)p 1.03(50.68%)d 0.00(0.07%)	(0.14568) LV (1)Ce 1<br>s(0.11%)p 1.55(0.17%)d99.99(47.58%)f99.99(51.91%)g 1.93(0.22%)	11.03
(1.89540) LP (1) O 10<br>s(49.25%)p 1.03(50.68%)d 0.00(0.07%)	(0.02558) LV (5)Ce 1<br>s(1.22%)p 1.38(1.67%)d39.80(48.38%)f38.96(47.36%)g 1.13(1.38%)	6.37
(1.89540) LP (1) O 10	(0.06819) BD*(1)Ce 1- O 6	8.48

s(49.25%)p 1.03(50.68%)d 0.00(0.07%)	(91.43%) 0.9562*Ce 1 s(0.45%)p 1.13(0.51%)d99.99(51.15%)f99.99(47.61%)g 0.60(0.27%)<br>(8.57%) -0.2928* O 6 s(16.25%)p 5.15(83.71%)d 0.00(0.03%)	
(1.87686) LP (2) O 13<br>s(20.04%)p 3.99(79.94%)d 0.00(0.02%)	(0.04437) LV (4)Ce 1<br>s(1.84%)p 0.52(0.95%)d15.59(28.66%)f36.72(67.49%)g 0.58(1.06%)	7.63
(1.87686) LP (2) O 13<br>s(20.04%)p 3.99(79.94%)d 0.00(0.02%)	(0.06819) BD*(1)Ce 1- O 6<br>(91.43%) 0.9562*Ce 1 s(0.45%)p 1.13(0.51%)d99.99(51.15%)f99.99(47.61%)g 0.60(0.27%)<br>(8.57%) -0.2928* O 6 s(16.25%)p 5.15(83.71%)d 0.00(0.03%)	6.91
(1.85014) LP (1) O 14<br>s(39.28%)p 1.55(60.69%)d 0.00(0.03%)	(0.14568) LV (1)Ce 1<br>s(0.11%)p 1.55(0.17%)d99.99(47.58%)f99.99(51.91%)g 1.93(0.22%)	24.51
(1.85014) LP (1) O 14<br>s(39.28%)p 1.55(60.69%)d 0.00(0.03%)	(0.06819) BD*(1)Ce 1- O 6<br>(91.43%) 0.9562*Ce 1 s(0.45%)p 1.13(0.51%)d99.99(51.15%)f99.99(47.61%)g 0.60(0.27%)<br>(8.57%) -0.2928* O 6 s(16.25%)p 5.15(83.71%)d 0.00(0.03%)	6.08
(1.85014) LP (1) O 14<br>s(39.28%)p 1.55(60.69%)d 0.00(0.03%)	(0.04756) BD*(2)Ce 1- O 10<br>(93.80%) 0.9685*Ce 1 s(0.03%)p 9.18(0.29%)d99.99(39.13%)f99.99(60.03%)g16.28(0.51%)<br>(6.20%) -0.2489* O 10 s(0.00%)p 1.00(99.88%)d 0.00(0.12%)	12.10

Figure S74. DFT computed MOs for Ce[OSi(O^{t}Bu)$_3$]$_4$ complex, s=0.

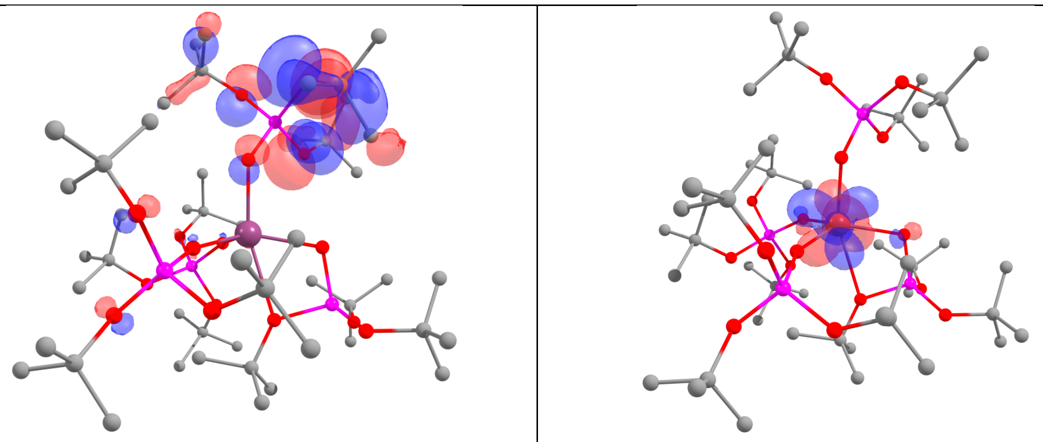

HOMO LUMO

Table S7. Selected structural parameters comparison between DFT optimized and X-ray structures for KCe[OSi(O^{t}Bu)$_3$]$_4$ complex (s=1/2)

Atom labels	DFT		X-ray
	No dispersion	dispersion	
Ce1-O10	2.24	2.21	2.24
Ce1-O11	2.29	2.27	2.29
Ce1-O15	2.25	2.22	2.26
Ce1-O19	2.29	2.31	2.29
Ce1-O20	2.84	2.64	2.85

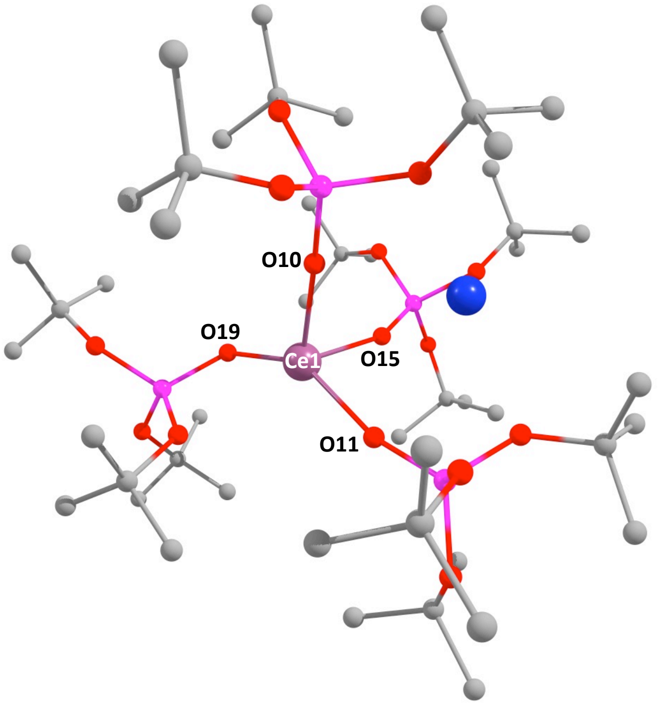

Table S8. Computed natural charges for selected atoms in $KCe[OSi(O^tBu)_3]_4$ complex (dispersion)

Atom labels	Natural charges
Ce1	1.94930
O10	-1.32820
O11	-1.31582
O15	-1.33280
O19	-1.28186
O20	-0.99111

Table S9. Computed Wiberg bond index between selected atoms in $KCe[OSi(O^tBu)_3]_4$ complex (dispersion)

Atom labels	Wiberg bond index	Atom labels	Wiberg bond index	Atom labels	Wiberg bond index
Ce1	0.0000	Ce1	0.0000	Ce1	0.0000
O10	0.4219	O11	0.3855	O15	0.4120
Atom labels	Wiberg bond index	Atom labels	Wiberg bond index		
Ce1	0.0000	Ce1	0.0000		
O19	0.4039	O20	0.1490		

Table S10. NBO Second order perturbation analysis (Alpha molecular orbital) for $KCe[OSi(O^tBu)_3]_4$ complex (dispersion)

Donor NBO	Acceptor NBO	E(2) kcal/mol
(0.94123) LP (1) O 10<br><br>s(38.13%)p 1.62(61.86%)d 0.00(0.01%)	(0.08949) LV (2)Ce 1<br><br>s(0.00%)p 1.00(0.55%)d99.99(92.53%)f12.42(6.87%)g 0.08(0.05%)	26.43
(0.92341) LP (3) O 10<br><br>s(0.03%)p99.99(99.95%)d 0.63(0.02%)	(0.07389) LV (4)Ce 1<br><br>s(0.04%)p 4.35(0.18%)d99.99(94.15%)f99.99(5.58%)g 1.26(0.05%)	5.14
(0.98139) BD (1)Si 3- O 10	(0.08949) LV (2)Ce 1	9.73

(12.23%) 0.3497*Si 3 s(24.55%)p 3.03(74.40%)d 0.04(1.05%)<br>(87.77%) 0.9369* O 10 s(61.77%)p 0.62(38.11%)d 0.00(0.12%)	s(0.00%)p 1.00(0.55%)d99.99(92.53%)f12.42(6.87%)g 0.08(0.05%)	
(0.94778) LP (1) O 11<br>s(43.31%)p 1.31(56.66%)d 0.00(0.03%)	(0.09623) LV (1)Ce 1<br>s(0.04%)p 8.27(0.30%)d99.99(96.44%)f85.41(3.15%)g 2.03(0.07%)	9.52
(0.94778) LP (1) O 11<br>s(43.31%)p 1.31(56.66%)d 0.00(0.03%)	(0.08731) LV (3)Ce 1<br>s(0.00%)p 1.00(0.22%)d99.99(95.79%)f18.04(3.88%)g 0.52(0.11%)	11.14
(0.97807) BD (1)Si 4- O 11<br>(12.58%) 0.3547*Si 4 s(25.56%)p 2.88(73.50%)d 0.04(0.94%)<br>(87.42%) 0.9350* O 11 s(56.39%)p 0.77(43.46%)d 0.00(0.15%)	(0.09623) LV (1)Ce 1<br>s(0.04%)p 8.27(0.30%)d99.99(96.44%)f85.41(3.15%)g 2.03(0.07%)	8.23
(0.94259) LP (1) O 15<br>s(37.29%)p 1.68(62.70%)d 0.00(0.00%)	(0.09623) LV (1)Ce 1<br>s(0.04%)p 8.27(0.30%)d99.99(96.44%)f85.41(3.15%)g 2.03(0.07%)	10.65
(0.94259) LP (1) O 15<br>s(37.29%)p 1.68(62.70%)d 0.00(0.00%)	(0.08949) LV (2)Ce 1<br>s(0.00%)p 1.00(0.55%)d99.99(92.53%)f12.42(6.87%)g 0.08(0.05%)	10.23
(0.94259) LP (1) O 15<br>s(37.29%)p 1.68(62.70%)d 0.00(0.00%)	(0.08731) LV (3)Ce 1<br>s(0.00%)p 1.00(0.22%)d99.99(95.79%)f18.04(3.88%)g 0.52(0.11%)	10.93
(0.92374) LP (3) O 15<br>s(0.00%)p 1.00(99.98%)d 0.00(0.02%)	(0.07031) LV (5)Ce 1<br>s(0.12%)p 0.84(0.10%)d99.99(91.50%)f68.34(8.24%)g 0.29(0.03%)	7.28
(0.95777) LP (1) O 19<br>s(49.05%)p 1.04(50.87%)d 0.00(0.07%)	(0.09623) LV (1)Ce 1<br>s(0.04%)p 8.27(0.30%)d99.99(96.44%)f85.41(3.15%)g 2.03(0.07%)	13.70
(0.92016) LP (2) O 19<br>s(1.46%)p67.60(98.43%)d 0.08(0.12%)	(0.09623) LV (1)Ce 1<br>s(0.04%)p 8.27(0.30%)d99.99(96.44%)f85.41(3.15%)g 2.03(0.07%)	12.04
(0.95443) LP (1) O 20	(0.08731) LV (3)Ce 1	5.84

s(17.89%)p 4.59(82.08%)d 0.00(0.03%)	s(0.00%)p 1.00(0.22%)d99.99(95.79%)f18.04(3.88%)g 0.52(0.11%)	

Figure S75. DFT computed MOs for $KCe[OSi(O^tBu)_3]_4$ complex, dispersion, Alpha MOs.

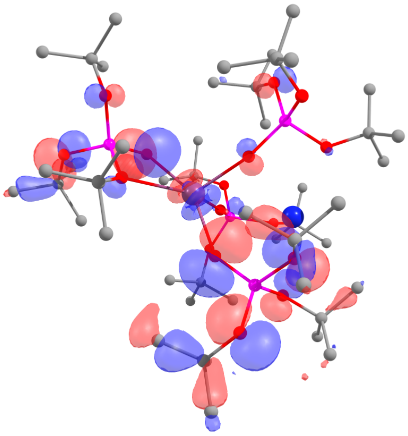 HOMO-1	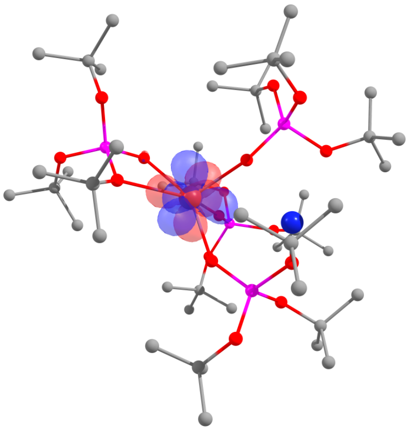 HOMO
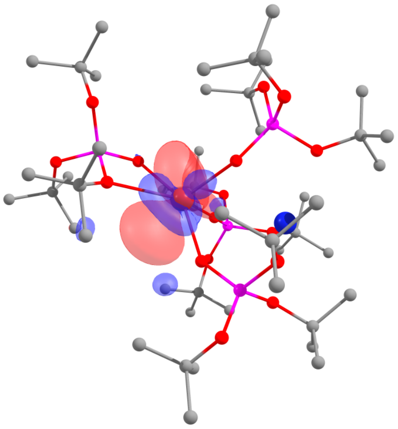 LUMO	

Table S11. Selected structural parameters comparison between DFT optimized and X-ray structures for $Pr[OSi(O^tBu)_3]_4$ (**2-Pr^{OtBu}**) complex (s=1/2)

Atom labels	DFT		X-ray	
	No dispersion	dispersion		
Pr1-O6	2.10	2.09	2.09	
Pr1-O7	2.09	2.09	2.09	
Pr1-O8	2.11	2.08	2.08	

Pr1-O11	2.60	2.55	2.57	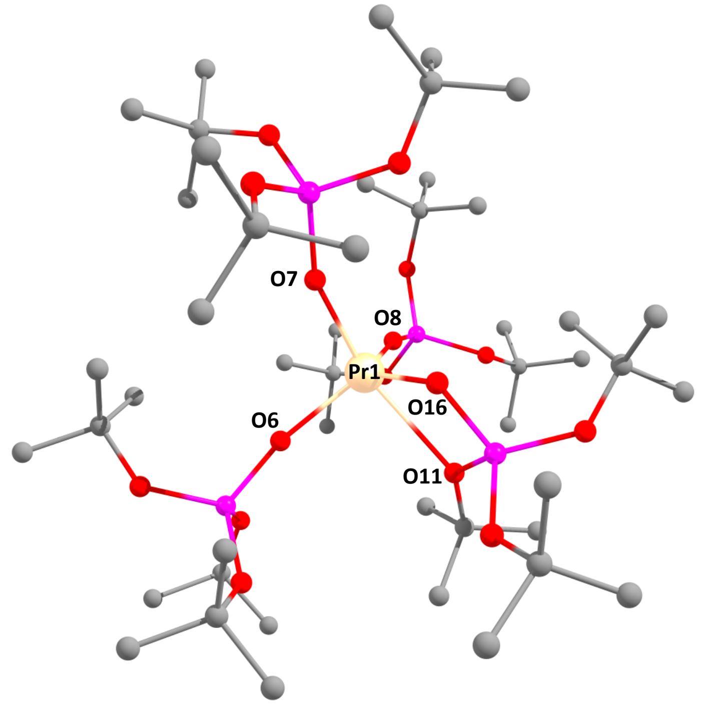
Pr1-O16	2.16	2.17	2.17	

Table S12. Computed natural charges for selected atoms in **2-Pr^{OtBu}** complex (dispersion)

Atom labels	Natural charges
Pr1	2.02177
O6	-1.18773
O7	-1.15979
O8	-1.17902
O11	-0.96351
O16	-1.16211

Table S13. Computed Wiberg bond index between selected atoms in **2-Pr^{OtBu}** complex (dispersion)

Atom labels	Wiberg bond index	Atom labels	Wiberg bond index	Atom labels	Wiberg bond index
Pr1	0.0000	Pr1	0.0000	Pr1	0.0000
O6	0.7488	O7	0.7834	O8	0.7700
Atom labels	Wiberg bond index	Atom labels	Wiberg bond index		

Pr1	0.0000	Pr1	0.0000		
O11	0.2463	O16	0.7080		

Bonding orbitals (Alpha molecular orbital) between praseodymium and oxygen atoms in the first coordination sphere of **2-Pr^OtBu^** complex (dispersion)
(0.97180) BD (1)Pr 1- O 6
(8.54%) 0.2923*Pr 1 s(0.23%)p 2.23(0.52%)d99.99(49.52%)f99.99(49.53%)g 0.86(0.20%)
(91.46%) 0.9563* O 6 s(10.63%)p 8.40(89.35%)d 0.00(0.01%)
(0.97638) BD (1)Pr 1- O 7
(11.33%) 0.3366*Pr 1 s(0.33%)p 1.13(0.38%)d99.99(41.17%)f99.99(57.98%)g 0.41(0.14%)
(88.67%) 0.9416* O 7 s(12.74%)p 6.85(87.23%)d 0.00(0.03%)
(0.96004) BD (2)Pr 1- O 7
(7.23%) 0.2688*Pr 1 s(0.20%)p 0.69(0.14%)d99.99(50.64%)f99.99(48.76%)g 1.22(0.25%)
(92.77%) 0.9632* O 7 s(0.20%)p99.99(99.75%)d 0.23(0.05%)
(0.97118) BD (1)Pr 1- O 8
(9.32%) 0.3054*Pr 1 s(0.03%)p17.49(0.57%)d99.99(46.76%)f99.99(52.36%)g 8.68(0.28%)
(90.68%) 0.9522* O 8 s(13.37%)p 6.48(86.61%)d 0.00(0.01%)
(0.96709) BD (2)Pr 1- O 8
(7.45%) 0.2729*Pr 1 s(0.07%)p 2.54(0.18%)d99.99(52.07%)f99.99(47.51%)g 2.48(0.17%)
(92.55%) 0.9620* O 8 s(0.01%)p 1.00(99.96%)d 0.00(0.03%)
(0.96779) BD (1)Pr 1- O 16
(9.55%) 0.3090*Pr 1 s(1.03%)p 0.15(0.15%)d51.89(53.33%)f44.00(45.23%)g 0.26(0.26%)
(90.45%) 0.9511* O 16 s(16.89%)p 4.92(83.02%)d 0.01(0.09%)
(0.95379) BD (2)Pr 1- O 16
(7.78%) 0.2789*Pr 1 s(0.01%)p19.36(0.25%)d99.99(35.86%)f99.99(63.66%)g17.13(0.22%)
(92.22%) 0.9603* O 16 s(0.03%)p99.99(99.84%)d 3.56(0.12%)

Table S17. NBO Second order perturbation analysis (Alpha molecular orbital) for **2-Pr^OtBu^** complex (dispersion)

Donor NBO	Acceptor NBO	E(2) kcal/mol
(0.91910) LP (1) O 6<br>s(47.36%)p 1.11(52.61%)d 0.00(0.03%)	(0.04139) LV (3)Pr 1<br>s(4.28%)p 0.11(0.45%)d 9.82(42.03%)f12.33(52.74%)g 0.11(0.49%)	8.65
(0.91910) LP (1) O 6<br>s(47.36%)p 1.11(52.61%)d 0.00(0.03%)	(0.04228) BD*(1)Pr 1- O 8<br>(90.68%) 0.9522*Pr 1 s(0.03%)p17.49(0.57%)d99.99(46.76%)f99.99(52.36%)g 8.68(0.28%)<br>(9.32%) -0.3054* O 8 s(13.37%)p 6.48(86.61%)d 0.00(0.01%)	6.37
(0.90176) LP (2) O 6	(0.07875) LV (1)Pr 1	5.78

s(0.07%)p99.99(99.90%)d 0.33(0.02%)	s(0.90%)p 0.04(0.03%)d84.51(76.29%)f25.01(22.58%)g 0.22(0.20%)	
(0.93307) LP (1) O 7<br>s(47.57%)p 1.10(52.38%)d 0.00(0.05%)	(0.07875) LV (1)Pr 1<br>s(0.90%)p 0.04(0.03%)d84.51(76.29%)f25.01(22.58%)g 0.22(0.20%)	11.07
(0.92273) LP (1) O 8<br>s(43.55%)p 1.30(56.41%)d 0.00(0.04%)	(0.07165) LV (2)Pr 1<br>s(0.04%)p 3.60(0.14%)d99.99(29.53%)f99.99(70.11%)g 4.57(0.18%)	14.32
(0.92273) LP (1) O 8<br>s(43.55%)p 1.30(56.41%)d 0.00(0.04%)	(0.03400) BD*(1)Pr 1- O 6<br>(91.46%) 0.9563*Pr 1 s(0.23%)p 2.23(0.52%)d99.99(49.52%)f99.99(49.53%)g 0.86(0.20%)<br>(8.54%) -0.2923* O 6 s(10.63%)p 8.40(89.35%)d 0.00(0.01%)	5.07
(0.92273) LP (1) O 8<br>s(43.55%)p 1.30(56.41%)d 0.00(0.04%)	(0.02778) BD*(2)Pr 1- O 16<br>(92.22%) 0.9603*Pr 1 s(0.01%)p19.36(0.25%)d99.99(35.86%)f99.99(63.66%)g17.13(0.22%)<br>(7.78%) -0.2789* O 16 s(0.03%)p99.99(99.84%)d 3.56(0.12%)	5.13
(0.93250) LP (2) O 11<br>s(19.49%)p 4.13(80.49%)d 0.00(0.02%)	(0.07875) LV (1)Pr 1<br>s(0.90%)p 0.04(0.03%)d84.51(76.29%)f25.01(22.58%)g 0.22(0.20%)	7.98
(0.95043) LP (1) O 16<br>s(49.32%)p 1.03(50.60%)d 0.00(0.08%)	(0.07875) LV (1)Pr 1<br>s(0.90%)p 0.04(0.03%)d84.51(76.29%)f25.01(22.58%)g 0.22(0.20%)	4.69

Figure S76. DFT computed MOs for **2-Pr^{OtBu}** complex, s=1/2, Alpha MOs.

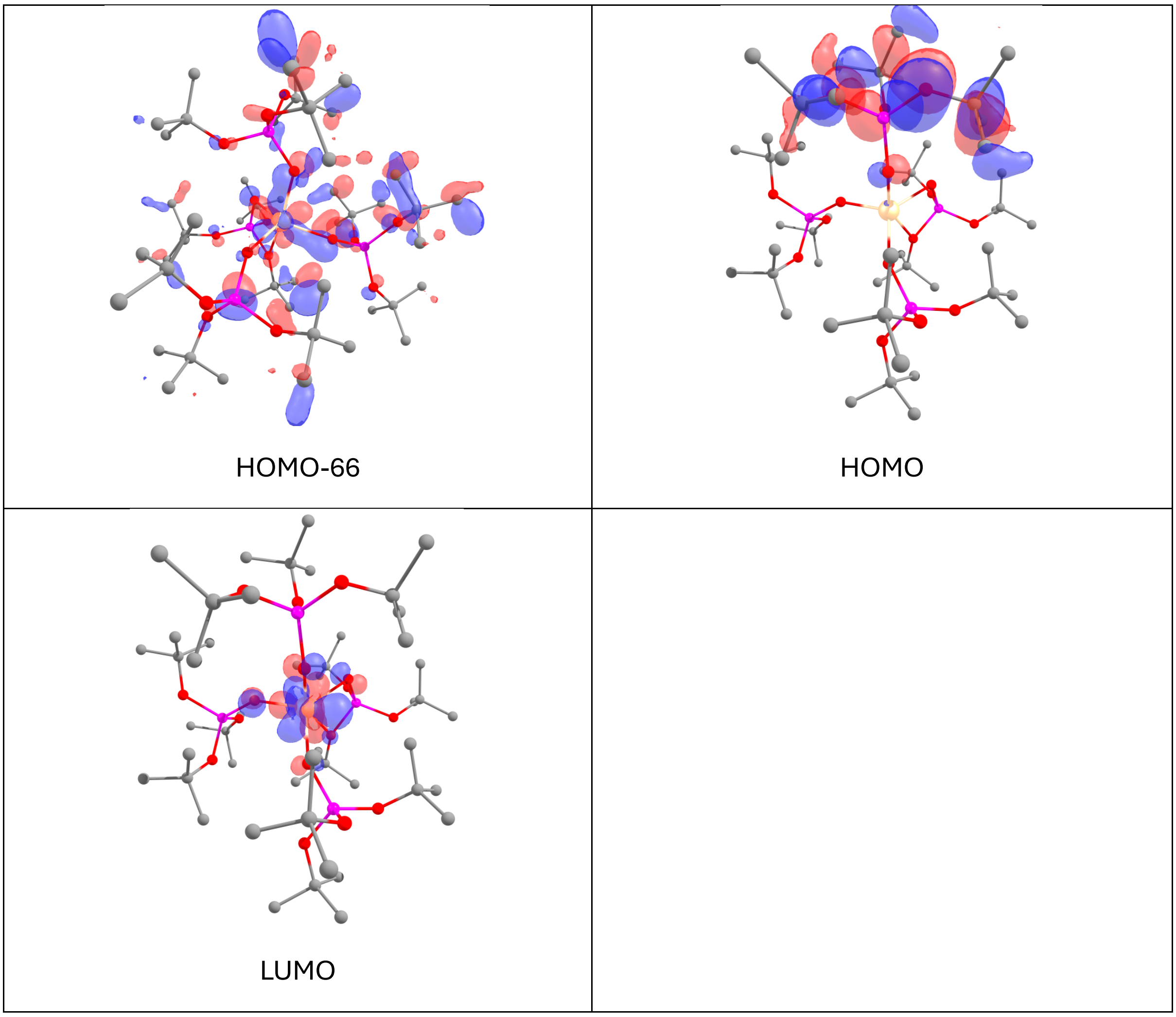

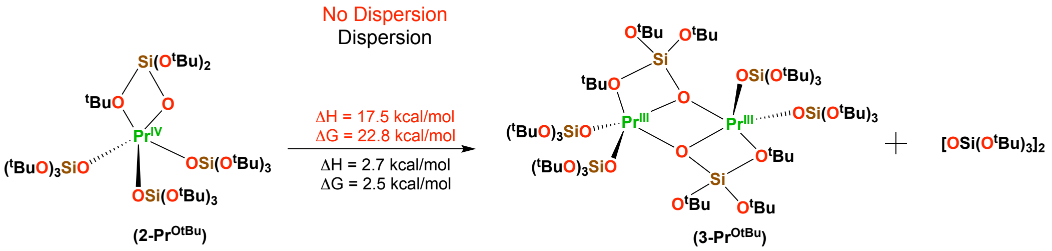

Table S22. Selected structural parameters comparison between DFT optimized structures (with and without dispersion) for {PrIII}$_2$, [Pr(OSi(O^{t}Bu)$_3$)$_3$]$_2$ (**3-Pr^{OtBu}**) complex, (s=2)

Atom labels	No dispersion	Dispersion
Pr1-O5	2.21	2.18
Pr1-O6	2.17	2.18

Pr1-O9	2.53	2.50
Pr1-O12	2.60	2.50
Pr1-O145	2.39	2.35
O12-Pr134	2.39	2.31
Pr134-O138	2.19	2.15
Pr134-O139	2.18	2.17
Pr134-O142	2.56	2.54
Pr134-O145	2.55	2.58

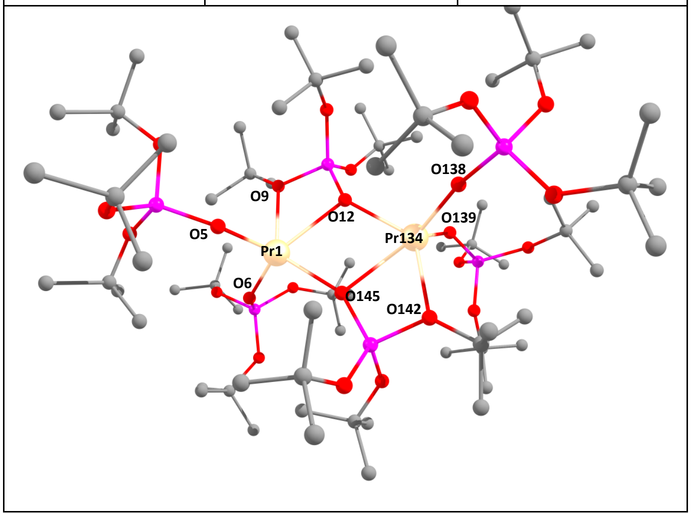

Table S23. Computed natural charges for selected atoms in **3-Pr^{OtBu}** complex, s=2, (dispersion)

Atom labels	Natural charges
Pr1	1.95170
O5	-1.29782
O6	-1.29406
O9	-0.99484
O12	-1.30166
Pr134	1.99801
O138	-1.30694
O139	-1.28936
O142	-0.99760
O145	-1.30266

Table S24. Computed Wiberg bond index between selected atoms in **3-Pr**$^{\mathbf{OtBu}}$ complex, s=2, (dispersion)

Atom labels	Wiberg bond index	Atom labels	Wiberg bond index	Atom labels	Wiberg bond index
Pr1	0.0000	Pr1	0.0000	Pr1	0.0000
O5	0.4994	O6	0.4994	O9	0.1868
Atom labels	Wiberg bond index	Atom labels	Wiberg bond index	Atom labels	Wiberg bond index
Pr1	0.0000	Pr1	0.0000	Pr134	0.0000
O12	0.2323	O145	0.2961	O12	0.3049
Atom labels	Wiberg bond index	Atom labels	Wiberg bond index	Atom labels	Wiberg bond index
Pr134	0.0000	Pr134	0.0000	Pr134	0.0000
O138	0.4917	O139	0.4929	O142	0.1657
Atom labels	Wiberg bond index				
Pr134	0.0000				
O145	0.2045				

Bonding orbitals (Alpha molecular orbital) between praseodymium and oxygen atoms in the first coordination sphere of **3-Pr**$^{\mathbf{OtBu}}$ complex, s=2, (dispersion)
(0.98824) BD (1)Pr 1- O 5
(5.19%) 0.2279*Pr 1 s(0.29%)p 1.93(0.57%)d99.99(80.37%)f63.19(18.55%)g 0.76(0.22%)
(94.81%) 0.9737* O 5 s(52.82%)p 0.89(47.15%)d 0.00(0.03%)
(0.98944) BD (1)Pr 1- O 6
(5.34%) 0.2311*Pr 1 s(0.23%)p 1.53(0.35%)d99.99(73.34%)f99.99(25.88%)g 0.89(0.20%)
(94.66%) 0.9729* O 6 s(53.24%)p 0.88(46.74%)d 0.00(0.02%)
(0.98852) BD (1)Pr134- O138
(5.20%) 0.2280*Pr134 s(0.67%)p 1.19(0.79%)d99.99(76.89%)f32.00(21.38%)g 0.40(0.27%)
(94.80%) 0.9737* O138 s(52.67%)p 0.90(47.32%)d 0.00(0.01%)
(0.98853) BD (1)Pr134- O139
(5.03%) 0.2242*Pr134 s(0.49%)p 0.85(0.41%)d99.99(75.41%)f48.25(23.45%)g 0.49(0.24%)
(94.97%) 0.9745* O139 s(53.07%)p 0.88(46.90%)d 0.00(0.03%)

Table S25. NBO Second order perturbation analysis (Alpha molecular orbital) for **3-Pr^{OtBu}** complex, s=2, (dispersion)

Donor NBO	Acceptor NBO	E(2) kcal/mol
(0.91971) LP (1) O 5<br>s(0.00%)p 1.00(99.95%)d 0.00(0.04%)	(0.08178) LV (1)Pr 1<br>s(0.11%)p 0.39(0.04%)d99.99(95.85%)f36.84(3.96%)g 0.44(0.05%)	4.15
(0.91234) LP (2) O 5<br>s(0.45%)p99.99(99.49%)d 0.11(0.05%)	(0.07956) LV (2)Pr 1<br>s(0.01%)p12.13(0.15%)d99.99(95.41%)f99.99(4.37%)g 4.10(0.05%)	8.42
(0.91833) LP (1) O 6<br>s(0.00%)p 1.00(99.95%)d 0.00(0.04%)	(0.08178) LV (1)Pr 1<br>s(0.11%)p 0.39(0.04%)d99.99(95.85%)f36.84(3.96%)g 0.44(0.05%)	6.03
(0.94160) LP (2) O 9<br>s(35.52%)p 1.82(64.47%)d 0.00(0.01%)	(0.04434) LV (4)Pr 1<br>s(19.84%)p 0.04(0.77%)d 2.39(47.43%)f 1.59(31.62%)g 0.02(0.33%)	7.06
(0.91572) LP (3) O 12<br>s(27.71%)p 2.61(72.25%)d 0.00(0.04%)	(0.07956) LV (2)Pr 1<br>s(0.01%)p12.13(0.15%)d99.99(95.41%)f99.99(4.37%)g 4.10(0.05%)	4.53
(0.91572) LP (3) O 12<br>s(27.71%)p 2.61(72.25%)d 0.00(0.04%)	(0.07205) LV (3)Pr 1<br>s(0.30%)p 0.25(0.07%)d99.99(72.91%)f88.14(26.56%)g 0.50(0.15%)	9.35
(0.93715) LP (1) O 12<br>s(42.37%)p 1.36(57.61%)d 0.00(0.02%)	(0.07187) LV (2)Pr134<br>s(0.15%)p 2.12(0.31%)d99.99(89.41%)f68.17(10.08%)g 0.27(0.04%)	9.39
(0.93715) LP (1) O 12<br>s(42.37%)p 1.36(57.61%)d 0.00(0.02%)	(0.03389) LV (4)Pr134<br>s(27.13%)p 0.02(0.45%)d 0.77(20.89%)f 1.88(51.06%)g 0.02(0.47%)	4.54
(0.91572) LP (3) O 12<br>s(27.71%)p 2.61(72.25%)d 0.00(0.04%)	(0.07187) LV (2)Pr134<br>s(0.15%)p 2.12(0.31%)d99.99(89.41%)f68.17(10.08%)g 0.27(0.04%)	7.63
(0.93622) LP (1) O145<br>s(50.71%)p 0.97(49.29%)d 0.00(0.00%)	(0.07205) LV (3)Pr 1<br>s(0.30%)p 0.25(0.07%)d99.99(72.91%)f88.14(26.56%)g 0.50(0.15%)	21.35

(0.91464) LP (2) O138<br><br>s(0.12%)p99.99(99.86%)d 0.12(0.01%)	(0.07381) LV (1)Pr134<br><br>s(0.03%)p 2.43(0.08%)d99.99(95.73%)f99.99(4.12%)g 1.13(0.04%)	4.55
(0.91464) LP (2) O138<br><br>s(0.12%)p99.99(99.86%)d 0.12(0.01%)	(0.07187) LV (2)Pr134<br><br>s(0.15%)p 2.12(0.31%)d99.99(89.41%)f68.17(10.08%)g 0.27(0.04%)	4.17
(0.91315) LP (1) O139<br><br>s(0.12%)p99.99(99.82%)d 0.43(0.05%)	(0.07381) LV (1)Pr134<br><br>s(0.03%)p 2.43(0.08%)d99.99(95.73%)f99.99(4.12%)g 1.13(0.04%)	6.90
(0.94232) LP (2) O142<br><br>s(27.44%)p 2.64(72.55%)d 0.00(0.01%)	(0.07187) LV (2)Pr134<br><br>s(0.15%)p 2.12(0.31%)d99.99(89.41%)f68.17(10.08%)g 0.27(0.04%)	4.63

Figure S77. DFT computed MOs for **3-Pr^{OtBu}** complex, (s=2), Alpha MOs.

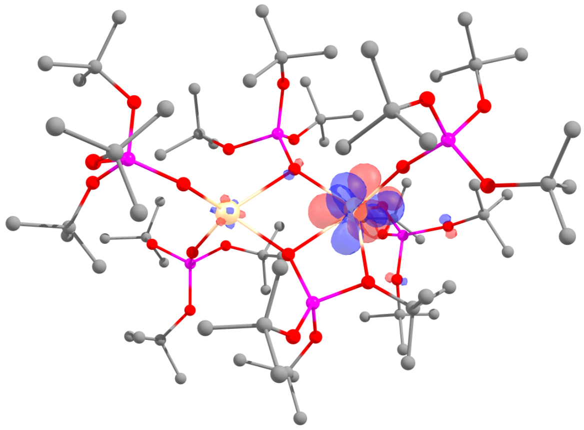

HOMO-3

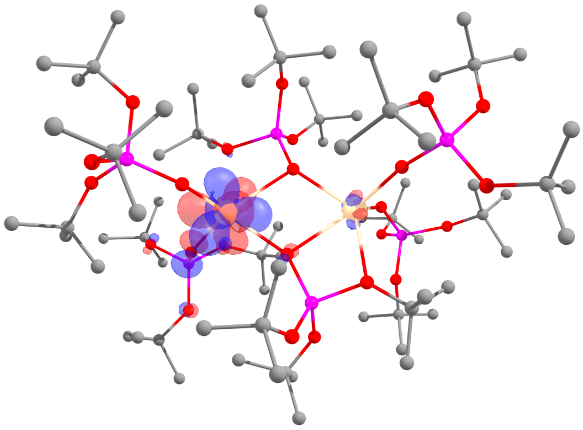

HOMO-2

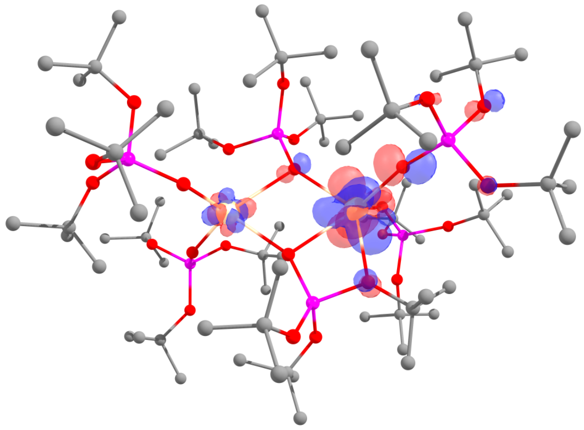

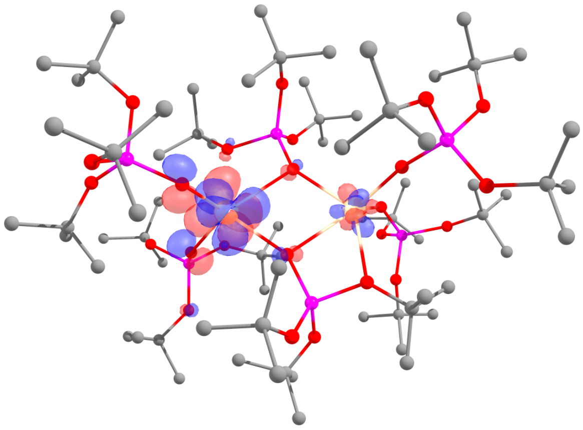

HOMO

HOMO-1	
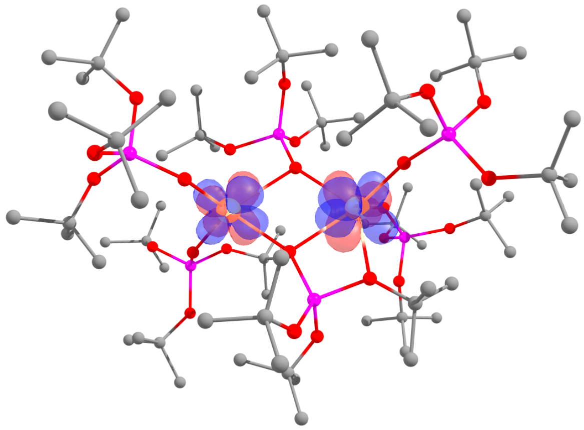 LUMO	

Table S26. Selected structural parameters comparison between DFT optimized and X-ray structures for **5K-Pr^{Ph}** complex, (s=1/2)

Atom labels	DFT		X-ray	
	No dispersion	dispersion		
Pr1-O8	2.21	2.20	2.18	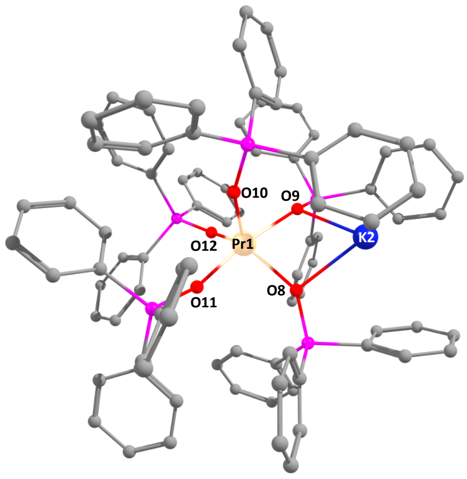
Pr1-O9	2.23	2.21	2.23	
Pr1-O10	2.13	2.09	2.10	
Pr1-O11	2.13	2.11	2.14	
Pr1-O12	2.13	2.10	2.13	

Table S27. Computed natural charges for selected atoms in **5K-Pr^{Ph}** complex, dispersion

Atom labels	Natural charges
Pr1	1.92811
K2	0.95902
O8	-1.22098
O9	-1.19291
O10	-1.15135
O11	-1.13047
O12	-1.14681

Table S28. Computed Wiberg bond index between selected atoms in **5K-Pr**^{Ph} complex, dispersion

Atom labels	Wiberg bond index	Atom labels	Wiberg bond index	Atom labels	Wiberg bond index
Pr1	0.0000	Pr1	0.0000	Pr1	0.0000
O8	0.5787	O9	0.6082	O10	0.7276
Atom labels	Wiberg bond index	Atom labels	Wiberg bond index		
Pr1	0.0000	Pr1	0.0000		
O11	0.7586	O12	0.7481		

Bonding orbitals (Alpha molecular orbital) between praseodymium and oxygen atoms in the first coordination sphere of **5K-Pr**^{Ph} complex, dispersion

(0.96982) BD (1)Pr 1- O 8
(7.24%) 0.2690*Pr 1 s(1.05%)p 0.18(0.19%)d36.71(38.64%) f56.93(59.93%)g 0.17(0.18%)
(92.76%) 0.9631* O 8 s(18.06%)p 4.54(81.93%)d 0.00(0.01%)
(0.95552) BD (2)Pr 1- O 8
(5.33%) 0.2309*Pr 1 s(0.10%)p 3.06(0.30%)d99.99(66.80%) f99.99(32.57%)g 2.47(0.24%)
(94.67%) 0.9730* O 8 s(0.01%)p99.99(99.96%)d 2.34(0.03%)
(0.96445) BD (1)Pr 1- O 9
(8.75%) 0.2959*Pr 1 s(1.77%)p 0.09(0.16%)d25.01(44.29%) f30.24(53.56%)g 0.12(0.21%)
(91.25%) 0.9552* O 9 s(11.23%)p 7.91(88.76%)d 0.00(0.01%)
(0.96771) BD (1)Pr 1- O 10
(8.02%) 0.2832*Pr 1 s(1.06%)p 0.47(0.50%)d34.83(36.82%) f57.99(61.31%)g 0.30(0.32%)
(91.98%) 0.9591* O 10 s(12.82%)p 6.80(87.17%)d 0.00(0.01%)
(0.95869) BD (2)Pr 1- O 10
(6.59%) 0.2567*Pr 1 s(0.01%)p 1.00(0.28%)d99.99(56.78%) f99.99(42.68%)g 0.90(0.25%)
(93.41%) 0.9665* O 10 s(0.01%)p 1.00(99.98%)d 0.00(0.02%)
(0.96577) BD (1)Pr 1- O 11
(9.33%) 0.3054*Pr 1 s(0.99%)p 0.23(0.23%)d44.75(44.17%) f55.07(54.35%)g 0.28(0.28%)
(90.67%) 0.9522* O 11 s(9.67%)p 9.34(90.32%)d 0.00(0.01%)
(0.96053) BD (2)Pr 1- O 11
(6.76%) 0.2601*Pr 1 s(0.03%)p 4.91(0.17%)d99.99(52.96%) f99.99(46.62%)g 5.96(0.21%)
(93.24%) 0.9656* O 11 s(0.02%)p99.99(99.96%)d 0.57(0.01%)
(0.96877) BD (1)Pr 1- O 12
(9.08%) 0.3014*Pr 1 s(0.87%)p 0.48(0.42%)d56.78(49.51%) f56.19(48.99%)g 0.25(0.22%)
(90.92%) 0.9535* O 12 s(8.81%)p10.35(91.18%)d 0.00(0.01%)
(0.96069) BD (2)Pr 1- O 12
(6.56%) 0.2561*Pr 1 s(0.03%)p 8.45(0.24%)d99.99(42.04%) f99.99(57.43%)g 9.00(0.26%)
(93.44%) 0.9667* O 12 s(0.19%)p99.99(99.79%)d 0.09(0.02%)

Table S29. NBO Second order perturbation analysis (Alpha molecular orbital) for **5K-Pr^{Ph}** complex, dispersion

Donor NBO	Acceptor NBO	E(2) kcal/mol
(0.94184) LP (1) O 8<br><br>s(34.98%)p 1.86(65.00%)d 0.00(0.03%)	(0.08523) LV (1)Pr 1<br><br>s(0.12%)p 0.63(0.08%)d99.99(52.84%) f99.99(46.78%)g 1.54(0.19%)	5.89
(0.93805) LP (1) O 9<br><br>s(41.20%)p 1.43(58.77%)d 0.00(0.03%)	(0.08523) LV (1)Pr 1<br><br>s(0.12%)p 0.63(0.08%)d99.99(52.84%) f99.99(46.78%)g 1.54(0.19%)	6.49
(0.91115) LP (2) O 9<br><br>s(0.59%)p99.99(99.38%)d 0.06(0.03%)	(0.08523) LV (1)Pr 1<br><br>s(0.12%)p 0.63(0.08%)d99.99(52.84%) f99.99(46.78%)g 1.54(0.19%)	7.83
(0.91985) LP (1) O 10<br><br>s(40.05%)p 1.50(59.92%)d 0.00(0.03%)	(0.08523) LV (1)Pr 1<br><br>s(0.12%)p 0.63(0.08%)d99.99(52.84%) f99.99(46.78%)g 1.54(0.19%)	11.32
(0.91808) LP (1) O 11<br><br>s(42.70%)p 1.34(57.28%)d 0.00(0.02%)	(0.08523) LV (1)Pr 1<br><br>s(0.12%)p 0.63(0.08%)d99.99(52.84%) f99.99(46.78%)g 1.54(0.19%)	5.97

Figure S78. Computed MOs for **5K-Pr^{Ph}** complex, (s=1/2), Alpha MOs.

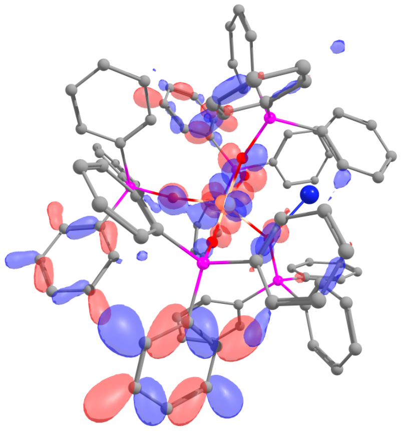

HOMO-52

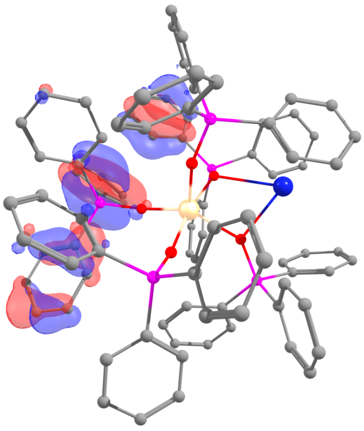

HOMO

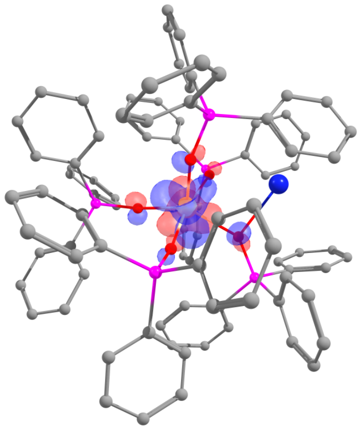

LUMO

Table S30. Selected structural parameters comparison between DFT optimized and X-ray structures for **5Cs-Pr^{Ph}** complex, (s=1/2)

Atom labels	DFT		X-ray	
	No dispersion	dispersion		
Pr1-O8	2.14	2.12	2.18	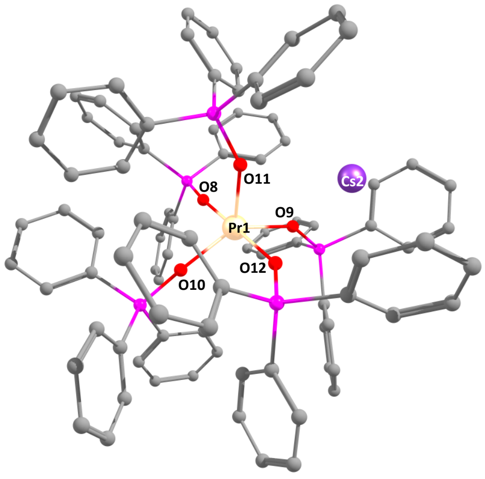
Pr1-O9	2.15	2.16	2.16	
Pr1-O10	2.14	2.12	2.17	
Pr1-O11	2.20	2.16	2.18	
Pr1-O12	2.21	2.19	2.23	

Table S31. Computed natural charges for selected atoms in **5Cs-Pr^{Ph}** complex, dispersion.

Atom labels	Natural charges
Pr1	1.89338
Cs2	0.99945
O8	-1.13817
O9	-1.18579
O10	-1.14662
O11	-1.19327
O12	-1.17140

Table S32. Computed Wiberg bond index between selected atoms in **5Cs-Pr^{Ph}** complex, dispersion.

Atom labels	Wiberg bond index
Pr1-O8	0.7606
Pr1-O9	0.6479
Pr1-O10	0.7503
Pr1-O11	0.6497
Pr1-O12	0.6541

Bonding orbitals (Alpha molecular orbital) between praseodymium and oxygen atoms in the first coordination sphere of **5Cs-Pr^{Ph}** complex, dispersion

(0.97744) BD (1)Pr 1- O 8
(7.59%) 0.2754*Pr 1 s(1.64%)p 1.43(2.34%)d27.90(45.71%) f29.02(47.55%)g 1.68(2.76%)
(92.41%) 0.9613* O 8 s(45.33%)p 1.21(54.67%)d 0.00(0.00%)
(0.95446) BD (2)Pr 1- O 8
(6.27%) 0.2504*Pr 1 s(1.37%)p 1.37(1.88%)d31.73(43.63%) f37.00(50.86%)g 1.64(2.25%)
(93.73%) 0.9681* O 8 s(0.00%)p 1.00(99.99%)d 0.00(0.01%)
(0.97499) BD (1)Pr 1- O 9
(5.70%) 0.2388*Pr 1 s(7.84%)p 0.51(4.00%)d 5.22(40.93%) f 5.50(43.10%)g 0.53(4.13%)
(94.30%) 0.9711* O 9 s(48.78%)p 1.05(51.21%)d 0.00(0.02%)
(0.94885) BD (2)Pr 1- O 9
(5.10%) 0.2258*Pr 1 s(1.05%)p 1.99(2.09%)d46.53(48.79%) f42.87(44.95%)g 2.97(3.12%)
(94.90%) 0.9742* O 9 s(0.02%)p99.99(99.96%)d 1.61(0.03%)
(0.97804) BD (1)Pr 1- O 10
(6.89%) 0.2625*Pr 1 s(1.96%)p 1.27(2.50%)d21.07(41.26%) f25.63(50.18%)g 2.10(4.11%)
(93.11%) 0.9649* O 10 s(48.33%)p 1.07(51.66%)d 0.00(0.01%)
(0.97488) BD (1)Pr 1- O 11
(5.72%) 0.2391*Pr 1 s(7.66%)p 0.40(3.05%)d 6.17(47.32%) f 4.99(38.22%)g 0.49(3.74%)
(94.28%) 0.9710* O 11 s(49.55%)p 1.02(50.44%)d 0.00(0.02%)
(0.97471) BD (1)Pr 1- O 12
(6.03%) 0.2455*Pr 1 s(8.15%)p 0.27(2.21%)d 5.28(42.98%) f 5.47(44.52%)g 0.26(2.14%)
(93.97%) 0.9694* O 12 s(47.55%)p 1.10(52.44%)d 0.00(0.02%)

Table S33. NBO Second order perturbation analysis (Alpha molecular orbital) for **5Cs-Pr^{Ph}** complex, dispersion

Donor NBO	Acceptor NBO	E(2) kcal/mol
(0.90029) LP (1) O 8<br>s(0.11%)p99.99(99.88%)d 0.08(0.01%)	(0.06725) LV (2)Pr 1<br>s(1.20%)p 2.76(3.31%)d28.47(34.14%) f44.88(53.81%)g 6.29(7.54%)	3.63

(0.91070) LP (1) O 9<br><br>s(0.01%)p 1.00(99.97%)d 0.00(0.03%)	(0.05777) LV (4)Pr 1<br><br>s(0.88%)p 5.84(5.11%)d45.18(39.54%) f52.85(46.25%)g 9.41(8.23%)	3.74
(0.89149) LP (2) O 10<br><br>s(0.00%)p 1.00(99.99%)d 0.00(0.01%)	(0.08010) LV (1)Pr 1<br><br>s(3.57%)p 0.66(2.36%)d11.28(40.31%) f14.21(50.78%)g 0.83(2.98%)	7.95
(0.90217) LP (1) O 12<br><br>s(0.18%)p99.99(99.79%)d 0.16(0.03%)	(0.05834) LV (3)Pr 1<br><br>s(2.22%)p 0.66(1.46%)d19.23(42.62%) f23.32(51.68%)g 0.91(2.03%)	5.32

Figure S79. Computed MOs for **5Cs-Pr^{Ph}** complex, (s=1/2), Alpha MOs.

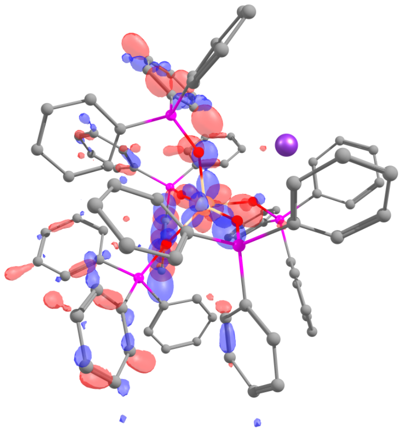

HOMO-52

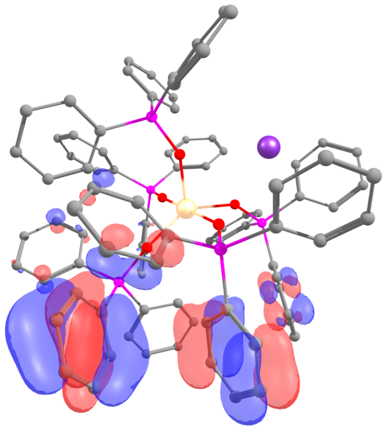

HOMO

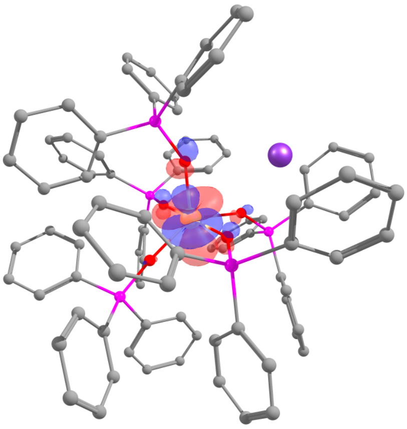

LUMO

Table S34. Selected structural parameters comparison between DFT optimized and X-ray structures for $[Pr(OSi(Ph)_3)_5]^-$ (**[5-Pr^{Ph}]$^-$**), s=1/2

Atom labels	DFT	
	No dispersion	dispersion
Pr1-O7	2.14	2.14
Pr1-O8	2.18	2.15
Pr1-O9	2.17	2.11
Pr1-O10	2.17	2.15
Pr1-O11	2.15	2.14

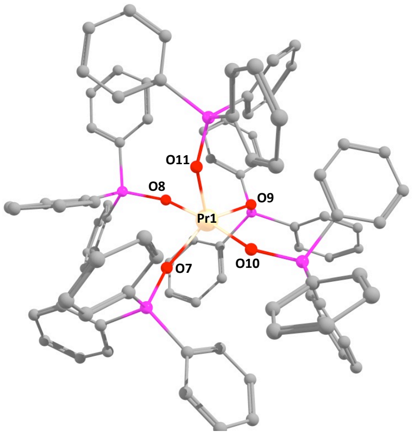

Table S35. Computed natural charges for selected atoms in **[5-Pr^{Ph}]$^-$**, s=1/2, dispersion

Atom labels	Natural charges
Pr1	1.94933
O7	-1.17024
O8	-1.13990
O9	-1.15074
O10	-1.14462
O11	-1.16885

Table S36. Computed Wiberg bond index between selected atoms in **[5-Pr^{Ph}]$^-$**, s=1/2, dispersion

Atom labels	Wiberg bond index
Pr1-O7	0.6565
Pr1-O8	0.6954
Pr1-O9	0.7056
Pr1-O10	0.6909
Pr1-O11	0.6616

Bonding orbitals (Alpha molecular orbital) between praseodymium and oxygen atoms in the first coordination sphere of **[5-Pr^{Ph}]$^{-}$**, s=1/2, dispersion
(0.96438) BD (1)Pr 1- O 7
(6.75%) 0.2599*Pr 1 s(0.05%)p 9.36(0.49%)d99.99(50.04%)f99.99(48.79%)g11.82(0.62%)
(93.25%) 0.9656* O 7 s(16.68%)p 4.99(83.30%)d 0.00(0.02%)
(0.95117) BD (2)Pr 1- O 7
(5.60%) 0.2365*Pr 1 s(0.13%)p 2.14(0.27%)d99.99(53.97%)f99.99(45.15%)g 3.74(0.48%)
(94.40%) 0.9716* O 7 s(0.01%)p99.99(99.96%)d 2.28(0.03%)
(0.96379) BD (1)Pr 1- O 8
(9.61%) 0.3100*Pr 1 s(0.86%)p 0.38(0.33%)d56.09(48.14%)f58.72(50.39%)g 0.34(0.29%)
(90.39%) 0.9507* O 8 s(10.34%)p 8.67(89.64%)d 0.00(0.02%)
(0.96888) BD (1)Pr 1- O 9
(8.67%) 0.2944*Pr 1 s(1.00%)p 0.43(0.43%)d32.85(32.76%)f65.67(65.49%)g 0.33(0.32%)
(91.33%) 0.9557* O 9 s(13.52%)p 6.39(86.46%)d 0.00(0.02%)
(0.95301) BD (2)Pr 1- O 9
(6.19%) 0.2488*Pr 1 s(0.03%)p17.59(0.48%)d99.99(66.20%)f99.99(32.83%)g16.83(0.46%)
(93.81%) 0.9686* O 9 s(0.08%)p99.99(99.88%)d 0.48(0.04%)
(0.96142) BD (1)Pr 1- O 10
(9.25%) 0.3041*Pr 1 s(0.66%)p 0.24(0.16%)d63.73(42.00%)f86.36(56.92%)g 0.40(0.26%)
(90.75%) 0.9526* O 10 s(9.71%)p 9.29(90.27%)d 0.00(0.01%)
(0.95349) BD (2)Pr 1- O 10
(5.42%) 0.2327*Pr 1 s(0.17%)p 1.48(0.26%)d99.99(44.26%)f99.99(54.89%)g 2.42(0.42%)
(94.58%) 0.9725* O 10 s(0.01%)p 1.00(99.97%)d 0.00(0.03%)
(0.96107) BD (1)Pr 1- O 11
(7.34%) 0.2709*Pr 1 s(1.02%)p 0.62(0.63%)d48.57(49.73%)f47.06(48.18%)g 0.43(0.44%)
(92.66%) 0.9626* O 11 s(9.93%)p 9.07(90.05%)d 0.00(0.01%)
(0.95340) BD (2)Pr 1- O 11
(5.44%) 0.2333*Pr 1 s(0.14%)p 2.75(0.39%)d99.99(38.72%)f99.99(60.29%)g 3.27(0.46%)
(94.56%) 0.9724* O 11 s(0.30%)p99.99(99.67%)d 0.10(0.03%)

Tabel S37. NBO Second order perturbation analysis (Alpha molecular orbital) for **[5-Pr^{Ph}]$^{-}$**, s=1/2, dispersion

Donor NBO	Acceptor NBO	E(2) kcal/mol
(0.90073) LP (2) O 8<br>s(0.01%)p 1.00(99.96%)d 0.00(0.04%)	(0.09001) LV (1)Pr 1<br>s(0.09%)p 4.36(0.41%)d99.99(59.35%)f99.99(39.56%)g 6.24(0.59%)	10.92
(0.92971) LP (1) O 9<br>s(42.16%)p 1.37(57.79%)d 0.00(0.04%)	(0.09001) LV (1)Pr 1<br>s(0.09%)p 4.36(0.41%)d99.99(59.35%)f99.99(39.56%)g 6.24(0.59%)	6.40
(0.92776) LP (1) O 10<br>s(43.75%)p 1.28(56.22%)d 0.00(0.03%)	(0.09001) LV (1)Pr 1<br>s(0.09%)p 4.36(0.41%)d99.99(59.35%)f99.99(39.56%)g 6.24(0.59%)	3.55

(0.92776) LP (1) O 10<br><br>s(43.75%)p 1.28(56.22%)d 0.00(0.03%)	(0.03325) LV (2)Pr 1<br><br>s(66.71%)p 0.00(0.08%)d 0.11(7.17%)f 0.39(25.86%)g 0.00(0.19%)	3.18

Figure S80. DFT computed MOs for **[5-Pr^{Ph}]$^{-}$**, s=1/2, dispersion.

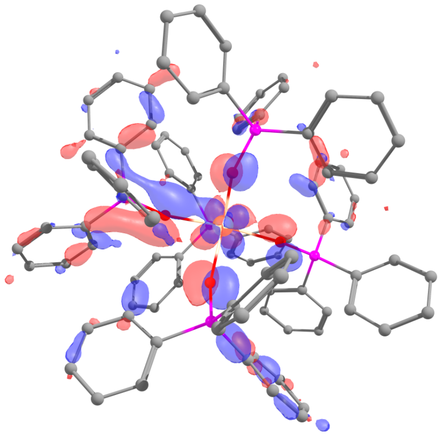<br>HOMO-48	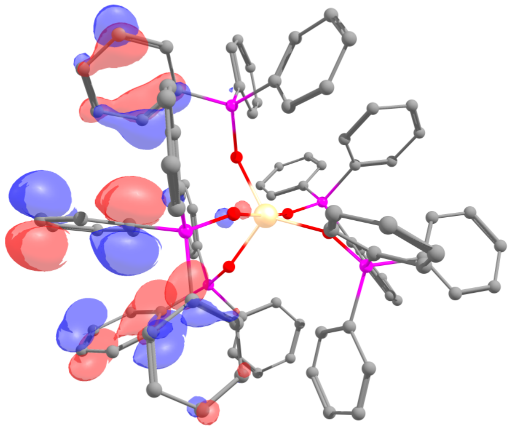<br>HOMO
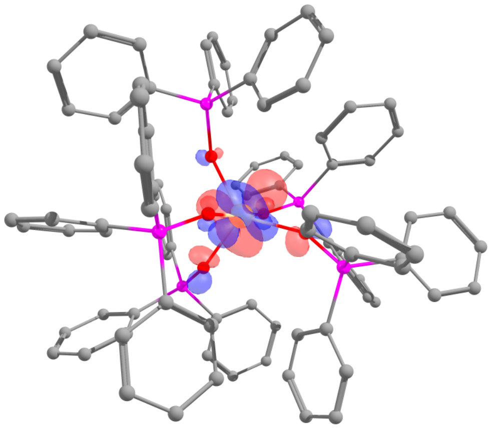<br>LUMO	

Table S38 Computed natural charges for selected atoms in [Pr^{IV}(OSi(Ph)$_3$)$_4$(CH$_3$CN)$_2$], s=1/2, dispersion

Atom labels	Natural charges	
O8	-1.13104	
Pr9	1.90354	
N10	-0.42303	
N25	-0.42719	
O28	-1.13808	
O36	-1.09063	

O44	-1.15449	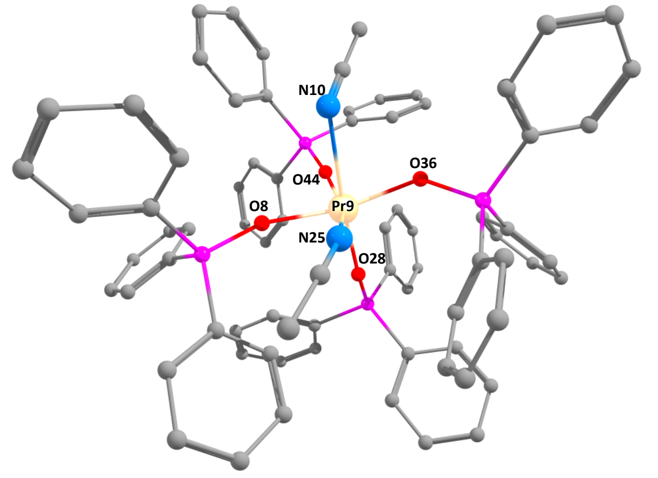

Table S39. Computed Wiberg bond index between selected atoms in [$Pr^{IV}(OSi(Ph)_3)_4(CH_3CN)_2$], s=1/2, dispersion

Atom labels	Wiberg bond index
Pr9-O8	0.7657
Pr9-N10	0.1943
Pr9-N25	0.2187
Pr9-O28	0.7659
Pr9-O36	0.8315
Pr9-O44	0.7376

Bonding orbitals (Alpha molecular orbital) between praseodymium and oxygen/nitrogen atoms in the first coordination sphere of [[$Pr^{IV}(OSi(Ph)_3)_4(CH_3CN)_2$], s=1/2, dispersion

(0.97030) BD (1) O 8-Pr 9
(90.94%) 0.9536* O 8 s(14.32%)p 5.98(85.68%)d 0.00(0.01%)
(9.06%) 0.3010*Pr 9 s(0.10%)p 3.44(0.35%)d99.99(41.10%)f99.99(58.18%)g 2.61(0.27%)
(0.95848) BD (2) O 8-Pr 9
(93.43%) 0.9666* O 8 s(0.01%)p99.99(99.97%)d 1.32(0.02%)
(6.57%) 0.2562*Pr 9 s(0.03%)p 4.80(0.17%)d99.99(43.86%)f99.99(55.57%)g10.74(0.37%)
(0.96682) BD (1)Pr 9- O 28
(8.96%) 0.2993*Pr 9 s(0.07%)p 4.45(0.33%)d99.99(49.04%)f99.99(50.34%)g 2.87(0.21%)
(91.04%) 0.9542* O 28 s(8.43%)p10.86(91.56%)d 0.00(0.01%)
(0.95613) BD (2)Pr 9- O 28
(6.25%) 0.2500*Pr 9 s(0.03%)p15.50(0.42%)d99.99(56.24%)f99.99(42.63%)g24.76(0.68%)
(93.75%) 0.9683* O 28 s(0.01%)p 1.00(99.98%)d 0.00(0.01%)
(0.96830) BD (1)Pr 9- O 36
(12.06%) 0.3473*Pr 9 s(0.03%)p 9.63(0.31%)d99.99(47.00%)f99.99(52.41%)g 7.68(0.24%)
(87.94%) 0.9378* O 36 s(10.47%)p 8.55(89.50%)d 0.00(0.04%)
(0.95648) BD (2)Pr 9- O 36

(7.06%) 0.2658*Pr 9 s(0.06%)p 5.84(0.35%)d99.99(43.73%)f99.99(55.41%)g 7.40(0.45%)
(92.94%) 0.9640* O 36 s(0.07%)p99.99(99.89%)d 0.75(0.05%)

Table S40. NBO Second order perturbation analysis (Alpha molecular orbital) for [[$Pr^{IV}(OSi(Ph)_3)_4(CH_3CN)_2$], s=1/2, dispersion

Donor NBO	Acceptor NBO	E(2) kcal/mol
(0.91550) LP (1) O 8<br>s(38.84%)p 1.57(61.14%)d 0.00(0.02%)	(0.09269) LV (3)Pr 9<br>s(0.10%)p 0.54(0.05%)d99.99(62.97%)f99.99(36.75%)g 1.21(0.12%)	6.06
(0.91550) LP (1) O 8<br>s(38.84%)p 1.57(61.14%)d 0.00(0.02%)	(0.03957) LV (5)Pr 9<br>s(12.29%)p 0.08(0.92%)d 1.47(18.03%)f 5.48(67.37%)g 0.11(1.39%)	8.47
(0.91872) LP (1) O 28<br>s(42.08%)p 1.38(57.90%)d 0.00(0.02%)	(0.09806) LV (2)Pr 9<br>s(0.02%)p 7.45(0.12%)d99.99(59.43%)f99.99(40.25%)g11.55(0.18%)	8.74
(0.91872) LP (1) O 28<br>s(42.08%)p 1.38(57.90%)d 0.00(0.02%)	(0.09269) LV (3)Pr 9<br>s(0.10%)p 0.54(0.05%)d99.99(62.97%)f99.99(36.75%)g 1.21(0.12%)	19.70
(0.93076) LP (1) O 36<br>s(50.39%)p 0.98(49.57%)d 0.00(0.04%)	(0.05647) LV (4)Pr 9<br>s(6.00%)p 0.01(0.06%)d 5.67(33.97%)f 9.92(59.45%)g 0.09(0.52%)	11.13
(0.94103) LP (1) N 10<br>s(50.04%)p 1.00(49.90%)d 0.00(0.05%)	(0.09269) LV (3)Pr 9<br>s(0.10%)p 0.54(0.05%)d99.99(62.97%)f99.99(36.75%)g 1.21(0.12%)	17.39
(0.93277) LP (1) N 25<br>s(47.70%)p 1.10(52.27%)d 0.00(0.04%)	(0.12336) LV (1)Pr 9<br>s(0.22%)p 2.88(0.64%)d99.99(49.45%)f99.99(49.11%)g 2.53(0.56%)	5.39
(0.93277) LP (1) N 25<br>s(47.70%)p 1.10(52.27%)d 0.00(0.04%)	(0.09269) LV (3)Pr 9<br>s(0.10%)p 0.54(0.05%)d99.99(62.97%)f99.99(36.75%)g 1.21(0.12%)	6.34
(0.91852) LP (1) O 44<br>s(34.88%)p 1.87(65.11%)d 0.00(0.02%)	(0.12336) LV (1)Pr 9<br>s(0.22%)p 2.88(0.64%)d99.99(49.45%)f99.99(49.11%)g 2.53(0.56%)	21.33
(0.89978) LP (2) O 44	(0.12336) LV (1)Pr 9	34.43

s(14.61%)p 5.85(85.39%)d 0.00(0.00%)	s(0.22%)p 2.88(0.64%)d99.99(49.45%)f99.99(49.11%)g 2.53(0.56%)	
(0.89409) LP (3) O 44<br><br>s(0.00%)p 1.00(99.99%)d 0.00(0.01%)	(0.09806) LV (2)Pr 9<br><br>s(0.02%)p 7.45(0.12%)d99.99(59.43%)f99.99(40.25%)g11.55(0.18%)	17.79

Figure S81. DFT computed MOs for [$Pr^{IV}(OSi(Ph)_3)_4(CH_3CN)_2$], s=1/2, dispersion.

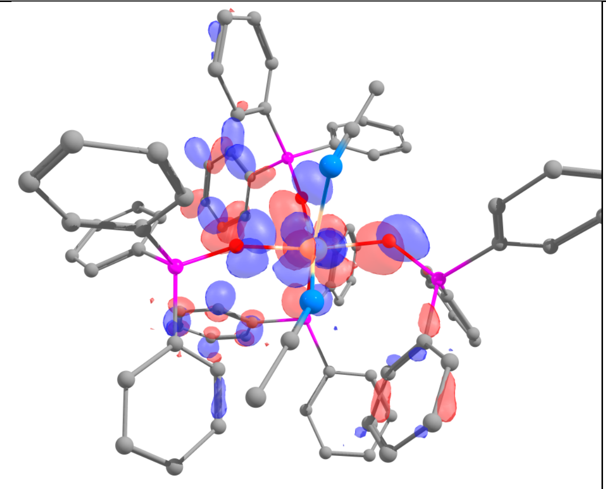

HOMO-39

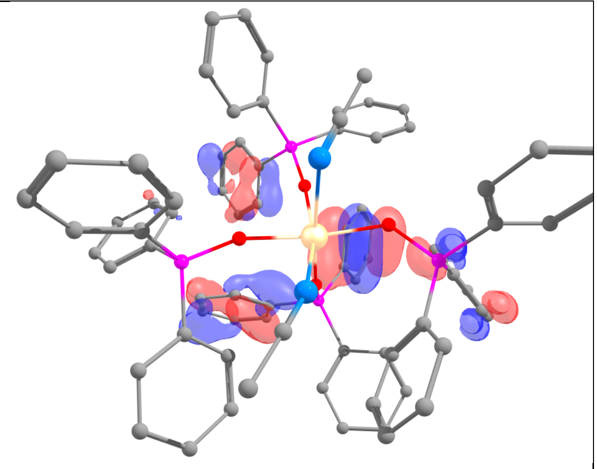

HOMO

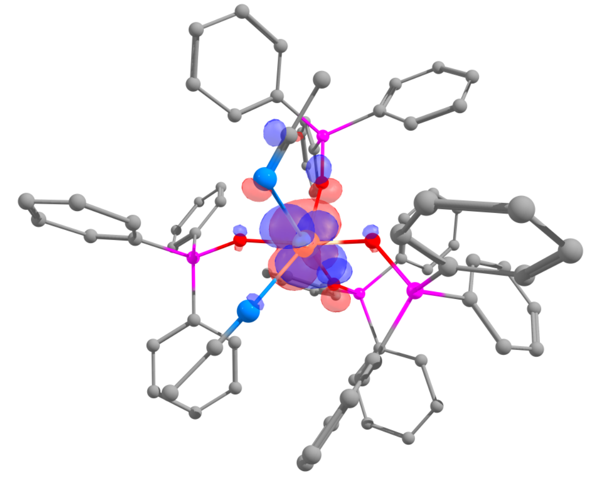

LUMO

Optimized geometries

Ce[OSi(O^{t}Bu)$_3$]$_4$ complex (s=0), dispersion

Ce	-0.047729000	-0.225669000	0.096325000
Si	-1.546072000	-3.197461000	-0.716035000
Si	0.357288000	0.353659000	3.132255000
Si	-2.105481000	2.468650000	-1.142681000
Si	3.346608000	0.280043000	-1.179037000
O	-0.325102000	-2.097074000	-0.847810000
O	-2.478771000	-3.322688000	-2.079243000
O	-0.983594000	-4.719441000	-0.372699000
O	-2.516696000	-2.567676000	0.478842000
O	-0.183127000	-0.850488000	2.161169000
O	1.725686000	0.017331000	3.996054000
O	-0.728062000	0.999933000	4.190331000
O	0.792995000	1.403420000	1.867813000
O	-1.616777000	1.021155000	-0.525594000
O	-2.331945000	3.450966000	0.178058000
O	-0.945845000	3.195782000	-2.074050000
O	-3.453502000	2.296340000	-2.097034000
O	1.717271000	0.168888000	-0.969234000
O	3.591622000	1.803842000	-1.780855000
O	3.922536000	-0.928422000	-2.161652000
O	4.203291000	0.175105000	0.239784000
C	-2.028361000	-3.509958000	-3.431988000
C	-1.526419000	-2.170825000	-3.967589000
H	-1.207898000	-2.265451000	-5.010640000
H	-2.319417000	-1.422041000	-3.913073000
H	-0.679389000	-1.818628000	-3.375518000
C	-3.262546000	-3.971210000	-4.200134000
H	-3.034074000	-4.104854000	-5.261954000
H	-3.625886000	-4.921157000	-3.797529000
H	-4.059805000	-3.228307000	-4.104867000
C	-0.925226000	-4.563204000	-3.497715000
H	-0.646166000	-4.750786000	-4.539569000
H	-0.033914000	-4.225529000	-2.962445000
H	-1.259147000	-5.499611000	-3.044883000
C	0.091216000	-5.083714000	0.507152000
C	1.409580000	-4.836922000	-0.221301000
H	2.263216000	-5.119158000	0.402862000
H	1.443142000	-5.422624000	-1.144589000
H	1.496695000	-3.780866000	-0.480771000
C	-0.103469000	-6.572511000	0.779458000
H	0.698686000	-6.960018000	1.415125000
H	-1.060432000	-6.745303000	1.280240000
H	-0.104074000	-7.128424000	-0.162318000
C	0.032290000	-4.292495000	1.812574000
H	0.837794000	-4.615078000	2.480469000

H	0.139562000	-3.218163000	1.649500000
H	-0.920517000	-4.457139000	2.322735000
C	-3.786893000	-3.075789000	0.928078000
C	-3.826858000	-4.601669000	0.875736000
H	-4.791152000	-4.957850000	1.251455000
H	-3.704299000	-4.962559000	-0.149096000
H	-3.033122000	-5.036264000	1.487048000
C	-3.934261000	-2.587184000	2.363686000
H	-4.878622000	-2.929761000	2.797677000
H	-3.108270000	-2.961137000	2.974993000
H	-3.915043000	-1.495493000	2.392304000
C	-4.876114000	-2.488034000	0.037566000
H	-5.867465000	-2.811705000	0.371094000
H	-4.834209000	-1.398082000	0.072791000
H	-4.724440000	-2.807005000	-0.996134000
C	1.759403000	-0.744144000	5.225861000
C	3.166823000	-1.319783000	5.318115000
H	3.286572000	-1.884191000	6.247832000
H	3.359438000	-1.988706000	4.475516000
H	3.910025000	-0.518555000	5.298798000
C	0.732804000	-1.874020000	5.179567000
H	0.817537000	-2.499755000	6.073285000
H	-0.284972000	-1.476997000	5.144225000
H	0.891746000	-2.499423000	4.297277000
C	1.484809000	0.208737000	6.385589000
H	1.544366000	-0.320860000	7.341754000
H	2.224144000	1.015153000	6.389874000
H	0.491075000	0.649456000	6.283406000
C	-2.162294000	1.138400000	4.129325000
C	-2.756127000	-0.055580000	4.869567000
H	-3.848495000	0.005804000	4.895630000
H	-2.477485000	-0.983785000	4.364733000
H	-2.385448000	-0.088919000	5.898433000
C	-2.477528000	2.442227000	4.854675000
H	-3.558956000	2.595949000	4.916118000
H	-2.068709000	2.420437000	5.868982000
H	-2.037328000	3.289179000	4.323151000
C	-2.664701000	1.188660000	2.692349000
H	-3.742469000	1.376711000	2.682350000
H	-2.190744000	1.987010000	2.117433000
H	-2.489295000	0.235613000	2.188189000
C	1.380042000	2.740983000	1.901211000
C	2.808340000	2.620037000	2.409144000
H	3.291001000	3.602044000	2.393746000
H	2.827816000	2.233242000	3.430859000
H	3.373410000	1.942971000	1.765592000
C	0.520138000	3.615786000	2.802491000

H	0.902245000	4.641008000	2.796732000
H	-0.508122000	3.624318000	2.432764000
H	0.521301000	3.251220000	3.832575000
C	1.346066000	3.244234000	0.468146000
H	1.733447000	4.266336000	0.424061000
H	1.959794000	2.624303000	-0.186877000
H	0.324694000	3.262930000	0.086175000
C	-2.664689000	4.851256000	0.188090000
C	-3.297506000	5.100157000	1.553724000
H	-3.568105000	6.153752000	1.671901000
H	-4.198596000	4.491040000	1.670182000
H	-2.598301000	4.830612000	2.350119000
C	-1.381656000	5.662843000	0.024747000
H	-1.602180000	6.735068000	0.036017000
H	-0.686490000	5.444753000	0.838665000
H	-0.897423000	5.407538000	-0.920453000
C	-3.651058000	5.195140000	-0.925847000
H	-3.914642000	6.256112000	-0.874716000
H	-3.217001000	4.995539000	-1.908995000
H	-4.566423000	4.606148000	-0.831444000
C	-0.544054000	2.831361000	-3.408139000
C	0.914643000	3.256093000	-3.523944000
H	1.295024000	3.058142000	-4.531033000
H	1.010042000	4.327370000	-3.322366000
H	1.533660000	2.710887000	-2.807495000
C	-1.433885000	3.597842000	-4.382501000
H	-1.142554000	3.397542000	-5.418569000
H	-2.477384000	3.304244000	-4.243134000
H	-1.346015000	4.673163000	-4.199885000
C	-0.675140000	1.326056000	-3.627007000
H	-0.360101000	1.060220000	-4.640143000
H	-0.037448000	0.782038000	-2.925610000
H	-1.708886000	0.999565000	-3.494944000
C	-4.593429000	1.455446000	-1.853897000
C	-4.294448000	0.075115000	-2.431383000
H	-5.159351000	-0.587256000	-2.334765000
H	-4.049949000	0.166898000	-3.494020000
H	-3.451082000	-0.387981000	-1.914633000
C	-4.895449000	1.370560000	-0.359475000
H	-5.799047000	0.777954000	-0.187848000
H	-4.065599000	0.897587000	0.171862000
H	-5.050273000	2.368007000	0.060660000
C	-5.750122000	2.113070000	-2.599840000
H	-6.661975000	1.515274000	-2.506985000
H	-5.944286000	3.111685000	-2.198545000
H	-5.504377000	2.212954000	-3.660916000
C	4.834440000	2.513924000	-1.915335000

C	5.133722000	3.206313000	-0.588335000
H	6.047686000	3.804694000	-0.660370000
H	4.305845000	3.866390000	-0.315929000
H	5.257398000	2.461888000	0.200985000
C	4.596783000	3.537542000	-3.020810000
H	5.489037000	4.150671000	-3.180198000
H	4.347217000	3.032178000	-3.958231000
H	3.763484000	4.192979000	-2.754382000
C	5.969215000	1.565849000	-2.296808000
H	6.897595000	2.128270000	-2.436977000
H	6.132874000	0.822027000	-1.512753000
H	5.738166000	1.038528000	-3.225773000
C	3.371285000	-1.393274000	-3.404747000
C	2.745844000	-0.241578000	-4.186637000
H	2.344802000	-0.602694000	-5.138701000
H	1.926629000	0.206162000	-3.621163000
H	3.484489000	0.537546000	-4.391639000
C	4.547531000	-1.986926000	-4.173976000
H	4.215445000	-2.402327000	-5.130428000
H	5.301595000	-1.219211000	-4.369968000
H	5.015040000	-2.785691000	-3.591090000
C	2.333335000	-2.465786000	-3.091113000
H	1.903549000	-2.872806000	-4.011745000
H	2.798529000	-3.286409000	-2.537488000
H	1.524655000	-2.058375000	-2.480211000
C	4.499313000	-1.005120000	1.008576000
C	5.609204000	-1.790423000	0.314136000
H	5.887092000	-2.666208000	0.909311000
H	5.284746000	-2.115446000	-0.675546000
H	6.495268000	-1.158814000	0.198001000
C	3.240822000	-1.855664000	1.164742000
H	3.454076000	-2.763688000	1.736662000
H	2.467886000	-1.303458000	1.706829000
H	2.860772000	-2.163512000	0.186351000
C	4.972080000	-0.492982000	2.363514000
H	5.302375000	-1.323724000	2.994053000
H	5.812567000	0.194005000	2.228044000
H	4.162305000	0.030233000	2.875239000

Ce[OSi(O^{t}Bu)$_3$]$_4$ complex (s=0)

Ce	0.021963000	-0.107829000	0.097215000
Si	-1.906833000	-3.058596000	-0.953566000
Si	0.546992000	-0.066819000	3.229493000
Si	-2.067058000	2.892514000	-0.770762000
Si	3.479693000	0.155403000	-1.351569000
O	-0.857013000	-1.777558000	-0.837811000
O	-2.803944000	-3.057009000	-2.353703000

O	-1.111183000	-4.520253000	-0.917618000
O	-2.988309000	-2.866394000	0.295053000
O	-0.064097000	-1.032599000	2.048061000
O	1.954806000	-0.571518000	3.938377000
O	-0.497139000	0.328564000	4.450021000
O	0.940834000	1.225469000	2.187462000
O	-1.292656000	1.464724000	-0.435699000
O	-2.330053000	3.602615000	0.714605000
O	-1.144994000	3.968461000	-1.640006000
O	-3.451643000	2.665015000	-1.665257000
O	1.913179000	-0.052714000	-0.851107000
O	3.580485000	1.751109000	-1.817879000
O	3.904026000	-0.920964000	-2.550792000
O	4.595968000	-0.058622000	-0.135514000
C	-2.470909000	-3.193823000	-3.743583000
C	-1.555800000	-2.045937000	-4.170578000
H	-1.346378000	-2.100206000	-5.244212000
H	-2.028807000	-1.083154000	-3.958649000
H	-0.605568000	-2.085166000	-3.632699000
C	-3.806541000	-3.109085000	-4.485050000
H	-3.658199000	-3.200299000	-5.565859000
H	-4.473581000	-3.912910000	-4.159039000
H	-4.295443000	-2.151971000	-4.281400000
C	-1.808317000	-4.547022000	-4.005637000
H	-1.610254000	-4.671267000	-5.075669000
H	-0.866437000	-4.633196000	-3.459692000
H	-2.461549000	-5.361009000	-3.678162000
C	-0.174872000	-5.097816000	0.005341000
C	1.234519000	-4.685142000	-0.417301000
H	1.987044000	-5.144021000	0.232440000
H	1.429264000	-4.999188000	-1.446868000
H	1.347227000	-3.599798000	-0.362159000
C	-0.345762000	-6.612229000	-0.133158000
H	0.364685000	-7.143803000	0.508276000
H	-1.359187000	-6.911804000	0.149582000
H	-0.176519000	-6.920594000	-1.169119000
C	-0.453981000	-4.660394000	1.443565000
H	0.252450000	-5.148224000	2.124046000
H	-0.350079000	-3.578181000	1.564507000
H	-1.465934000	-4.942461000	1.745761000
C	-4.267121000	-3.486793000	0.517451000
C	-4.282272000	-4.943141000	0.047852000
H	-5.255473000	-5.396339000	0.264398000
H	-4.107507000	-5.007196000	-1.029493000
H	-3.512288000	-5.527446000	0.558477000
C	-4.501409000	-3.415922000	2.026963000
H	-5.479147000	-3.834045000	2.287864000

H	-3.730268000	-3.977301000	2.562855000
H	-4.466019000	-2.376991000	2.366997000
C	-5.333022000	-2.680718000	-0.225989000
H	-6.330954000	-3.094843000	-0.045216000
H	-5.322139000	-1.641700000	0.114671000
H	-5.131953000	-2.696298000	-1.299593000
C	2.163632000	-1.413784000	5.093986000
C	3.604187000	-1.910025000	4.982616000
H	3.856757000	-2.547514000	5.835929000
H	3.741090000	-2.488071000	4.064500000
H	4.300120000	-1.066391000	4.965001000
C	1.194566000	-2.595471000	5.072714000
H	1.395887000	-3.269635000	5.911576000
H	0.159882000	-2.252397000	5.156490000
H	1.300536000	-3.160756000	4.142380000
C	1.984243000	-0.573001000	6.358429000
H	2.177127000	-1.176350000	7.251741000
H	2.684451000	0.268298000	6.356446000
H	0.967533000	-0.177699000	6.412122000
C	-1.934074000	0.448135000	4.535063000
C	-2.479810000	-0.882657000	5.052781000
H	-3.565510000	-0.829384000	5.184204000
H	-2.260382000	-1.686234000	4.343760000
H	-2.030567000	-1.132392000	6.018963000
C	-2.192957000	1.565214000	5.545927000
H	-3.266809000	1.688501000	5.717791000
H	-1.714232000	1.333138000	6.502063000
H	-1.789915000	2.514377000	5.182174000
C	-2.556246000	0.788178000	3.182841000
H	-3.630164000	0.964576000	3.301451000
H	-2.118883000	1.691444000	2.748160000
H	-2.431157000	-0.043732000	2.484080000
C	1.668069000	2.470291000	2.422290000
C	3.134100000	2.146027000	2.692530000
H	3.702689000	3.073762000	2.815386000
H	3.240391000	1.547065000	3.599440000
H	3.561860000	1.587339000	1.856194000
C	1.030242000	3.200395000	3.601371000
H	1.543969000	4.151895000	3.772060000
H	-0.023577000	3.409096000	3.397165000
H	1.095607000	2.608856000	4.518996000
C	1.519330000	3.279005000	1.138948000
H	2.012668000	4.250202000	1.247892000
H	1.986705000	2.761986000	0.294767000
H	0.465209000	3.456288000	0.913952000
C	-2.743431000	4.947317000	1.029089000
C	-3.375137000	4.867759000	2.419418000

H	-3.692550000	5.859165000	2.758419000
H	-4.248871000	4.209293000	2.408422000
H	-2.656957000	4.470215000	3.142489000
C	-1.507716000	5.848916000	1.055512000
H	-1.784614000	6.876205000	1.315852000
H	-0.789863000	5.489897000	1.798652000
H	-1.021912000	5.850106000	0.077040000
C	-3.763913000	5.476728000	0.020077000
H	-4.080403000	6.486047000	0.303352000
H	-3.334137000	5.522560000	-0.983688000
H	-4.650386000	4.837741000	-0.013262000
C	-0.841148000	4.043475000	-3.044486000
C	0.341952000	5.005456000	-3.146900000
H	0.653817000	5.130242000	-4.188732000
H	0.069288000	5.987196000	-2.747711000
H	1.188857000	4.622027000	-2.572465000
C	-2.047711000	4.605019000	-3.798957000
H	-1.820617000	4.702517000	-4.866009000
H	-2.913088000	3.950782000	-3.677022000
H	-2.306508000	5.596943000	-3.414964000
C	-0.452813000	2.665723000	-3.580727000
H	-0.186493000	2.728874000	-4.640987000
H	0.410697000	2.274823000	-3.035028000
H	-1.284694000	1.961305000	-3.483392000
C	-4.567590000	1.771905000	-1.527026000
C	-4.260793000	0.500360000	-2.315566000
H	-5.114975000	-0.183386000	-2.301368000
H	-4.038839000	0.753085000	-3.357289000
H	-3.400556000	-0.023301000	-1.892361000
C	-4.839526000	1.450313000	-0.058049000
H	-5.725586000	0.813883000	0.033127000
H	-3.992266000	0.920413000	0.386490000
H	-5.013918000	2.364823000	0.515285000
C	-5.762453000	2.503175000	-2.142671000
H	-6.658893000	1.875433000	-2.113070000
H	-5.970437000	3.429806000	-1.599814000
H	-5.554621000	2.759103000	-3.185916000
C	4.748152000	2.556478000	-2.077603000
C	5.303022000	3.067834000	-0.747134000
H	6.167723000	3.719141000	-0.914647000
H	4.538993000	3.642049000	-0.215138000
H	5.607800000	2.229830000	-0.116758000
C	4.259184000	3.725606000	-2.931742000
H	5.087002000	4.399650000	-3.174934000
H	3.821392000	3.362956000	-3.866539000
H	3.496985000	4.296775000	-2.394999000
C	5.815039000	1.765554000	-2.836304000

H	6.678247000	2.405700000	-3.046775000
H	6.159975000	0.909168000	-2.251557000
H	5.422511000	1.397672000	-3.788274000
C	3.243340000	-1.328997000	-3.759755000
C	2.367472000	-0.209500000	-4.321342000
H	1.909320000	-0.521661000	-5.265345000
H	1.565012000	0.044059000	-3.623123000
H	2.959240000	0.691217000	-4.505094000
C	4.357494000	-1.678573000	-4.748252000
H	3.938085000	-2.048469000	-5.689432000
H	4.971333000	-0.799526000	-4.965074000
H	5.006880000	-2.454158000	-4.331350000
C	2.406435000	-2.567594000	-3.443422000
H	1.912012000	-2.947463000	-4.343435000
H	3.043715000	-3.360879000	-3.040984000
H	1.641636000	-2.330816000	-2.699267000
C	5.206156000	-1.250261000	0.394870000
C	6.319087000	-1.707767000	-0.549918000
H	6.819795000	-2.596535000	-0.151153000
H	5.909488000	-1.943327000	-1.534530000
H	7.068510000	-0.917881000	-0.663406000
C	4.160318000	-2.349055000	0.577282000
H	4.615633000	-3.242313000	1.017887000
H	3.359030000	-2.009710000	1.240234000
H	3.728080000	-2.630934000	-0.386844000
C	5.791947000	-0.842093000	1.746250000
H	6.313222000	-1.685265000	2.211102000
H	6.507046000	-0.023537000	1.619320000
H	4.999496000	-0.508300000	2.420910000

KCe[OSi(O^{t}Bu)$_3$]$_4$ complex (s=1/2), dispersion

Ce	-0.721861000	0.214033000	-0.530479000
K	2.502176000	-0.413574000	0.816462000
Si	0.136425000	-2.717596000	1.737522000
Si	2.349438000	-0.482326000	-2.531183000
Si	1.209934000	2.631733000	1.697746000
Si	-3.877594000	0.517200000	-0.777410000
O	1.779578000	-2.618365000	2.104891000
O	-0.108766000	-4.269363000	1.197959000
O	-0.812294000	-2.564820000	3.096060000
O	-0.078867000	-1.560440000	0.625762000
O	0.949889000	0.176713000	-2.072850000
O	3.585783000	0.248803000	-1.636357000
O	2.620748000	-0.387677000	-4.173847000
O	2.571347000	-2.076008000	-2.069588000
O	0.554287000	1.517521000	0.729468000
O	2.664259000	1.916722000	2.167205000

O	1.435947000	4.109904000	0.960259000
O	0.403290000	2.958333000	3.111902000
O	-2.746552000	0.998935000	0.262533000
O	-2.876420000	-0.099020000	-2.026936000
O	-4.824925000	-0.782909000	-0.371532000
O	-4.901559000	1.695208000	-1.354379000
C	2.573901000	-3.508898000	2.905524000
C	3.798456000	-2.693613000	3.318578000
H	4.469042000	-3.284256000	3.949324000
H	4.364867000	-2.378688000	2.434712000
H	3.492945000	-1.804108000	3.879166000
C	2.987740000	-4.701262000	2.048053000
H	3.624843000	-5.385067000	2.618081000
H	2.100423000	-5.237928000	1.705736000
H	3.542299000	-4.359553000	1.169826000
C	1.807487000	-3.972108000	4.141005000
H	2.436766000	-4.632382000	4.745904000
H	1.509615000	-3.118292000	4.754068000
H	0.904372000	-4.517366000	3.857626000
C	-1.389348000	-4.886362000	0.973354000
C	-1.825433000	-5.551545000	2.276099000
H	-2.780814000	-6.071085000	2.149205000
H	-1.073702000	-6.280711000	2.593846000
H	-1.931101000	-4.795701000	3.057790000
C	-2.422343000	-3.857463000	0.516846000
H	-3.376275000	-4.345124000	0.295873000
H	-2.598292000	-3.105661000	1.290259000
H	-2.081903000	-3.349694000	-0.387197000
C	-1.154036000	-5.920521000	-0.122574000
H	-2.070775000	-6.479464000	-0.334103000
H	-0.824920000	-5.425899000	-1.040401000
H	-0.376553000	-6.625878000	0.184933000
C	-1.125113000	-1.371014000	3.832076000
C	-1.335927000	-1.824105000	5.274517000
H	-1.632574000	-0.980512000	5.905185000
H	-2.118596000	-2.586953000	5.319389000
H	-0.416175000	-2.254521000	5.680023000
C	-2.417609000	-0.788420000	3.268097000
H	-2.739943000	0.078861000	3.853120000
H	-2.300365000	-0.463228000	2.232187000
H	-3.210551000	-1.541727000	3.307969000
C	0.012134000	-0.357990000	3.753926000
H	-0.187779000	0.506004000	4.390354000
H	0.951078000	-0.815044000	4.077713000
H	0.133560000	0.021470000	2.737123000
C	4.989903000	-0.071519000	-1.675535000
C	5.293021000	-1.100699000	-0.581406000

H	6.333758000	-1.432567000	-0.641099000
H	5.162429000	-0.666538000	0.416962000
H	4.643487000	-1.972088000	-0.702327000
C	5.720132000	1.236370000	-1.388789000
H	6.801781000	1.076502000	-1.340244000
H	5.507906000	1.968708000	-2.171904000
H	5.384847000	1.654822000	-0.436053000
C	5.419985000	-0.634005000	-3.028131000
H	6.497861000	-0.823805000	-3.020008000
H	4.902514000	-1.570993000	-3.244537000
H	5.196032000	0.068880000	-3.831702000
C	2.360898000	0.772189000	-4.983185000
C	2.786228000	2.046690000	-4.255317000
H	2.603919000	2.922857000	-4.885691000
H	2.215648000	2.162700000	-3.330116000
H	3.849419000	2.017261000	-4.003589000
C	3.181191000	0.571806000	-6.253311000
H	2.893714000	-0.363506000	-6.742010000
H	3.021083000	1.396474000	-6.954704000
H	4.247680000	0.518746000	-6.016117000
C	0.869634000	0.824513000	-5.300358000
H	0.551625000	-0.111847000	-5.768095000
H	0.306106000	0.961963000	-4.375970000
H	0.643505000	1.649732000	-5.983526000
C	1.926058000	-3.256460000	-2.569460000
C	2.526701000	-3.606781000	-3.928629000
H	2.071617000	-4.518128000	-4.329823000
H	2.373291000	-2.787881000	-4.635146000
H	3.603579000	-3.775616000	-3.829427000
C	0.421471000	-3.029777000	-2.675161000
H	-0.081894000	-3.940349000	-3.014644000
H	0.025310000	-2.756736000	-1.692491000
H	0.201170000	-2.233990000	-3.391529000
C	2.215386000	-4.345629000	-1.544309000
H	1.810798000	-5.307076000	-1.875417000
H	3.295925000	-4.454237000	-1.409284000
H	1.757956000	-4.097710000	-0.584606000
C	3.543195000	2.323110000	3.227106000
C	4.903878000	1.722582000	2.880210000
H	5.647995000	1.969055000	3.643410000
H	4.836996000	0.630297000	2.819570000
H	5.254903000	2.104405000	1.916985000
C	3.017651000	1.744051000	4.538478000
H	3.687997000	1.986142000	5.369510000
H	2.024501000	2.146114000	4.747353000
H	2.939428000	0.655033000	4.463000000
C	3.638374000	3.844383000	3.307025000

H	4.344018000	4.137674000	4.090521000
H	3.977757000	4.261506000	2.355794000
H	2.662523000	4.276013000	3.540357000
C	1.858071000	4.362478000	-0.386665000
C	2.181710000	5.853382000	-0.423193000
H	2.511585000	6.158361000	-1.421040000
H	1.297326000	6.437741000	-0.152604000
H	2.975197000	6.082646000	0.294044000
C	3.092000000	3.533701000	-0.728068000
H	3.472420000	3.806274000	-1.717175000
H	3.884086000	3.709540000	0.004857000
H	2.861907000	2.466048000	-0.750931000
C	0.720669000	4.036526000	-1.354795000
H	0.979461000	4.346267000	-2.372143000
H	0.538538000	2.959959000	-1.364741000
H	-0.195778000	4.553426000	-1.056464000
C	-0.751846000	3.801822000	3.267315000
C	-0.263656000	5.228630000	3.507394000
H	-1.107926000	5.908455000	3.661863000
H	0.375064000	5.264538000	4.395666000
H	0.315066000	5.570085000	2.645844000
C	-1.488464000	3.264290000	4.490192000
H	-2.353911000	3.890005000	4.729347000
H	-1.839819000	2.246711000	4.299735000
H	-0.821233000	3.246783000	5.357290000
C	-1.653846000	3.735401000	2.037788000
H	-2.545616000	4.349330000	2.198062000
H	-1.131886000	4.126013000	1.159757000
H	-1.972797000	2.711505000	1.826275000
C	-3.281155000	-0.830779000	-3.209679000
C	-2.224080000	-0.520277000	-4.259410000
H	-2.400301000	-1.093866000	-5.174141000
H	-2.235518000	0.544477000	-4.509662000
H	-1.228471000	-0.768128000	-3.884284000
C	-3.297119000	-2.312580000	-2.853461000
H	-3.594315000	-2.916629000	-3.716369000
H	-2.301914000	-2.637748000	-2.539026000
H	-3.998350000	-2.486161000	-2.034517000
C	-4.657380000	-0.373619000	-3.681188000
H	-4.922959000	-0.904440000	-4.600540000
H	-5.417865000	-0.585591000	-2.926315000
H	-4.664239000	0.699792000	-3.882087000
C	-5.942803000	-0.772523000	0.532827000
C	-5.760471000	0.295604000	1.609449000
H	-6.574651000	0.243069000	2.339004000
H	-4.811226000	0.156261000	2.131423000
H	-5.767295000	1.295183000	1.165790000

C	-5.971458000	-2.163055000	1.158527000
H	-6.824651000	-2.270112000	1.835440000
H	-6.047749000	-2.925531000	0.377814000
H	-5.051338000	-2.340471000	1.721651000
C	-7.204005000	-0.507665000	-0.285655000
H	-8.094122000	-0.517931000	0.351738000
H	-7.129132000	0.466685000	-0.775225000
H	-7.321358000	-1.276267000	-1.055838000
C	-4.583031000	3.088601000	-1.505253000
C	-5.605895000	3.630309000	-2.498494000
H	-5.483275000	4.709259000	-2.634235000
H	-5.491107000	3.141435000	-3.470499000
H	-6.618997000	3.436045000	-2.135294000
C	-4.727190000	3.760642000	-0.142839000
H	-4.490151000	4.827784000	-0.203034000
H	-5.752631000	3.654763000	0.224219000
H	-4.046846000	3.287031000	0.568264000
C	-3.164092000	3.276931000	-2.044460000
H	-2.979708000	4.331133000	-2.272300000
H	-2.433626000	2.960290000	-1.295449000
H	-3.018183000	2.694160000	-2.958522000

KCe[OSi(O^{t}Bu)$_3$]$_4$ complex (s=1/2)

Ce	-0.719416000	-0.085981000	-0.370734000
K	2.548999000	0.508770000	0.754454000
Si	0.752980000	-1.894581000	2.697006000
Si	2.420082000	-1.014755000	-2.466583000
Si	0.519343000	3.341393000	0.752812000
Si	-3.942241000	-0.442350000	-0.837250000
O	2.352761000	-1.335468000	2.806117000
O	0.871551000	-3.536701000	2.426738000
O	-0.048823000	-1.756575000	4.157286000
O	0.124560000	-1.017560000	1.479861000
O	0.989468000	-0.666681000	-1.787579000
O	3.521371000	0.113385000	-1.826084000
O	2.432057000	-1.015863000	-4.141044000
O	3.116146000	-2.471022000	-2.024054000
O	0.242581000	1.906904000	0.037875000
O	2.093524000	3.169815000	1.366880000
O	0.394521000	4.667274000	-0.260690000
O	-0.422927000	3.704973000	2.077607000
O	-2.899527000	0.584468000	-0.149505000
O	-2.837813000	-1.457321000	-1.667241000
O	-4.783904000	-1.480196000	0.158922000
O	-5.083776000	0.223275000	-1.860352000
C	3.467760000	-1.905222000	3.521695000
C	4.484933000	-0.771750000	3.679635000

H	5.364243000	-1.113879000	4.234378000
H	4.834470000	-0.418454000	2.702352000
H	4.043210000	0.070695000	4.221057000
C	4.064130000	-3.040808000	2.688128000
H	4.951020000	-3.458065000	3.177300000
H	3.325794000	-3.833381000	2.552162000
H	4.357867000	-2.675451000	1.699000000
C	3.049113000	-2.411803000	4.902027000
H	3.917357000	-2.819098000	5.430665000
H	2.627872000	-1.602987000	5.505063000
H	2.299317000	-3.200848000	4.816642000
C	-0.055969000	-4.605308000	2.686185000
C	0.122474000	-5.073251000	4.132064000
H	-0.548206000	-5.910098000	4.354770000
H	1.151190000	-5.407991000	4.300425000
H	-0.098378000	-4.254052000	4.820853000
C	-1.497043000	-4.157011000	2.444433000
H	-2.186684000	-4.996688000	2.580120000
H	-1.775302000	-3.366941000	3.145714000
H	-1.617389000	-3.779351000	1.426000000
C	0.322110000	-5.723137000	1.713713000
H	-0.313828000	-6.601555000	1.864671000
H	0.207270000	-5.386207000	0.679309000
H	1.364221000	-6.021864000	1.862559000
C	-0.672700000	-0.627270000	4.788461000
C	-0.731346000	-0.970203000	6.278533000
H	-1.231784000	-0.175230000	6.840825000
H	-1.282797000	-1.902607000	6.432650000
H	0.276954000	-1.098592000	6.683533000
C	-2.086503000	-0.465865000	4.227107000
H	-2.605873000	0.364796000	4.716385000
H	-2.053327000	-0.269689000	3.152371000
H	-2.668294000	-1.377456000	4.396057000
C	0.143108000	0.645935000	4.569226000
H	-0.303861000	1.485976000	5.109611000
H	1.167083000	0.512211000	4.930288000
H	0.174128000	0.913302000	3.509595000
C	4.954830000	0.160934000	-1.984312000
C	5.599562000	-0.688690000	-0.884427000
H	6.690121000	-0.693786000	-0.982860000
H	5.368587000	-0.287319000	0.110049000
H	5.231552000	-1.715259000	-0.942725000
C	5.347647000	1.630878000	-1.821753000
H	6.433835000	1.751839000	-1.888463000
H	4.881480000	2.240929000	-2.600389000
H	5.024106000	2.019010000	-0.850252000
C	5.405234000	-0.341676000	-3.356307000

H	6.492597000	-0.246968000	-3.445958000
H	5.136561000	-1.390204000	-3.498522000
H	4.942130000	0.240838000	-4.155706000
C	1.794036000	-0.135692000	-5.078173000
C	1.872812000	1.317375000	-4.609067000
H	1.396164000	1.980969000	-5.338265000
H	1.361630000	1.438360000	-3.650007000
H	2.912465000	1.634132000	-4.487349000
C	2.550177000	-0.313827000	-6.396231000
H	2.514026000	-1.359605000	-6.716059000
H	2.108534000	0.304307000	-7.184728000
H	3.600159000	-0.029072000	-6.281127000
C	0.334939000	-0.560318000	-5.244386000
H	0.277863000	-1.603129000	-5.570861000
H	-0.193386000	-0.467792000	-4.292771000
H	-0.171527000	0.061960000	-5.990043000
C	2.901456000	-3.815403000	-2.477569000
C	3.762159000	-4.061515000	-3.718903000
H	3.637066000	-5.087350000	-4.082179000
H	3.481063000	-3.368231000	-4.515568000
H	4.820846000	-3.912235000	-3.483335000
C	1.426587000	-4.055628000	-2.793716000
H	1.255895000	-5.100613000	-3.073037000
H	0.809356000	-3.826143000	-1.920492000
H	1.105721000	-3.424447000	-3.627193000
C	3.349024000	-4.717104000	-1.326361000
H	3.241687000	-5.773340000	-1.594659000
H	4.399776000	-4.529920000	-1.083855000
H	2.749835000	-4.520477000	-0.432866000
C	2.817286000	4.022191000	2.276367000
C	4.299437000	3.718119000	2.043125000
H	4.929071000	4.318873000	2.707108000
H	4.518549000	2.663528000	2.246610000
H	4.581006000	3.940159000	1.009104000
C	2.421091000	3.664684000	3.709853000
H	2.981643000	4.269835000	4.430549000
H	1.352379000	3.834381000	3.855436000
H	2.631269000	2.609649000	3.912832000
C	2.546632000	5.501835000	2.001484000
H	3.141607000	6.123139000	2.679210000
H	2.809556000	5.763645000	0.973372000
H	1.492151000	5.740234000	2.155546000
C	0.752654000	4.824095000	-1.640958000
C	0.794765000	6.334048000	-1.883116000
H	1.037015000	6.555601000	-2.927594000
H	-0.175528000	6.782263000	-1.649368000
H	1.549378000	6.803021000	-1.244587000

C	2.120494000	4.201986000	-1.926353000
H	2.408823000	4.375959000	-2.968372000
H	2.887009000	4.641946000	-1.281811000
H	2.099960000	3.121805000	-1.757366000
C	-0.323524000	4.175591000	-2.513752000
H	-0.092607000	4.300267000	-3.576917000
H	-0.388442000	3.107311000	-2.292832000
H	-1.299449000	4.629944000	-2.317910000
C	-1.709417000	4.345320000	2.175692000
C	-1.496120000	5.859881000	2.221516000
H	-2.453037000	6.383056000	2.323405000
H	-0.867074000	6.131843000	3.075526000
H	-1.008046000	6.198434000	1.303973000
C	-2.314693000	3.851166000	3.489760000
H	-3.287672000	4.320310000	3.668967000
H	-2.452622000	2.766926000	3.459164000
H	-1.655170000	4.093338000	4.329078000
C	-2.610490000	3.963552000	1.002078000
H	-3.599980000	4.417051000	1.125542000
H	-2.186395000	4.329860000	0.063105000
H	-2.735529000	2.879137000	0.931193000
C	-3.093937000	-2.718088000	-2.339028000
C	-2.128683000	-2.775083000	-3.518905000
H	-2.199163000	-3.741490000	-4.028628000
H	-2.360671000	-1.988503000	-4.242903000
H	-1.098441000	-2.638511000	-3.181413000
C	-2.810425000	-3.840477000	-1.342542000
H	-2.987701000	-4.820956000	-1.796820000
H	-1.766287000	-3.806493000	-1.014158000
H	-3.457831000	-3.734147000	-0.468458000
C	-4.535589000	-2.800373000	-2.840027000
H	-4.679228000	-3.736650000	-3.389304000
H	-5.242696000	-2.775952000	-2.007912000
H	-4.763705000	-1.967864000	-3.509959000
C	-5.934274000	-1.223760000	0.984378000
C	-5.876117000	0.185955000	1.574582000
H	-6.720677000	0.352684000	2.251547000
H	-4.947637000	0.331739000	2.133037000
H	-5.923572000	0.940289000	0.783419000
C	-5.879310000	-2.269917000	2.097875000
H	-6.744213000	-2.177670000	2.762898000
H	-5.877765000	-3.277412000	1.670839000
H	-4.968638000	-2.147431000	2.691181000
C	-7.197034000	-1.405841000	0.139549000
H	-8.095281000	-1.245057000	0.745581000
H	-7.200739000	-0.698354000	-0.693152000
H	-7.238358000	-2.420759000	-0.268662000

C	-5.002947000	1.390424000	-2.694777000
C	-6.160309000	1.265513000	-3.686836000
H	-6.204423000	2.139894000	-4.344157000
H	-6.042539000	0.371391000	-4.306650000
H	-7.111098000	1.186448000	-3.151471000
C	-5.172845000	2.635296000	-1.822989000
H	-5.128945000	3.546531000	-2.429281000
H	-6.140454000	2.612102000	-1.311782000
H	-4.380662000	2.674562000	-1.071357000
C	-3.668691000	1.437951000	-3.441722000
H	-3.652177000	2.276776000	-4.145210000
H	-2.843216000	1.573196000	-2.736966000
H	-3.506338000	0.513627000	-4.003609000

2-Pr^{OtBu} complex (s=1/2), dispersion

Pr	-0.014054000	-0.202384000	0.089496000
Si	-1.846384000	2.693478000	-0.994751000
Si	-1.830260000	-2.957763000	-0.941837000
Si	3.398016000	0.100252000	-1.123500000
Si	0.310162000	0.071419000	3.153374000
O	1.758644000	0.100845000	-0.980611000
O	-0.517272000	-1.955971000	-0.929164000
O	-1.470720000	1.172163000	-0.484739000
O	-0.621853000	3.411039000	-1.846150000
O	-2.029538000	3.582906000	0.397470000
O	0.869619000	1.177510000	1.981563000
O	3.782471000	1.643120000	-1.589063000
O	-3.183841000	2.691725000	-1.977058000
O	1.627182000	-0.418830000	4.023859000
O	4.187121000	-0.180266000	0.312042000
O	-0.288168000	-1.010069000	2.080471000
O	-2.693426000	-2.887525000	-2.354706000
O	-0.758740000	0.726282000	4.222870000
O	3.916255000	-1.070630000	-2.181060000
O	-1.392129000	-4.542742000	-0.725891000
O	-2.816617000	-2.377610000	0.261163000
C	5.083220000	2.253355000	-1.641952000
C	-0.221558000	3.139066000	-3.202760000
C	-2.197562000	-3.016515000	-3.698368000
C	1.555754000	2.460367000	2.133000000
C	4.376289000	-1.439032000	0.983848000
C	-4.385697000	1.918404000	-1.821001000
C	-2.273925000	4.997117000	0.508249000
C	3.335559000	-1.429394000	-3.445851000
C	4.939698000	3.421358000	-2.612241000
H	4.646952000	3.058306000	-3.601638000
H	5.883431000	3.967670000	-2.704482000

H	4.168106000	4.111111000	-2.259991000
C	-0.386777000	-5.071466000	0.151873000
C	-4.137927000	-2.836690000	0.599982000
C	1.573849000	-1.242022000	5.212401000
C	-0.454412000	1.672820000	-3.555323000
H	0.120101000	1.026486000	-2.887787000
H	-0.129945000	1.472125000	-4.580475000
H	-1.512774000	1.415394000	-3.475576000
C	3.062987000	-2.216900000	1.014774000
H	2.301246000	-1.664514000	1.573151000
H	3.194272000	-3.181296000	1.514538000
H	2.708823000	-2.416460000	-0.000519000
C	5.439526000	2.749863000	-0.242915000
H	4.669666000	3.437610000	0.117124000
H	6.400012000	3.275113000	-0.251126000
H	5.500855000	1.906473000	0.448040000
C	-2.630496000	1.153243000	2.691349000
H	-2.088058000	1.966938000	2.205051000
H	-3.693427000	1.411444000	2.668758000
H	-2.498718000	0.237296000	2.110817000
C	-1.037850000	4.049869000	-4.115262000
H	-2.101500000	3.817899000	-4.016527000
H	-0.741329000	3.921929000	-5.161312000
H	-0.880555000	5.096135000	-3.836110000
C	-2.182057000	0.945754000	4.131913000
C	-3.205612000	5.489694000	-0.597134000
H	-2.759375000	5.337248000	-1.583305000
H	-3.400793000	6.558949000	-0.469011000
H	-4.159492000	4.957663000	-0.570377000
C	1.592491000	3.082523000	0.747582000
H	2.163796000	2.469875000	0.048566000
H	2.069945000	4.065687000	0.797101000
H	0.584898000	3.226116000	0.356159000
C	1.265408000	3.466670000	-3.261765000
H	1.432930000	4.505033000	-2.959897000
H	1.648973000	3.335586000	-4.278509000
H	1.831290000	2.814403000	-2.592394000
C	0.745504000	3.321770000	3.091486000
H	-0.266181000	3.455453000	2.700459000
H	1.214883000	4.305270000	3.189294000
H	0.682196000	2.866835000	4.082967000
C	5.465838000	-2.231012000	0.265085000
H	5.168022000	-2.442641000	-0.763047000
H	5.659882000	-3.173734000	0.786630000
H	6.394183000	-1.651961000	0.245313000
C	-5.476984000	2.717691000	-2.526303000
H	-5.617231000	3.687245000	-2.040086000

H	-6.427951000	2.176610000	-2.506571000
H	-5.197506000	2.894976000	-3.568734000
C	2.802982000	-0.193989000	-4.166266000
H	2.014935000	0.282805000	-3.580503000
H	2.384032000	-0.471679000	-5.138462000
H	3.600331000	0.536711000	-4.325566000
C	-4.722113000	1.733419000	-0.343494000
H	-3.949253000	1.140437000	0.152214000
H	-5.679562000	1.215430000	-0.234124000
H	-4.793069000	2.701126000	0.160248000
C	-0.937992000	5.733507000	0.436857000
H	-0.278904000	5.407105000	1.244662000
H	-1.089664000	6.813681000	0.530428000
H	-0.446480000	5.523563000	-0.515743000
C	-4.174490000	0.571495000	-2.505863000
H	-3.893214000	0.731082000	-3.551276000
H	-5.089143000	-0.027573000	-2.485808000
H	-3.385153000	0.001703000	-2.010628000
C	2.219917000	-2.437103000	-3.187498000
H	2.622246000	-3.316542000	-2.676346000
H	1.763942000	-2.764146000	-4.126980000
H	1.441403000	-2.001695000	-2.557171000
C	4.467342000	-2.069179000	-4.244078000
H	5.277626000	-1.350709000	-4.397591000
H	4.109682000	-2.407353000	-5.221456000
H	4.870101000	-2.930527000	-3.703526000
C	6.130246000	1.262631000	-2.145903000
H	6.220405000	0.412245000	-1.465079000
H	7.107142000	1.750983000	-2.217405000
H	5.858782000	0.881772000	-3.133802000
C	-2.925634000	5.182221000	1.875078000
H	-3.864584000	4.623466000	1.925975000
H	-3.134575000	6.238853000	2.067854000
H	-2.264506000	4.810735000	2.662959000
C	4.817620000	-1.080945000	2.397488000
H	5.704676000	-0.441806000	2.360328000
H	5.066206000	-1.985372000	2.960590000
H	4.019557000	-0.554084000	2.923657000
C	-1.512451000	-1.711093000	-4.095783000
H	-2.210149000	-0.876332000	-4.007010000
H	-1.157410000	-1.759526000	-5.130202000
H	-0.659073000	-1.515227000	-3.443969000
C	-4.270356000	-4.346762000	0.413386000
H	-4.113594000	-4.629021000	-0.631361000
H	-5.274291000	-4.668285000	0.708113000
H	-3.539840000	-4.882832000	1.023366000
C	-5.139883000	-2.104062000	-0.285729000

H	-5.034567000	-1.026611000	-0.147875000
H	-6.165513000	-2.391649000	-0.032295000
H	-4.950952000	-2.338481000	-1.335648000
C	-2.862294000	-0.277236000	4.737697000
H	-2.616929000	-1.166644000	4.151592000
H	-3.950131000	-0.157524000	4.739327000
H	-2.528328000	-0.428641000	5.768630000
C	2.958841000	2.180866000	2.647926000
H	2.924615000	1.705947000	3.631169000
H	3.519857000	3.117102000	2.726917000
H	3.479591000	1.517636000	1.954004000
C	-0.716130000	-6.554937000	0.291460000
H	-1.701490000	-6.684729000	0.748101000
H	0.027319000	-7.060475000	0.915574000
H	-0.729141000	-7.031048000	-0.693073000
C	-2.451944000	2.194410000	4.964328000
H	-2.082581000	2.058541000	5.984883000
H	-3.524904000	2.404264000	5.005088000
H	-1.946621000	3.058752000	4.526312000
C	0.973192000	-4.882233000	-0.514658000
H	0.992852000	-5.394810000	-1.480750000
H	1.777535000	-5.283670000	0.109708000
H	1.155938000	-3.820179000	-0.684726000
C	-0.425057000	-4.386488000	1.516875000
H	-0.214635000	-3.316987000	1.447563000
H	0.320869000	-4.836714000	2.180296000
H	-1.408308000	-4.503909000	1.980655000
C	2.937815000	-1.913266000	5.312829000
H	2.993841000	-2.526452000	6.217308000
H	3.731375000	-1.162466000	5.351457000
H	3.111690000	-2.553917000	4.444554000
C	-4.331443000	-2.461253000	2.064073000
H	-3.570829000	-2.948188000	2.680590000
H	-5.321042000	-2.766031000	2.418351000
H	-4.234172000	-1.380283000	2.189191000
C	-1.226353000	-4.188799000	-3.812792000
H	-0.331025000	-4.013333000	-3.211006000
H	-0.917661000	-4.318616000	-4.855112000
H	-1.693568000	-5.112504000	-3.463523000
C	-3.436754000	-3.258743000	-4.554144000
H	-3.931855000	-4.184539000	-4.247266000
H	-3.170917000	-3.337393000	-5.612739000
H	-4.142957000	-2.432463000	-4.431624000
C	1.326248000	-0.333044000	6.412807000
H	2.117099000	0.419973000	6.479873000
H	1.320320000	-0.913083000	7.341100000
H	0.368085000	0.179442000	6.305730000

C	0.477956000	-2.296070000	5.073948000
H	-0.509541000	-1.829938000	5.029335000
H	0.491353000	-2.973497000	5.933166000
H	0.625717000	-2.881747000	4.162793000

2-Pr^{OtBu} complex (s=1/2)

Pr	0.022308000	-0.098170000	0.112518000
Si	-1.958041000	3.003618000	-0.587754000
Si	-1.973912000	-2.896467000	-1.159951000
Si	3.463956000	0.169569000	-1.331230000
Si	0.528283000	-0.348586000	3.216109000
O	1.894921000	-0.035028000	-0.838160000
O	-0.904814000	-1.643819000	-0.941289000
O	-1.214320000	1.535848000	-0.388581000
O	-1.004935000	4.155391000	-1.314284000
O	-2.244783000	3.557675000	0.960073000
O	0.935319000	1.020310000	2.277749000
O	3.590859000	1.783555000	-1.720159000
O	-3.329008000	2.897652000	-1.523776000
O	1.925429000	-0.892297000	3.915297000
O	4.576777000	-0.124538000	-0.128205000
O	-0.061002000	-1.223193000	1.956973000
O	-2.834787000	-2.793400000	-2.579146000
O	-0.538958000	-0.038040000	4.440493000
O	3.869092000	-0.853411000	-2.582229000
O	-1.202171000	-4.369264000	-1.199592000
O	-3.086282000	-2.762000000	0.069387000
C	4.771772000	2.581483000	-1.940446000
C	-0.629430000	4.361943000	-2.687389000
C	-2.477382000	-2.866131000	-3.967956000
C	1.684074000	2.232388000	2.606454000
C	5.171479000	-1.353611000	0.331233000
C	-4.454437000	2.006831000	-1.509594000
C	-2.658063000	4.866220000	1.402262000
C	3.196575000	-1.195016000	-3.805203000
C	4.313418000	3.770860000	-2.783478000
H	3.903184000	3.430123000	-3.738842000
H	5.150639000	4.446399000	-2.986956000
H	3.536389000	4.332781000	-2.259006000
C	-0.331245000	-5.053482000	-0.285594000
C	-4.385530000	-3.366540000	0.205920000
C	2.114530000	-1.839973000	4.990519000
C	-0.144032000	3.051189000	-3.306178000
H	0.703971000	2.651483000	-2.743092000
H	0.176382000	3.212017000	-4.340818000
H	-0.945853000	2.306060000	-3.313753000
C	4.110966000	-2.447200000	0.449848000

H	3.313672000	-2.136677000	1.131428000
H	4.554763000	-3.370356000	0.837591000
H	3.675356000	-2.667023000	-0.528847000
C	5.299622000	3.060088000	-0.587029000
H	4.527675000	3.628543000	-0.060218000
H	6.172863000	3.707931000	-0.720225000
H	5.584455000	2.206096000	0.031494000
C	-2.584332000	0.496418000	3.179121000
H	-2.145525000	1.428159000	2.810364000
H	-3.660017000	0.662717000	3.296399000
H	-2.450606000	-0.290521000	2.431376000
C	-1.816758000	4.930139000	-3.467293000
H	-2.652796000	4.228398000	-3.459628000
H	-1.529920000	5.133160000	-4.504736000
H	-2.151636000	5.869896000	-3.016732000
C	-1.976718000	0.073914000	4.514856000
C	-3.653157000	5.504143000	0.431317000
H	-3.202807000	5.642866000	-0.554749000
H	-3.965690000	6.484310000	0.806466000
H	-4.544402000	4.881303000	0.318494000
C	1.538795000	3.143385000	1.393653000
H	1.980214000	2.681148000	0.504746000
H	2.060676000	4.089131000	1.570605000
H	0.487433000	3.365694000	1.199116000
C	0.505927000	5.384463000	-2.639744000
H	0.156684000	6.319984000	-2.191968000
H	0.878785000	5.601014000	-3.645868000
H	1.332261000	5.001628000	-2.035402000
C	1.064808000	2.871488000	3.846308000
H	0.010147000	3.101237000	3.671581000
H	1.587216000	3.803704000	4.083838000
H	1.138318000	2.209846000	4.714143000
C	6.278240000	-1.770328000	-0.639305000
H	5.866504000	-1.935216000	-1.637197000
H	6.761397000	-2.691791000	-0.297066000
H	7.042691000	-0.989190000	-0.700591000
C	-5.617786000	2.802417000	-2.105599000
H	-5.843789000	3.677481000	-1.489221000
H	-6.517984000	2.182965000	-2.172191000
H	-5.363349000	3.151088000	-3.110940000
C	2.331044000	-0.039135000	-4.306351000
H	1.538268000	0.194001000	-3.590037000
H	1.860972000	-0.301199000	-5.259639000
H	2.934322000	0.860513000	-4.454731000
C	-4.788440000	1.563098000	-0.085797000
H	-3.965648000	0.985371000	0.344853000
H	-5.683813000	0.933579000	-0.085702000

H	-4.976874000	2.427537000	0.556491000
C	-1.419305000	5.752427000	1.547337000
H	-0.718543000	5.315135000	2.264530000
H	-1.698022000	6.749026000	1.906336000
H	-0.912683000	5.852229000	0.584670000
C	-4.125660000	0.804817000	-2.392865000
H	-3.867773000	1.143726000	-3.401370000
H	-4.982175000	0.127709000	-2.464707000
H	-3.281624000	0.243011000	-1.986651000
C	2.348050000	-2.438434000	-3.543125000
H	2.979161000	-3.256334000	-3.182273000
H	1.845694000	-2.770502000	-4.457535000
H	1.588929000	-2.229899000	-2.784866000
C	4.300649000	-1.509219000	-4.816657000
H	4.924166000	-0.628103000	-4.993967000
H	3.870855000	-1.826698000	-5.772220000
H	4.942892000	-2.312537000	-4.443419000
C	5.848548000	1.795323000	-2.689642000
H	6.179282000	0.930948000	-2.108716000
H	6.717873000	2.434419000	-2.876819000
H	5.470862000	1.440239000	-3.652266000
C	-3.320631000	4.649564000	2.763320000
H	-4.198257000	4.003614000	2.665424000
H	-3.638652000	5.603603000	3.196143000
H	-2.621383000	4.173116000	3.456770000
C	5.764101000	-1.033443000	1.703135000
H	6.489157000	-0.217706000	1.622750000
H	6.276149000	-1.909143000	2.115141000
H	4.977782000	-0.730735000	2.399196000
C	-1.527358000	-1.720414000	-4.316706000
H	-1.987414000	-0.757505000	-4.078887000
H	-1.285088000	-1.733801000	-5.384713000
H	-0.595932000	-1.802300000	-3.751739000
C	-4.408704000	-4.791874000	-0.349772000
H	-4.181072000	-4.794887000	-1.419134000
H	-5.400924000	-5.233567000	-0.208972000
H	-3.678082000	-5.424201000	0.161506000
C	-5.407313000	-2.496663000	-0.527229000
H	-5.385230000	-1.478551000	-0.129315000
H	-6.419424000	-2.896475000	-0.401576000
H	-5.170931000	-2.458728000	-1.592938000
C	-2.525960000	-1.287148000	4.943036000
H	-2.304841000	-2.043940000	4.184752000
H	-3.612167000	-1.241413000	5.073017000
H	-2.081001000	-1.599011000	5.892982000
C	3.146925000	1.865928000	2.836392000
H	3.252976000	1.199917000	3.695253000

H	3.730507000	2.773829000	3.021414000
H	3.558313000	1.365481000	1.956023000
C	-0.570699000	-6.545340000	-0.530673000
H	-1.606433000	-6.811844000	-0.300662000
H	0.090291000	-7.153089000	0.095880000
H	-0.380478000	-6.793906000	-1.578976000
C	-2.250093000	1.126328000	5.589482000
H	-1.784451000	0.835695000	6.535990000
H	-3.326215000	1.239628000	5.753498000
H	-1.842338000	2.095984000	5.290631000
C	1.111363000	-4.688327000	-0.633447000
H	1.325553000	-4.938528000	-1.676551000
H	1.815227000	-5.233231000	0.004370000
H	1.279301000	-3.617792000	-0.494636000
C	-0.639846000	-4.694895000	1.167732000
H	-0.482643000	-3.629705000	1.362088000
H	0.014785000	-5.263926000	1.836910000
H	-1.675532000	-4.940270000	1.416573000
C	3.560868000	-2.315560000	4.865473000
H	3.800103000	-3.027609000	5.661795000
H	4.250307000	-1.469742000	4.941151000
H	3.721738000	-2.805552000	3.901288000
C	-4.676111000	-3.377759000	1.707238000
H	-3.934840000	-3.982365000	2.238240000
H	-5.669651000	-3.792110000	1.907598000
H	-4.637519000	-2.360474000	2.107290000
C	-1.842093000	-4.219865000	-4.288982000
H	-0.912127000	-4.358101000	-3.733508000
H	-1.628369000	-4.292897000	-5.360737000
H	-2.519852000	-5.033726000	-4.015381000
C	-3.795202000	-2.709921000	-4.729480000
H	-4.487159000	-3.514288000	-4.462045000
H	-3.625710000	-2.745076000	-5.810429000
H	-4.266163000	-1.753955000	-4.482941000
C	1.900213000	-1.124831000	6.324705000
H	2.592264000	-0.282006000	6.418097000
H	2.080494000	-1.809040000	7.160526000
H	0.878874000	-0.744080000	6.394212000
C	1.154461000	-3.019056000	4.834114000
H	0.115681000	-2.693175000	4.936681000
H	1.347648000	-3.774575000	5.602695000
H	1.277603000	-3.485891000	3.852744000

3-Pr^{OtBu} complex, (s=2), dispersion

Pr	-2.046963000	0.164133000	0.098242000
Si	-3.817932000	-2.996009000	-0.048647000
Si	-4.397252000	2.859290000	0.062448000

Si	-0.417158000	0.541125000	-2.714759000
O	-3.005888000	2.089082000	0.434354000
O	-3.425731000	-1.487316000	0.441491000
O	-2.676278000	-3.601736000	-1.107747000
O	-5.244319000	-2.922571000	-0.906894000
O	-2.067939000	0.275839000	-2.397116000
O	-3.945258000	-4.069444000	1.229513000
O	0.087163000	-0.749687000	-3.595237000
O	0.080169000	0.547569000	-1.152892000
O	-5.710535000	1.842283000	-0.009854000
O	-0.122136000	1.902017000	-3.592280000
O	-4.789654000	4.058700000	1.155420000
O	-4.255904000	3.465299000	-1.488652000
C	-1.326927000	-4.028020000	-0.871291000
C	-6.423663000	1.225434000	1.071961000
C	-3.138424000	0.059963000	-3.373581000
C	-4.917215000	-4.099242000	2.288681000
C	-5.804020000	-3.851578000	-1.855240000
C	-3.894655000	5.010520000	1.743321000
C	-5.260335000	4.153862000	-2.256618000
C	1.099603000	-0.860833000	-4.615132000
C	-0.548631000	-2.961429000	-0.103097000
H	-0.504671000	-2.049819000	-0.711873000
H	0.483850000	-3.279683000	0.065241000
H	-0.994706000	-2.744056000	0.869644000
C	-1.007773000	3.538552000	-1.999034000
H	-2.047662000	3.273055000	-2.195522000
H	-0.984712000	4.597486000	-1.725770000
H	-0.663202000	2.967192000	-1.134714000
C	-1.346292000	-5.348636000	-0.107691000
H	-1.840062000	-5.225440000	0.858667000
H	-0.329354000	-5.719476000	0.046767000
H	-1.905366000	-6.096700000	-0.677243000
C	-0.106252000	3.291150000	-3.199084000
C	-5.407571000	-5.296639000	-1.557524000
H	-4.320377000	-5.400077000	-1.580384000
H	-5.834554000	-5.957683000	-2.318369000
H	-5.767746000	-5.621020000	-0.582802000
C	-2.881485000	-1.259075000	-4.083433000
H	-1.977109000	-1.208841000	-4.689056000
H	-3.729114000	-1.501221000	-4.729486000
H	-2.760578000	-2.056120000	-3.347623000
C	-0.695894000	-4.198050000	-2.247029000
H	-1.237988000	-4.950893000	-2.826829000
H	0.347916000	-4.507109000	-2.146280000
H	-0.721771000	-3.248672000	-2.787694000
C	-4.416422000	0.016344000	-2.555071000

H	-4.427649000	-0.835039000	-1.871120000
H	-5.284005000	-0.081660000	-3.213326000
H	-4.543562000	0.938992000	-1.987270000
C	-4.304981000	-4.982114000	3.372984000
H	-4.134894000	-5.987170000	2.975974000
H	-4.972416000	-5.057485000	4.237053000
H	-3.345595000	-4.584734000	3.701884000
C	-6.215149000	-4.737638000	1.801137000
H	-6.639584000	-4.149817000	0.986330000
H	-6.943549000	-4.775305000	2.617542000
H	-6.038767000	-5.759352000	1.455266000
C	-5.309326000	-3.481383000	-3.248374000
H	-5.553699000	-2.442038000	-3.476516000
H	-5.777089000	-4.122295000	-4.002727000
H	-4.227746000	-3.610278000	-3.303794000
C	-5.226623000	-2.691459000	2.793677000
H	-4.322728000	-2.157010000	3.080839000
H	-5.898028000	-2.739263000	3.657097000
H	-5.721710000	-2.111775000	2.010979000
C	-7.316494000	-3.662647000	-1.771870000
H	-7.695978000	-3.945576000	-0.786642000
H	-7.823173000	-4.276483000	-2.523051000
H	-7.570240000	-2.613313000	-1.946756000
C	-5.482575000	0.874690000	2.223502000
H	-4.737187000	0.145725000	1.895483000
H	-6.046929000	0.428403000	3.048321000
H	-4.980922000	1.770413000	2.599962000
C	-6.106085000	5.083660000	-1.388861000
H	-6.643330000	4.527100000	-0.617866000
H	-6.837577000	5.603883000	-2.015245000
H	-5.481229000	5.831208000	-0.895731000
C	-6.160414000	3.126223000	-2.939953000
H	-5.580618000	2.496615000	-3.617759000
H	-6.940498000	3.628420000	-3.521249000
H	-6.628443000	2.486786000	-2.188696000
C	1.340634000	3.643049000	-2.886592000
H	1.707471000	3.064453000	-2.034548000
H	1.441282000	4.704055000	-2.643900000
H	1.974736000	3.430508000	-3.750889000
C	-3.151096000	1.230850000	-4.344518000
H	-3.282372000	2.164584000	-3.794376000
H	-3.982613000	1.118030000	-5.046648000
H	-2.221824000	1.284784000	-4.916039000
C	-4.730410000	6.248954000	2.051713000
H	-5.113518000	6.697018000	1.131284000
H	-4.131479000	6.998433000	2.578817000
H	-5.583644000	5.978184000	2.680121000

C	-0.600002000	4.066257000	-4.415642000
H	0.030847000	3.852641000	-5.283395000
H	-0.572350000	5.142334000	-4.220536000
H	-1.627763000	3.785265000	-4.656036000
C	-3.357924000	4.387610000	3.025661000
H	-4.184850000	4.142039000	3.699127000
H	-2.672859000	5.065523000	3.545918000
H	-2.829486000	3.468276000	2.771897000
C	-2.742735000	5.353048000	0.798393000
H	-2.146698000	4.461342000	0.586714000
H	-2.091054000	6.107270000	1.251143000
H	-3.121279000	5.745961000	-0.148731000
C	1.236342000	-2.358741000	-4.846053000
H	1.998709000	-2.570436000	-5.601243000
H	0.284982000	-2.780672000	-5.182816000
H	1.515707000	-2.844194000	-3.908924000
C	-4.487684000	4.970842000	-3.288786000
H	-3.819556000	5.677861000	-2.788323000
H	-5.172318000	5.530846000	-3.933033000
H	-3.880948000	4.316157000	-3.919751000
C	-7.500739000	2.203337000	1.535866000
H	-7.035307000	3.119363000	1.908820000
H	-8.113587000	1.763923000	2.329691000
H	-8.154295000	2.462637000	0.697258000
C	-7.036269000	-0.041480000	0.482842000
H	-7.687887000	0.213610000	-0.358375000
H	-7.633941000	-0.569147000	1.233120000
H	-6.254023000	-0.713060000	0.120108000
C	0.622236000	-0.152771000	-5.880080000
H	-0.322448000	-0.582424000	-6.227374000
H	1.361873000	-0.266834000	-6.678791000
H	0.473619000	0.911376000	-5.686190000
C	2.401292000	-0.259216000	-4.101427000
H	2.266511000	0.809573000	-3.915848000
H	3.199645000	-0.371101000	-4.841741000
H	2.713051000	-0.749938000	-3.174852000
Pr	1.917554000	-0.022299000	0.131154000
Si	3.773847000	-2.934178000	-0.695585000
Si	4.304149000	2.888328000	0.074933000
Si	0.415120000	-0.501630000	3.007578000
O	3.230951000	1.671311000	-0.073782000
O	2.978156000	-1.544355000	-0.995434000
O	3.256411000	-4.218972000	-1.622663000
O	3.376547000	-3.241542000	0.905267000
O	2.024149000	-0.073266000	2.667832000
O	5.419907000	-2.888385000	-0.941895000
O	0.449387000	-2.119984000	3.353005000

O	-0.229718000	-0.135940000	1.552725000
O	5.513117000	2.929295000	-1.064136000
O	-0.160849000	0.262831000	4.353233000
O	3.551574000	4.379615000	0.005799000
O	5.092797000	2.667109000	1.530971000
C	3.871783000	-4.852598000	-2.759002000
C	5.507838000	3.223056000	-2.464910000
C	3.150687000	0.036929000	3.594734000
C	6.431965000	-1.906347000	-0.697945000
C	3.490575000	-4.476664000	1.633436000
C	2.513987000	4.903992000	0.840440000
C	6.414102000	3.095246000	1.902307000
C	-0.501164000	-2.847879000	4.159205000
C	4.433215000	-3.818873000	-3.734113000
H	3.665308000	-3.095913000	-4.010825000
H	4.785459000	-4.313220000	-4.645198000
H	5.268349000	-3.284799000	-3.280104000
C	-0.337349000	2.554942000	3.558814000
H	0.747629000	2.652498000	3.604530000
H	-0.775217000	3.532185000	3.770810000
H	-0.615184000	2.271725000	2.540844000
C	4.985489000	-5.781970000	-2.280570000
H	5.755399000	-5.206254000	-1.762333000
H	5.445658000	-6.295869000	-3.130724000
H	4.584640000	-6.540462000	-1.601167000
C	-0.820412000	1.522130000	4.565197000
C	4.837742000	-5.144961000	1.381213000
H	4.949920000	-5.425499000	0.333815000
H	4.923642000	-6.050903000	1.989709000
H	5.654192000	-4.470434000	1.647054000
C	4.365957000	-0.396521000	2.789061000
H	4.187114000	-1.382770000	2.351383000
H	5.251270000	-0.453977000	3.429107000
H	4.579918000	0.335888000	2.004842000
C	2.762302000	-5.666502000	-3.420009000
H	2.325210000	-6.362938000	-2.698263000
H	3.157462000	-6.241970000	-4.262875000
H	1.968633000	-5.013997000	-3.790717000
C	3.249771000	1.494052000	4.014585000
H	3.383498000	2.134625000	3.141718000
H	4.114903000	1.637353000	4.667103000
H	2.356123000	1.788140000	4.568364000
C	7.543088000	-2.207918000	-1.702543000
H	7.863797000	-3.249559000	-1.610043000
H	8.407129000	-1.558836000	-1.530055000
H	7.189308000	-2.044646000	-2.724847000
C	6.948709000	-2.079339000	0.727421000

H	6.133300000	-1.954783000	1.442461000
H	7.721907000	-1.337562000	0.951181000
H	7.376790000	-3.077184000	0.859733000
C	2.338565000	-5.389259000	1.221396000
H	1.387497000	-4.904285000	1.451167000
H	2.379727000	-6.339573000	1.763341000
H	2.382166000	-5.585386000	0.147898000
C	5.900736000	-0.497742000	-0.921351000
H	5.376863000	-0.445372000	-1.876716000
H	6.716445000	0.228040000	-0.925608000
H	5.197665000	-0.193785000	-0.145479000
C	3.363547000	-4.092525000	3.102076000
H	4.224671000	-3.495517000	3.416973000
H	3.316164000	-4.984526000	3.734918000
H	2.454632000	-3.503663000	3.242397000
C	4.649185000	2.195965000	-3.193777000
H	5.084847000	1.199091000	-3.108304000
H	4.556463000	2.444197000	-4.255737000
H	3.652526000	2.162076000	-2.754142000
C	6.690393000	4.519109000	1.422834000
H	6.630668000	4.572243000	0.334077000
H	7.692034000	4.833080000	1.733540000
H	5.963645000	5.215518000	1.846597000
C	7.426343000	2.117383000	1.309359000
H	7.188676000	1.098092000	1.627512000
H	8.440847000	2.359182000	1.642851000
H	7.389231000	2.161572000	0.219821000
C	-2.323805000	1.292383000	4.452431000
H	-2.600598000	1.000022000	3.435234000
H	-2.875221000	2.202392000	4.698055000
H	-2.640002000	0.504100000	5.140862000
C	2.933570000	-0.850382000	4.812225000
H	2.050246000	-0.531772000	5.371465000
H	3.801935000	-0.768707000	5.472705000
H	2.809273000	-1.893108000	4.525861000
C	2.004384000	6.153256000	0.126292000
H	2.834405000	6.838321000	-0.066836000
H	1.254638000	6.670146000	0.733422000
H	1.548319000	5.886386000	-0.830284000
C	-0.451949000	1.939547000	5.985568000
H	-0.759040000	1.168923000	6.698209000
H	-0.946996000	2.878203000	6.250984000
H	0.628847000	2.079650000	6.075887000
C	1.378886000	3.898374000	1.009478000
H	1.025487000	3.554629000	0.036976000
H	0.539820000	4.357158000	1.539351000
H	1.713172000	3.030241000	1.581205000

C	3.096087000	5.275979000	2.203340000
H	3.553591000	4.401213000	2.670734000
H	2.314388000	5.662108000	2.865510000
H	3.864227000	6.046215000	2.090674000
C	-0.365571000	-4.310781000	3.756549000
H	-1.064259000	-4.928082000	4.328775000
H	0.647197000	-4.672659000	3.945183000
H	-0.588605000	-4.434846000	2.694585000
C	6.455374000	3.035393000	3.426466000
H	5.696375000	3.695099000	3.856778000
H	7.436815000	3.346295000	3.797226000
H	6.263514000	2.015417000	3.770295000
C	4.992503000	4.640486000	-2.705057000
H	3.960331000	4.741529000	-2.367044000
H	5.046668000	4.888198000	-3.770029000
H	5.597041000	5.361706000	-2.147816000
C	6.965454000	3.106699000	-2.903114000
H	7.584187000	3.826904000	-2.359675000
H	7.065233000	3.300613000	-3.975524000
H	7.341190000	2.100695000	-2.695122000
C	-0.143238000	-2.660039000	5.630801000
H	0.883494000	-2.989144000	5.813279000
H	-0.812192000	-3.251938000	6.263354000
H	-0.230700000	-1.608636000	5.911999000
C	-1.911291000	-2.355459000	3.868017000
H	-1.991076000	-1.282732000	4.044699000
H	-2.627042000	-2.846235000	4.531511000
H	-2.199168000	-2.570734000	2.835773000

3-Pr^{OtBu} complex, (s=2)

Pr	-1.995545000	0.062999000	-0.237540000
Si	-3.465650000	-3.420914000	-0.002997000
Si	-4.942895000	2.405370000	0.195720000
Si	-0.180794000	0.231350000	-3.052413000
O	-3.481005000	1.666393000	0.057762000
O	-2.866182000	-1.891283000	0.095376000
O	-2.314841000	-4.624808000	0.029473000
O	-4.206889000	-3.534241000	-1.499943000
O	-1.865421000	0.009550000	-2.768373000
O	-4.509929000	-3.805359000	1.246391000
O	0.369094000	-1.007666000	-3.997045000
O	0.283809000	0.197056000	-1.475052000
O	-5.780003000	1.988502000	1.581405000
O	0.145628000	1.607395000	-3.917594000
O	-4.869708000	4.080635000	0.254702000
O	-5.858330000	1.896299000	-1.109571000
C	-1.737939000	-5.363003000	1.118692000

C	-5.904434000	2.618037000	2.865045000
C	-2.949473000	-0.319230000	-3.707934000
C	-5.636630000	-3.123835000	1.808335000
C	-4.693450000	-4.693108000	-2.202468000
C	-4.353899000	5.078997000	-0.630717000
C	-7.274134000	1.936872000	-1.356657000
C	1.063106000	-1.092955000	-5.266110000
C	-1.394363000	-4.426776000	2.275083000
H	-0.721291000	-3.631070000	1.943951000
H	-0.904214000	-4.976888000	3.084936000
H	-2.306627000	-3.971800000	2.670231000
C	-0.691193000	3.356776000	-2.436841000
H	-1.705898000	2.954918000	-2.503623000
H	-0.766518000	4.439307000	-2.303190000
H	-0.199497000	2.941419000	-1.552481000
C	-2.714965000	-6.448970000	1.577535000
H	-3.648061000	-6.001200000	1.926474000
H	-2.277685000	-7.036950000	2.391870000
H	-2.938880000	-7.131779000	0.751438000
C	0.099553000	3.026150000	-3.696444000
C	-5.396947000	-5.675959000	-1.264217000
H	-4.710348000	-6.059771000	-0.506877000
H	-5.783518000	-6.525164000	-1.837837000
H	-6.237761000	-5.198445000	-0.754482000
C	-4.223983000	-0.345378000	-2.878043000
H	-4.187313000	-1.145436000	-2.131831000
H	-5.074397000	-0.567881000	-3.529924000
H	-4.424987000	0.614713000	-2.395833000
C	-0.474975000	-6.005314000	0.550513000
H	-0.726700000	-6.627429000	-0.313975000
H	0.006616000	-6.639061000	1.301607000
H	0.237485000	-5.241653000	0.229029000
C	-3.032424000	0.764443000	-4.777006000
H	-3.244684000	1.735577000	-4.320542000
H	-3.847166000	0.533986000	-5.470499000
H	-2.107005000	0.841223000	-5.351503000
C	-6.513772000	-4.209584000	2.436367000
H	-6.860733000	-4.912932000	1.673842000
H	-7.388730000	-3.768552000	2.925113000
H	-5.946539000	-4.771005000	3.185182000
C	-6.416196000	-2.357592000	0.741726000
H	-5.785727000	-1.595806000	0.274998000
H	-7.277089000	-1.852477000	1.190331000
H	-6.779678000	-3.031234000	-0.038558000
C	-3.521018000	-5.384821000	-2.903043000
H	-3.038159000	-4.703547000	-3.609257000
H	-3.868282000	-6.262341000	-3.459418000

H	-2.778399000	-5.700093000	-2.167133000
C	-5.133279000	-2.165196000	2.885849000
H	-4.541467000	-2.708018000	3.629039000
H	-5.968329000	-1.676529000	3.397090000
H	-4.506753000	-1.392537000	2.432850000
C	-5.693015000	-4.160322000	-3.231131000
H	-6.517504000	-3.642691000	-2.731385000
H	-6.108026000	-4.979036000	-3.827975000
H	-5.205361000	-3.452970000	-3.907178000
C	-4.555690000	3.160309000	3.335557000
H	-3.814046000	2.356951000	3.343748000
H	-4.636416000	3.566551000	4.349595000
H	-4.210186000	3.954216000	2.668740000
C	-7.896781000	3.259513000	-0.906999000
H	-7.754580000	3.415204000	0.163509000
H	-8.972499000	3.254097000	-1.112529000
H	-7.456061000	4.105554000	-1.440965000
C	-7.934749000	0.765675000	-0.628773000
H	-7.506576000	-0.179566000	-0.972968000
H	-9.013270000	0.746832000	-0.820182000
H	-7.765458000	0.849861000	0.447188000
C	1.541025000	3.515200000	-3.584105000
H	2.025617000	3.102667000	-2.695527000
H	1.574600000	4.607265000	-3.515735000
H	2.110157000	3.214719000	-4.468569000
C	-2.694502000	-1.698187000	-4.304228000
H	-1.756063000	-1.733665000	-4.855220000
H	-3.512778000	-1.956071000	-4.984389000
H	-2.662964000	-2.444777000	-3.507619000
C	-5.237732000	6.314634000	-0.441018000
H	-6.273767000	6.092726000	-0.711919000
H	-4.886830000	7.144397000	-1.063542000
H	-5.219538000	6.636591000	0.604682000
C	-0.565612000	3.644849000	-4.927687000
H	-0.015167000	3.374699000	-5.833874000
H	-0.575583000	4.736479000	-4.847433000
H	-1.596221000	3.298554000	-5.033752000
C	-2.919354000	5.396438000	-0.208448000
H	-2.901591000	5.737572000	0.830906000
H	-2.488789000	6.184470000	-0.835976000
H	-2.295922000	4.503342000	-0.287623000
C	-4.393856000	4.622009000	-2.089814000
H	-3.822621000	3.699471000	-2.222335000
H	-3.962900000	5.390820000	-2.739771000
H	-5.418795000	4.431184000	-2.414907000
C	1.075080000	-2.580938000	-5.617077000
H	1.635276000	-2.748528000	-6.542529000

H	0.056108000	-2.951498000	-5.762363000
H	1.537092000	-3.162838000	-4.818896000
C	-7.440755000	1.779576000	-2.869875000
H	-6.930358000	2.589319000	-3.400458000
H	-8.500410000	1.801306000	-3.144731000
H	-7.017739000	0.829594000	-3.206316000
C	-6.933805000	3.747854000	2.789662000
H	-6.614388000	4.501651000	2.066497000
H	-7.050521000	4.227092000	3.767844000
H	-7.910344000	3.356547000	2.487388000
C	-6.394965000	1.525711000	3.815995000
H	-7.335367000	1.100996000	3.451758000
H	-6.564259000	1.931002000	4.819011000
H	-5.659369000	0.720265000	3.885190000
C	0.335070000	-0.318442000	-6.366669000
H	-0.681396000	-0.696428000	-6.509521000
H	0.869577000	-0.443771000	-7.314135000
H	0.289784000	0.745979000	-6.132114000
C	2.481544000	-0.554362000	-5.096755000
H	2.453730000	0.507783000	-4.841891000
H	3.048956000	-0.669892000	-6.026066000
H	3.009173000	-1.092240000	-4.305819000
Pr	2.020350000	0.136193000	0.166308000
Si	3.919062000	-3.168561000	0.161594000
Si	4.497874000	2.953908000	-0.234597000
Si	0.191772000	0.501667000	2.933944000
O	3.199720000	1.943861000	-0.202820000
O	3.226003000	-1.680370000	0.039230000
O	3.080406000	-4.359310000	-0.659383000
O	3.907612000	-3.577872000	1.783844000
O	1.879608000	0.268416000	2.714543000
O	5.470725000	-3.267465000	-0.463737000
O	-0.393117000	-0.699217000	3.905482000
O	-0.229673000	0.377626000	1.347242000
O	5.713729000	2.498221000	-1.283375000
O	-0.156445000	1.917648000	3.721953000
O	4.110003000	4.517998000	-0.687900000
O	5.191703000	2.914950000	1.289333000
C	3.194062000	-4.770946000	-2.033749000
C	5.898715000	2.526152000	-2.703990000
C	2.921773000	-0.046247000	3.705577000
C	6.695925000	-2.559603000	-0.258533000
C	4.101809000	-4.856352000	2.420585000
C	3.316885000	5.573786000	-0.140160000
C	6.546119000	3.167563000	1.712640000
C	-1.225439000	-0.722366000	5.087755000
C	3.512963000	-3.577096000	-2.931485000

H	2.736092000	-2.814711000	-2.825637000
H	3.558447000	-3.888044000	-3.980208000
H	4.477264000	-3.141202000	-2.660696000
C	-0.778220000	3.576315000	2.056519000
H	-0.201503000	3.107552000	1.254893000
H	-0.823334000	4.649981000	1.856020000
H	-1.794772000	3.177688000	2.023523000
C	4.287985000	-5.834908000	-2.152237000
H	5.248225000	-5.428623000	-1.826351000
H	4.382865000	-6.173812000	-3.189762000
H	4.044590000	-6.705380000	-1.534641000
C	-0.133042000	3.323093000	3.413400000
C	5.018743000	-5.779045000	1.615860000
H	4.591133000	-5.987218000	0.633996000
H	5.144322000	-6.728917000	2.146348000
H	6.006278000	-5.333309000	1.474660000
C	4.234292000	0.000126000	2.935509000
H	4.245617000	-0.773444000	2.162219000
H	5.067828000	-0.203809000	3.615239000
H	4.396168000	0.982515000	2.480388000
C	1.837274000	-5.365615000	-2.408823000
H	1.595497000	-6.205691000	-1.751909000
H	1.846560000	-5.728927000	-3.442003000
H	1.046929000	-4.615961000	-2.309663000
C	2.924927000	1.001458000	4.812856000
H	3.188052000	1.983780000	4.416313000
H	3.671531000	0.727368000	5.565003000
H	1.954789000	1.071449000	5.309488000
C	7.776760000	-3.410044000	-0.932155000
H	7.821344000	-4.403039000	-0.474791000
H	8.760517000	-2.939241000	-0.835662000
H	7.555406000	-3.533470000	-1.996652000
C	7.010981000	-2.404315000	1.230090000
H	6.223508000	-1.849464000	1.744939000
H	7.953719000	-1.863117000	1.361565000
H	7.108708000	-3.381189000	1.710943000
C	2.728053000	-5.504015000	2.590174000
H	2.063259000	-4.843210000	3.154551000
H	2.806374000	-6.456678000	3.125736000
H	2.282544000	-5.687089000	1.610334000
C	6.610366000	-1.192714000	-0.933323000
H	6.387082000	-1.314204000	-1.997689000
H	7.560691000	-0.657741000	-0.840023000
H	5.827429000	-0.578282000	-0.483776000
C	4.732014000	-4.569734000	3.784888000
H	5.687808000	-4.051348000	3.665619000
H	4.911452000	-5.504742000	4.325886000

H	4.075009000	-3.943720000	4.393018000
C	4.939087000	1.533060000	-3.357309000
H	5.091004000	0.531327000	-2.947015000
H	5.097172000	1.491547000	-4.440092000
H	3.902372000	1.824576000	-3.173843000
C	7.186586000	4.323520000	0.941101000
H	7.250517000	4.086443000	-0.122843000
H	8.198715000	4.506734000	1.317374000
H	6.608382000	5.242409000	1.058863000
C	7.367510000	1.892343000	1.513636000
H	6.930389000	1.061670000	2.074611000
H	8.396307000	2.036972000	1.861368000
H	7.388139000	1.625497000	0.454929000
C	-0.939626000	3.999299000	4.523231000
H	-1.973219000	3.644748000	4.523292000
H	-0.948326000	5.084781000	4.383012000
H	-0.498105000	3.782743000	5.500617000
C	2.673999000	-1.445568000	4.254338000
H	1.708322000	-1.508306000	4.756252000
H	3.462275000	-1.694704000	4.972094000
H	2.706238000	-2.179227000	3.445299000
C	3.526717000	6.769956000	-1.072091000
H	4.585605000	7.042249000	-1.106956000
H	2.955173000	7.637978000	-0.727298000
H	3.204015000	6.524158000	-2.088312000
C	1.305671000	3.832798000	3.439618000
H	1.739382000	3.708709000	4.435125000
H	1.333592000	4.897022000	3.187728000
H	1.930935000	3.295919000	2.720805000
C	1.845207000	5.166740000	-0.130405000
H	1.500981000	4.960937000	-1.145455000
H	1.222810000	5.963828000	0.289849000
H	1.705211000	4.263014000	0.465463000
C	3.776720000	5.933492000	1.274573000
H	3.740135000	5.062481000	1.932686000
H	3.136742000	6.717889000	1.692787000
H	4.803003000	6.309983000	1.263595000
C	-1.458102000	-2.202161000	5.385841000
H	-2.078909000	-2.318527000	6.279866000
H	-0.506548000	-2.714025000	5.557062000
H	-1.959893000	-2.687355000	4.546174000
C	6.454119000	3.516329000	3.199581000
H	5.859393000	4.422839000	3.346356000
H	7.450979000	3.685897000	3.619538000
H	5.981506000	2.700976000	3.755123000
C	5.689057000	3.934352000	-3.264035000
H	4.664912000	4.275627000	-3.104476000

H	5.904290000	3.945467000	-4.338065000
H	6.357974000	4.645155000	-2.770472000
C	7.348011000	2.092585000	-2.938405000
H	8.037747000	2.791478000	-2.455369000
H	7.576331000	2.068896000	-4.008896000
H	7.522301000	1.095737000	-2.525639000
C	-0.508404000	-0.056780000	6.263303000
H	0.437499000	-0.561259000	6.481727000
H	-1.132498000	-0.113593000	7.161285000
H	-0.310995000	0.995801000	6.048617000
C	-2.548236000	-0.016772000	4.803271000
H	-2.377148000	1.043341000	4.605326000
H	-3.222336000	-0.102665000	5.661677000
H	-3.041015000	-0.467304000	3.938126000

5K-Pr^{Ph} complex, (s=1/2), dispersion

Pr	0.015881000	-0.068086000	-0.048770000
K	1.342370000	-3.126545000	1.654924000
Si	-1.114878000	-3.383742000	-1.053994000
Si	3.558815000	-0.991116000	-0.120057000
Si	0.052600000	0.876507000	3.504065000
Si	-3.577408000	0.804919000	-0.375349000
Si	1.045942000	2.834048000	-2.073597000
O	-0.360318000	-2.232460000	-0.136142000
O	2.033307000	-0.792427000	0.488993000
O	-0.178681000	0.735371000	1.874584000
O	-2.011602000	0.273047000	-0.521323000
O	0.802087000	1.343439000	-1.395194000
C	-0.072019000	-4.958159000	-0.875508000
C	-0.415505000	-5.939178000	0.070424000
H	-1.374009000	-5.870701000	0.581142000
C	0.449627000	-6.996164000	0.362066000
H	0.163128000	-7.747379000	1.093508000
C	1.675867000	-7.094524000	-0.296468000
H	2.348179000	-7.919048000	-0.075428000
C	2.028951000	-6.139087000	-1.250807000
H	2.977110000	-6.218214000	-1.776239000
C	1.165264000	-5.081467000	-1.534188000
H	1.461411000	-4.337705000	-2.269490000
C	-2.813674000	-3.690045000	-0.332133000
C	-3.140860000	-3.112687000	0.901099000
H	-2.408542000	-2.488335000	1.403289000
C	-4.401697000	-3.291796000	1.464021000
H	-4.648450000	-2.808055000	2.403355000
C	-5.357270000	-4.049019000	0.790026000
H	-6.348859000	-4.171637000	1.216538000
C	-5.049546000	-4.631595000	-0.440431000

H	-5.799240000	-5.213860000	-0.969307000
C	-3.784625000	-4.453911000	-0.995592000
H	-3.555826000	-4.892926000	-1.964471000
C	-1.202315000	-2.893309000	-2.863017000
C	-1.052293000	-3.838823000	-3.888009000
H	-0.861436000	-4.879972000	-3.638979000
C	-1.126545000	-3.459008000	-5.227972000
H	-1.006468000	-4.203805000	-6.010137000
C	-1.338667000	-2.121534000	-5.562649000
H	-1.383954000	-1.822611000	-6.606196000
C	-1.493972000	-1.167782000	-4.557022000
H	-1.662930000	-0.124745000	-4.805743000
C	-1.436288000	-1.555753000	-3.220994000
H	-1.611539000	-0.809320000	-2.450320000
C	4.317651000	-2.407970000	0.887929000
C	4.716235000	-2.193909000	2.220092000
H	4.736890000	-1.180732000	2.615861000
C	5.065163000	-3.263534000	3.044197000
H	5.371655000	-3.080885000	4.070753000
C	5.021700000	-4.570185000	2.550594000
H	5.290652000	-5.403469000	3.193990000
C	4.644774000	-4.801654000	1.227025000
H	4.612997000	-5.814999000	0.836793000
C	4.302609000	-3.726361000	0.404505000
H	4.001056000	-3.910002000	-0.623909000
C	4.609925000	0.540147000	0.089183000
C	5.985583000	0.486263000	0.354260000
H	6.475551000	-0.476726000	0.480642000
C	6.732515000	1.658199000	0.472870000
H	7.798044000	1.604618000	0.679802000
C	6.109667000	2.898782000	0.331363000
H	6.691104000	3.811540000	0.427865000
C	4.741868000	2.967698000	0.073121000
H	4.243767000	3.925712000	-0.030833000
C	4.005334000	1.795140000	-0.049787000
H	2.941910000	1.846229000	-0.247478000
C	3.466293000	-1.508497000	-1.922964000
C	4.620708000	-1.562187000	-2.716037000
H	5.586747000	-1.321086000	-2.278180000
C	4.544349000	-1.888884000	-4.068409000
H	5.448946000	-1.926625000	-4.669698000
C	3.304525000	-2.143635000	-4.656402000
H	3.242142000	-2.375187000	-5.715955000
C	2.144379000	-2.096633000	-3.884820000
H	1.174783000	-2.289615000	-4.330779000
C	2.235947000	-1.795898000	-2.527075000
H	1.328605000	-1.796888000	-1.930975000

C	0.061443000	-0.884346000	4.208056000
C	-1.044547000	-1.727351000	3.988417000
H	-1.929261000	-1.338027000	3.492515000
C	-1.023546000	-3.066604000	4.381333000
H	-1.889620000	-3.697022000	4.197361000
C	0.111432000	-3.590797000	5.009865000
H	0.129162000	-4.631082000	5.324703000
C	1.216323000	-2.767778000	5.245179000
H	2.096728000	-3.168282000	5.741154000
C	1.187969000	-1.427031000	4.847274000
H	2.055619000	-0.795328000	5.021624000
C	1.703481000	1.683581000	3.857393000
C	2.815920000	1.343093000	3.072765000
H	2.696584000	0.636901000	2.255269000
C	4.059202000	1.926594000	3.305380000
H	4.904647000	1.667485000	2.675592000
C	4.203289000	2.863342000	4.328478000
H	5.169781000	3.327534000	4.505096000
C	3.106487000	3.216806000	5.114299000
H	3.217792000	3.955123000	5.903951000
C	1.863945000	2.629326000	4.879256000
H	1.006068000	2.918774000	5.482157000
C	-1.377981000	1.875721000	4.174097000
C	-1.962439000	1.664861000	5.429247000
H	-1.556278000	0.905630000	6.094522000
C	-3.079118000	2.401520000	5.824317000
H	-3.530592000	2.225156000	6.797063000
C	-3.627400000	3.350413000	4.961985000
H	-4.510021000	3.908342000	5.262767000
C	-3.052326000	3.576924000	3.711503000
H	-3.480934000	4.300221000	3.024905000
C	-1.932148000	2.847547000	3.326097000
H	-1.507185000	3.008337000	2.340118000
C	-4.448165000	0.102196000	1.135043000
C	-3.803815000	0.138817000	2.378546000
H	-2.775698000	0.480757000	2.431589000
C	-4.471934000	-0.220287000	3.546250000
H	-3.956839000	-0.161311000	4.500325000
C	-5.809491000	-0.617393000	3.484684000
H	-6.339763000	-0.886155000	4.394525000
C	-6.464813000	-0.661934000	2.254917000
H	-7.504155000	-0.975674000	2.203658000
C	-5.785885000	-0.307354000	1.088925000
H	-6.298769000	-0.355493000	0.131828000
C	-3.587475000	2.665173000	-0.132958000
C	-2.410564000	3.417638000	-0.201137000
H	-1.474898000	2.932102000	-0.454861000

C	-2.411761000	4.783750000	0.071342000
H	-1.482724000	5.340674000	0.026934000
C	-3.606725000	5.422143000	0.399668000
H	-3.612324000	6.488545000	0.609200000
C	-4.793610000	4.690622000	0.465251000
H	-5.725464000	5.184863000	0.727250000
C	-4.778299000	3.321048000	0.212060000
H	-5.700251000	2.750692000	0.303697000
C	-4.525410000	0.277583000	-1.905197000
C	-4.570793000	-1.089758000	-2.216984000
H	-4.090008000	-1.808837000	-1.561010000
C	-5.218044000	-1.541483000	-3.362504000
H	-5.226651000	-2.602959000	-3.593053000
C	-5.846401000	-0.629301000	-4.210295000
H	-6.351318000	-0.977895000	-5.107270000
C	-5.829502000	0.731139000	-3.904325000
H	-6.324542000	1.442639000	-4.560097000
C	-5.170326000	1.180305000	-2.759343000
H	-5.145921000	2.243158000	-2.531674000
C	2.753897000	2.907928000	-2.854156000
C	3.324561000	1.743515000	-3.386226000
H	2.767332000	0.811855000	-3.366050000
C	4.613877000	1.758846000	-3.911763000
H	5.041512000	0.842328000	-4.304412000
C	5.352573000	2.940716000	-3.919038000
H	6.362454000	2.949429000	-4.320375000
C	4.796113000	4.109410000	-3.403629000
H	5.370179000	5.032256000	-3.400655000
C	3.507772000	4.088848000	-2.873790000
H	3.099541000	4.997502000	-2.438070000
C	-0.262909000	3.135782000	-3.382632000
C	-0.057180000	4.075053000	-4.402149000
H	0.876742000	4.632227000	-4.440432000
C	-1.018951000	4.283488000	-5.388956000
H	-0.844185000	5.015896000	-6.172634000
C	-2.196784000	3.537874000	-5.378086000
H	-2.944415000	3.690702000	-6.151988000
C	-2.412638000	2.590410000	-4.377722000
H	-3.324178000	2.001215000	-4.365366000
C	-1.454628000	2.396959000	-3.384893000
H	-1.631971000	1.660013000	-2.606903000
C	0.995014000	4.124403000	-0.698631000
C	1.104012000	3.742110000	0.646448000
H	1.161535000	2.692375000	0.915771000
C	1.128526000	4.688202000	1.668719000
H	1.213969000	4.359394000	2.700049000
C	1.051377000	6.045277000	1.358737000

H	1.072083000	6.787127000	2.152244000
C	0.939104000	6.448383000	0.027822000
H	0.866282000	7.504612000	-0.217306000
C	0.903073000	5.493925000	-0.988501000
H	0.780484000	5.820286000	-2.019109000

5K-Pr^{Ph} complex, (s=1/2)

Pr	0.082279000	-0.121225000	0.044064000
K	-1.915981000	3.012237000	-0.126892000
Si	0.356632000	2.249403000	-2.843355000
Si	-3.747226000	-0.075661000	-0.235602000
Si	-0.150706000	1.618983000	3.375300000
Si	3.854848000	-0.212343000	0.130149000
Si	-0.084264000	-3.894224000	-0.289413000
O	0.116027000	1.617063000	-1.327772000
O	-2.120176000	0.207712000	-0.089257000
O	0.049365000	0.685960000	2.019355000
O	2.200437000	-0.252004000	-0.105867000
O	-0.160560000	-2.235190000	-0.130030000
C	-1.040736000	3.512763000	-3.189222000
C	-0.830353000	4.886732000	-2.969132000
H	0.154215000	5.232191000	-2.661639000
C	-1.849673000	5.821214000	-3.162276000
H	-1.655657000	6.877695000	-2.994127000
C	-3.107575000	5.401700000	-3.595235000
H	-3.898386000	6.128341000	-3.762515000
C	-3.339459000	4.045798000	-3.827817000
H	-4.311762000	3.711792000	-4.180907000
C	-2.320100000	3.114787000	-3.620707000
H	-2.523887000	2.063765000	-3.811780000
C	1.975540000	3.219380000	-2.955020000
C	2.623989000	3.696795000	-1.806735000
H	2.244856000	3.423627000	-0.826010000
C	3.766188000	4.493024000	-1.901544000
H	4.260377000	4.834480000	-0.996988000
C	4.282134000	4.822294000	-3.153696000
H	5.173994000	5.438998000	-3.230447000
C	3.660290000	4.346224000	-4.308431000
H	4.066661000	4.590634000	-5.286689000
C	2.519559000	3.552654000	-4.207201000
H	2.050339000	3.181776000	-5.116303000
C	0.326648000	0.904484000	-4.174306000
C	-0.192779000	1.128955000	-5.460123000
H	-0.639916000	2.090352000	-5.702694000
C	-0.152051000	0.135171000	-6.439167000
H	-0.557222000	0.332932000	-7.428542000
C	0.404520000	-1.109665000	-6.146907000

H	0.432806000	-1.886639000	-6.906401000
C	0.930264000	-1.354095000	-4.878348000
H	1.373590000	-2.316909000	-4.641328000
C	0.894525000	-0.353384000	-3.908185000
H	1.343567000	-0.555333000	-2.937394000
C	-4.679789000	1.554028000	0.153295000
C	-4.833559000	1.985899000	1.484975000
H	-4.462407000	1.365062000	2.297529000
C	-5.473129000	3.188117000	1.789744000
H	-5.592552000	3.489043000	2.827493000
C	-5.972349000	3.994006000	0.765285000
H	-6.478255000	4.926844000	1.000714000
C	-5.834537000	3.587070000	-0.561515000
H	-6.233580000	4.202384000	-1.363781000
C	-5.198850000	2.379314000	-0.859539000
H	-5.118513000	2.063635000	-1.896828000
C	-4.418651000	-1.374830000	0.963319000
C	-5.745966000	-1.329145000	1.421514000
H	-6.388498000	-0.497677000	1.139864000
C	-6.259657000	-2.329068000	2.247051000
H	-7.290503000	-2.274606000	2.588208000
C	-5.448488000	-3.394607000	2.637204000
H	-5.845754000	-4.175675000	3.280479000
C	-4.126295000	-3.455507000	2.197316000
H	-3.488351000	-4.284273000	2.490428000
C	-3.621161000	-2.456372000	1.366460000
H	-2.595472000	-2.521164000	1.015598000
C	-4.181558000	-0.551730000	-2.018621000
C	-5.494577000	-0.906646000	-2.368864000
H	-6.269013000	-0.937730000	-1.605164000
C	-5.828673000	-1.236035000	-3.681441000
H	-6.851870000	-1.507732000	-3.928824000
C	-4.848225000	-1.224796000	-4.674744000
H	-5.105801000	-1.485308000	-5.698222000
C	-3.535986000	-0.884472000	-4.348188000
H	-2.761737000	-0.880286000	-5.110384000
C	-3.212132000	-0.548655000	-3.031977000
H	-2.184314000	-0.289855000	-2.790215000
C	-0.538393000	3.403376000	2.806437000
C	0.386906000	4.102046000	2.006446000
H	1.342383000	3.644772000	1.760458000
C	0.108226000	5.379505000	1.517419000
H	0.844311000	5.898317000	0.908777000
C	-1.110110000	5.993481000	1.820536000
H	-1.326798000	6.991235000	1.447790000
C	-2.038727000	5.325075000	2.619066000
H	-2.983226000	5.800922000	2.869716000

C	-1.752014000	4.045759000	3.105303000
H	-2.483424000	3.539501000	3.730493000
C	-1.588898000	0.992632000	4.429385000
C	-2.475858000	0.021383000	3.943649000
H	-2.332102000	-0.384862000	2.946884000
C	-3.538492000	-0.441315000	4.721609000
H	-4.206450000	-1.199140000	4.321708000
C	-3.732327000	0.068318000	6.004852000
H	-4.558082000	-0.289879000	6.614456000
C	-2.857325000	1.030760000	6.509986000
H	-2.997628000	1.421467000	7.514763000
C	-1.794208000	1.482832000	5.730121000
H	-1.106849000	2.215775000	6.148279000
C	1.408901000	1.645871000	4.438445000
C	1.770662000	2.770561000	5.197604000
H	1.182860000	3.683867000	5.126264000
C	2.881317000	2.741942000	6.041663000
H	3.144890000	3.622360000	6.622578000
C	3.655809000	1.586325000	6.133108000
H	4.526244000	1.564778000	6.783731000
C	3.317037000	0.461229000	5.381696000
H	3.925023000	-0.437323000	5.434233000
C	2.201564000	0.491540000	4.546985000
H	1.956627000	-0.389301000	3.959076000
C	4.490966000	1.499156000	0.652516000
C	3.866577000	2.201473000	1.695871000
H	2.967946000	1.796115000	2.152025000
C	4.392772000	3.396619000	2.183952000
H	3.896578000	3.907529000	3.004939000
C	5.570330000	3.912805000	1.639991000
H	5.992402000	4.836784000	2.027844000
C	6.208043000	3.230816000	0.605097000
H	7.126522000	3.625055000	0.177006000
C	5.669484000	2.039234000	0.115547000
H	6.175771000	1.523463000	-0.696232000
C	4.330605000	-1.413556000	1.513348000
C	3.466305000	-2.439335000	1.921980000
H	2.494423000	-2.544280000	1.448237000
C	3.827065000	-3.328487000	2.934946000
H	3.137340000	-4.113095000	3.232250000
C	5.070141000	-3.208145000	3.555135000
H	5.356552000	-3.902497000	4.341310000
C	5.945053000	-2.193467000	3.165165000
H	6.913447000	-2.091834000	3.649144000
C	5.573632000	-1.303303000	2.158704000
H	6.257650000	-0.503985000	1.881246000
C	4.771371000	-0.679747000	-1.465509000

C	4.517826000	0.014842000	-2.661287000
H	3.762866000	0.796875000	-2.684965000
C	5.228677000	-0.268702000	-3.826203000
H	5.011288000	0.281327000	-4.737948000
C	6.220286000	-1.250239000	-3.817630000
H	6.780286000	-1.468318000	-4.723613000
C	6.490960000	-1.948601000	-2.641647000
H	7.261684000	-2.715251000	-2.627512000
C	5.768905000	-1.667545000	-1.480360000
H	5.985028000	-2.225801000	-0.572903000
C	-1.754296000	-4.615369000	-0.824850000
C	-2.468501000	-4.017659000	-1.875545000
H	-2.091501000	-3.109301000	-2.337397000
C	-3.665347000	-4.559679000	-2.338821000
H	-4.203401000	-4.067062000	-3.143889000
C	-4.169263000	-5.725955000	-1.762998000
H	-5.102361000	-6.152931000	-2.122068000
C	-3.474198000	-6.340072000	-0.722932000
H	-3.862997000	-7.248614000	-0.269415000
C	-2.280497000	-5.786668000	-0.257692000
H	-1.753481000	-6.274423000	0.559271000
C	1.165912000	-4.398650000	-1.617751000
C	0.991675000	-5.595216000	-2.333185000
H	0.119737000	-6.216163000	-2.140174000
C	1.907620000	-5.995260000	-3.305471000
H	1.752690000	-6.925613000	-3.846425000
C	3.013337000	-5.195256000	-3.590391000
H	3.726512000	-5.501120000	-4.351974000
C	3.201328000	-3.998866000	-2.897633000
H	4.058904000	-3.368100000	-3.112555000
C	2.286671000	-3.609804000	-1.918430000
H	2.445005000	-2.674833000	-1.387701000
C	0.368576000	-4.671339000	1.382378000
C	-0.057128000	-4.085682000	2.586025000
H	-0.582682000	-3.134096000	2.565071000
C	0.183837000	-4.699687000	3.814885000
H	-0.153688000	-4.225450000	4.732922000
C	0.858636000	-5.920017000	3.863648000
H	1.044419000	-6.403074000	4.819609000
C	1.300233000	-6.513367000	2.681244000
H	1.836235000	-7.458643000	2.713246000
C	1.059355000	-5.891377000	1.455706000
H	1.420335000	-6.359985000	0.542902000

5Cs-Pr^{Ph} complex, (s=1/2), dispersion

Pr	-0.017276000	-0.110892000	0.042627000
Cs	1.809593000	-3.014666000	-1.688822000
Si	3.649313000	0.247567000	0.151636000
Si	0.210012000	3.218072000	1.659791000
Si	-3.765870000	0.193615000	0.093344000
Si	-0.277252000	-3.173399000	2.033652000
Si	-0.242613000	0.114675000	-3.602292000
O	-2.111028000	0.166448000	0.043017000
O	-0.189383000	-2.148627000	0.735157000
O	0.312366000	1.834618000	0.756874000
O	0.129623000	-0.392087000	-2.073127000
O	2.143217000	-0.435236000	0.110967000
C	-0.482987000	2.795490000	-2.590849000
H	-0.626565000	2.354695000	-1.609071000
C	2.185392000	-2.110791000	3.039137000
H	2.411394000	-1.882599000	2.000609000
C	1.562847000	-2.565371000	5.717468000
H	1.314152000	-2.731938000	6.762269000
C	4.137162000	0.705140000	1.902725000
C	0.815445000	-1.772059000	-5.495074000
H	-0.199369000	-2.163350000	-5.530238000
C	4.745954000	1.385092000	4.559583000
H	4.981455000	1.638273000	5.589796000
C	-4.421678000	1.387676000	-1.201602000
C	5.446893000	0.586096000	2.389083000
H	6.233166000	0.209054000	1.738976000
C	-2.527067000	-1.535136000	-3.223620000
H	-2.010266000	-1.871195000	-2.329250000
C	1.074471000	-0.608691000	-4.752164000
C	-1.038938000	5.706209000	0.991921000
H	-0.173556000	6.167414000	1.461796000
C	3.148110000	1.177707000	2.771634000
H	2.133328000	1.271426000	2.406768000
C	5.280222000	-1.089431000	-1.818773000
H	5.032506000	-0.246432000	-2.459428000
C	-4.480580000	-1.509612000	-0.241949000
C	1.811030000	4.194130000	1.599255000
C	2.587748000	4.460511000	2.732612000
H	2.261630000	4.097361000	3.702359000
C	3.803603000	1.717348000	-1.001621000
C	-3.223651000	4.536564000	-0.286591000
H	-4.062178000	4.074589000	-0.794724000
C	-1.995466000	-3.184456000	2.770497000
C	-4.305584000	0.778924000	1.793598000
C	2.254487000	4.674520000	0.357420000
H	1.673164000	4.476593000	-0.539025000

C	-3.371989000	1.400988000	2.629669000
H	-2.351958000	1.528250000	2.286075000
C	0.943118000	-2.673904000	3.367705000
C	-3.620480000	1.696740000	-2.304643000
H	-2.640486000	1.247816000	-2.382847000
C	4.850024000	-1.082400000	-0.481279000
C	-0.129371000	2.657477000	3.432348000
C	-3.707042000	-2.658368000	-0.041804000
H	-2.677772000	-2.552455000	0.284780000
C	-5.613956000	0.610758000	2.265329000
H	-6.348573000	0.093605000	1.651988000
C	2.389243000	-0.110771000	-4.736146000
H	2.617930000	0.788827000	-4.171660000
C	-2.632954000	-0.121836000	-5.171722000
H	-2.193292000	0.635877000	-5.816682000
C	-4.607602000	-3.045327000	3.780476000
H	-5.624887000	-2.987178000	4.157418000
C	-3.805782000	-2.016368000	-3.496124000
H	-4.265471000	-2.738323000	-2.829551000
C	-2.243835000	3.738565000	0.297572000
H	-2.319190000	2.659731000	0.217178000
C	-3.721694000	1.841118000	3.903697000
H	-2.972059000	2.306872000	4.535666000
C	-2.523916000	-1.970153000	3.234259000
H	-1.928810000	-1.061118000	3.193834000
C	0.643964000	-2.892243000	4.721512000
H	-0.322889000	-3.308342000	4.994197000
C	2.797752000	-2.017791000	5.370754000
H	3.513108000	-1.750990000	6.143584000
C	-5.802531000	-1.660832000	-0.681375000
H	-6.407201000	-0.779820000	-0.884043000
C	-0.532142000	4.183463000	-2.692858000
H	-0.723111000	4.780212000	-1.806734000
C	-5.682519000	1.993852000	-1.107545000
H	-6.310487000	1.807914000	-0.239295000
C	-0.397547000	3.581752000	4.451721000
H	-0.455454000	4.641515000	4.213538000
C	0.169012000	-4.885322000	1.361795000
C	-0.343046000	4.792328000	-3.932852000
H	-0.379889000	5.874894000	-4.016221000
C	-0.245866000	1.989957000	-3.715110000
C	-3.817364000	-1.897083000	3.739173000
H	-4.208853000	-0.947160000	4.085518000
C	5.918463000	-3.256228000	-0.184587000
H	6.176362000	-4.094139000	0.458186000
C	-2.794984000	-4.331669000	2.842157000
H	-2.402817000	-5.283189000	2.491758000

C	-5.979366000	1.063321000	3.531959000
H	-6.997602000	0.924643000	3.885824000
C	-1.922195000	-0.583047000	-4.055058000
C	-0.102932000	4.011995000	-5.063973000
H	0.052489000	4.484918000	-6.029918000
C	5.161738000	3.504869000	-1.931465000
H	6.086777000	4.075105000	-1.935599000
C	3.440499000	5.394506000	0.253972000
H	3.770282000	5.752187000	-0.715533000
C	-4.500127000	-1.540984000	-4.606555000
H	-5.505496000	-1.899186000	-4.809636000
C	3.439545000	1.508373000	4.090741000
H	2.642855000	1.843211000	4.748210000
C	-5.565205000	-4.061943000	-0.686731000
H	-5.984590000	-5.050351000	-0.854325000
C	-4.048667000	2.579601000	-3.290485000
H	-3.390630000	2.813359000	-4.122164000
C	4.988434000	2.466658000	-1.020815000
H	5.781708000	2.240239000	-0.311810000
C	-0.548367000	-5.403771000	0.267629000
H	-1.392081000	-4.844029000	-0.130870000
C	-4.095835000	-4.263186000	3.337811000
H	-4.712330000	-5.157347000	3.370569000
C	5.191407000	-2.181332000	0.327745000
H	4.877723000	-2.197523000	1.369403000
C	-3.913454000	-0.593900000	-5.446075000
H	-4.460521000	-0.214133000	-6.304629000
C	-4.244898000	-3.925117000	-0.258465000
H	-3.641616000	-4.809758000	-0.075267000
C	-6.129294000	2.866259000	-2.099540000
H	-7.107929000	3.330868000	-2.012449000
C	2.788840000	2.053620000	-1.902618000
H	1.866237000	1.483853000	-1.902993000
C	0.910314000	-7.325652000	0.161282000
H	1.193792000	-8.269309000	-0.296939000
C	4.139656000	3.823332000	-2.826890000
H	4.269832000	4.638153000	-3.534208000
C	3.109331000	-1.790428000	4.031730000
H	4.057790000	-1.335198000	3.765280000
C	5.751622000	0.927381000	3.706889000
H	6.770559000	0.827890000	4.071564000
C	-1.140268000	4.307839000	0.948012000
C	-0.186428000	-6.611725000	-0.327840000
H	-0.760080000	-6.999963000	-1.165913000
C	4.208995000	5.638363000	1.391956000
H	5.140409000	6.192468000	1.309973000
C	-0.052853000	2.622655000	-4.952458000

H	0.157117000	2.024108000	-5.836508000
C	-5.031702000	1.675932000	4.353853000
H	-5.313415000	2.018341000	5.345974000
C	-6.344977000	-2.925379000	-0.900615000
H	-7.370729000	-3.025512000	-1.245401000
C	1.263666000	-5.620271000	1.838700000
H	1.834230000	-5.235576000	2.680599000
C	2.945812000	3.104909000	-2.805972000
H	2.140177000	3.356524000	-3.487835000
C	-0.593835000	3.164885000	5.768287000
H	-0.797674000	3.897821000	6.544220000
C	-3.109702000	5.924734000	-0.228689000
H	-3.869139000	6.549579000	-0.691215000
C	6.004848000	-2.163956000	-2.338154000
H	6.326276000	-2.151357000	-3.376434000
C	3.782058000	5.171164000	2.632835000
H	4.379662000	5.354712000	3.521501000
C	6.320954000	-3.251521000	-1.522269000
H	6.889796000	-4.086511000	-1.922629000
C	-5.311218000	3.161781000	-3.191492000
H	-5.654785000	3.854114000	-3.955397000
C	1.633750000	-6.828980000	1.245235000
H	2.484085000	-7.385116000	1.630944000
C	3.139472000	-1.929517000	-6.140337000
H	3.936040000	-2.437963000	-6.677105000
C	-0.532404000	1.808024000	6.088388000
H	-0.685445000	1.483127000	7.113717000
C	1.837479000	-2.429765000	-6.183116000
H	1.617322000	-3.324931000	-6.759405000
C	3.412690000	-0.764836000	-5.420289000
H	4.422376000	-0.363436000	-5.395306000
C	-2.017041000	6.511081000	0.410057000
H	-1.921873000	7.593236000	0.445747000
C	-0.082028000	1.299782000	3.779441000
H	0.130733000	0.552096000	3.019450000
C	-0.279168000	0.869458000	5.088573000
H	-0.223459000	-0.189741000	5.320253000

5Cs-Pr^{Ph} complex, (s=1/2)

Pr	-0.159396000	0.157992000	0.010865000
Cs	2.517855000	-2.895541000	0.146435000
Si	3.572951000	1.037830000	-0.104467000
Si	-0.874884000	3.866847000	-0.351360000
Si	-3.872882000	-0.582620000	0.033070000
Si	0.200502000	-1.395503000	3.469074000
Si	0.182752000	-2.050834000	-3.019712000
O	-2.256862000	-0.205584000	-0.153846000

O	0.014919000	-0.748259000	1.955057000
O	-0.439267000	2.269139000	-0.173303000
O	0.209480000	-1.403330000	-1.494842000
O	2.035285000	0.431839000	0.005068000
C	-0.768400000	0.420982000	-4.111921000
H	-1.270976000	0.545572000	-3.155324000
C	2.168917000	0.659781000	3.912021000
H	2.020967000	0.904760000	2.863614000
C	2.554445000	0.063184000	6.604504000
H	2.695820000	-0.168177000	7.657357000
C	3.911347000	2.475654000	1.078425000
C	1.819401000	-4.438734000	-3.168334000
H	0.890087000	-4.966869000	-2.965686000
C	4.423849000	4.703942000	2.725887000
H	4.620768000	5.564296000	3.360501000
C	-4.798829000	-0.346428000	-1.609020000
C	5.196514000	2.715926000	1.592905000
H	6.008326000	2.028295000	1.365417000
C	-1.795943000	-3.959656000	-2.132317000
H	-1.531218000	-3.649873000	-1.124607000
C	1.799515000	-3.038916000	-3.308187000
C	-2.357248000	5.284145000	-2.361250000
H	-1.691031000	6.113989000	-2.134642000
C	2.891063000	3.379260000	1.410773000
H	1.892090000	3.221974000	1.015518000
C	5.575259000	-1.017648000	-0.628156000
H	5.481678000	-0.720645000	-1.669905000
C	-4.177539000	-2.378946000	0.581331000
C	0.590904000	4.943997000	-0.889412000
C	0.830089000	6.204997000	-0.321148000
H	0.197940000	6.557653000	0.490574000
C	3.962347000	1.575618000	-1.880259000
C	-4.031721000	3.158378000	-3.018510000
H	-4.678053000	2.322940000	-3.270523000
C	-1.416227000	-1.467443000	4.444107000
C	-4.655952000	0.536275000	1.344197000
C	1.433293000	4.523455000	-1.930937000
H	1.278452000	3.551967000	-2.392492000
C	-4.093460000	1.779575000	1.666973000
H	-3.166874000	2.089241000	1.192248000
C	1.445806000	-0.396952000	4.483008000
C	-4.270413000	-0.842688000	-2.812581000
H	-3.299236000	-1.330019000	-2.816267000
C	4.830140000	-0.337462000	0.349917000
C	-1.487725000	4.539582000	1.316411000
C	-3.642221000	-2.860728000	1.788430000
H	-3.002179000	-2.220757000	2.389262000

C	-5.847258000	0.165194000	1.989733000
H	-6.297104000	-0.802545000	1.778541000
C	3.017157000	-2.401120000	-3.607982000
H	3.044270000	-1.321751000	-3.735928000
C	-1.617033000	-3.705983000	-4.518653000
H	-1.194846000	-3.210974000	-5.390813000
C	-3.756909000	-1.462275000	6.009876000
H	-4.663369000	-1.462444000	6.609692000
C	-2.735505000	-4.976556000	-2.309246000
H	-3.184909000	-5.451251000	-1.442196000
C	-3.056280000	3.004686000	-2.032690000
H	-2.954426000	2.044878000	-1.533196000
C	-4.697769000	2.628829000	2.594222000
H	-4.239260000	3.585865000	2.825573000
C	-2.316917000	-0.390843000	4.395296000
H	-2.121424000	0.451280000	3.736739000
C	1.648498000	-0.675549000	5.845423000
H	1.080373000	-1.469645000	6.326432000
C	3.267586000	1.107858000	6.015352000
H	3.968351000	1.690737000	6.607772000
C	-5.032196000	-3.229560000	-0.136685000
H	-5.467276000	-2.888507000	-1.072211000
C	-0.954756000	1.395696000	-5.091035000
H	-1.576502000	2.260239000	-4.876798000
C	-6.061709000	0.265957000	-1.651879000
H	-6.492623000	0.670849000	-0.739655000
C	-2.418708000	5.588413000	1.386846000
H	-2.862799000	5.976195000	0.472600000
C	0.853246000	-3.182380000	3.272070000
C	-0.349993000	1.249808000	-6.339565000
H	-0.495210000	2.006227000	-7.106452000
C	0.027654000	-0.712471000	-4.348792000
C	-3.478589000	-0.388096000	5.164935000
H	-4.168922000	0.447002000	5.092151000
C	5.868557000	-1.773966000	2.034351000
H	5.991653000	-2.052024000	3.078235000
C	-1.717377000	-2.541245000	5.297942000
H	-1.044317000	-3.394411000	5.352330000
C	-6.463243000	1.012796000	2.909831000
H	-7.387674000	0.707561000	3.394221000
C	-1.211500000	-3.313315000	-3.231664000
C	0.438539000	0.129745000	-6.601341000
H	0.909506000	0.009464000	-7.573894000
C	5.463451000	2.616501000	-3.495040000
H	6.398641000	3.127831000	-3.709462000
C	2.479870000	5.324457000	-2.382858000
H	3.125720000	4.967047000	-3.179974000

C	-3.113877000	-5.359251000	-3.594973000
H	-3.848535000	-6.148129000	-3.734850000
C	3.140126000	4.480617000	2.228388000
H	2.331927000	5.164996000	2.469419000
C	-4.791684000	-4.970017000	1.524064000
H	-5.036033000	-5.963694000	1.891882000
C	-4.975027000	-0.734525000	-4.011087000
H	-4.541999000	-1.125995000	-4.927700000
C	5.162521000	2.236608000	-2.187952000
H	5.870046000	2.470924000	-1.395268000
C	0.085836000	-4.132638000	2.571267000
H	-0.897178000	-3.859844000	2.192952000
C	-2.874320000	-2.539130000	6.077718000
H	-3.088825000	-3.379865000	6.732931000
C	5.003311000	-0.734569000	1.689615000
H	4.460023000	-0.219046000	2.478589000
C	-2.557889000	-4.717260000	-4.701712000
H	-2.859482000	-5.003082000	-5.706311000
C	-3.938707000	-4.140916000	2.254405000
H	-3.520273000	-4.478462000	3.199080000
C	-6.774716000	0.371287000	-2.846789000
H	-7.751359000	0.848901000	-2.854088000
C	3.068219000	1.320111000	-2.929428000
H	2.125291000	0.822824000	-2.718138000
C	1.813290000	-5.806191000	2.831514000
H	2.178263000	-6.817468000	2.670113000
C	4.560386000	2.348851000	-4.525116000
H	4.791290000	2.648425000	-5.544313000
C	3.070918000	1.410476000	4.668669000
H	3.611777000	2.229670000	4.203542000
C	5.454185000	3.819996000	2.405623000
H	6.456664000	3.987531000	2.791715000
C	-2.203575000	4.062488000	-1.684338000
C	0.556960000	-5.427852000	2.350980000
H	-0.060795000	-6.145187000	1.816007000
C	2.698431000	6.575573000	-1.805922000
H	3.514039000	7.202974000	-2.156749000
C	0.626461000	-0.838221000	-5.613416000
H	1.252294000	-1.700763000	-5.831890000
C	-5.888570000	2.247656000	3.212523000
H	-6.367049000	2.910656000	3.929369000
C	-5.340852000	-4.509568000	0.328941000
H	-6.014207000	-5.144283000	-0.242222000
C	2.108933000	-3.588978000	3.753284000
H	2.721036000	-2.882071000	4.308860000
C	3.357310000	1.703486000	-4.240644000
H	2.640759000	1.499017000	-5.031353000

C	-2.795628000	6.140731000	2.611892000
H	-3.516764000	6.953842000	2.640540000
C	-4.175343000	4.383429000	-3.669831000
H	-4.936929000	4.506481000	-4.436016000
C	6.442338000	-2.060104000	-0.291285000
H	7.012651000	-2.564236000	-1.067615000
C	1.870364000	7.016458000	-0.775539000
H	2.036812000	7.990036000	-0.320842000
C	6.588097000	-2.443381000	1.042126000
H	7.270350000	-3.246662000	1.308942000
C	-6.231952000	-0.129420000	-4.029737000
H	-6.784745000	-0.047260000	-4.962299000
C	2.587255000	-4.884612000	3.537552000
H	3.558519000	-5.176922000	3.929563000
C	4.197399000	-4.512666000	-3.597553000
H	5.117093000	-5.079354000	-3.719010000
C	-2.252653000	5.645528000	3.797325000
H	-2.545616000	6.073002000	4.752893000
C	3.002049000	-5.169245000	-3.303131000
H	2.986016000	-6.251744000	-3.200109000
C	4.200779000	-3.126159000	-3.755266000
H	5.124033000	-2.608417000	-4.002791000
C	-3.337353000	5.448118000	-3.339314000
H	-3.439948000	6.402625000	-3.849887000
C	-0.960159000	4.051352000	2.523220000
H	-0.244955000	3.232504000	2.506155000
C	-1.336731000	4.594302000	3.751380000
H	-0.915757000	4.196219000	4.671108000

$[Pr(OSi(Ph)_3)_5]^-$ ($[5\text{-}Pr^{Ph}]^-$)

Pr	0.019099000	0.081986000	0.043155000
Si	-1.163160000	3.255853000	1.372173000
Si	3.447253000	1.218985000	-0.105086000
Si	-0.043865000	-0.449457000	-3.486342000
Si	-3.476980000	-0.948430000	0.178178000
Si	1.160226000	-2.837351000	1.961479000
O	-0.645701000	2.091009000	0.340376000
O	1.924756000	0.845539000	-0.601454000
O	-0.365603000	-0.680957000	-1.888073000
O	-1.960159000	-0.497979000	0.633679000
O	0.943718000	-1.320003000	1.374815000
C	-0.053669000	4.743687000	1.074962000
C	-0.296324000	6.025277000	1.586564000
H	-1.189113000	6.209293000	2.181661000
C	0.582386000	7.075867000	1.326728000
H	0.382034000	8.065425000	1.730837000
C	1.714585000	6.857895000	0.540653000

H	2.399419000	7.677398000	0.336150000
C	1.965342000	5.592423000	0.011527000
H	2.839551000	5.414697000	-0.606721000
C	1.086031000	4.545371000	0.279577000
H	1.274814000	3.561401000	-0.140106000
C	-2.983736000	3.620290000	1.075200000
C	-3.540239000	3.366184000	-0.184179000
H	-2.904586000	3.009972000	-0.988031000
C	-4.906886000	3.513020000	-0.408852000
H	-5.317899000	3.274600000	-1.384186000
C	-5.739135000	3.925179000	0.630140000
H	-6.808863000	4.022517000	0.463297000
C	-5.201802000	4.196142000	1.888929000
H	-5.851436000	4.510039000	2.702483000
C	-3.834958000	4.037353000	2.109255000
H	-3.428044000	4.203582000	3.104357000
C	-1.058166000	2.655984000	3.157959000
C	-0.747987000	3.501276000	4.231281000
H	-0.487435000	4.539718000	4.039589000
C	-0.739289000	3.020725000	5.541606000
H	-0.494395000	3.689232000	6.363476000
C	-1.017107000	1.676985000	5.792895000
H	-0.991940000	1.297122000	6.811049000
C	-1.321351000	0.819277000	4.735538000
H	-1.533699000	-0.230252000	4.917627000
C	-1.355687000	1.310749000	3.432608000
H	-1.631074000	0.639248000	2.621557000
C	4.049739000	2.743457000	-1.028037000
C	3.491840000	3.065769000	-2.273158000
H	2.689809000	2.454140000	-2.676113000
C	3.939939000	4.175921000	-2.986929000
H	3.488191000	4.410622000	-3.945228000
C	4.950612000	4.982770000	-2.465490000
H	5.295857000	5.851386000	-3.020917000
C	5.509065000	4.680808000	-1.223247000
H	6.286254000	5.315762000	-0.805099000
C	5.057241000	3.570688000	-0.511998000
H	5.478439000	3.356022000	0.467502000
C	4.614618000	-0.222129000	-0.429489000
C	5.868869000	-0.109267000	-1.040444000
H	6.200990000	0.858357000	-1.410121000
C	6.692716000	-1.227598000	-1.184036000
H	7.665155000	-1.128036000	-1.660483000
C	6.265548000	-2.473843000	-0.724245000
H	6.906667000	-3.344218000	-0.839797000
C	5.013754000	-2.603811000	-0.121655000
H	4.666917000	-3.566382000	0.240536000

C	4.203392000	-1.483545000	0.024487000
H	3.233835000	-1.577574000	0.501526000
C	3.505263000	1.514674000	1.752460000
C	4.710867000	1.384036000	2.455171000
H	5.616298000	1.106766000	1.919753000
C	4.759618000	1.568769000	3.835546000
H	5.706211000	1.476672000	4.363077000
C	3.588166000	1.839094000	4.542433000
H	3.616697000	1.951766000	5.623166000
C	2.376435000	1.956671000	3.862882000
H	1.458562000	2.157159000	4.403025000
C	2.346388000	1.817761000	2.477473000
H	1.401541000	1.949282000	1.957743000
C	0.282880000	1.379379000	-3.822634000
C	-0.175895000	2.379341000	-2.952285000
H	-0.695529000	2.123040000	-2.034379000
C	0.053945000	3.727727000	-3.214945000
H	-0.280337000	4.477443000	-2.503959000
C	0.731517000	4.103837000	-4.373741000
H	0.910673000	5.155477000	-4.581726000
C	1.201802000	3.125949000	-5.250200000
H	1.748469000	3.413329000	-6.145153000
C	0.988746000	1.776918000	-4.967243000
H	1.390663000	1.019397000	-5.637144000
C	1.473066000	-1.438147000	-3.984060000
C	2.652193000	-1.255628000	-3.245037000
H	2.662326000	-0.541076000	-2.426437000
C	3.796285000	-1.996771000	-3.526787000
H	4.691363000	-1.852559000	-2.930674000
C	3.777059000	-2.927328000	-4.566809000
H	4.667133000	-3.511583000	-4.787099000
C	2.616218000	-3.115921000	-5.317097000
H	2.601075000	-3.845860000	-6.123010000
C	1.469280000	-2.377103000	-5.022642000
H	0.557855000	-2.538206000	-5.593871000
C	-1.559036000	-0.984978000	-4.462984000
C	-1.918888000	-0.383947000	-5.675888000
H	-1.279621000	0.384728000	-6.105103000
C	-3.106428000	-0.726664000	-6.320175000
H	-3.378364000	-0.241619000	-7.254666000
C	-3.954691000	-1.676704000	-5.752537000
H	-4.890699000	-1.931936000	-6.243348000
C	-3.608200000	-2.288421000	-4.548933000
H	-4.267598000	-3.017258000	-4.088305000
C	-2.419257000	-1.945534000	-3.910570000
H	-2.175670000	-2.399847000	-2.954882000
C	-4.223911000	0.085143000	-1.207506000

C	-3.424984000	0.642209000	-2.211496000
H	-2.349283000	0.535218000	-2.145076000
C	-3.985120000	1.302604000	-3.300899000
H	-3.337273000	1.715562000	-4.067813000
C	-5.371788000	1.396753000	-3.413848000
H	-5.815554000	1.895573000	-4.271745000
C	-6.187491000	0.848446000	-2.423404000
H	-7.269043000	0.929076000	-2.502497000
C	-5.614934000	0.208502000	-1.324977000
H	-6.256865000	-0.192120000	-0.543370000
C	-3.407524000	-2.716455000	-0.457689000
C	-2.201873000	-3.424731000	-0.395132000
H	-1.341119000	-2.973205000	0.084809000
C	-2.076957000	-4.688298000	-0.968182000
H	-1.124852000	-5.204710000	-0.924705000
C	-3.173262000	-5.270691000	-1.601514000
H	-3.079001000	-6.254044000	-2.055450000
C	-4.389125000	-4.587890000	-1.660617000
H	-5.244645000	-5.039758000	-2.157426000
C	-4.498991000	-3.316153000	-1.101566000
H	-5.436755000	-2.771855000	-1.191520000
C	-4.611071000	-0.782891000	1.669783000
C	-4.734208000	0.483327000	2.263338000
H	-4.215800000	1.335980000	1.833501000
C	-5.510793000	0.664244000	3.403729000
H	-5.582609000	1.651289000	3.851707000
C	-6.189171000	-0.419518000	3.963184000
H	-6.794713000	-0.281314000	4.855655000
C	-6.090855000	-1.680524000	3.376111000
H	-6.618915000	-2.526290000	3.809883000
C	-5.302754000	-1.858867000	2.238035000
H	-5.208955000	-2.847126000	1.793968000
C	2.837858000	-2.970061000	2.812706000
C	3.413831000	-1.837208000	3.405103000
H	2.896257000	-0.883023000	3.359831000
C	4.659661000	-1.913741000	4.022988000
H	5.090825000	-1.020381000	4.462536000
C	5.351723000	-3.123200000	4.060119000
H	6.327683000	-3.178316000	4.536222000
C	4.795535000	-4.258189000	3.473168000
H	5.336099000	-5.201626000	3.487195000
C	3.550193000	-4.176931000	2.852228000
H	3.138424000	-5.059605000	2.367881000
C	-0.187426000	-3.266982000	3.202422000
C	0.018878000	-4.213897000	4.214813000
H	0.983995000	-4.709882000	4.298518000
C	-0.984732000	-4.507199000	5.137150000

H	-0.807393000	-5.243209000	5.917838000
C	-2.209077000	-3.843435000	5.066779000
H	-2.990335000	-4.063787000	5.790308000
C	-2.429065000	-2.891831000	4.070792000
H	-3.378057000	-2.367744000	4.008771000
C	-1.426523000	-2.610810000	3.144135000
H	-1.606192000	-1.867623000	2.372205000
C	1.190415000	-4.067049000	0.529679000
C	1.476865000	-3.622117000	-0.768898000
H	1.606765000	-2.563659000	-0.966805000
C	1.601071000	-4.515662000	-1.830480000
H	1.817647000	-4.138614000	-2.824862000
C	1.449828000	-5.883117000	-1.603458000
H	1.549885000	-6.585146000	-2.427345000
C	1.154248000	-6.347876000	-0.320942000
H	1.016740000	-7.411991000	-0.144772000
C	1.014835000	-5.443790000	0.731906000
H	0.751107000	-5.811518000	1.721514000

$[5\text{-}Pr^{Ph}]^{-}$ no dispersion

Pr	-0.008507000	-0.027702000	-0.032962000
Si	-0.120925000	2.280305000	-3.036339000
Si	3.783940000	-0.213838000	-0.291979000
Si	-0.242166000	-3.806015000	-0.346446000
Si	-3.789181000	0.096592000	0.256407000
Si	0.341044000	1.636896000	3.356918000
O	-0.027623000	1.208344000	-1.784023000
O	2.138199000	-0.332934000	-0.205909000
O	-0.334628000	-2.159563000	-0.303366000
O	-2.143592000	0.233600000	0.224888000
O	0.307855000	0.972182000	1.849067000
C	1.082965000	1.791289000	-4.416556000
C	0.964449000	2.314966000	-5.714690000
H	0.148393000	2.995963000	-5.949960000
C	1.862207000	1.960189000	-6.720514000
H	1.752509000	2.378400000	-7.718761000
C	2.891737000	1.058866000	-6.448082000
H	3.590770000	0.776675000	-7.232183000
C	3.015818000	0.512759000	-5.171383000
H	3.806619000	-0.198581000	-4.949940000
C	2.118808000	0.879427000	-4.167875000
H	2.217883000	0.441367000	-3.179156000
C	-1.851836000	2.334921000	-3.817013000
C	-2.609794000	1.161776000	-3.956441000
H	-2.235864000	0.226968000	-3.548276000
C	-3.850835000	1.172244000	-4.591257000
H	-4.424391000	0.252604000	-4.664213000

C	-4.356993000	2.363621000	-5.110288000
H	-5.326092000	2.375099000	-5.603577000
C	-3.622301000	3.541647000	-4.983349000
H	-4.015753000	4.475479000	-5.379015000
C	-2.385540000	3.525393000	-4.337275000
H	-1.830439000	4.455001000	-4.228886000
C	0.272024000	4.036056000	-2.418018000
C	0.921108000	4.991441000	-3.216233000
H	1.271296000	4.714833000	-4.208389000
C	1.143478000	6.289916000	-2.754190000
H	1.648327000	7.012838000	-3.391175000
C	0.728144000	6.655780000	-1.474018000
H	0.905499000	7.664886000	-1.109530000
C	0.090151000	5.719358000	-0.660387000
H	-0.231653000	5.990872000	0.341549000
C	-0.135528000	4.426686000	-1.131458000
H	-0.635237000	3.710595000	-0.483886000
C	4.420918000	-1.181121000	-1.800203000
C	3.666839000	-2.247401000	-2.312402000
H	2.697524000	-2.471665000	-1.877174000
C	4.130010000	-3.017146000	-3.379855000
H	3.520242000	-3.833848000	-3.755817000
C	5.366459000	-2.731759000	-3.957972000
H	5.732342000	-3.330331000	-4.789229000
C	6.129183000	-1.669059000	-3.471651000
H	7.089266000	-1.433580000	-3.925767000
C	5.656496000	-0.901468000	-2.407063000
H	6.254644000	-0.065063000	-2.051629000
C	4.653046000	-0.936549000	1.242261000
C	5.700242000	-1.865609000	1.138925000
H	5.990521000	-2.237712000	0.159445000
C	6.375962000	-2.325231000	2.270923000
H	7.184671000	-3.044781000	2.164214000
C	6.009543000	-1.867243000	3.535740000
H	6.533789000	-2.223741000	4.419583000
C	4.964976000	-0.950256000	3.662117000
H	4.670189000	-0.583989000	4.641840000
C	4.299127000	-0.490391000	2.526981000
H	3.497899000	0.233806000	2.644692000
C	4.373736000	1.584575000	-0.435994000
C	5.683112000	1.943585000	-0.076091000
H	6.348807000	1.195371000	0.349865000
C	6.145148000	3.249990000	-0.234632000
H	7.164626000	3.503105000	0.048339000
C	5.294999000	4.232008000	-0.743404000
H	5.650259000	5.252829000	-0.865281000
C	3.985028000	3.901187000	-1.090419000

H	3.310831000	4.658522000	-1.480479000
C	3.535455000	2.588956000	-0.941235000
H	2.512689000	2.345369000	-1.214819000
C	0.331181000	-4.369053000	-2.072366000
C	0.075211000	-3.566459000	-3.195473000
H	-0.388015000	-2.592676000	-3.058108000
C	0.413030000	-3.988647000	-4.481230000
H	0.211205000	-3.344183000	-5.333080000
C	1.015627000	-5.232505000	-4.670057000
H	1.277453000	-5.566105000	-5.671475000
C	1.291154000	-6.041380000	-3.567142000
H	1.772636000	-7.006763000	-3.706030000
C	0.955390000	-5.608808000	-2.283738000
H	1.189686000	-6.243898000	-1.431779000
C	0.942777000	-4.465631000	0.980136000
C	2.020835000	-3.687507000	1.428981000
H	2.175328000	-2.694350000	1.014328000
C	2.896899000	-4.162057000	2.405547000
H	3.721202000	-3.537596000	2.737376000
C	2.711200000	-5.433608000	2.948257000
H	3.393600000	-5.805483000	3.709322000
C	1.646568000	-6.223879000	2.516160000
H	1.492350000	-7.213054000	2.941913000
C	0.769444000	-5.739371000	1.545915000
H	-0.072986000	-6.355012000	1.237213000
C	-1.920250000	-4.643993000	-0.026601000
C	-2.375072000	-5.720052000	-0.804791000
H	-1.784303000	-6.055376000	-1.654124000
C	-3.576651000	-6.367742000	-0.514689000
H	-3.908360000	-7.198272000	-1.134066000
C	-4.353203000	-5.944486000	0.562009000
H	-5.293764000	-6.442364000	0.786621000
C	-3.923709000	-4.873318000	1.345610000
H	-4.527553000	-4.523872000	2.178311000
C	-2.719410000	-4.235436000	1.053881000
H	-2.406354000	-3.398669000	1.672821000
C	-4.447726000	-0.896519000	-1.222296000
C	-3.659247000	-1.894996000	-1.814175000
H	-2.657180000	-2.074441000	-1.433569000
C	-4.140867000	-2.660888000	-2.875539000
H	-3.510790000	-3.430719000	-3.313130000
C	-5.430487000	-2.446486000	-3.362740000
H	-5.810096000	-3.046810000	-4.186474000
C	-6.230452000	-1.458579000	-2.788523000
H	-7.234754000	-1.281473000	-3.167360000
C	-5.738358000	-0.688898000	-1.733753000
H	-6.364328000	0.095494000	-1.313119000

C	-4.342598000	-0.757028000	1.859513000
C	-3.495631000	-0.769211000	2.977756000
H	-2.509414000	-0.319557000	2.904170000
C	-3.889855000	-1.358430000	4.179412000
H	-3.209278000	-1.356770000	5.026374000
C	-5.151265000	-1.943630000	4.286029000
H	-5.463601000	-2.401143000	5.222087000
C	-6.009062000	-1.945409000	3.185410000
H	-6.991271000	-2.407290000	3.259783000
C	-5.602716000	-1.363753000	1.984298000
H	-6.272867000	-1.396922000	1.127452000
C	-4.632215000	1.803539000	0.182960000
C	-4.203741000	2.752079000	-0.760963000
H	-3.368965000	2.520284000	-1.416547000
C	-4.833801000	3.989689000	-0.881541000
H	-4.479441000	4.702454000	-1.621422000
C	-5.917401000	4.305114000	-0.060971000
H	-6.412964000	5.268703000	-0.155847000
C	-6.359372000	3.379267000	0.883348000
H	-7.199079000	3.619407000	1.531788000
C	-5.718352000	2.144878000	1.004369000
H	-6.066105000	1.439625000	1.755673000
C	2.045307000	2.367812000	3.774995000
C	2.753580000	3.100608000	2.808749000
H	2.353515000	3.194763000	1.802781000
C	3.976847000	3.698662000	3.106925000
H	4.511113000	4.241774000	2.332593000
C	4.515131000	3.582343000	4.388421000
H	5.470440000	4.046111000	4.623171000
C	3.829297000	2.860765000	5.364235000
H	4.246965000	2.761723000	6.363882000
C	2.609454000	2.256301000	5.056253000
H	2.091414000	1.683645000	5.822726000
C	-0.905359000	3.058597000	3.503595000
C	-0.765147000	4.056105000	4.482291000
H	0.087773000	4.031334000	5.157942000
C	-1.688882000	5.094994000	4.591205000
H	-1.561336000	5.857580000	5.356538000
C	-2.767087000	5.161886000	3.708739000
H	-3.486037000	5.974423000	3.786657000
C	-2.918187000	4.188350000	2.721737000
H	-3.751767000	4.233903000	2.026825000
C	-1.995310000	3.146173000	2.624658000
H	-2.119785000	2.393069000	1.850629000
C	-0.038583000	0.305588000	4.662588000
C	0.418455000	-1.009481000	4.474572000
H	0.951092000	-1.270078000	3.563333000

C	0.201727000	-1.996876000	5.434728000
H	0.561772000	-3.007109000	5.258312000
C	-0.476756000	-1.685272000	6.613219000
H	-0.646325000	-2.452800000	7.364836000
C	-0.942283000	-0.386762000	6.820195000
H	-1.478863000	-0.138796000	7.733343000
C	-0.727950000	0.594611000	5.850792000
H	-1.112675000	1.598545000	6.017230000

[Pr^{IV}(OSi(Ph)$_3$)$_4$(CH$_3$CN)$_2$], s=1/2, dispersion

C	5.387460000	-2.182768000	-2.469068000
C	4.050539000	-1.797036000	-2.298572000
C	3.284386000	-1.519307000	-3.439857000
C	3.835775000	-1.638276000	-4.714477000
C	5.163314000	-2.037917000	-4.867680000
C	5.941014000	-2.307293000	-3.742001000
Si	3.274697000	-1.613468000	-0.596185000
O	1.928657000	-0.671591000	-0.735713000
Pr	-0.113611000	-0.188358000	-0.808062000
N	0.304209000	0.500980000	-3.396411000
C	-0.024133000	1.562945000	-3.729360000
C	-0.454639000	2.889890000	-4.113401000
C	2.780220000	-3.308273000	0.072709000
C	3.582677000	-4.435881000	-0.150612000
C	3.222400000	-5.687406000	0.349205000
C	2.037534000	-5.831228000	1.071995000
C	1.221042000	-4.720294000	1.294505000
C	1.592242000	-3.470871000	0.802623000
C	4.467266000	-0.757888000	0.567962000
C	5.550137000	-1.409353000	1.173879000
C	6.422682000	-0.713895000	2.009752000
C	6.213661000	0.643296000	2.259321000
C	5.133632000	1.302582000	1.674409000
C	4.271141000	0.604303000	0.833352000
N	0.030247000	-2.444792000	-2.099942000
C	0.414324000	-3.528459000	-2.224384000
C	0.937640000	-4.866640000	-2.383462000
O	-0.576538000	-0.749532000	1.144863000
Si	-0.942793000	-0.573899000	2.755541000
C	-2.088972000	-1.980952000	3.241080000
C	-1.567185000	-3.223475000	3.631054000
C	-2.406885000	-4.316714000	3.839722000
C	-3.784112000	-4.179611000	3.660856000
C	-4.318599000	-2.946997000	3.287950000
C	-3.476545000	-1.856284000	3.083076000
O	-2.065624000	0.288339000	-1.445235000
Si	-3.487826000	-0.577081000	-1.525987000

C	-4.563566000	-0.103568000	-0.068657000
C	-5.633707000	-0.906443000	0.351082000
C	-6.403020000	-0.548635000	1.456101000
C	-6.094858000	0.610643000	2.171018000
C	-5.028729000	1.414778000	1.770721000
C	-4.275964000	1.060983000	0.653186000
O	0.370284000	1.784537000	-0.377908000
Si	1.012374000	3.293490000	-0.264626000
C	2.129913000	3.540740000	-1.757427000
C	2.794066000	2.422366000	-2.287159000
C	3.570135000	2.527976000	-3.438525000
C	3.700606000	3.761746000	-4.076805000
C	3.055780000	4.885696000	-3.560011000
C	2.272174000	4.772797000	-2.411226000
C	-1.773543000	1.071244000	3.126627000
C	-1.615617000	2.192140000	2.300544000
C	-2.151516000	3.429092000	2.652691000
C	-2.858490000	3.563674000	3.847689000
C	-3.038782000	2.457062000	4.677325000
C	-2.502330000	1.222890000	4.315664000
C	0.657955000	-0.701362000	3.731426000
C	1.893836000	-0.808906000	3.082082000
C	3.082444000	-0.883006000	3.805955000
C	3.047363000	-0.850847000	5.197815000
C	1.825818000	-0.739309000	5.864295000
C	0.642265000	-0.666057000	5.134013000
C	-4.272638000	-0.179622000	-3.183981000
C	-3.454675000	-0.119637000	-4.324160000
C	-3.997494000	0.150987000	-5.578118000
C	-5.368847000	0.371950000	-5.709950000
C	-6.192375000	0.322356000	-4.586399000
C	-5.645282000	0.050283000	-3.333096000
C	-3.106936000	-2.419735000	-1.413544000
C	-2.698971000	-2.924269000	-0.168388000
C	-2.316197000	-4.254445000	-0.019158000
C	-2.353274000	-5.109056000	-1.120377000
C	-2.778395000	-4.630271000	-2.361694000
C	-3.150663000	-3.294859000	-2.506567000
C	1.982537000	3.468497000	1.328766000
C	2.884736000	4.519628000	1.544231000
C	3.553323000	4.643509000	2.761818000
C	3.339522000	3.706207000	3.773706000
C	2.464123000	2.641353000	3.565888000
C	1.791132000	2.531996000	2.352626000
C	-0.453867000	4.473958000	-0.307053000
C	-0.401781000	5.778506000	0.198516000
C	-1.534649000	6.591934000	0.203859000

C	-2.743745000	6.105133000	-0.291829000
C	-2.816872000	4.803752000	-0.790167000
C	-1.681341000	3.996529000	-0.797478000
H	1.928893000	-0.806742000	1.997695000
H	4.028699000	-0.955918000	3.282833000
H	3.972731000	-0.906919000	5.764920000
H	1.797873000	-0.708784000	6.950363000
H	-0.304355000	-0.572576000	5.661417000
H	-0.493978000	-3.331642000	3.774224000
H	-1.989866000	-5.273333000	4.144189000
H	-4.439571000	-5.032136000	3.817241000
H	-5.388985000	-2.832215000	3.145492000
H	-3.901583000	-0.903022000	2.782353000
H	-1.073041000	2.099587000	1.365326000
H	-2.013885000	4.281764000	1.995149000
H	-3.271412000	4.528717000	4.129171000
H	-3.599597000	2.554788000	5.603137000
H	-2.664093000	0.363760000	4.962895000
H	-2.679370000	-2.273984000	0.698183000
H	-2.009709000	-4.614521000	0.957311000
H	-2.066161000	-6.151986000	-1.010910000
H	-2.826839000	-5.302530000	-3.214991000
H	-3.471152000	-2.924672000	-3.477235000
H	-5.852838000	-1.831720000	-0.177655000
H	-7.230818000	-1.179006000	1.769979000
H	-6.679141000	0.879968000	3.046637000
H	-4.765389000	2.302552000	2.336541000
H	-3.439200000	1.683116000	0.353928000
H	-2.385249000	-0.288439000	-4.222939000
H	-3.354431000	0.189690000	-6.453824000
H	-5.794044000	0.585244000	-6.686991000
H	-7.259982000	0.498057000	-4.686922000
H	-6.289926000	0.024729000	-2.457574000
H	0.526642000	6.153825000	0.624093000
H	-1.477440000	7.599528000	0.606734000
H	-3.629884000	6.733671000	-0.278166000
H	-3.761708000	4.409867000	-1.154708000
H	-1.759662000	2.968992000	-1.146170000
H	2.680561000	1.452468000	-1.810861000
H	4.061431000	1.644159000	-3.837088000
H	4.301951000	3.847385000	-4.977898000
H	3.156563000	5.847418000	-4.056187000
H	1.746327000	5.647544000	-2.033859000
H	3.079861000	5.237408000	0.749843000
H	4.247977000	5.464561000	2.918748000
H	3.867604000	3.799616000	4.718743000
H	2.311440000	1.887927000	4.332321000

H	1.124730000	1.693781000	2.193148000
H	-0.271875000	3.049167000	-5.179250000
H	0.118453000	3.624827000	-3.542126000
H	-1.519979000	3.003238000	-3.895850000
H	2.258510000	-1.185836000	-3.325557000
H	3.230593000	-1.411608000	-5.588486000
H	5.594339000	-2.128999000	-5.861074000
H	6.980137000	-2.604003000	-3.856112000
H	6.015008000	-2.360323000	-1.598280000
H	5.701761000	-2.475391000	1.016376000
H	7.255104000	-1.232679000	2.477669000
H	6.886397000	1.183590000	2.919774000
H	4.948602000	2.352133000	1.879266000
H	3.416485000	1.115312000	0.404036000
H	0.937442000	-2.622142000	0.979906000
H	0.293047000	-4.822734000	1.848664000
H	1.750178000	-6.805182000	1.458944000
H	3.858275000	-6.549887000	0.167895000
H	4.490260000	-4.340287000	-0.743326000
H	1.941229000	-4.814694000	-2.810502000
H	0.275350000	-5.444933000	-3.031311000
H	0.992279000	-5.339788000	-1.398322000

References

1. R. P. Kelly, L. Maron, R. Scopelliti, and M. Mazzanti, Reduction of a Cerium(III) Siloxide Complex To Afford a Quadruple-Decker Arene-Bridged Cerium(II) Sandwich, *Angew. Chem. Int. Ed.* 2017**, 56**, 15663–15666.

2.A. I. Ojeda-Amador, A. J. Martínez-Martínez, A. R. Kennedy and C. T. O'Hara, Structural Studies of Cesium, Lithium/Cesium, and Sodium/Cesium Bis(trimethylsilyl)amide (HMDS) Complexes, *Inorg. Chem.* 2016, **55**, 11, 5719–5728.

3. A. R. Willauer, C. T. Palumbo, F. Fadaei-Tirani, I. Zivkovic, I. Douair, L. Maron and M. Mazzanti, Accessing the plus IV Oxidation State in Molecular Complexes of Praseodymium, *J. Am. Chem. Soc.,* 2020, **142**, 5538–5542.

4. C. T. Palumbo, I. Zivkovic, R. Scopelliti and M. Mazzanti, Molecular Complex of Tb in the+4 Oxidation State, *J. Am. Chem. Soc.*, 2019, **141**, 9827–9831

5. R. J. LeSuer, C. Buttolph and W. E. Geiger, Comparison of the Conductivity Properties of the Tetrabutylammonium Salt of Tetrakis(Pentafluorophenyl)Borate Anion with Those of Traditional Supporting Electrolyte Anions in Nonaqueous Solvents, *Anal. Chem.* 2004, **76**, 21, 6395–6401.

6. B. Boduszek and H. J. Shine, Preparation of Solid Thianthrene Cation Radical Tetrafluoroborate, *J. Org. Chem.* 1988**, 53**, 5142–5143.

7. G. A. Bain, and J. F. Berry, Diamagnetic Corrections and Pascal's Constants. *J. Chem. Educ.* 2008**, 85**, 4, 532.

8. CrysAlisPro Software System, Rigaku Oxford Diffraction, (2024).

9. G.M. Sheldrick, ShelXT-Integrated space-group and crystal-structure determination, *Acta Cryst.*, 2015, **A71**, 3–8.

10. O.V. Dolomanov and L.J. Bourhis and R.J. Gildea and J.A.K. Howard and H. Puschmann, Olex2: A complete structure solution, refinement and analysis program, *J. Appl. Cryst.*, 2009, **42,** 339–341.

11. G.M. Sheldrick, Crystal structure refinement with ShelXL, *Acta Cryst.*, 2015, **C71**, 3–8.

12. A. D. Becke, J. Chem. Phys., 1993, **98**, 5648; A.D. Becke, *Phys. Rev. A.* 1993, **48**, 6, 3098; J.P. Perdew, Y. Wang, *Phys. Rev. B,* 1992, **45**, 23, 13244.

13. D. Andrae, U. Häussermann, M. Dolg, H. Stoll and H. Preuss, Theor. Chim. Acta, 1990, **77**, 123; J. M. L. Martin and A. Sundermann, *J. Chem. Phys.,* 2001, **114**, 3408; M. Dolg, H. Stoll, A. Savin, H. Preuss, *Theor. Chim. Acta* 1989, **75**, 173; M. Dolg, H. Stoll, H. Preuss, *Theor. Chim. Acta* 1993, **85**, 441.

14. A. Hollwarth, M. Bohme, S. Dapprich, A.W. Ehlers, A. Gobbi, V. Jonas, K.F. Kohler, R. Stegmann, A. Veldkamp, G. Frenking *J. Chem. Phys.* 1993, **208**, 237.

15. R. Ditchfield, W. J. Hehre and J. A. Pople, *J. Chem. Phys.*, 1971, **54**, 724; W. J. Hehre, R. Ditchfield and J. A. Pople, *J. Chem. Phys.,* 1972, **56**, 2257; P. C. Hariharan and J. A. Pople, *Theor. Chem. Acc.*, 1973, **28**, 213.

16. S. Grimme, S. Ehrlich, L. Goerigk, J. *Comp. Chem.,* 2011, **32**, 1456.

17. Gaussian 09, Revision D.01: M. J. Frisch, G. W. Trucks, H. B. Schlegel, G. E. Scuseria, M. A. Robb, J. R. Cheesman, G. Scalmani, V. Barone, B. Mennucci, G. A. Petersson, H. Nakatsuji, M. Caricato, X. Li, H. P. Hratchian, A. F. Izmaylov, J. Bloino, G. Zheng, J. L. Sonnenberg, M. Hada, M. Ehara, K. Toyota, R. Fukuda, J. Hasegawa, M. Ishida, T. Nakajima, Y. Honda, O. Kitao, H. Nakai, T. Vreven, J. A., Jr. Montgomery, J. E. Peralta, F. Ogliaro, M. Bearpark, J. J. Heyd, E. Brothers, K. N. Kudin, V. N. Staroverov, R. Kobayashi, J. Normand, K. Raghavachari, J. C. Burant, S. S. Iyengar, J. Tomasi, M. Cossi, N. Rega, M. J. Millam, M. Klene, J. E. Knox, J. B. Cross, V. Bakken, C. Adamo, J. Jaramillo, R. Gomperts, R. E. Stratmann, O. Yazyev, A. J. Austin, R. Cammi, C. Pomelli, J. W. Ochterski, R. L. Martin, K. Morokuma, V. G. Zakrzewski, G. A. Voth, P. Salvador, J. J. Dannenberg, S. Dapprich, A. D. Daniels, O. Farkas, J. B. Foresman, J. V. Ortiz, J. Cioslowski and D. J. Fox, Gaussian Inc., 2009, Wallingford CT.

18. NBO 6.0. E. D. Glendening, J. K. Badenhoop, A. E. Reed, J. E. Carpenter, J. A. Bohmann, C. M. Morales, C. R. Landis, and F. Weinhold (Theoretical Chemistry Institute, University of Wisconsin, Madison, WI, 2013); http://nbo6.chem.wisc.edu/.
